# Causal association between 3 adiposity indices and 5 infectious diseases: A Mendelian randomization study

**DOI:** 10.1097/MD.0000000000045775

**Published:** 2025-11-07

**Authors:** Xiaohong Chen, Jia Yin, Chao Chen, Tao Xiang

**Affiliations:** a Department of Emergency, Affiliated Hospital of Southwest Jiaotong University, The Third People’s Hospital of Chengdu, Chengdu, Sichuan, China; b Department of Thyroid Surgery, Sichuan Provincial People’s Hospital, University of Electronic Science and Technology of China, Chengdu, China.

**Keywords:** adiposity indices, body mass index, hip circumference, infections, Mendelian randomization, waist circumference

## Abstract

Previous studies on the association between adiposity indices and risk of infection have been inconsistent. To investigate the causality between different adiposity indices (body mass index [BMI], waist circumference [WC], and hip circumference [HC]) and the risk of infection (sepsis susceptibility (SS), sepsis mortality (SM), cholecystitis, intestinal infections, infections of the skin and subcutaneous tissue [SSTI], and acute lower respiratory infections). Mendelian randomization (MR) analysis was conducted to explore the causality between the 3 adiposity indices and the risk of 5 infectious diseases. Twenty-eight MR analyses were conducted to evaluate the final results, comprising 18 forward univariate MR, 5 reverse univariate MR, and 5 multivariable MR (MVMR) analyses. Data on adiposity indices and infections were obtained from the UK Biobanks, FinnGen Biobanks, Medical Research Council Integrative Epidemiology Unit, and within the family genome-wide association study consortium. Preliminary genetic variants associated with BMI (n = 11), WC (n = 374), and HC (n = 420) were selected as instrumental variables. The inverse-variance weighted (IVW) method combined with different types of MR methods was used to enhance the robustness of the final results. The statistical significance threshold was corrected using the Bonferroni method. The MVMR-IVW (random) analysis revealed that only WC had a significant and independent causal association with SS (odds ratio [OR] = 1.95; 95% confidence interval [CI]: 1.41–2.69; *P* < .001), SM (OR = 3.23; 95% CI: 1.52–6.86; *P* = .002), cholecystitis (OR = 1.87; 95% CI: 1.36–2.55; *P* < .001), and SSTI (OR = 1.74; 95% CI: 1.26–2.41; *P* = .001). These results were confirmed by other MVMR methods (least absolute shrinkage and selection operator regression, and MVMR-robust). Neither pleiotropy nor reverse causality was detected. WC may predict infectious disease risk more efficiently than other indices, and controlling WC may help decrease the risk of infectious diseases. Future long-term prospective studies are needed to explore the associations between dynamic adiposity indices and diverse infectious diseases in different populations.

## 1. Introduction

Body mass index (BMI) is the adiposity index recommended by the World Health Organization for assessing normal body weight.^[1]^ Consequently, it is extensively used in clinical practice to evaluate or predict an individual’s health and is the most commonly employed adiposity index in scientific research. However, prior studies that utilized BMI as the adiposity index to investigate the relationship between body weight and infection yielded perplexing results, known as the “obesity paradox.” This paradox posits that obesity elevates the risk and severity of infections yet paradoxically lowers mortality from such infections.^[2,3]^ Notably, this paradox has seldom been observed in studies using waist circumference (WC) as a diagnostic criterion for obesity.^[4,5]^ BMI is a measure of general obesity but cannot differentiate between skeletal muscle, adipose tissue, and other body components. Consequently, it may provide misleading insights into disease risk reduction.^[6]^ WC reflects abdominal obesity and demonstrates a stable association with various health conditions, particularly cardiovascular diseases and multimorbidity.^[7,8]^ This discrepancy highlights the fundamental differences between these two adiposity indices. Therefore, comparisons between BMI and WC are needed to establish evidence-based clinical risk stratification for infectious diseases.

Numerous observational studies have compared the relationship between BMI, WC, and infection risk; however, these findings remain inconclusive and debatable. Some observational studies have found that both BMI and WC can predict the risk of coronavirus disease 2019 (COVID‑19), influenza, and bacterial infection.^[9,10]^ However, Salman et al suggested that neither BMI nor WC was associated with COVID‑19 severity.^[11]^ Traditional observational studies have limitations in terms of controlling variables and addressing reverse causality, which limits the quality and reliability of the findings.^[12]^ A multicenter randomized controlled trial (RCT) indicated that WC, but not BMI, was linked to sepsis risk^[13]^; nonetheless, its generalizability may be restricted to specific populations, such as those undergoing major abdominal surgery. Therefore, current evidence from both observational studies and RCTs is insufficient to determine which adiposity index has a causal relationship with infectious diseases.

Mendelian randomization (MR) offers an alternative approach to traditional observational studies and RCTs for assessing causal relationships using single-nucleotide polymorphisms (SNPs) as genetic instrumental variables (IV). Owing to the random allocation of genetic variants at conception, MR studies are less prone to bias than are conventional observational studies. Additionally, MR studies can leverage existing genetic data to provide solutions to common practical and ethical issues in RCTs. Given that available observational studies on different adiposity indices and infectious diseases remain inconclusive and debatable, and that RCTs have limited generalizability, an MR study was conducted to investigate the causal effects of adiposity indices on various infections.

## 2. Materials and methods

### 2.1. Study design

This study was conducted in accordance with the strengthening the reporting of observational studies in epidemiology guidelines.^[14]^ Bidirectional and multivariable MR (MVMR) analyses were conducted to explore the causality between the 3 adiposity indices and 5 infectious diseases. To address population stratification, this study focuses on individuals of European ancestry. Importantly, there was no significant sample overlap between the exposures and outcomes.

In the forward MR analysis, the exposure variables included 3 common adiposity indices: BMI, hip circumference (HC), and WC. BMI was calculated as weight (kg) divided by height squared (m^2^). The HC was measured at the widest part of the hip. WC was measured at the midpoint between the lowest rib and iliac crest, using a measuring tape that was snug but not compressing the skin. The outcomes included 5 infectious diseases that may be associated with obesity or adiposity indices in previous studies: sepsis,^[15]^ cholecystitis,^[16]^ intestinal infections (II),^[17]^ skin and subcutaneous tissue infection (SSTI),^[18]^ and acute lower respiratory infection (ALRI).^[19]^ Russell et al proposed that the causal relationship between exposure and sepsis is more robust when exposure is associated with both sepsis susceptibility (SS) and sepsis mortality (SM) because sepsis is a severe disease that can lead to death.^[20]^ Therefore, the present study examined the association between each adiposity index, SS and SM. The outcome diagnoses were based on hospital episode statistical codes.

If multiple adiposity indices showed causal associations with the same outcome in the forward univariate two-sample MR (TSMR) analyses, an MVMR analysis was conducted to assess the independent causal relationships of each adiposity index with the outcome. If an adiposity index had an independent causal relationship with an outcome, a reverse TSMR analysis was used to explore the possibility of reverse causality.

### 2.2. Genetic association datasets for adiposity indices

Summary-level genome-wide association study (GWAS) results for BMI were obtained from the latest dataset of the within-family GWAS consortium, including 51,852 European ancestries. The within-family GWAS consortium is a collaborative organization of international researchers aiming to advance GWAS by studying the impact of genetic factors within families on complex human diseases. Summary-level GWAS datasets for WC (462,166 individuals with 9,851,867 SNP) and HC (462,117 individuals with 9,851,867 SNP) were obtained from the Medical Research Council Integrative Epidemiology Unit, which has an openly available dataset with scalable and high-performance cloud-based data for the scientific community.

### 2.3. Genetic association datasets for infectious diseases

Summary-level GWAS data on SS and SM were obtained from the UK Biobank, with adjustments for age and sex. The UK Biobank is an open dataset that includes health-related data of more than 500,000 participants.^[21]^ The GWAS datasets for SS and SM included 486,484 individuals (11,643 cases and 474,841 controls) and 486,484 individuals (1896 cases), respectively. Summary-level GWAS data on patients with ALRI (218,792), II (201,463), SSTI (218,792), and cholecystitis (215,027) were obtained from the FinnGen Biobank, a large-scale research project in Finland that aims to create a unique and comprehensive dataset by combining genomic, health, and lifestyle information from 500,000 individuals.^[22]^

Further information is summarized in Table S1, Supplemental Digital Content https://links.lww.com/MD/Q599 (the datasets are accessible at https://gwas.mrcieu.ac.uk/). Ethical approval was not required for this study, as has been obtained in previous individual studies. Written informed consent for participation was not required in accordance with national legislation and institutional requirements.

### 2.4. Instrumental variable selection

Each genetic variant applied as an IV to estimate the causal effect must meet the following assumptions.^[23]^

1. Relevance hypothesis: IV is associated with exposure. The significance threshold was set at *P* < 5E−08. If no SNP were available, the exposure threshold was increased by 10 until at least one SNP was available. If the *F*-statistic was >10, the MR result was considered strong enough to avoid bias caused by weak IV^[24]^:

$F=\frac{N-k-1}{k}\times \frac{R^{2}}{1-R^{2}}$, where *R*^2^ is the number of IVs representing exposure, *k* is the number of SNPs selected, and *N* is the exposure sample size. SNPs with an *F*-statistic < 10 were deleted.

1. Independence hypothesis: IV is not associated with confounders. IVs were screened to ensure that they were not associated with confounders. To maintain independence, this study excluded SNPs identified using PhenoScanner v2 (https://github.com/PheWAS/phenoscanner) that were associated with atrial fibrillation, atrial flutter, and hyperglycemia, which have been previously shown to be associated with the risk of infectious diseases.^[25,26]^
2. Exclusion hypothesis: IV affects the outcomes only through exposure. Therefore, SNPs associated with outcomes were deleted. The significance threshold was set at *P* < .05.

### 2.5. Linkage disequilibrium

Linkage disequilibrium (LD) can cause bias in MR analysis because of the effects of a single genetic variant on multiple correlated genes or loci.^[27]^ To limit the effect of LD, SNPs with LD were excluded and clumped at *r*^2^ < 0.001 and kb = 10,000.

### 2.6. Outliers and harmonization

Outliers can have a significant impact on MR studies by biasing the estimation of the causal effects.^[28]^ Therefore, outliers detected using the MR pleiotropy residual sum and outlier (MR-PRESSO) approach were excluded.

Estimates of the effect of SNPs for exposures were harmonized with those for outcomes, for each exposure-outcome pair. SNP were excluded if there were no matching SNP as IV in the outcome summary datasets.

### 2.7. Power calculation

Power was calculated according to the following formula (https://shiny.cnsgenomics.com/mRnd/). A power of >80% was considered sufficient for the analysis.^[29]^ It included the total sample size for exposure, type-I error rate (α = 0.05), *K* (proportion of cases in the study), odds ratio (true OR of the outcome variable per standard deviation of the exposure variable), and *R*^2^ (proportion of variance explained for the association between SNPs and exposure).

## 3. Statistical analyses

### 3.1. Univariate TSMR analysis

If only one SNP is available, Wald ratio is the only MR method that can be used to explore the causal association between exposure and outcome.^[30]^ If ≥2 SNPs were available, the inverse-variance weighted (IVW) method was employed as the primary analysis owing to its precise estimation, with the random-effects model used when heterogeneity was statistically significant and the fixed-effects model used when it was not.^[31]^ The maximum likelihood (ML) method provides estimates that are accurate and stable with low variance when the sample size is large enough.^[32]^ The penalized weighted median (PWM) estimator is a robust estimator that is less sensitive to outliers than other methods, even when significant heterogeneity exists.^[33]^ Therefore, the ML and PWM analyses were performed as complementary analyses.

### 3.2. MVMR analysis

MVMR-IVW analysis was performed as the primary analysis.^[34]^ The least absolute shrinkage and selection operator (LASSO) regression provides an accurate estimation, even with moderate-to-high levels of pleiotropy, and can help identify true causal variants and avoid false positive results.^[35]^ The MVMR-robust method provides precise estimates in nearly all scenarios, including those with high levels of pleiotropy, while also correcting for type-I error rates.^[35]^ Therefore, LASSO regression and MVMR-robust were used as complementary analyses.

## 4. Sensitivity analysis

Cochran *Q* statistic was used to test SNP heterogeneity based on IVW and MR-Egger analyses.^[28]^ In MR analysis, heterogeneity refers to the presence of variability or differences in the causal estimates obtained from different genetic variants used as IV. This variability can arise from various factors, such as the effect size of the genetic variant, underlying biological mechanisms, or populations being studied. The MR-Egger method was used to test pleiotropy.^[36]^ Pleiotropy is an important consideration in MR analysis because it can affect the stability and generalizability of the causal inference results. In the MR analysis, if an intercept significantly different from zero suggests the presence of directional pleiotropy, which is a genetic variant that influences exposures and outcomes independently, then the independence hypotheses and exclusion will not be met, ultimately affecting the reliability of the results.

All data were analyzed using the “TwoSampleMR,” “MR-PRESSO,” “MendelianRandomization,” and “MVMR” packages in R software 4.0.5 (R Foundation for Statistical Computing).

## 5. Results

### 5.1. Forward MR analysis of adiposity indices on infectious diseases

#### 5.1.1. Genetic instrumental variables

**Figure 1.**
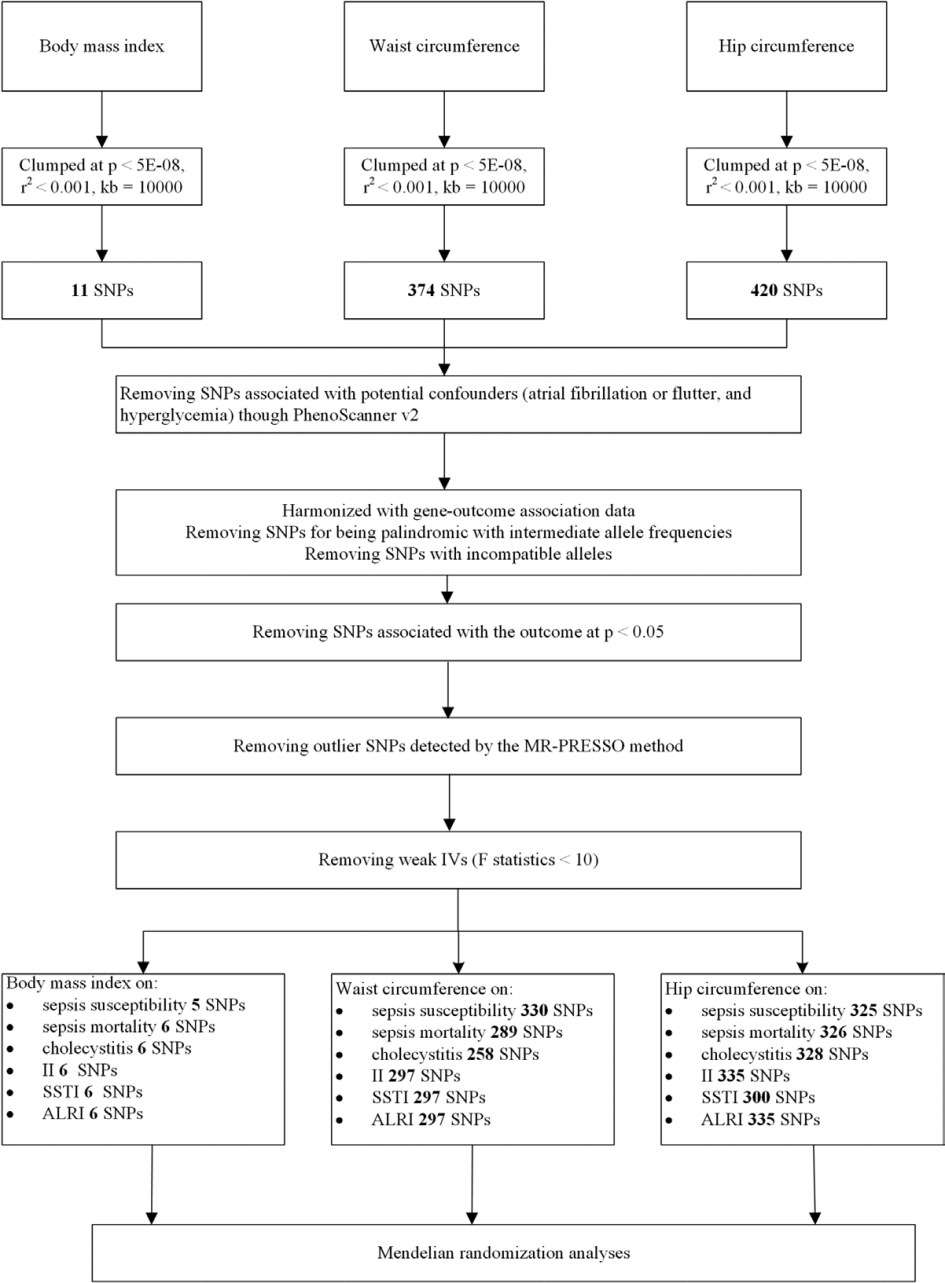
Instrumental variant selection and analysis flowchart. ALRI = acute lower respiratory infections, II = intestinal infections, MR = Mendelian randomization, MR-PRESSO = MR pleiotropy residual sum and outlier, SNP = single-nucleotide polymorphisms, SSTI = skin and subcutaneous tissue infection.

The IV selection flowchart is shown in Figure 1. After strict screening, 5 SNPs were selected as the IVs for BMI on SS, and 6 SNPs were selected as the IVs for BMI on the risk of SM, cholecystitis, II, SSTI, and ALRI. The mean *F*-statistics of the IVs for the BMI were >36. The number of SNPs as IVs for WC on the risk of SS, SM, cholecystitis, II, SSTI, and ALRI was 330, 289, 258, 297, 297, and 297, respectively. The mean *F*-statistics of the IVs for WC were >22. The number of SNPs as IVs for HC on the risk of SS, SM, cholecystitis, II, SSTI, and ALRI was 325, 326, 328, 335, 300, and 335, respectively. The mean *F*-statistics of the IVs for HC were more than 29. Detailed information on the deleted SNPs is provided in Table S2, Supplemental Digital Content, https://links.lww.com/MD/Q599, and detailed information on the effect of SNPs of each adiposity index on infectious diseases is displayed in Tables S3–20, Supplemental Digital Content, https://links.lww.com/MD/Q599.

Moreover, *P* < .003 (0.05/18 [18 forward TSMR analyses]) indicated a significant causal association and .003 ≤ *P* < .05, indicating a potential causal association.

#### 5.1.2. Univariate TSMR results

The IVW method revealed significant causal relationships between BMI, HC, WC, and cholecystitis and SSTI risk. Notably, each 1-unit increase in BMI, HC, and WC was associated with 9% (OR = 1.09; 95% confidence interval [CI]: 1.04–1.34; *P* = 5.72E−05), 55% (OR = 1.55; 95% CI: 1.42–1.68; *P* = 9.18E−23), and 51% (OR = 1.51; 95% CI: 1.35–1.68; *P* = 2.17E−13) increases in cholecystitis risk, respectively, and 10% (OR = 1.10; 95% CI: 1.04–1.15; *P* = 5.41E−04), 23% (OR = 1.23; 95% CI: 1.11–1.36; *P* = 5.27E−05), and 55% (OR = 1.55; 95% CI: 1.38–1.75; *P* = 9.65E−13) increases in SSTI risk, respectively. Both HC and WC demonstrated significant causal relationships with SS and SM risk. Each 1-unit increase in HC and WC was associated with 40% (OR = 1.40; 95% CI: 1.27–1.54; *P* = 1.21E−11) and 75% (OR = 1.75; 95% CI: 1.57–1.95; *P* = 3.83E−24) increases in SS risk, respectively, and 44% (OR = 1.44; 95% CI: 1.16–1.78; *P* = .001) and 98% (OR = 1.98; 95% CI: 1.50–2.61; *P* = 3.86E−24) increases in SM risk, respectively. Moreover, BMI exhibited a potential causal association with SS and SM risks, whereas HC and WC showed potential causal associations with ALRI risk. No causal associations were found between the II and BMI, HC, or WC. These findings were further corroborated by the ML and PWM methods, except for the association between WC and ALRI, where the PWM method yielded an OR of 1.22 (95% CI: 0.99–1.50; *P* = .056).

**Figure 2.**
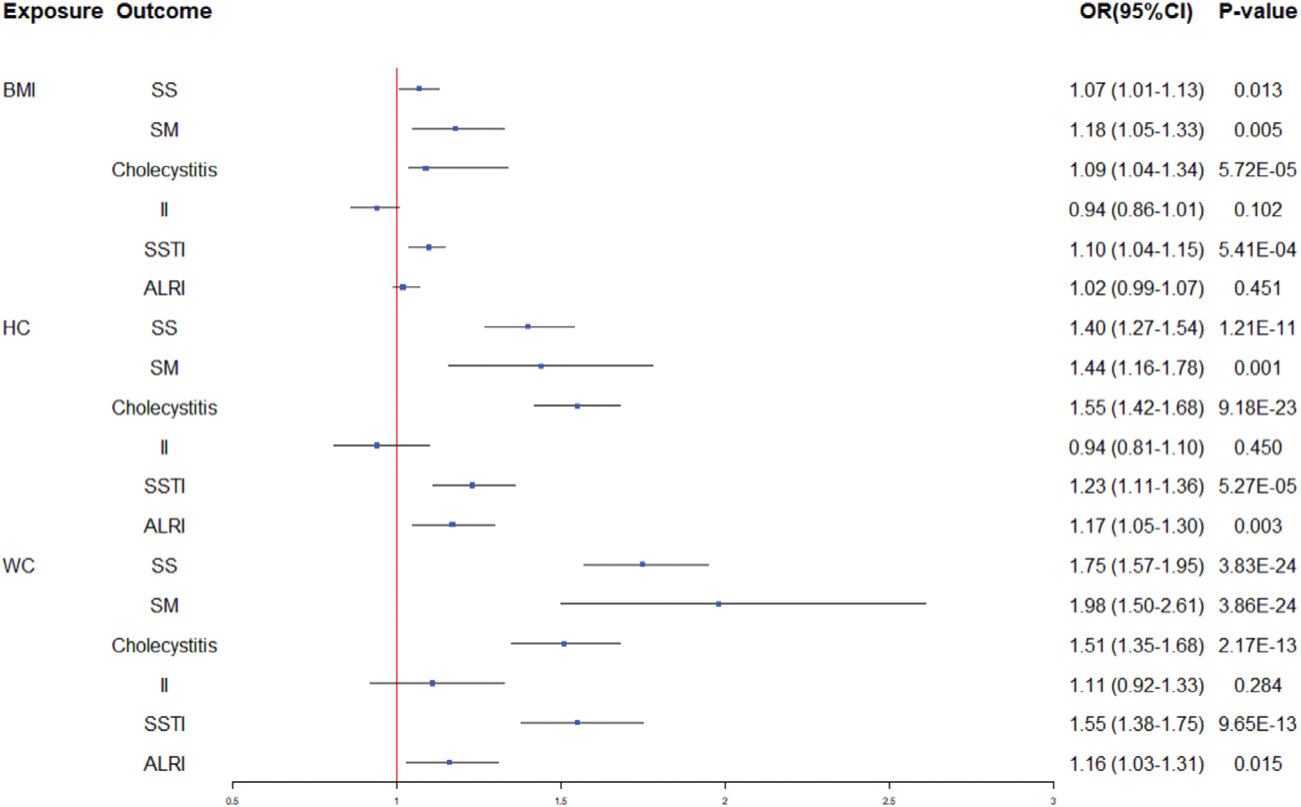
Univariate two-sample Mendelian randomization analysis of the association between adiposity indices and infectious diseases. ALRI = acute lower respiratory infection, BMI = body mass index, CI = confidence interval, HC = hip circumference, II = intestinal infections, OR = odds ratio, SM = sepsis mortality, SS = sepsis susceptibility, SSTI = skin and subcutaneous tissue infection, WC = waist circumference.

Except for some heterogeneity found in the MR analysis of the relationship between HC and SS, cholecystitis, and ALRI risk, no other heterogeneity or directional pleiotropy was observed. Notably, the relationships between HC and WC and SS, SM, cholecystitis, ALRI, and SSTI had sufficient power to explore causal associations (Table 1 and Fig. 2). Figure S1, Supplemental Digital Content, https://links.lww.com/MD/Q599 illustrates the associations between BMI and 6 infectious disease outcomes using scatter plots, funnel plots, forest plots, and leave-one-out sensitivity analyses (a1–a4, b1–b4, c1–c4, d1–d4, e1–e4, and f1–f4). Similarly, Figures S2 and 3, Supplemental Digital Content, https://links.lww.com/MD/Q599 present HC and WC associations with the same outcomes using scatter plots and funnel plots (a1–a2, b1–b2, c1–c2, d1–d2, e1–e2, and f1–f2, respectively).

**Table 1:** Mendelian randomization analysis of the association between adiposity indices and infectious diseases.

Exposure	Outcome	No. of SNP (*F*-statistic)	*R* ^2^	Power* (%)	Methods	Odds ratio (95% CI)	*P*-value**	Heterogeneity (*P*-value)***		Pleiotropy (MR-Egger)	
								IVW	MR-Egger	Intercept	*P*-value*
BMI	SS	5 (49.16)	0.004719	5	IVW-fixed	1.07 (1.01–1.13)	**.013**	.93	.85	0.02	.81
					ML	1.07 (1.01–1.13)	**.013**				
					PWM	1.07 (1.00–1.13)	**.040**				
	SM	6 (44.23)	0.005092	5	IVW-fixed	1.18 (1.05–1.33)	**.005**	.37	.62	0.05	.17
					ML	1.18 (1.05–1.34)	**.005**				
					PWM	1.17 (1.02–1.35)	**.029**				
	Cholecystitis	6 (42.58)	0.004904	7	IVW-fixed	1.09 (1.04–1.34)	**5.72E−05**	.58	.52	0.02	.51
					ML	1.09 (1.04–1.34)	**7.42E−05**				
					PWM	1.09 (1.04–1.34)	**1.29E−03**				
	II	6 (42.58)	0.004904	5	IVW-fixed	0.94 (0.86–1.01)	.102	.82	.71	0.03	.76
					ML	0.94 (0.86–1.01)	.102				
					PWM	0.94 (0.85–1.03)	.184				
	SSTI	6 (42.58)	0.004903	6	IVW-fixed	1.10 (1.04–1.15)	**5.41E−04**	.35	.87	0.02	.11
					ML	1.10 (1.04–1.16)	**5.98E−04**				
					PWM	1.08 (1.01–1.15)	**.028**				
	ALRI	6 (36.38)	0.004193	5	IVW-fixed	1.02 (0.99–1.07)	.451	.89	.87	0.02	.57
					ML	1.02 (0.97–1.07)	.451				
					PWM	1.03 (0.97–1.07)	.339				
WC	SS	330 (22.83)	0.016054	100	IVW-fixed	1.75 (1.57–1.95)	**3.83E−24**	.40	.43	2.47E−03	.11
					ML	1.76 (1.58–1.97)	**3.87E−24**				
					PWM	1.71 (1.41–2.06)	**3.30E−08**				
	SM	289 (26.08)	0.016054	100	IVW-fixed	1.98 (1.50–2.61)	**3.86E−24**	.38	.40	9.14E−03	.14
					ML	2.00 (1.51–2.65)	**1.15E−06**				
					PWM	1.84 (1.09–3.11)	**.022**				
	Cholecystitis	258 (24.19)	0.013332	100	IVW-fixed	1.51 (1.35–1.68)	**2.17E−13**	.89	.89	2.51E−03	.09
					ML	1.51 (1.35–1.69)	**1.92E−13**				
					PWM	1.68 (1.41–1.99)	**5.27E−09**				
	II	297 (26.69)	0.016875	28	IVW-fixed	1.11 (0.92–1.33)	.284	.08	.08	5.52E−03	.21
					ML	1.11 (0.92–1.34)	.282				
					PWM	0.99 (0.72–1.36)	.960				
	SSTI	297 (26.69)	0.016875	100	IVW-fixed	1.55 (1.38–1.75)	**9.65E−13**	.23	.22	2.76E−03	.99
					ML	1.55 (1.38–1.76)	**1.44E-12**				
					PWM	1.44 (1.16–1.79)	**9.57E−04**				
	ALRI	297 (26.69)	0.016875	84	IVW-fixed	1.16 (1.03–1.31)	**.015**	.12	.13	2.79E−03	.15
					ML	1.16 (1.03–1.31)	**.015**				
					PWM	1.22 (0.99–1.50)	.056				
HC	SS	325 (29.20)	0.020134	100	IVW-random	1.40 (1.27–1.54)	**1.21E−11**	.02	.02	2.90E−04	.91
					ML	1.41 (1.27–1.55)	**2.01E−13**				
					PWM	1.45 (1.24–1.69)	**1.93E−06**				
	SM	326 (29.16)	0.020169	75	IVW-fixed	1.44 (1.16–1.78)	**.001**	.06	.05	5.90E−03	1.00
					ML	1.44 (1.16–1.80)	**.001**				
					PWM	1.63 (1.12–2.37)	**.012**				
	Cholecystitis	328 (29.49))	0.020516	100	IVW-random	1.55 (1.42–1.68)	**9.18E−23**	.005	.005	−1.07E−03	.63
					ML	1.54 (1.42–1.67)	**4.91E−27**				
					PWM	1.56 (1.37–1.77)	**6.68E−12**				
	II	335 (29.37)	0.020858	13	IVW-fixed	0.94 (0.81–1.10)	.450	.93	.93	3.80E−03	.35
					ML	0.94 (0.81–1.10)	.448				
					PWM	0.92 (0.72–1.18)	.527				
	SSTI	300 (29.72)	0.018939	99	IVW-fixed	1.23 (1.11–1.36)	**5.27E−05**	.98	.98	3.10E−03	.23
					ML	1.23 (1.11–1.37)	**5.36E−05**				
					PWM	1.24 (1.01–1.47)	**.010**				
	ALRI	335 (29.36)	0.020858	94	IVW-random	1.17 (1.05–1.30)	**.003**	.02	.02	8.93E−05	.97
					ML	1.17 (1.06–1.29)	**.001**				
					PWM	1.21 (1.03–1.41)	**.018**				

*R*^2^ refers to the proportion of variability (variance) in the dependent variable that can be explained or predicted by the independent variable. Bold values indicate statistically significant differences (*P* < .05).

ALRI = acute lower respiratory infection, BMI = body mass index, HC = hip circumference, II = intestinal infections, IVW = inverse-variance weighting, ML = maximum likelihood, MR = Mendelian randomization, PWM = penalized weighted median, SM = sepsis mortality, SNP = single-nucleotide polymorphisms, SS = sepsis susceptibility, SSTI = infections of the skin and subcutaneous tissue, WC = waist circumference.

* The power, calculated with a significance level (α) of .05, can be computed using the method available on the web application at https://shiny.cnsgenomics.com/mRnd/.

** Applying the Bonferroni method, *P* <.003 (.05/18) was considered significant, and 18 was the number of times forward univariate two-sample MR was performed. A *P*-value between .003 and .05 was considered potentially significant.

*** *P* <.05 was considered significant.

#### 5.1.3. MVMR results

**Figure 3.**
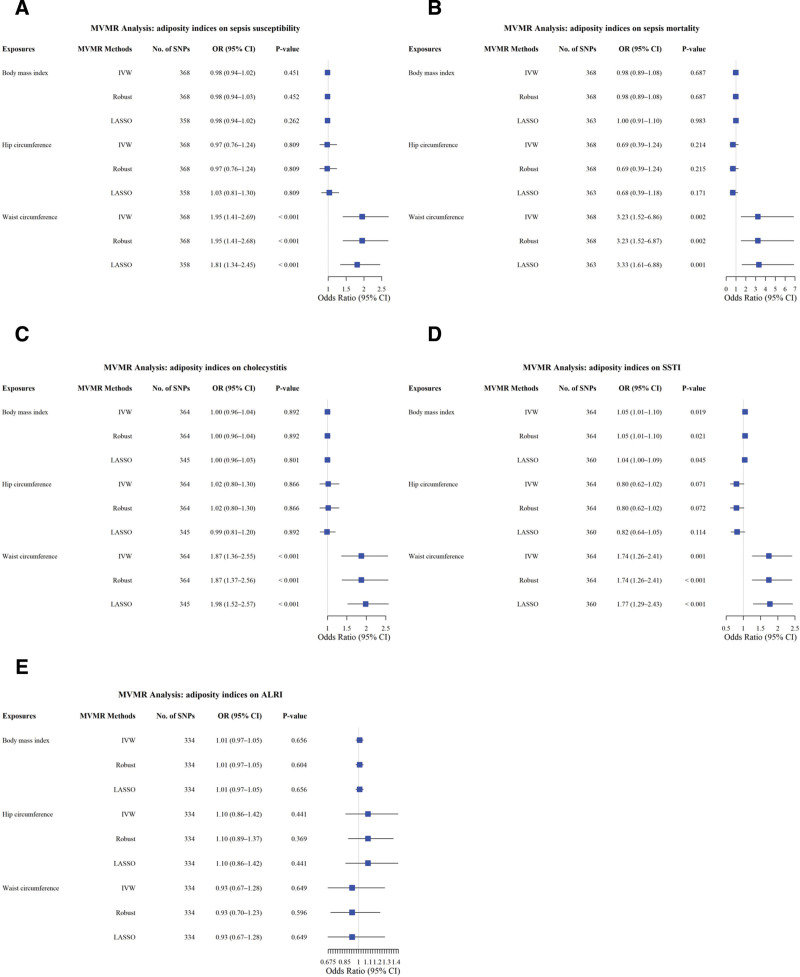
Multivariable Mendelian randomization analysis of the association between adiposity indices and infectious diseases. ALRI = acute lower respiratory infection, BMI = body mass index, CI = confidence interval, HC = hip circumference, IVW = inverse-variance weighted, LASSO = least absolute shrinkage and selection operator, OR = odds ratio, SNP = single-nucleotide polymorphism, SSTI = skin and subcutaneous tissue infection, WC = waist circumference.

In the MVMR analysis, *P* < .01 (0.05/5 [five MVMR analyses]) indicated a significant causal association. The MVMR-IVW (random) analysis revealed a significant and independent causal association between WC and SS (OR = 1.95; 95% CI: 1.41–2.69; *P* < .001), SM (OR = 3.23; 95% CI: 1.52–6.86; *P* = .002), cholecystitis (OR = 1.87; 95% CI: 1.36–2.55; *P* < .001), and SSTI (OR = 1.74; 95% CI: 1.26–2.41; *P* = .001), after controlling for the effects of BMI and HC. However, the association between BMI or HC and infectious diseases decreased dramatically after controlling for the effects of the other 2 adiposity indices. WC, HC, and BMI showed no an independent significant causal association with ALRI. All results were confirmed using MVMR-robust and LASSO methods (Fig. 3A–E). Heterogeneity was observed in the MVMR analyses of the associations between adiposity indices and cholecystitis and SS, whereas no associations were observed between adiposity indices and ALRI, SSTI, and SM. No pleiotropy (MVMR-Egger test) was observed (Table 2).

**Table 2:** Sensitivity analyses for multivariable Mendelian randomization analysis of the association between adiposity indices and infectious diseases.

Outcome	Heterogeneity *P*-value		Pleiotropy (MVMR-Egger)	
	MVMR-IVW	MVMR-Egger	Intercept	*P*-value
ALRI	1.000	1.000	−0.001	.609
Cholecystitis	<.001	<0.001	−0.001	.485
SSTI	.301	0.289	<0.001	.773
Sepsis susceptibility	.012	0.011	<0.001	.818
Sepsis mortality	.131	0.123	<0.001	.947

ALRI = acute lower respiratory infection, IVW = inverse-variance weighting, MVMR = multivariable Mendelian randomization, SSTI = infections of the skin and subcutaneous tissue.

### 5.2. Reverse MR analysis of infectious diseases on WC

The MVMR analysis revealed an independent causal relationship between WC and SS, SM, cholecystitis, and SSTI. Subsequently, reverse MR analysis was performed.

SNPs associated with SS, SM, SSTI, and ALRI were selected at a *P* < 5E−06 threshold because no SNP could be selected at a *P* < 5E−08 or *P* < 5E−07 threshold. SNPs associated with cholecystitis were selected at a *P* < 5E−08 threshold. All SNPs were clustered at *r*^2^ < 0.001 and kb = 10,000. A total of 14, 16, 30, 11, and 13 SNPs were significantly associated with SS, SM, cholecystitis, SSTI, and ALRI, respectively. After strict screening of the remaining SNPs, the IVs for SS, SM, SSTI, and ALRI in WC were rs3851566 (*F*-statistic = 10.36), rs34209645 (*F*-statistic = 10.86), rs6761459 (*F*-statistic = 11.88), and rs685341 (*F*-statistic = 10.34), respectively. Additionally, 24 SNPs were selected as IVs for the MR analysis of cholecystitis on WC, with a mean *F*-statistics of 31.21 (Table S21, Supplemental Digital Content, https://links.lww.com/MD/Q599. Detailed information on the deleted SNPs is provided in Table S2, Supplemental Digital Content, https://links.lww.com/MD/Q599.

The reverse MR analysis showed that no infectious diseases were associated with WC (Table 3). Because a single SNP served as the IV for SS, SM, SSTI, and ALRI, it was not possible to conduct Cochran *Q* test or MR-Egger test to detect heterogeneity and pleiotropy, nor was it possible to generate visualization plots. For MR analysis of cholecystitis based on WC, heterogeneity was detected using Cochran *Q* test (IVW, *P* = .009; MR-Egger, *P* = .03); however, no significant pleiotropy was observed (MR-Egger, intercept = 0.0017, *P* = .069).

**Table 3:** Mendelian randomization analysis of the association between infectious diseases and waist circumference.

Exposure	No. of SNPs (*F*-statistic)	*R* ^2^	Methods	β (95% CI)	*P*-value
Sepsis susceptibility	1 (10.36)	2.13E−05	Wald ratio	−0.03 (−0.09–0.02)	.256
Sepsis mortality	1 (10.86)	2.23E−05	Wald ratio	−0.01 (−0.03–0.01)	.292
Cholecystitis	24 (31.21)	0.003471	IVW-random	8.50E−04 (−8.58E−04, 0.0069)	.829
SSTI	1 (11.88)	5.43E−05	Wald ratio	−0.01 (−0.06–0.04)	.606
ALRI	1 (10.34)	4.73E−05	Wald ratio	−0.01 (−0.04–0.07)	.607

*R*^2^ refers to the proportion of variability (variance) in the dependent variable that can be explained or predicted by the independent variable.

ALRI = acute lower respiratory infection, CI = confidence interval, IVW = inverse-variance weighting, SNP = single-nucleotide polymorphism, SSTI = infections of the skin and subcutaneous tissue.

*P* <.05 was considered significant.

## 6. Discussion

### 6.1. Principal findings

This study represents the first genetic investigation that combines various MR methods to compare the predictive values of 3 adiposity indices (BMI, WC, and HC) in relation to infection risk. The univariate and multivariate MR analyses conducted herein consistently demonstrated that only WC, not BMI or HC, exhibited a stable causal association with various infectious diseases, such as sepsis, cholecystitis, and SSTI.

### 6.2. Comparison with other studies

Previous studies have used BMI to assess the association between body weight and infectious diseases, owing to its ease of calculation. However, the results were inconsistent.^[15,37]^ In a large cohort study,^[37]^ participants were divided into different subgroups according to BMI; all of them were associated with sepsis, with no notable differences. Conversely, another matched cohort study^[15]^ argued that only a BMI > 40 or <20 kg/m^2^ was associated with sepsis. This paradox may be attributed to inherent defects in BMI, as it fails to account for fat distribution; its accuracy in distinguishing normal weight from obesity may be influenced by the body fat-to-muscle ratio. Additionally, various infectious diseases, such as pneumonia and wound infection, have been associated with sarcopenic obesity phenotypes, independent of BMI.^[38]^ Moreover, traditional observational studies have often struggled to address the influence of reverse causality and confounding factors.^[39]^

A population-based cohort study of >30,000 participants showed that sepsis risk was primarily associated with increased WC rather than BMI, indicating that WC may be a better predictor of sepsis risk.^[40]^ Similarly, in a recent RCT involving 2954 patients, WC was a better predictor than BMI and waist-to-hip ratio in predicting sepsis incidence and mortality, surgical site infection, and pneumonia.^[13]^ However, the results of this RCT were limited to patients who underwent major abdominal surgery, thereby restricting its generalizability. To address this limitation, the present study expands on previous research by employing significantly larger sample sizes (51,852–486,484) and encompassing a broader study population that included various infectious diseases. Using different MR methods, the present study consistently supports the notion that WC outperforms BMI and HC in predicting cholecystitis, ALRI, and SSTI risks as well as sepsis-related morbidity and mortality.

The results of this study are consistent with those of previous MR analyses, which demonstrated a positive linear association between BMI and infectious disease risk.^[41–43]^ However, these analyses focused solely on the causal relationship between BMI and infectious diseases and, neglected the role of other adiposity indices. Although BMI provides an overall measure of obesity, it fails to account for body fat distribution and distinguish between muscle and fat. Additionally, being underweight (BMI < 18.5 kg/m^2^) is often associated with weakness, malnutrition, and chronic diseases, all of which can increase the infection risk.^[44,45]^ Thus, it is crucial to critically evaluate the plausibility of the linear relationship between BMI and different infectious disease risk factors. Jensen et al discovered that the relationship between BMI and infection followed a U-shaped curve, whereas MR analysis suggested a linear relationship.^[43]^ This paradox may be attributed to confounding factors or inherent limitations of BMI.

To address BMI limitations, the present MR study analyzed each adiposity index (BMI, WC, and HC) using the same infectious disease set. Univariate TSMR and MVMR analyses were performed. Ultimately, the findings from the univariate TSMR and MVMR analyses consistently demonstrated that WC, unlike BMI and HC, exhibited independent causal relationships with SS, SM, cholecystitis, and SSTI risk.

In the MVMR analysis adjusted for BMI and HC, the association between WC and SM (OR = 3.23; 95% CI: 1.52–6.86; *P* = .002) was much stronger than that in the univariable analysis (OR = 1.98; 95% CI: 1.50–2.61; *P* = 3.86E−24). This discrepancy may have been influenced by residual pleiotropy, weak instrument bias, or collider bias. HC exerts protective effects against various diseases.^[46,47]^ Meanwhile, BMI’s inability to differentiate between muscle and fat composition may lead to confounding in risk estimation.^[6]^ Adjusting for these factors in the MVMR analysis likely uncovered WC’s independent effect of WC by removing protective signals from HC or BMI-related biases. Furthermore, the MVMR result (WC to SM) aligns with the findings from a recent large cohort study involving 77,810 adults with sepsis.^[48]^ Importantly, the sensitivity analyses (Table 2) revealed no significant pleiotropy or heterogeneity, further supporting the robustness of our results.

### 6.3. Possible mechanisms

Increased WC may elevate the risk of infectious disease due to the following factors. First, visceral adipose tissue, a hallmark of abdominal obesity, is metabolically active and promotes the production of pro-inflammatory cytokines such as interleukin-6 and tumor necrosis factor-alpha.^[49]^ These cytokines contribute to a state of chronic low-grade inflammation, which impairs normal immune responses and predisposes individuals to infections.^[50]^ For instance, elevated interleukin-6 levels have been associated with hyperinflammatory states that drive endothelial glycocalyx damage, thereby increasing susceptibility to infection and mortality in sepsis and COVID‑19.^[51]^ Second, abdominal obesity plays a role in immunomodulation by increasing adipose tissue macrophages, promoting the release of reactive oxygen species, and activating T and B cells as well as Toll-like receptor-4.^[52]^ Chronic inflammation is closely associated with metabolic syndrome, insulin resistance, and cardiovascular disease, all of which contribute to a higher risk of infection.^[53,54]^ Lastly, studies have shown that individuals with abdominal obesity are at a higher risk for conditions such as COVID‑19, sepsis, surgical site infections, and postoperative pneumonia. This may be due to the combined effects of obesity impairing immune function, provoking chronic inflammation, promoting the development of heart failure and diabetes, and impairing lung ventilation.^[48,55,56]^

### 6.4. Strengths and limitations

The present MR study has several notable strengths. First, causal estimates derived from the largest or most recent GWAS datasets of WC, HC, and BMI were utilized to ensure comprehensive coverage of adiposity indices. Second, a rigorous screening process was implemented for IVs, adhering to 3 key assumptions. Third, the stability of the study findings was ensured by consistent results obtained using different MR methods and sensitivity analyses. No significant pleiotropy was detected during the analysis, which further enhanced the robustness and reliability of the results.

This study has some limitations. First, to ensure no sample overlap between exposures and outcomes, the most recent GWAS for BMI was selected, instead of the GWAS with the largest sample size. This decision might have resulted in lower statistical power in the univariate TSMR analysis of the relationship between BMI and infectious diseases. Second, the generalizability of our study results is limited, as the analysis was conducted exclusively within European populations, which may not reflect the genetic and environmental diversity of other ethnic groups. Additionally, the findings may not apply to different exposure periods or varying levels of WC, warranting further research in diverse populations and contexts. Third, the limited number of SNPs available as IVs in the reverse MR analysis prevented us from conducting sensitivity analysis. Therefore, future investigations with larger numbers of IVs are required to explore and confirm the existence of a reverse causal relationship between infectious diseases and WC. Fourth, although strict methods for instrumental variable selection were applied to satisfy the 3 fundamental assumptions of the MR analysis, the chosen instruments may still be susceptible to the winner’s curse phenomenon. While the reverse MR analysis was limited by an insufficient number of eligible SNPs for pleiotropy testing, other MR analyses did not detect statistically significant horizontal pleiotropy. However, undetected horizontal pleiotropy could theoretically persist given that infectious diseases are multifactorial disorders influenced by host survival status, environmental exposures, and pathogen characteristics. Furthermore, selection bias remains an unavoidable challenge in MR analyses using large-scale biobanks.

## 7. Conclusions

WC may outperform BMI and HC as predictors of the risk of infection. We suggest using WC as a predictor and promoting WC control to decrease the risk of infectious diseases. However, because our study relied on adiposity indices from GWAS datasets, which are primarily based on cross-sectional data, we were unable to assess the dynamic relationship between adiposity indices and infection risk or perform subgroup analyses. Future long-term prospective studies are needed to address these limitations and explore the associations with diverse infectious diseases in different populations.

## Acknowledgments

Summary-level data for the exposures and outcomes were extracted from the WFGC database, Medical Research Council Integrative Epidemiology Unit, UK Biobank, and FinnGen Biobank. We thank all the participants for their data sharing.

## Author contributions

**Conceptualization:** Xiaohong Chen, Chao Chen, Tao Xiang.

**Formal analysis:** Xiaohong Chen, Jia Yin, Chao Chen.

**Funding acquisition:** Xiaohong Chen.

**Investigation:** Xiaohong Chen, Jia Yin, Chao Chen.

**Methodology:** Xiaohong Chen, Jia Yin.

**Supervision:** Chao Chen, Tao Xiang.

**Writing – original draft:** Xiaohong Chen.

**Writing – review & editing:** Chao Chen, Tao Xiang.

## Supplementary Material


