# Supplementary material for: Causal association between 3 adiposity indices and 5 infectious diseases: A Mendelian randomization study
Source: Medicine (Baltimore). 2025 Nov 7;104(45):e45775. doi: 10.1097/MD.0000000000045775 (PMC12599714; doi:10.1097/MD.0000000000045775)

Table S1. Genome-wide association study data information of exposures and outcomes.

Exposure	GWAS ID	Unit	Year	Consortium	Sample size (cases)	Population
**BMI**	ieu-b-4815	kg/m^2	2022	WFGC	51,852	European
**WC**	ukb-b-9405	SD	2018	MRC-IEU	462,166	European
**HC**	ukb-b-15590	SD	2018	MRC-IEU	462,117	European
**Outcomes**	**GWAS ID**	**Unit**	**Year**	**Consortium**	**Sample size (cases)**	**Population**
**SS**	ieu-b-4980	logOR	2021	UK Biobank	486,484 (11,643)	European
**SM**	ieu-b-5086	logOR	2021	UK Biobank	486,484 (1,896)	European
**ALRI**	finn-b-J10_LOWERINF	NA	2021	FinnGen biobank	218,792 (10,103)	European
**II**	finn-b-AB1_VIRAL_OTHER_INTEST_INFECTIONS	NA	2021	FinnGen biobank	201,463 (4,165)	European
**SSTI**	finn-b-L12_INFECT_SKIN	NA	2021	FinnGen biobank	218,792 (10,343)	European
**cholecystitis**	finn-b-CHOLELITH_BROAD	NA	2021	FinnGen biobank	215,027 (19,883)	European

Abbreviations: BMI, body mass index; WC, waist circumference; HC, hip circumference; SS, sepsis susceptibility; SM, sepsis mortality; ALRI, acute lower respiratory infections; II, intestinal infections; SSTI, infections of the skin and subcutaneous tissue; WFGC, within family genome-wide association study consortium; MRI-IEU, medical research council integrative epidemiology unit; UK, United Kingdom; GWAS, Genome-wide association study.

Table S2. Deleted single-nucleotide polymorphisms in univariate two-sample Mendelian randomization analyses.

Exposure	Outcome	The deleted SNP (NO. of deleted SNP)
BMI	SS	Being palindromic with intermediate allele frequencies (**4**): rs10203386, rs13011109, rs4776970, rs8083289;
		F-statistics <10 (**1**): rs77165542;
		Associated with potential confounders# (**0**): NA;
		Associated with the outcome ($p< 0.05$) (**0**): NA;
		Outliers (MR-PRESSO) (**1**): rs1320336
	SM	Being palindromic with intermediate allele frequencies (**4**): rs10203386, rs13011109, rs4776970, rs8083289;
		F-statistics <10 (**1**): rs77165542;
		Associated with potential confounders# (**0**): NA;
		Associated with the outcome ($p< 0.05$) (**0**): NA;
		Outliers (MR-PRESSO) (**0**): NA
	cholecystitis	Being palindromic with intermediate allele frequencies (**4**): rs10203386, rs13011109, rs4776970, rs8083289;
		F-statistics <10 (**1**): rs77165542;
		Associated with potential confounders# (**0**): NA;
		Associated with the outcome ($p< 0.05$) (**0**): NA;
		Outliers (MR-PRESSO) (**0**): NA
	II	Being palindromic with intermediate allele frequencies (**4**): rs10203386, rs13011109, rs4776970, rs8083289;
		F-statistics <10 (**1**): rs77165542;
		Associated with potential confounders# (**0**): NA;
		Associated with the outcome ($p< 0.05$) (**0**): NA;
		Outliers (MR-PRESSO) (**0**): NA
	SSTI	Being palindromic with intermediate allele frequencies (**4**): rs10203386, rs13011109, rs4776970, rs8083289;
		F-statistics <10 (**1**): rs77165542;
		Associated with potential confounders# (**0**): NA;
		Associated with the outcome ($p< 0.05$) (**0**): NA;

Exposure	Outcome	The deleted SNP (NO. of deleted SNP)
HC	ALRI	Outliers (MR-PRESSO) (**0**): NA<br>Being palindromic with intermediate allele frequencies (**4**): rs10203386, rs13011109, rs4776970, rs8083289;<br>F-statistics <10 (**1**): rs77165542;<br>Associated with potential confounders# (**0**): NA;<br>Associated with the outcome (p< 0.05) (**0**): NA;
	SS	Outliers (MR-PRESSO) (**0**): NA;<br>Being palindromic with intermediate allele frequencies (**12**): rs10069930, rs10954284, rs11778934, rs11882796, rs12631813, rs133015, rs2371911, rs2593169, rs56097510, rs6597975, rs765874, rs7695177;<br>Incompatible alleles (**1**): rs1294438;<br>F-statistics <10 (**53**): rs10158176, rs10188429, rs10499343, rs10788797, rs10947137, rs10984732, rs11056870, rs113866544, rs113866544, rs116072427, rs117176448, rs117342986, rs12096864, rs12140153, rs12245654, rs12881629, rs13081528, rs13204087, rs139218003, rs141622900, rs143786003, rs148636479, rs17446299, rs17604662, rs1801123, rs1801282, rs186017271, rs190543502, rs1982441, rs34045894, rs34505073, rs35589824, rs3730071, rs41279738, rs4339309, rs4350264, rs551935, rs614520, rs6465828, rs667515, rs675162, rs72656010, rs72801843, rs73175572, rs7365, rs7523668, rs754635, rs7582359, rs7632381, rs7805441, rs78058190, rs7996639, rs9951619;<br>Associated with potential confounders# (**0**): NA;<br>Associated with the outcome (p< 0.05) (**0**): NA;
	SM	Outliers (MR-PRESSO) (**1**): rs4589131;<br>Being palindromic with intermediate allele frequencies (**12**): rs10069930, rs10954284, rs11778934, rs11882796, rs12631813, rs133015, rs2371911, rs2593169, rs56097510, rs6597975, rs765874, rs7695177;<br>Incompatible alleles (**1**): rs1294438;<br>F-statistics <10 (**52**): rs10158176, rs10188429, rs10499343, rs10788797, rs10947137, rs10984732, rs11056870, rs113866544, rs116072427, rs117176448, rs117342986, rs12096864, rs12140153, rs12245654, rs12881629, rs13081528, rs13204087, rs139218003, rs141622900, rs143786003, rs148636479, rs17446299, rs17604662,

Exposure	Outcome	The deleted SNP (NO. of deleted SNP)
		rs1801123, rs1801282, rs186017271, rs190543502, rs1982441, rs34045894, rs34505073, rs35589824, rs3730071, rs41279738, rs4339309, rs4350264, rs55707359, rs61898343, rs6470771, rs6712653, rs67560975, rs72681698, rs72877553, rs73197346, rs73650963, rs754635, rs75543804, rs76082446, rs76577501, rs78058190, rs7824785, rs8007429, rs9955276;<br>Associated with potential confounders# (**0**): NA;<br>Associated with the outcome ($p < 0.05$) (**0**): NA;<br>Outliers (MR-PRESSO) (**0**):NA
	cholecystitis	Being palindromic with intermediate allele frequencies (**15**): rs10954284, rs11778934, rs11882796, rs12631813, rs13264909, rs133015, rs2032251, rs2238435, rs2253310, rs2371911, rs2593169, rs56097510, rs6597975, rs765874, rs7695177;<br>Incompatible alleles (**1**): rs7793674;<br>F-statistics <10 (**54**): rs10158176, rs10188429, rs10499343, rs10788797, rs10947137, rs11056870, rs113866544, rs116072427, rs117176448, rs117342986, rs12096864, rs12140153, rs12245654, rs12881629, rs13081528, rs13204087, rs139218003, rs143786003, rs148636479, rs17446299, rs17604662, rs1801123, rs1801282, rs186017271, rs190543502, rs1982441, rs34045894, rs34505073, rs35589824, rs3730071, rs41271299, rs4339309, rs4350264, rs4897174, rs55707359, rs61898343, rs62246311, rs6470771, rs6712653, rs67560975, rs72681698, rs72877553, rs73197346, rs73650963, rs754635, rs75543804, rs76082446, rs76577501, rs76895963, rs78058190, rs7824785, rs8007429, rs9611160, rs9955276;<br>Associated with potential confounders# (**0**): NA;<br>Associated with the outcome ($p < 0.05$) (**2**): rs1260326, rs41279738;<br>Outliers (MR-PRESSO) (**7**): rs1047891, rs112875651, rs141622900, rs28711392, rs4467770, rs72959041, rs7523668;
	II	Being palindromic with intermediate allele frequencies (**15**): rs10954284, rs11778934, rs11882796, rs12631813, rs13264909, rs133015, rs2032251, rs2238435, rs2253310, rs2371911, rs2593169, rs56097510, rs6597975, rs765874, rs7695177;

Exposure	Outcome	The deleted SNP (NO. of deleted SNP)
		Incompatible alleles (**1**): rs7793674;
		F-statistics <10 (**56**): rs10158176, rs10188429, rs10499343, rs10788797, rs10947137, rs11056870, rs113866544, rs116072427, rs117176448, rs117342986, rs12096864, rs12140153, rs12245654, rs12881629, rs13081528, rs13204087, rs139218003, rs141622900, rs143786003, rs148636479, rs17446299, rs17604662, rs1801123, rs1801282, rs186017271, rs190543502, rs1982441, rs34045894, rs34505073, rs35589824, rs3730071, rs41271299, rs41279738, rs4339309, rs4350264, rs4897174, rs55707359, rs61898343, rs62246311, rs6470771, rs6712653, rs67560975, rs72681698, rs72877553, rs73197346, rs73650963, rs754635, rs75543804, rs76082446, rs76577501, rs76895963, rs78058190, rs7824785, rs8007429, rs9611160, rs9955276;
		Associated with potential confounders[#] (**0**): NA;
		Associated with the outcome (p< 0.05) (**0**): NA;
		Outliers (MR-PRESSO) (**0**): NA;
	SSTI	Being palindromic with intermediate allele frequencies (**15**): rs10954284, rs11778934, rs11882796, rs12631813, rs13264909, rs133015, rs2032251, rs2238435, rs2253310, rs2371911, rs2593169, rs56097510, rs6597975, rs765874, rs7695177;
		Incompatible alleles (**1**): rs7793674;
		F-statistics <10 (**56**): rs10158176, rs10188429, rs10499343, rs10788797, rs10947137, rs11056870, rs113866544, rs116072427, rs117176448, rs117342986, rs12096864, rs12140153, rs12245654, rs12881629, rs13081528, rs13204087, rs139218003, rs141622900, rs143786003, rs148636479, rs17446299, rs17604662, rs1801123, rs1801282, rs186017271, rs190543502, rs1982441, rs34045894, rs34505073, rs35589824, rs3730071, rs41271299, rs41279738, rs4339309, rs4350264, rs4897174, rs55707359, rs61898343, rs62246311, rs6470771, rs6712653, rs67560975, rs72681698, rs72877553, rs73197346, rs73650963, rs754635, rs75543804, rs76082446, rs76577501, rs76895963, rs78058190, rs7824785, rs8007429, rs9611160, rs9955276;
		Associated with potential confounders[#] (**0**): NA;
		Associated with the outcome (**p<0.05**) (**35**): rs10153248, rs1056441, rs10938397, rs11012732, rs11150745, rs11762444, rs12831185, rs12880641, rs13034765, rs13264909, rs1727901, rs2133561, rs2151248, rs253444,

Exposure	Outcome	The deleted SNP (NO. of deleted SNP)
		rs2737263, rs3110942, rs34517439, rs34765854, rs34811474, rs35792595, rs35910339, rs36140, rs3746759, rs3845344, rs4017425, rs4820346, rs4985407, rs57235969, rs57989773, rs6973700, rs7038943, rs7094073, rs743572, rs750090, rs9951619;
		Outliers (MR-PRESSO) (**1**): rs11513729;
	ALRI	Being palindromic with intermediate allele frequencies (**15**): rs10954284, rs11778934, rs11882796, rs12631813, rs13264909, rs133015, rs2032251, rs2238435, rs2253310, rs2371911, rs2593169, rs56097510, rs6597975, rs765874, rs7695177;
		Incompatible alleles (**1**): rs7793674;
		F-statistics <10 (**56**): rs10158176, rs10188429, rs10499343, rs10788797, rs10947137, rs11056870, rs113866544, rs116072427, rs117176448, rs117342986, rs12096864, rs12140153, rs12245654, rs12881629, rs13081528, rs13204087, rs139218003, rs141622900, rs143786003, rs148636479, rs17446299, rs17604662, rs1801123, rs1801282, rs186017271, rs190543502, rs1982441, rs34045894, rs34505073, rs35589824, rs3730071, rs41271299, rs41279738, rs4339309, rs4350264, rs4897174, rs55707359, rs61898343, rs62246311, rs6470771, rs6712653, rs67560975, rs72681698, rs72877553, rs73197346, rs73650963, rs754635, rs75543804, rs76082446, rs76577501, rs76895963, rs78058190, rs7824785, rs8007429, rs9611160, rs9955276;
		Associated with potential confounders[#] (**0**): NA;
		Associated with the outcome ($p< 0.05$) (**0**): NA;
		Outliers (MR-PRESSO) (**1**): rs7365;
WC	SS	Being palindromic with intermediate allele frequencies (**12**): rs10406327, rs10887578, rs11666480, rs11778934, rs1405261, rs1441098, rs1441098, rs1454687, rs165656, rs347551, rs3949781, rs4856720;
		Incompatible alleles (**2**): rs1441098, rs156902;
		F-statistics <10 (**41**): rs10505836, rs10824211, rs11160600, rs113132247, rs113866544, rs114964326, rs115056380, rs11603984, rs12042959, rs12107172, rs12245654, rs12273545, rs1229984, rs12549000, rs145350287, rs1619442, rs1799923, rs1942826, rs3087523, rs35216639, rs36007635, rs41279738, rs4148155, rs4344019, rs4482463, rs4722398, rs4844809, rs55794894, rs6030803, rs62072003, rs67632512, rs71495038, rs72618637, rs72959041,

Exposure	Outcome	The deleted SNP (NO. of deleted SNP)
		rs75035127, rs76040172, rs8024137, rs9370243, rs9478496, rs9654453, rs9926784;
		Associated with potential confounders# (**0**): NA;
		Associated with the outcome (p< 0.05) (**0**): NA;
		Outliers (MR-PRESSO) (**0**): NA;
	SM	Being palindromic with intermediate allele frequencies (**12**): rs10406327, rs10887578, rs11666480, rs11778934, rs1405261, rs1441098, rs1441098, rs1454687, rs165656, rs347551, rs3949781, rs4856720;
		Incompatible alleles (**2**): rs1441098, rs156902;
		F-statistics <10 (**41**): rs10505836, rs10824211, rs11160600, rs113132247, rs113866544, rs114964326, rs115056380, rs11603984, rs12042959, rs12107172, rs12245654, rs12273545, rs1229984, rs12549000, rs145350287, rs1619442, rs1799923, rs1942826, rs3087523, rs35216639, rs36007635, rs41279738, rs4148155, rs4344019, rs4482463, rs4722398, rs4844809, rs55794894, rs6030803, rs62072003, rs67632512, rs71495038, rs72618637, rs72959041, rs75035127, rs76040172, rs8024137, rs9370243, rs9478496, rs9654453, rs9926784;
		Associated with potential confounders# (**0**): NA;
		Associated with the outcome (p< 0.05) (**0**): NA;
		Outliers (MR-PRESSO) (**0**): NA;
	cholecystitis	Being palindromic with intermediate allele frequencies (**16**): rs10406327, rs10887578, rs11666480, rs11778934, rs13047416, rs13264909, rs1405261, rs1441098, rs1454687, rs165656, rs2253310, rs2618039, rs347551, rs3949781, rs4419475, rs4856720;
		Incompatible alleles (**2**): rs12926311, rs156902;
		F-statistics <10 (**44**): rs10505836, rs10824211, rs10957087, rs11160600, rs113132247, rs113866544, rs114964326, rs115056380, rs11603984, rs12042959, rs12107172, rs12245654, rs12273545, rs1229984, rs12549000, rs145350287, rs1619442, rs1799923, rs1942826, rs3087523, rs35216639, rs36007635, rs4148155, rs4344019, rs4482463, rs4722398, rs4844809, rs55794894, rs6030803, rs62072003, rs62246311, rs67632512, rs71495038, rs72618637, rs72959041, rs74395133, rs75035127, rs76040172, rs76895963, rs8024137, rs9370243, rs9478496, rs9654453, rs9926784;

Exposure	Outcome	The deleted SNP (NO. of deleted SNP)
		Associated with potential confounders# (**0**): NA;
		Associated with the outcome (p<0.05) (**40**): rs11824092, rs41279738, rs6001877, rs1013402, rs10184230, rs10236214, rs10423928, rs10938398, rs10947793, rs11704728, rs13047416, rs1327259, rs13322435, rs13333747, rs13420048, rs13427822, rs1411432, rs1861410, rs1902066, rs2183947, rs2470946, rs2618039, rs2744938, rs34517439, rs36140, rs3826408, rs429358, rs56094641, rs61969511, rs6536575, rs6693294, rs7034554, rs7845090, rs7925100, rs7952436, rs8013377, rs945211, rs9568867, rs9584870, rs9843653;
		Outliers (MR-PRESSO) (**2**): rs2376885, rs77165542;
	II	Being palindromic with intermediate allele frequencies (**16**): rs10406327, rs10887578, rs11666480, rs11778934, rs13047416, rs13264909, rs1405261, rs1441098, rs1454687, rs165656, rs2253310, rs2618039, rs347551, rs3949781, rs4419475, rs4856720;
		Incompatible alleles (**2**): rs12926311, rs156902;
		F-statistics <10 (**45**): rs10505836, rs10824211, rs10957087, rs11160600, rs113132247, rs113866544, rs114964326, rs115056380, rs11603984, rs12042959, rs12107172, rs12245654, rs12273545, rs1229984, rs12549000, rs145350287, rs1619442, rs1799923, rs1942826, rs3087523, rs35216639, rs36007635, rs41279738, rs4148155, rs4344019, rs4482463, rs4722398, rs4844809, rs55794894, rs6030803, rs62072003, rs62246311, rs67632512, rs71495038, rs72618637, rs72959041, rs74395133, rs75035127, rs76040172, rs76895963, rs8024137, rs9370243, rs9478496, rs9654453, rs9926784;
		Associated with potential confounders# (**0**): NA;
		Associated with the outcome (p< 0.05) (**0**): NA;
		Outliers (MR-PRESSO) (**0**): NA;
	SSTI	Being palindromic with intermediate allele frequencies (**16**): rs10406327, rs10887578, rs11666480, rs11778934, rs13047416, rs13264909, rs1405261, rs1441098, rs1454687, rs165656, rs2253310, rs2618039, rs347551, rs3949781, rs4419475, rs4856720;
		Incompatible alleles (**2**): rs12926311, rs156902;
		F-statistics <10 (**45**): rs10505836, rs10824211, rs10957087, rs11160600, rs113132247, rs113866544, rs114964326,

Exposure	Outcome	The deleted SNP (NO. of deleted SNP)
	ALRI	rs115056380, rs11603984, rs12042959, rs12107172, rs12245654, rs12273545, rs1229984, rs12549000, rs145350287, rs1619442, rs1799923, rs1942826, rs3087523, rs35216639, rs36007635, rs41279738, rs4148155, rs4344019, rs4482463, rs4722398, rs4844809, rs55794894, rs6030803, rs62072003, rs62246311, rs67632512, rs71495038, rs72618637, rs72959041, rs74395133, rs75035127, rs76040172, rs76895963, rs8024137, rs9370243, rs9478496, rs9654453, rs9926784;<br>Associated with potential confounders# (**0**): NA;<br>Associated with the outcome (p< 0.05) (**0**): NA;<br>Outliers (MR-PRESSO) (**0**): NA;<br>Being palindromic with intermediate allele frequencies (**16**): rs10406327, rs10887578, rs11666480, rs11778934, rs13047416, rs13264909, rs1405261, rs1441098, rs1454687, rs165656, rs2253310, rs2618039, rs347551, rs3949781, rs4419475, rs4856720;<br>Incompatible alleles (**2**): rs12926311, rs156902;<br>F-statistics <10 (**45**): rs10505836, rs10824211, rs10957087, rs11160600, rs113132247, rs113866544, rs114964326, rs115056380, rs11603984, rs12042959, rs12107172, rs12245654, rs12273545, rs1229984, rs12549000, rs145350287, rs1619442, rs1799923, rs1942826, rs3087523, rs35216639, rs36007635, rs41279738, rs4148155, rs4344019, rs4482463, rs4722398, rs4844809, rs55794894, rs6030803, rs62072003, rs62246311, rs67632512, rs71495038, rs72618637, rs72959041, rs74395133, rs75035127, rs76040172, rs76895963, rs8024137, rs9370243, rs9478496, rs9654453, rs9926784;<br>Associated with potential confounders# (**0**): NA;<br>Associated with the outcome (p< 0.05) (**0**): NA;<br>Outliers (MR-PRESSO) (**0**): NA;
SS	WC	Incompatible alleles (**1**): rs7103228;<br>F-statistics <10 (**13**): rs11068069, rs112431283, rs11980516, rs12544445, rs139409755, rs147734876, rs147793338, rs150753765, rs2226602, rs7103228, rs7103228, rs72820148, rs80054869;<br>Associated with potential confounders*(**1**): rs4841254;

Exposure	Outcome	The deleted SNP (NO. of deleted SNP)
		Associated with the outcome (p< 0.05) (**0**): NA;
SM	WC	F-statistics <10 (**13**): rs114724320, rs145413915, rs148818459, rs17090050, rs177999, rs190351470, rs470236, rs4787745, rs62340396, rs7155416, rs77613868, rs824453, rs9955473;
		Associated with potential confounders# (**0**): NA;
		Associated with the outcome (p< 0.05) (**0**): NA;
cholecystitis	WC	F-statistics <10 (**6**): rs114041253, rs114350689, rs12775076, rs149519950, rs191092790, rs28929474;
		Associated with potential confounders*(**1**): rs1260326;
		Associated with the outcome (p< 0.05) (**0**): NA;
SSTI	WC	Being palindromic with intermediate allele frequencies (**1**): rs3847708;
		F-statistics <10 (**9**): rs11507681, rs115500600, rs149206131, rs150168502, rs2441946, rs2760985, rs4939270, rs72795151, rs78769046;
		Associated with potential confounders# (**0**): NA;
		Associated with the outcome (p< 0.05) (**0**): NA;
ALRI	WC	F-statistics <10 (**11**): rs10736688, rs111322934, rs117551230, rs11876267, rs12502278, rs138573236, rs2132031, rs72726131, rs72820855, rs7798902, rs78063827;
		Associated with potential confounders# (**0**): NA;
		Associated with the outcome (p< 0.05) (**0**): NA;

# Atrial fibrillation, atrial flutter, and hyperglycemia were considered potential confounders associated with infectious diseases.

* Body mass index and hip circumference were considered potential confounders associated with waist circumference.

Abbreviations: BMI, body mass index; WC, waist circumference; HC, hip circumference; SNP, single nucleotide polymorphism; SS, sepsis susceptibility; SM, sepsis mortality; II, intestinal infections; SSTI, infections of the skin and subcutaneous tissue; ALRI, acute lower respiratory infections; MR-PRESSO, Mendelian randomization pleiotropy residual sum and outlier.

Table S3. Detailed information about single-nucleotide polymorphisms of body mass index on sepsis susceptibility.

SNP	effect_allele BMI	other_alleleBMI	eaf.SS	beta.BMI	se.BMI	pval.BMI	beta.SS	se.SS	pval.SS	F-statistics
rs10203386	A	T	0.451931	0.1623	0.0274	3.35E-09	0.001508	0.013737	0.912583	17.39
rs13011109	C	G	0.388533	-0.1757	0.0287	8.69E-10	0.026658	0.014028	0.057385	17.81
rs13130484	T	C	0.43364	0.1666	0.028	2.61E-09	0.019038	0.013819	0.168303	17.39
rs1342396	T	C	0.670197	0.1678	0.0292	8.72E-09	0.003855	0.01458	0.791456	14.60
rs4776970	T	A	0.35779	-0.1729	0.0287	1.71E-09	0.003616	0.014249	0.799664	16.68
rs543874	G	A	0.205211	0.2652	0.0333	1.67E-15	0.024784	0.016909	0.142718	20.70
rs62033400	G	A	0.394817	0.3768	0.0286	1.26E-39	0.020982	0.013982	0.133456	83.08
rs8083289	G	C	0.264097	0.3436	0.0317	2.44E-27	-0.01165	0.015492	0.452018	45.71
rs9378638	T	C	0.252715	0.1762	0.0319	3.40E-08	0.013306	0.015923	0.403343	11.53

Abbreviations: SNP, single nucleotide polymorphism; se, standard error; BMI, body mass index; SS, sepsis susceptibility; pval, p-value.

Table S4. Detailed information about single-nucleotide polymorphisms of body mass index on sepsis mortality.

SNP	effect_allele BMI	other_alleleBMI	eaf.SM	beta.BMI	se.BMI	pval.BMI	beta.SM	se.SM	pval.SM	F-statistics
rs10203386	A	T	0.451931	0.1623	0.0274	3.35E-09	-0.05881	0.033556	0.079693	17.39
rs13011109	C	G	0.388533	-0.1757	0.0287	8.69E-10	0.027659	0.034253	0.419384	17.81
rs13130484	T	C	0.43364	0.1666	0.028	2.61E-09	0.072342	0.033699	0.031817	17.39
rs1320336	A	G	0.172079	-0.3025	0.0367	1.56E-16	-0.08966	0.044118	0.042136	19.36
rs1342396	T	C	0.670197	0.1678	0.0292	8.72E-09	0.043579	0.03559	0.220767	14.60
rs4776970	T	A	0.35779	-0.1729	0.0287	1.71E-09	-0.08334	0.034763	0.016509	16.68
rs543874	G	A	0.205211	0.2652	0.0333	1.67E-15	0.003688	0.041292	0.92883	20.70
rs62033400	G	A	0.394817	0.3768	0.0286	1.26E-39	0.027764	0.034065	0.415046	83.08
rs8083289	G	C	0.264097	0.3436	0.0317	2.44E-27	-0.03483	0.037774	0.356512	45.71
rs9378638	T	C	0.252715	0.1762	0.0319	3.40E-08	0.060349	0.038832	0.120158	11.53

Abbreviations: SNP, single nucleotide polymorphism; se, standard error; BMI, body mass index; SM, sepsis mortality; pval, p-value.

Table S5. Detailed information about single-nucleotide polymorphisms of body mass index on cholecystitis.

SNP	effect_allele BMI	other_alleleBMI	eaf. cholecystitis	beta.BMI	se.BMI	pval.BMI	beta. cholecystitis	se. cholecystitis	pval. cholecystitis	F-statistics
rs10203386	A	T	0.3949	0.1623	0.0274	3.35E-09	0.0059	0.012	0.621999	16.77
rs13011109	C	G	0.4204	-0.1757	0.0287	8.69E-10	-0.0223	0.012	0.0624094	18.27
rs13130484	T	C	0.4727	0.1666	0.028	2.61E-09	0.0266	0.0118	0.0239001	17.65
rs1320336	A	G	0.1626	-0.3025	0.0367	1.56E-16	-0.0201	0.0159	0.2048	18.51
rs1342396	T	C	0.6913	0.1678	0.0292	8.72E-09	0.0013	0.0127	0.92	14.10
rs4776970	T	A	0.3453	-0.1729	0.0287	1.71E-09	-0.017	0.0123	0.1674	16.41
rs543874	G	A	0.1783	0.2652	0.0333	1.67E-15	0.0202	0.0154	0.189	18.59
rs62033400	G	A	0.4027	0.3768	0.0286	1.26E-39	0.0407	0.012	6.62E-04	83.63
rs8083289	G	C	0.1947	0.3436	0.0317	2.44E-27	0.0259	0.0148	0.0791498	36.87
rs9378638	T	C	0.3375	0.1762	0.0319	3.40E-08	0.0028	0.0125	0.8226	13.65

Abbreviations: SNP, single nucleotide polymorphism; se, standard error; BMI, body mass index; pval, p-value.

Table S6. Detailed information about single-nucleotide polymorphisms of body mass index on intestinal infections.

SNP	effect_allele BMI	other_alleleBMI	eaf.II	beta.BMI	se.BMI	pval.BMI	beta. II	se. II	pval. II	F-statistics
rs10203386	A	T	0.3946	0.1623	0.0274	3.35E-09	0.0086	0.0231	0.710799	16.77
rs13011109	C	G	0.4204	-0.1757	0.0287	8.69E-10	0.0051	0.023	0.8258	18.27
rs13130484	T	C	0.4732	0.1666	0.028	2.61E-09	0.0094	0.0226	0.6773	17.66
rs1320336	A	G	0.1625	-0.3025	0.0367	1.56E-16	0.0231	0.0309	0.4546	18.50
rs1342396	T	C	0.6912	0.1678	0.0292	8.72E-09	-0.0381	0.0244	0.1188	14.10
rs4776970	T	A	0.3455	-0.1729	0.0287	1.71E-09	-0.0286	0.0237	0.2282	16.42
rs543874	G	A	0.1782	0.2652	0.0333	1.67E-15	-0.0229	0.0295	0.4369	18.58
rs62033400	G	A	0.4027	0.3768	0.0286	1.26E-39	-0.0181	0.0231	0.4333	83.63
rs8083289	G	C	0.1949	0.3436	0.0317	2.44E-27	0.0078	0.0285	0.785001	36.90
rs9378638	T	C	0.3375	0.1762	0.0319	3.40E-08	-0.017	0.024	0.4795	13.65

Abbreviations: SNP, single nucleotide polymorphism; se, standard error; BMI, body mass index; II, intestinal infections; pval, p-value.

Table S7. Detailed information about single-nucleotide polymorphisms of body mass index on infections of the skin and subcutaneous tissue.

SNP	effect_allele BMI	other_alleleBMI	eaf.SSTI	beta.BMI	se.BMI	pval.BMI	beta. SSTI	se. SSTI	pval. SSTI	F-statistics
rs10203386	A	T	0.3949	0.1623	0.0274	3.35E-09	0.0184	0.015	0.2202	16.77
rs13011109	C	G	0.4205	-0.1757	0.0287	8.69E-10	0.0225	0.0149	0.1319	18.27
rs13130484	T	C	0.4728	0.1666	0.028	2.61E-09	0.0341	0.0147	0.0207	17.65
rs1320336	A	G	0.1626	-0.3025	0.0367	1.56E-16	-0.0361	0.02	0.0710902	18.51
rs1342396	T	C	0.6913	0.1678	0.0292	8.72E-09	0.034	0.0159	0.0321699	14.10
rs4776970	T	A	0.3452	-0.1729	0.0287	1.71E-09	0.0067	0.0154	0.6637	16.41
rs543874	G	A	0.1782	0.2652	0.0333	1.67E-15	0.0093	0.0192	0.6292	18.58
rs62033400	G	A	0.4025	0.3768	0.0286	1.26E-39	0.0169	0.015	0.2596	83.62
rs8083289	G	C	0.1948	0.3436	0.0317	2.44E-27	0.026	0.0186	0.1611	36.88
rs9378638	T	C	0.3377	0.1762	0.0319	3.40E-08	0.0264	0.0156	0.0914597	13.65

Abbreviations: SNP, single nucleotide polymorphism; se, standard error; BMI, body mass index; SSTI, infections of the skin and subcutaneous tissue; pval, p-value.

Table S8. Detailed information about single-nucleotide polymorphisms of body mass index on acute lower respiratory infections.

SNP	effect_allele BMI	other_alleleBMI	eaf.ALRI	beta.BMI	se.BMI	pval.BMI	beta.ALRI	se. ALRI	pval.ALRI	F-statistics
rs10203386	A	T	0.3949	0.1623	0.0274	3.35E-09	0.027	0.015	0.0717398	16.77
rs13011109	C	G	0.4205	-0.1757	0.0287	8.69E-10	0.0268	0.0149	0.07172	18.27
rs13130484	T	C	0.4728	0.1666	0.028	2.61E-09	0.0049	0.0147	0.7366	17.65
rs1320336	A	G	0.1626	-0.3025	0.0367	1.56E-16	-0.0213	0.0199	0.2838	18.51
rs1342396	T	C	0.6913	0.1678	0.0292	8.72E-09	-0.0057	0.0158	0.716101	14.10
rs4776970	T	A	0.3452	-0.1729	0.0287	1.71E-09	0.0082	0.0154	0.5944	16.41
rs543874	G	A	0.1782	0.2652	0.0333	1.67E-15	-0.0088	0.0191	0.6458	18.58
rs62033400	G	A	0.4025	0.3768	0.0286	1.26E-39	0.0117	0.0149	0.4315	83.62
rs9378638	T	C	0.3377	0.1762	0.0319	3.40E-08	-0.0016	0.0156	0.9156	13.65

Abbreviations: SNP, single nucleotide polymorphism; se, standard error; BMI, body mass index; ALRI, acute lower respiratory infections; pval, p-value.

Table S9. Detailed information about single-nucleotide polymorphisms of hip circumference on sepsis susceptibility.

SNP	effect_allele HC	other_allele HC	eaf. HC	beta. HC	se. HC	pval. HC	beta.SS	se. SS	pval.SS	F-statistics
rs1006399	A	G	0.458873	-0.01149	0.002001	9.50E-09	-0.00788	0.013735	0.566169	16.36
rs10069930	A	T	0.506036	0.012125	0.002054	3.60E-09	-0.026	0.014092	0.06501	17.42
rs10100245	A	G	0.564506	0.020539	0.002012	1.80E-24	0.000816	0.013779	0.952805	51.24
rs10118701	G	A	0.32209	0.016807	0.002131	3.10E-15	0.026543	0.014635	0.069732	27.17
rs10132514	T	C	0.273182	-0.01314	0.002255	5.60E-09	-0.01141	0.01544	0.460038	13.49
rs1013402	G	A	0.318427	0.02937	0.002136	5.10E-43	0.016945	0.014668	0.247992	82.08
rs10145154	T	C	0.22178	0.025384	0.002404	4.60E-26	0.016779	0.016489	0.308891	38.49
rs10210468	C	T	0.464304	-0.01376	0.002019	9.60E-12	-0.00972	0.013887	0.484019	23.09
rs1023767	A	G	0.235192	-0.01535	0.002348	6.20E-11	-0.04465	0.016089	0.005519	15.38
rs1037702	A	G	0.62178	-0.01162	0.00206	1.70E-08	0.002703	0.014131	0.848286	14.97
rs10404726	T	C	0.465501	-0.01665	0.002003	9.60E-17	0.004599	0.013713	0.73733	34.36
rs10407871	C	T	0.155426	-0.0182	0.002765	4.70E-11	-0.0003	0.018907	0.98731	11.37
rs10471636	A	G	0.508884	-0.01183	0.002032	5.70E-09	0.006093	0.013942	0.662096	16.95
rs1047891	A	C	0.315753	0.016603	0.002137	7.80E-15	0.007571	0.01468	0.606021	26.09
rs1056720	T	C	0.234833	-0.01306	0.002351	2.80E-08	0.009696	0.016097	0.546942	11.10
rs10744145	A	C	0.513795	-0.01212	0.001997	1.30E-09	0.00843	0.013693	0.538102	18.40
rs10746833	G	A	0.581617	-0.01494	0.002036	2.20E-13	-0.01188	0.013951	0.394351	26.21
rs10773051	T	C	0.222167	0.023142	0.002403	5.90E-22	-0.01737	0.016494	0.292211	32.06
rs10810598	A	T	0.639535	-0.01293	0.002078	4.90E-10	-0.04807	0.01425	0.000743	17.84
rs10883553	A	C	0.445855	0.015105	0.002007	5.20E-14	-0.02782	0.013762	0.043225	27.99
rs10938397	G	A	0.434366	0.02439	0.002011	7.70E-34	0.019091	0.013809	0.166821	72.26
rs10954284	A	T	0.488124	0.019314	0.001992	3.20E-22	-0.00293	0.013674	0.83017	46.96
rs10987417	T	G	0.385638	0.012808	0.00207	6.10E-10	0.004287	0.014187	0.762516	18.14

SNP	effect_allele HC	other_allele HC	eaf. HC	beta. HC	se. HC	pval. HC	beta.SS	se. SS	pval.SS	F-statistics
rs11012732	G	A	0.331674	0.020089	0.002119	2.50E-21	0.020107	0.014513	0.165904	39.86
rs11030016	T	C	0.739706	0.018143	0.002276	1.60E-15	0.012272	0.015621	0.43211	24.47
rs11045163	G	A	0.430597	0.011451	0.002016	1.30E-08	0.012495	0.01383	0.366282	15.82
rs11075252	G	A	0.284481	-0.01256	0.002215	1.40E-08	-0.01746	0.015182	0.250146	13.10
rs1108548	G	A	0.27725	0.015652	0.002227	2.10E-12	-0.00151	0.015314	0.921641	19.79
rs11107114	A	G	0.228885	0.013324	0.002375	2.00E-08	0.002603	0.016304	0.873163	11.11
rs11113445	G	A	0.391358	0.01275	0.002039	4.00E-10	0.015337	0.013994	0.273066	18.64
rs11150461	G	C	0.727351	-0.01416	0.002247	3.00E-10	-0.01491	0.015418	0.333436	15.74
rs11150745	G	A	0.317702	-0.02028	0.002146	3.40E-21	-0.00506	0.014695	0.730745	38.70
rs11164630	T	C	0.608331	-0.01381	0.002042	1.30E-11	0.028274	0.014034	0.043945	21.81
rs11165643	T	C	0.590085	0.018526	0.00202	4.70E-20	0.017869	0.013894	0.198405	40.68
rs11173522	A	C	0.214329	0.013694	0.002429	1.70E-08	0.01074	0.01665	0.51888	10.70
rs1123295	G	A	0.444369	0.011954	0.002005	2.50E-09	-0.01511	0.013788	0.273213	17.55
rs11263719	T	C	0.469657	0.011499	0.002008	1.00E-08	-0.0133	0.013754	0.333487	16.33
rs112875651	A	G	0.391041	0.018266	0.002068	1.00E-18	-0.02219	0.014184	0.117654	37.14
rs113230003	A	G	0.260523	-0.01486	0.002292	9.00E-11	0.000887	0.015692	0.954913	16.19
rs11513729	T	C	0.413028	-0.01665	0.002051	4.70E-16	-0.00921	0.01409	0.513229	31.97
rs11664106	T	A	0.37403	0.016775	0.002113	2.00E-15	-0.00677	0.014467	0.639667	29.51
rs11704728	T	C	0.196356	0.014832	0.002524	4.20E-09	0.022957	0.017294	0.184362	10.90
rs11751684	T	G	0.275389	0.016732	0.002223	5.20E-14	-0.01285	0.015294	0.400831	22.60
rs11778934	G	C	0.536083	-0.01413	0.002005	1.80E-12	0.02861	0.013751	0.037476	24.71
rs11779446	G	A	0.161259	-0.02018	0.00272	1.20E-13	0.015825	0.018622	0.395429	14.89
rs1182199	A	C	0.304395	-0.02465	0.002167	5.40E-30	0.009913	0.01484	0.504142	54.83
rs11882796	T	A	0.541285	-0.01138	0.002003	1.30E-08	-0.00716	0.013764	0.602844	16.04

SNP	effect_allele HC	other_allele HC	eaf. HC	beta. HC	se. HC	pval. HC	beta.SS	se. SS	pval.SS	F-statistics
rs11997077	G	A	0.380458	-0.01129	0.002066	4.70E-08	-0.01755	0.014171	0.215455	14.07
rs12072739	G	A	0.224461	0.01499	0.002387	3.40E-10	0.005467	0.016423	0.739205	13.73
rs12122361	G	A	0.265617	-0.01377	0.00226	1.10E-09	0.020431	0.015558	0.189107	14.49
rs12128526	A	G	0.457416	0.011368	0.001996	1.20E-08	0.006007	0.013736	0.661896	16.10
rs12364470	G	T	0.164552	0.017881	0.002687	2.80E-11	-0.00322	0.018403	0.861021	12.17
rs12375196	A	C	0.424347	0.016759	0.002028	1.40E-16	0.016871	0.013896	0.224705	33.36
rs12427047	T	C	0.24273	-0.0164	0.002323	1.70E-12	0.01361	0.015937	0.393121	18.32
rs12441543	A	G	0.2871	0.015234	0.002209	5.30E-12	0.003466	0.015135	0.818865	19.47
rs12519997	A	G	0.559278	-0.014	0.002004	2.90E-12	0.026166	0.013758	0.057189	24.05
rs12568411	A	G	0.166749	0.016481	0.002694	9.40E-10	-0.01004	0.018526	0.588008	10.40
rs12569355	G	A	0.119536	0.021744	0.003077	1.60E-12	-0.02543	0.02114	0.228968	10.51
rs1260326	C	T	0.604267	0.012037	0.00203	3.00E-09	0.007709	0.01397	0.581085	16.81
rs12607512	G	A	0.44632	0.011784	0.002008	4.40E-09	-0.00792	0.013753	0.564599	17.02
rs12631813	G	C	0.50262	-0.01227	0.002	8.50E-10	0.016574	0.013756	0.228234	18.82
rs12762744	T	C	0.248261	0.013317	0.002312	8.40E-09	-0.01236	0.015844	0.435194	12.38
rs12831185	G	A	0.170442	-0.01764	0.002655	3.10E-11	-0.00763	0.018219	0.675384	12.48
rs1285992	G	A	0.710447	0.020316	0.002203	2.90E-20	-0.01254	0.015078	0.405508	34.99
rs12880641	G	T	0.661578	-0.01492	0.002106	1.40E-12	-0.03024	0.014401	0.035759	22.47
rs12883788	T	C	0.459801	0.014386	0.002009	7.90E-13	0.032985	0.013746	0.016414	25.48
rs12921916	C	T	0.28756	0.012596	0.002228	1.60E-08	0.003815	0.015271	0.802718	13.10
rs12939848	T	C	0.401034	0.01264	0.002038	5.60E-10	0.000295	0.013985	0.983168	18.48
rs1294438	T	C	0.354583	0.018627	0.002108	9.70E-19	-0.00805	0.014464	0.577701	35.76
rs1294438	T	C	0.354583	0.018627	0.002108	9.70E-19	0.107669	0.109797	0.326779	35.76
rs1296328	C	A	0.559033	-0.01582	0.002015	4.20E-15	0.00413	0.013825	0.765147	30.37

SNP	effect_allele HC	other_allele HC	eaf. HC	beta. HC	se. HC	pval. HC	beta.SS	se. SS	pval.SS	F-statistics
rs13017207	A	G	0.393634	-0.01615	0.002034	2.00E-15	-0.02087	0.013984	0.135682	30.11
rs13034765	C	G	0.370011	0.013375	0.002064	9.10E-11	0.009832	0.014206	0.488864	19.58
rs13107325	T	C	0.074895	0.038665	0.003786	1.70E-24	-0.01999	0.02602	0.442261	14.46
rs13148263	A	G	0.335162	0.015944	0.002105	3.60E-14	0.007812	0.014456	0.588933	25.57
rs13156484	A	G	0.472171	-0.01749	0.002015	3.90E-18	-0.01669	0.013816	0.227028	37.58
rs13284988	C	T	0.305411	0.012076	0.002179	3.00E-08	0.02048	0.014929	0.17012	13.04
rs13292699	C	A	0.433708	-0.01627	0.002015	6.70E-16	0.0085	0.013804	0.538043	32.05
rs133015	G	C	0.439948	0.012962	0.002016	1.30E-10	0.007521	0.013772	0.585016	20.38
rs13333747	C	T	0.182674	-0.02197	0.002592	2.30E-17	-0.00163	0.017754	0.927018	21.46
rs13374459	C	T	0.411989	0.012675	0.002023	3.70E-10	0.004873	0.013911	0.726109	19.02
rs13389219	T	C	0.392395	0.02276	0.002034	4.70E-29	-0.03604	0.01398	0.009927	59.69
rs143384	G	A	0.404382	0.02675	0.002031	1.30E-39	0.013172	0.013917	0.343899	83.55
rs1446585	G	A	0.244506	-0.01285	0.002268	1.50E-08	-0.02283	0.016353	0.162695	11.86
rs1452082	A	C	0.557071	0.012586	0.00204	6.80E-10	0.001946	0.013999	0.889439	18.79
rs1458156	T	C	0.488404	0.012649	0.001995	2.30E-10	-0.0135	0.013687	0.323943	20.10
rs1477290	C	T	0.136945	0.029863	0.002921	1.60E-24	0.038369	0.020037	0.055505	24.70
rs147730268	T	G	0.087233	-0.05061	0.003611	1.30E-44	-0.03586	0.024825	0.148651	31.28
rs1480474	G	A	0.417444	-0.01451	0.002023	7.40E-13	0.017683	0.013892	0.203049	25.01
rs1481630	C	T	0.176871	-0.01539	0.002615	4.00E-09	-0.03382	0.017987	0.060044	10.09
rs1502317	T	C	0.27657	-0.01516	0.002232	1.10E-11	0.003686	0.015309	0.809727	18.46
rs1514895	G	A	0.285289	0.01747	0.002202	2.10E-15	0.02087	0.015123	0.167584	25.67
rs1538535	T	C	0.27456	0.012439	0.002237	2.70E-08	0.024756	0.015328	0.106288	12.32
rs1573736	T	G	0.345277	-0.0128	0.002112	1.40E-09	-0.006	0.014473	0.678524	16.60
rs1576655	C	A	0.59598	0.016133	0.002075	7.70E-15	-0.0076	0.014231	0.593455	29.10

SNP	effect_allele HC	other_allele HC	eaf. HC	beta. HC	se. HC	pval. HC	beta.SS	se. SS	pval.SS	F-statistics
rs1609783	A	G	0.525344	0.014272	0.002004	1.10E-12	0.002294	0.013771	0.867691	25.28
rs16868443	C	G	0.360418	0.015435	0.002076	1.00E-13	0.010911	0.014228	0.443166	25.50
rs170016	A	G	0.633948	-0.01165	0.002093	2.60E-08	-0.00746	0.014358	0.603568	14.37
rs17361789	G	T	0.32182	0.012642	0.002148	4.00E-09	-0.01681	0.014765	0.254996	15.12
rs17639546	A	G	0.14849	-0.01877	0.0028	2.00E-11	-0.01662	0.019219	0.387088	11.36
rs17733217	G	A	0.229127	-0.01581	0.002379	3.10E-11	-0.01409	0.016292	0.386997	15.59
rs17766836	T	C	0.267888	0.023232	0.002252	6.10E-25	-0.00326	0.015437	0.832563	41.73
rs17770336	T	C	0.322443	0.019146	0.002129	2.40E-19	0.005309	0.014602	0.716166	35.34
rs1813212	G	A	0.445576	-0.01301	0.002008	9.20E-11	0.021508	0.013768	0.11825	20.74
rs1868069	A	G	0.229248	0.015095	0.002369	1.90E-10	0.015808	0.016285	0.331686	14.34
rs1934394	C	G	0.228157	0.012999	0.002383	4.90E-08	-0.00815	0.01639	0.619012	10.48
rs1955695	G	A	0.62227	-0.01712	0.002059	8.90E-17	-0.01343	0.014119	0.341377	32.53
rs2022050	A	G	0.160924	-0.02088	0.00272	1.70E-14	-0.02215	0.018665	0.235427	15.90
rs2023211	C	T	0.232254	0.016039	0.002362	1.10E-11	0.028561	0.016182	0.077566	16.45
rs211434	A	G	0.681996	0.013134	0.002141	8.60E-10	0.011312	0.014703	0.441692	16.32
rs2133292	T	C	0.373582	0.01323	0.002066	1.50E-10	-0.0181	0.014179	0.201689	19.20
rs2133561	T	A	0.611067	-0.01819	0.002064	1.20E-18	-0.01432	0.014165	0.312017	36.92
rs2151248	C	G	0.730659	-0.01493	0.00224	2.60E-11	0.000738	0.015413	0.961826	17.49
rs2159437	G	A	0.517573	0.019624	0.001998	8.90E-23	-0.00017	0.013692	0.989859	48.20
rs2172131	C	T	0.578698	-0.01369	0.00202	1.20E-11	-0.01233	0.013853	0.373363	22.39
rs2178385	T	G	0.320337	0.018547	0.002133	3.40E-18	-0.01815	0.014671	0.215983	32.93
rs2238435	G	C	0.613709	0.025955	0.002052	1.10E-36	0.023188	0.01408	0.099584	75.89
rs2238689	C	T	0.399438	-0.01417	0.002037	3.60E-12	0.005701	0.013949	0.682738	23.19
rs2253310	G	C	0.626098	0.019536	0.002056	2.10E-21	0.026259	0.014129	0.0631	42.27

SNP	effect_allele HC	other_allele HC	eaf. HC	beta. HC	se. HC	pval. HC	beta.SS	se. SS	pval.SS	F-statistics
rs2270894	G	C	0.203219	-0.02164	0.002568	3.50E-17	-0.01888	0.017623	0.284035	23.01
rs2288745	T	C	0.299451	0.013663	0.002177	3.50E-10	0.018952	0.014966	0.205415	16.53
rs2307111	C	T	0.394977	-0.02854	0.002038	1.60E-44	-0.00104	0.013991	0.940657	93.69
rs2371911	A	T	0.544326	0.013106	0.001999	5.50E-11	0.036532	0.013722	0.007762	21.33
rs2384054	C	T	0.489428	0.021906	0.001987	2.90E-28	-0.00757	0.013673	0.579895	60.76
rs2479958	G	A	0.516982	-0.01299	0.002019	1.20E-10	-0.02462	0.013836	0.0752	20.67
rs2494196	A	C	0.286135	0.031274	0.002199	6.90E-46	0.003548	0.015123	0.814509	82.61
rs2499468	A	C	0.651137	0.013183	0.00209	2.80E-10	0.006906	0.014363	0.630673	18.07
rs253444	A	G	0.12923	-0.01982	0.002972	2.50E-11	-0.01157	0.020411	0.570692	10.01
rs2568958	A	G	0.603693	0.020671	0.002029	2.20E-24	0.000633	0.013955	0.96383	49.67
rs2577955	T	C	0.802767	-0.01467	0.002505	4.80E-09	0.005661	0.017157	0.741419	10.85
rs25849	G	C	0.288829	0.019767	0.002208	3.50E-19	-0.01506	0.015154	0.320331	32.92
rs2585526	G	A	0.557413	0.012267	0.002011	1.10E-09	0.016942	0.013782	0.218976	18.35
rs2593169	C	G	0.567965	-0.01163	0.002018	8.20E-09	0.010202	0.013819	0.460369	16.31
rs2606227	C	T	0.63145	-0.01243	0.002083	2.40E-09	0.012758	0.014307	0.372538	16.59
rs2642305	T	A	0.369566	0.011304	0.002067	4.50E-08	0.035855	0.014161	0.011346	13.94
rs2660241	C	T	0.364912	0.013914	0.002073	1.90E-11	0.001152	0.014202	0.935369	20.88
rs2678204	G	T	0.340172	0.019675	0.002099	7.10E-21	0.029017	0.01446	0.044784	39.44
rs273505	C	T	0.422021	0.012874	0.002021	1.90E-10	0.024731	0.013844	0.074025	19.79
rs2737263	T	G	0.28052	-0.02366	0.00222	1.60E-26	-0.04119	0.015233	0.00685	45.84
rs2744956	C	T	0.139254	0.053758	0.00287	2.80E-78	0.0284	0.019705	0.1495	84.11
rs2814350	A	G	0.304993	0.013872	0.002209	3.40E-10	-0.00133	0.015147	0.929811	16.72
rs2821226	G	A	0.527763	0.015138	0.002004	4.30E-14	0.023044	0.01378	0.094471	28.44
rs28366156	C	T	0.130582	-0.02658	0.002952	2.20E-19	-0.03504	0.020251	0.083601	18.41

SNP	effect_allele HC	other_allele HC	eaf. HC	beta. HC	se. HC	pval. HC	beta.SS	se. SS	pval.SS	F-statistics
rs28457680	A	T	0.136611	0.019523	0.00292	2.30E-11	0.019189	0.020011	0.337585	10.55
rs2861685	C	T	0.411983	-0.01478	0.002015	2.30E-13	0.002265	0.013847	0.870047	26.05
rs28711392	C	T	0.367255	-0.01252	0.002084	1.90E-09	-0.00978	0.014292	0.4939	16.78
rs28778940	A	G	0.330838	0.015151	0.002124	9.70E-13	-0.01592	0.014532	0.273205	22.54
rs287837	G	A	0.523844	-0.01308	0.002002	6.50E-11	0.000844	0.01376	0.951071	21.28
rs28971796	G	A	0.620409	-0.01515	0.002276	2.80E-11	-0.01235	0.015638	0.429523	20.86
rs3012053	G	A	0.714878	-0.01497	0.002209	1.20E-11	-0.00033	0.015124	0.982849	18.73
rs308911	G	A	0.714423	-0.01428	0.002206	9.70E-11	-0.00451	0.015154	0.765947	17.09
rs310796	T	G	0.680961	0.012109	0.002144	1.60E-08	-0.00718	0.014708	0.625371	13.86
rs3110942	A	G	0.481664	0.012712	0.001998	2.00E-10	0.021261	0.013694	0.120509	20.21
rs3116600	G	A	0.215319	-0.01877	0.002425	1.00E-14	0.029901	0.016611	0.071851	20.24
rs314288	C	T	0.886256	-0.02271	0.003138	4.60E-13	0.025729	0.021534	0.232162	10.56
rs329118	T	C	0.419386	-0.01635	0.00202	5.80E-16	-0.04353	0.013856	0.001682	31.90
rs33503	A	G	0.80589	-0.0175	0.002514	3.40E-12	0.002585	0.017256	0.880904	15.16
rs340025	C	T	0.57994	0.012741	0.002028	3.30E-10	-0.01005	0.01388	0.469137	19.23
rs34223321	A	C	0.300092	0.014092	0.002173	8.80E-11	0.01631	0.014875	0.272872	17.67
rs34517439	A	C	0.121795	0.045455	0.003076	2.00E-49	0.048766	0.021172	0.021261	46.73
rs34594435	T	C	0.195585	0.021579	0.002512	8.60E-18	-0.01548	0.017229	0.36896	23.22
rs34629844	G	A	0.12828	0.021295	0.002976	8.30E-13	-0.02409	0.02041	0.237825	11.45
rs34656389	G	A	0.367473	0.011297	0.002066	4.60E-08	0.014893	0.014186	0.293778	13.89
rs34765854	G	T	0.298258	0.019332	0.002172	5.50E-19	0.021052	0.014911	0.157991	33.17
rs34769775	T	C	0.29735	-0.01411	0.002183	1.00E-10	-0.01705	0.014952	0.254057	17.46
rs34772064	G	T	0.555918	-0.01187	0.002003	3.10E-09	0.011156	0.013763	0.417591	17.35
rs34811474	A	G	0.230735	-0.01979	0.002362	5.40E-17	-0.00586	0.01622	0.718019	24.92

SNP	effect_allele HC	other_allele HC	eaf. HC	beta. HC	se. HC	pval. HC	beta.SS	se. SS	pval.SS	F-statistics
rs34840745	T	C	0.262486	0.016643	0.002272	2.40E-13	0.00027	0.015545	0.986128	20.78
rs34848742	G	T	0.788211	-0.01436	0.002436	3.70E-09	0.004306	0.016742	0.79702	11.60
rs35506085	A	G	0.185085	-0.02048	0.002586	2.40E-15	-0.03398	0.017771	0.055878	18.92
rs35537311	T	C	0.388607	-0.01476	0.00205	6.00E-13	-0.00624	0.014043	0.65672	24.65
rs35792595	A	T	0.297453	0.013773	0.002192	3.30E-10	-0.01573	0.015031	0.295449	16.51
rs35882248	T	C	0.317213	0.015668	0.002138	2.30E-13	0.022521	0.014705	0.125634	23.27
rs35910339	G	C	0.683708	-0.0127	0.002142	3.00E-09	-0.02365	0.014686	0.107363	15.21
rs35917007	G	A	0.533673	0.014757	0.001999	1.50E-13	0.011654	0.013684	0.394427	27.14
rs36140	C	A	0.635313	0.014843	0.00208	9.50E-13	0.012909	0.014287	0.366203	23.61
rs365352	A	G	0.24439	-0.02054	0.002313	6.60E-19	0.002024	0.015891	0.898639	29.13
rs3737992	A	G	0.168963	-0.02151	0.002651	4.90E-16	0.009953	0.018224	0.584941	18.49
rs3762988	T	C	0.388074	0.01265	0.002048	6.50E-10	0.016501	0.014052	0.240261	18.12
rs3807566	T	G	0.438301	-0.01495	0.002012	1.10E-13	-0.01038	0.013787	0.451351	27.18
rs3810291	A	G	0.674991	0.022735	0.002129	1.30E-26	0.01288	0.014588	0.377257	50.05
rs3811951	G	A	0.282058	0.015018	0.002211	1.10E-11	0.006416	0.015163	0.67217	18.68
rs3814883	T	C	0.482403	0.025069	0.002	5.00E-36	0.009265	0.013734	0.499929	78.43
rs3826408	T	C	0.456783	0.013589	0.002001	1.10E-11	0.00298	0.013712	0.82794	22.89
rs3845344	T	C	0.391164	0.01387	0.002036	9.70E-12	0.027762	0.013997	0.047315	22.10
rs3935190	A	G	0.536778	-0.01411	0.002012	2.30E-12	-0.01666	0.013779	0.226519	24.46
rs394608	C	T	0.537697	0.014611	0.00201	3.60E-13	0.009763	0.013736	0.477264	26.27
rs40071	C	T	0.179489	-0.01741	0.002603	2.20E-11	0.013166	0.017855	0.46088	13.18
rs4017425	T	C	0.470189	-0.01184	0.001997	3.10E-09	-0.01111	0.013719	0.418142	17.51
rs4240326	G	A	0.550142	-0.02793	0.002001	2.70E-44	-0.03494	0.013735	0.010958	96.50
rs4253755	A	G	0.128527	0.021132	0.003003	2.00E-12	0.001825	0.020542	0.929198	11.09

SNP	effect_allele HC	other_allele HC	eaf. HC	beta. HC	se. HC	pval. HC	beta.SS	se. SS	pval.SS	F-statistics
rs429343	G	A	0.576577	-0.0122	0.002015	1.40E-09	-0.03045	0.013844	0.027854	17.89
rs4310395	G	A	0.442854	0.012218	0.002002	1.00E-09	-0.02152	0.013756	0.117714	18.37
rs4467770	A	G	0.731091	0.015878	0.002254	1.80E-12	0.019203	0.01547	0.214495	19.52
rs4476935	T	C	0.433157	-0.01287	0.002011	1.50E-10	-0.02844	0.013793	0.039234	20.13
rs4477562	T	C	0.128661	0.02922	0.003004	2.30E-22	0.024146	0.02054	0.239778	21.22
rs4486868	C	T	0.447992	-0.01111	0.002011	3.40E-08	-0.01634	0.013765	0.235144	15.08
rs4567604	T	G	0.193192	-0.01668	0.002539	5.00E-11	-0.00761	0.017366	0.661237	13.46
rs4613074	C	T	0.18516	-0.01702	0.002566	3.30E-11	0.009083	0.017637	0.606553	13.28
rs463376	A	G	0.490305	0.011993	0.001998	1.90E-09	0.029177	0.013661	0.032703	18.02
rs4671328	G	T	0.551345	-0.01631	0.002015	5.70E-16	0.014994	0.013848	0.278927	32.43
rs4678016	T	C	0.366307	0.011514	0.002065	2.50E-08	0.01535	0.014204	0.279821	14.43
rs475390	A	G	0.776432	-0.01407	0.002389	3.90E-09	0.023829	0.016418	0.146671	12.04
rs4790292	A	C	0.153689	-0.02619	0.002777	4.10E-21	-0.00939	0.019035	0.621676	23.13
rs4792716	G	A	0.562173	0.014561	0.002012	4.60E-13	0.024812	0.013785	0.07187	25.79
rs4800490	C	A	0.495235	-0.02317	0.001996	3.60E-31	-0.01605	0.013682	0.240836	67.42
rs4820346	G	C	0.69295	-0.01305	0.002171	1.80E-09	-0.00988	0.014849	0.505865	15.38
rs483465	G	A	0.749331	0.015493	0.002297	1.50E-11	-0.02557	0.015777	0.105119	17.09
rs4843158	C	G	0.68516	0.021846	0.002149	2.80E-24	-0.01382	0.014708	0.347551	44.59
rs4870057	G	A	0.341223	0.012693	0.002127	2.40E-09	-0.00043	0.014609	0.976297	16.01
rs4909309	C	T	0.393916	-0.01735	0.002039	1.70E-17	-0.00245	0.013986	0.860735	34.58
rs4962424	A	T	0.326831	0.015585	0.002127	2.40E-13	-0.00083	0.014572	0.954369	23.61
rs4963975	A	G	0.244315	-0.01879	0.002324	6.10E-16	0.0137	0.015946	0.390272	24.15
rs4970946	G	A	0.217454	0.014119	0.002409	4.60E-09	0.003632	0.016584	0.826638	11.69
rs4976994	G	A	0.452549	0.012839	0.002005	1.50E-10	0.004513	0.013733	0.742433	20.32

SNP	effect_allele HC	other_allele HC	eaf. HC	beta. HC	se. HC	pval. HC	beta.SS	se. SS	pval.SS	F-statistics
rs522110	G	A	0.555042	0.018119	0.002007	1.70E-19	0.01296	0.013767	0.346512	40.27
rs543874	G	A	0.205218	0.045364	0.002462	8.00E-76	0.024784	0.016909	0.142718	110.79
rs551935	G	T	0.440778	-0.01693	0.002005	3.10E-17	-0.00021	0.013749	0.987984	35.13
rs55726687	A	G	0.209736	0.021817	0.002445	4.50E-19	0.013979	0.016782	0.404846	26.40
rs56094641	G	A	0.404596	0.061633	0.002031	1.00E-200	0.022914	0.013924	0.099837	444.31
rs56097510	G	C	0.51052	0.013713	0.001998	6.70E-12	0.009852	0.013681	0.471444	23.54
rs56288810	G	A	0.213142	0.013615	0.002448	2.70E-08	0.054145	0.016769	0.001243	10.37
rs56399737	T	C	0.449109	-0.01182	0.002013	4.30E-09	0.016022	0.013776	0.244801	17.07
rs57222629	C	G	0.311673	0.013815	0.002156	1.50E-10	0.020785	0.014803	0.160302	17.61
rs57235969	T	C	0.554199	0.012462	0.002	4.60E-10	-0.00838	0.013747	0.541975	19.19
rs57636386	C	T	0.08382	-0.03508	0.003612	2.60E-22	-0.0169	0.024673	0.493323	14.49
rs57989773	C	T	0.245008	0.021874	0.002381	4.00E-20	0.020442	0.016346	0.21108	31.24
rs58584712	A	G	0.211251	0.016977	0.002436	3.20E-12	-0.00403	0.016752	0.809998	16.19
rs58862095	T	C	0.419278	-0.01811	0.002024	3.70E-19	-0.01651	0.013881	0.234242	38.96
rs60226453	T	C	0.177273	0.016272	0.0026	3.90E-10	0.043649	0.017931	0.014922	11.42
rs6066104	T	C	0.324108	0.019283	0.002135	1.70E-19	-0.01147	0.014609	0.432242	35.76
rs6142059	C	T	0.492562	0.014997	0.002	6.50E-14	-0.0111	0.013689	0.417296	28.10
rs614520	A	G	0.640637	-0.01303	0.00212	8.00E-10	-0.00143	0.014521	0.921308	17.38
rs61941722	A	G	0.188737	0.015599	0.002566	1.20E-09	0.015616	0.017599	0.374891	11.32
rs61969510	C	T	0.278956	0.012845	0.002243	1.00E-08	0.002399	0.015363	0.875936	13.19
rs62070648	A	G	0.268727	-0.01994	0.00225	7.80E-19	0.006632	0.015414	0.667022	30.88
rs62107261	C	T	0.04832	-0.08085	0.00465	1.10E-67	-0.04568	0.031929	0.152482	27.80
rs62301134	C	T	0.263286	-0.01364	0.002267	1.80E-09	-0.01133	0.015593	0.46736	14.03

SNP	effect_allele HC	other_allele HC	eaf. HC	beta. HC	se. HC	pval. HC	beta.SS	se. SS	pval.SS	F-statistics
rs62396185	C	G	0.259989	-0.03461	0.00227	1.80E-52	-0.00285	0.015624	0.855329	89.44
rs62515438	G	T	0.227665	0.01504	0.002377	2.50E-10	0.035229	0.016278	0.030443	14.08
rs6438656	A	G	0.635867	-0.01196	0.002067	7.20E-09	-0.0118	0.014209	0.406286	15.50
rs6465828	T	G	0.482837	0.016391	0.001992	1.90E-16	-0.02129	0.013658	0.119115	33.82
rs6545714	A	G	0.60145	-0.01583	0.002034	7.10E-15	-0.03394	0.013965	0.015075	29.04
rs6567160	C	T	0.232716	0.052354	0.002361	5.70E-109	-0.00995	0.016151	0.537746	175.71
rs6575340	A	G	0.636028	0.018455	0.002078	6.70E-19	0.024804	0.014238	0.081489	36.51
rs6597975	G	C	0.543875	0.012292	0.002011	9.80E-10	0.011687	0.013808	0.397333	18.53
rs6598540	G	A	0.276885	-0.01479	0.002231	3.40E-11	-0.00683	0.015269	0.654835	17.60
rs6606686	C	G	0.680627	-0.01657	0.002137	8.80E-15	0.005918	0.01465	0.686262	26.15
rs66679256	T	C	0.445805	0.01499	0.002004	7.50E-14	-0.00871	0.013775	0.527032	27.64
rs6669341	G	A	0.582713	-0.01513	0.002015	6.10E-14	0.011883	0.013856	0.391085	27.41
rs667515	C	G	0.386143	-0.01417	0.002053	5.20E-12	-0.01298	0.014081	0.356468	22.58
rs6744646	G	A	0.828323	0.050054	0.002636	2.00E-80	0.065945	0.018129	0.000275	102.60
rs6747657	A	G	0.281723	0.012934	0.002208	4.70E-09	0.029875	0.01517	0.048906	13.89
rs675162	G	A	0.482059	0.018008	0.001995	1.80E-19	0.004586	0.013707	0.737943	40.69
rs6821305	C	A	0.399287	0.014897	0.002032	2.30E-13	-0.02337	0.013962	0.094169	25.77
rs6868125	T	C	0.521906	-0.01195	0.001988	1.90E-09	0.011475	0.013665	0.401066	18.02
rs6946415	G	A	0.627099	0.015883	0.002064	1.40E-14	0.007132	0.014159	0.6145	27.69
rs6958365	T	C	0.411809	-0.01154	0.002036	1.50E-08	0.004161	0.013971	0.765831	15.56
rs698147	G	A	0.543563	-0.01214	0.002001	1.30E-09	-0.02175	0.013731	0.113138	18.28
rs7002088	C	A	0.445199	0.013717	0.002006	7.90E-12	0.028094	0.013757	0.041134	23.11
rs7034554	G	A	0.373815	-0.01242	0.00206	1.60E-09	-0.00246	0.014109	0.86141	17.03

SNP	effect_allele HC	other_allele HC	eaf. HC	beta. HC	se. HC	pval. HC	beta.SS	se. SS	pval.SS	F-statistics
rs7038943	C	T	0.338791	-0.01211	0.002104	8.60E-09	-0.00545	0.014425	0.705528	14.84
rs705165	T	G	0.247646	0.014809	0.002307	1.40E-10	-0.00919	0.015802	0.561067	15.36
rs7094073	T	C	0.191266	0.016348	0.002735	2.30E-09	0.005246	0.018716	0.779247	11.05
rs7111235	C	T	0.493399	0.011542	0.002004	8.40E-09	-0.0209	0.013739	0.128161	16.59
rs7124681	A	C	0.408387	0.020503	0.002022	3.70E-24	-0.02878	0.01387	0.038001	49.67
rs7132908	A	G	0.384469	0.025774	0.002049	2.80E-36	-0.01234	0.014058	0.379938	74.90
rs7171864	A	G	0.660205	0.016199	0.002116	1.90E-14	0.015939	0.014493	0.271428	26.30
rs7218014	C	T	0.197308	0.020767	0.002511	1.30E-16	-0.00739	0.017183	0.666994	21.66
rs7248205	T	C	0.600262	-0.01612	0.002043	3.00E-15	0.016132	0.014002	0.249297	29.87
rs7257083	A	G	0.288605	0.016261	0.002205	1.70E-13	-0.01872	0.015095	0.214889	22.33
rs72634826	A	G	0.259879	-0.01533	0.002299	2.60E-11	-0.04402	0.015793	0.005314	17.11
rs72656010	C	T	0.132181	-0.02525	0.002953	1.20E-17	-0.00484	0.020267	0.811247	16.77
rs7274811	T	G	0.258589	-0.01951	0.002279	1.10E-17	-0.01192	0.015641	0.446108	28.11
rs72756476	C	T	0.141428	-0.01925	0.002873	2.10E-11	0.030201	0.019652	0.12435	10.90
rs72801843	A	T	0.301389	0.018679	0.002172	8.00E-18	-0.00907	0.014894	0.542393	31.14
rs72959041	A	G	0.049137	-0.06625	0.004667	9.50E-46	-0.00512	0.031978	0.872736	18.84
rs73052033	C	T	0.184918	-0.02081	0.002568	5.30E-16	-0.00548	0.017653	0.756305	19.80
rs73175572	G	A	0.111726	0.028208	0.003192	1.00E-18	-0.0188	0.021927	0.391248	15.50
rs73213484	T	A	0.141217	-0.0207	0.00286	4.60E-13	0.004428	0.019691	0.82206	12.70
rs7365	G	A	0.482654	-0.01109	0.001994	2.60E-08	0.000531	0.013679	0.969021	15.46
rs7372674	A	C	0.357244	0.014356	0.002076	4.60E-12	0.019988	0.014256	0.160887	21.97
rs743572	G	A	0.376473	0.015119	0.002057	2.00E-13	-0.00025	0.014113	0.985925	25.35
rs7442885	G	C	0.214027	-0.01987	0.002432	3.10E-16	-0.01327	0.016745	0.428067	22.45
rs7460093	A	G	0.53118	0.013664	0.002014	1.20E-11	0.006804	0.013828	0.622677	22.92

SNP	effect_allele HC	other_allele HC	eaf. HC	beta. HC	se. HC	pval. HC	beta.SS	se. SS	pval.SS	F-statistics
rs74749286	A	G	0.107637	0.025896	0.003236	1.20E-15	0.00794	0.022233	0.72099	12.30
rs7498665	G	A	0.399666	0.031786	0.002036	6.40E-55	0.031473	0.013976	0.024325	116.93
rs750090	C	T	0.35675	-0.0133	0.002101	2.50E-10	-0.01948	0.014422	0.17688	18.38
rs7519259	A	G	0.528388	0.014144	0.002001	1.50E-12	0.021699	0.013745	0.11442	24.91
rs7523668	A	G	0.565827	-0.01372	0.002009	8.60E-12	-0.00719	0.013806	0.602597	22.91
rs756717	A	G	0.399077	-0.01403	0.00206	9.70E-12	0.018268	0.014127	0.195979	22.25
rs7570446	A	C	0.544459	0.011016	0.001994	3.30E-08	0.004467	0.013722	0.744759	15.13
rs7571496	G	A	0.260562	-0.01382	0.002274	1.20E-09	-0.02611	0.01563	0.094829	14.22
rs7582359	A	G	0.331712	-0.01355	0.002121	1.70E-10	0.004905	0.01458	0.736536	18.09
rs7632381	C	T	0.444325	0.027043	0.002	1.20E-41	0.003213	0.013738	0.815078	90.26
rs765874	A	T	0.489353	-0.0168	0.00199	3.10E-17	0.012437	0.013655	0.362387	35.62
rs76647086	T	G	0.176857	-0.02022	0.002603	8.00E-15	-0.00238	0.01791	0.894498	17.57
rs76798800	T	G	0.266025	0.024423	0.002253	2.20E-27	0.013775	0.015509	0.374453	45.89
rs7695177	G	C	0.470976	-0.01515	0.001995	3.10E-14	0.01461	0.013701	0.286262	28.75
rs7696175	C	T	0.526478	0.013607	0.002004	1.10E-11	0.011038	0.013824	0.424577	22.99
rs7707394	A	G	0.357269	-0.01847	0.002075	5.50E-19	0.007851	0.014253	0.581735	36.39
rs7708584	G	A	0.572378	-0.0132	0.002011	5.30E-11	-0.01166	0.013797	0.398141	21.08
rs7740107	A	T	0.736289	-0.02365	0.002257	1.10E-25	-0.02367	0.015486	0.126352	42.65
rs7793674	C	A	0.144347	0.019239	0.002844	1.30E-11	-0.00565	0.019455	0.771514	11.31
rs779655	C	G	0.729184	0.014326	0.002241	1.60E-10	0.001256	0.015366	0.93487	16.15
rs7805441	T	C	0.502255	0.012684	0.002005	2.50E-10	-0.00387	0.013758	0.778626	20.01
rs7864465	A	G	0.559379	-0.01108	0.002006	3.30E-08	0.002474	0.013756	0.857276	15.04
rs7893571	T	G	0.665871	0.013838	0.002119	6.50E-11	0.036203	0.014512	0.012607	18.98
rs7903146	T	C	0.290662	-0.02141	0.002194	1.70E-22	-0.01684	0.015037	0.262794	39.29

SNP	effect_allele HC	other_allele HC	eaf. HC	beta. HC	se. HC	pval. HC	beta.SS	se. SS	pval.SS	F-statistics
rs7944782	G	T	0.50981	0.012958	0.002003	9.90E-11	0.009508	0.013739	0.488906	20.91
rs7952436	T	C	0.081996	-0.03546	0.003632	1.60E-22	0.036137	0.024885	0.146459	14.35
rs7996639	A	G	0.449371	0.013714	0.002019	1.10E-11	-0.00345	0.013841	0.803056	22.84
rs8030456	T	C	0.226311	-0.02477	0.002379	2.10E-25	-0.00359	0.016313	0.825607	37.98
rs8132129	T	C	0.184901	-0.01592	0.002595	8.40E-10	0.00018	0.01775	0.99192	11.35
rs8133137	G	A	0.664097	0.015778	0.002115	8.80E-14	0.003234	0.014483	0.823324	24.82
rs815163	C	T	0.563175	-0.0169	0.002003	3.20E-17	0.007071	0.013776	0.607754	35.03
rs852042	G	A	0.758582	-0.01537	0.00233	4.30E-11	-0.03454	0.01596	0.030441	15.93
rs852983	A	G	0.459576	-0.01187	0.001997	2.80E-09	-0.00502	0.013697	0.713954	17.53
rs882378	C	A	0.306629	0.015255	0.002167	1.90E-12	0.002954	0.014848	0.842282	21.07
rs897186	G	A	0.548592	-0.01606	0.001999	9.20E-16	-0.00646	0.01372	0.637561	31.99
rs9284814	A	G	0.884815	0.02302	0.003115	1.50E-13	0.039492	0.021464	0.065773	11.13
rs9378684	T	C	0.200589	0.018067	0.002507	5.80E-13	0.006153	0.01722	0.720847	16.65
rs9385385	T	C	0.447856	0.012816	0.002015	2.00E-10	-0.00343	0.013844	0.804478	20.00
rs9512696	G	A	0.66154	0.017211	0.002111	3.60E-16	-0.00521	0.014473	0.718641	29.77
rs9513018	T	G	0.616147	-0.01124	0.002054	4.50E-08	-0.00819	0.014071	0.560392	14.15
rs968379	T	C	0.229098	-0.01878	0.002372	2.40E-15	-0.01915	0.0163	0.239983	22.14
rs9764678	C	T	0.272455	0.016129	0.00225	7.60E-13	0.005011	0.015446	0.745636	20.37
rs9788550	C	G	0.247466	-0.02141	0.00232	2.70E-20	-0.00976	0.015901	0.539293	31.73
rs9830592	A	C	0.582425	0.013836	0.002018	7.10E-12	0.006051	0.013863	0.662467	22.86
rs9843653	C	T	0.511657	0.018327	0.001992	3.60E-20	0.013888	0.013692	0.310423	42.30
rs9845755	T	A	0.198555	0.027361	0.002498	6.30E-28	0.011005	0.017153	0.521149	38.19
rs9850529	A	G	0.339796	0.011482	0.002099	4.50E-08	0.000657	0.014429	0.963686	13.43
rs987237	G	A	0.17957	0.034454	0.002591	2.50E-40	-0.00166	0.017822	0.925938	52.09

SNP	effect_allele HC	other_allele HC	eaf. HC	beta. HC	se. HC	pval. HC	beta.SS	se. SS	pval.SS	F-statistics
rs9876664	T	G	0.3754	-0.01438	0.002059	2.90E-12	-0.00094	0.014139	0.947053	22.87
rs9951619	G	T	0.767352	0.013853	0.002376	5.60E-09	0.009064	0.016295	0.578051	12.13
rs9967287	T	G	0.251722	0.014145	0.002301	7.80E-10	0.012926	0.015763	0.412194	14.24
rs9967367	T	C	0.293494	-0.01742	0.002204	2.70E-15	0.013756	0.015087	0.361902	25.90
rs998584	A	C	0.482802	-0.02227	0.001995	5.90E-29	-0.00222	0.013695	0.871436	62.28

Abbreviations: SNP, single nucleotide polymorphism; se, standard error; HC, hip circumference; SS, sepsis susceptibility; pval, p-value.

Table S10. Detailed information about single-nucleotide polymorphisms of hip circumference on sepsis mortality.

SNP	effect_allele HC	other_alleleHC	eaf.HC	beta.HC	se.HC	pval.HC	beta.SM	se.SM	pval.SM	F-statistics
rs1006399	A	G	0.458873	-0.0114857	0.00200117	9.50E-09	0.0167913	0.0335162	0.616378	16.36
rs10069930	A	T	0.506036	0.0121252	0.00205404	3.60E-09	0.00307852	0.0344602	0.928815	17.42
rs10100245	A	G	0.564506	0.0205386	0.0020121	1.80E-24	0.000375709	0.0336604	0.991094	51.24
rs10118701	G	A	0.32209	0.0168068	0.00213097	3.10E-15	0.0266649	0.0356843	0.454916	27.17
rs10132514	T	C	0.273182	-0.0131415	0.00225463	5.60E-09	0.0530021	0.0376714	0.15944	13.49
rs1013402	G	A	0.318427	0.0293703	0.002136	5.10E-43	0.00560092	0.035741	0.875474	82.08
rs10145154	T	C	0.22178	0.0253835	0.00240401	4.60E-26	0.0491398	0.0402904	0.222601	38.49
rs10210468	C	T	0.464304	-0.0137575	0.00201939	9.60E-12	0.00257027	0.0339093	0.939579	23.09
rs1023767	A	G	0.235192	-0.015354	0.00234795	6.20E-11	-0.089577	0.039329	0.0227484	15.38
rs1037702	A	G	0.62178	-0.0116182	0.00205972	1.70E-08	-0.0237928	0.0344567	0.489871	14.97
rs10404726	T	C	0.465501	-0.0166459	0.00200329	9.60E-17	0.00340267	0.0335073	0.919114	34.36
rs10407871	C	T	0.155426	-0.0181966	0.00276505	4.70E-11	-0.0342937	0.0461144	0.457079	11.37
rs10471636	A	G	0.508884	-0.0118317	0.00203156	5.70E-09	0.00483721	0.0340038	0.886879	16.95
rs1047891	A	C	0.315753	0.0166025	0.00213661	7.80E-15	0.00920993	0.0359048	0.797557	26.09
rs1056720	T	C	0.234833	-0.0130647	0.00235122	2.80E-08	0.010044	0.0392577	0.798068	11.10
rs10744145	A	C	0.513795	-0.0121183	0.00199668	1.30E-09	-0.0305424	0.0334029	0.360525	18.40
rs10746833	G	A	0.581617	-0.0149421	0.0020362	2.20E-13	-0.0230626	0.0340426	0.498113	26.21
rs10773051	T	C	0.222167	0.0231417	0.00240285	5.90E-22	-0.0205585	0.0402678	0.60967	32.06
rs10810598	A	T	0.639535	-0.0129254	0.00207775	4.90E-10	-0.0289518	0.0347936	0.405352	17.84
rs10883553	A	C	0.445855	0.0151046	0.00200686	5.20E-14	-0.022545	0.033577	0.501939	27.99
rs10938397	G	A	0.434366	0.02439	0.00201147	7.70E-34	0.0736398	0.0336828	0.0287959	72.26
rs10954284	A	T	0.488124	0.0193137	0.00199245	3.20E-22	-0.019633	0.0333617	0.556205	46.96
rs10987417	T	G	0.385638	0.0128078	0.00206992	6.10E-10	0.000895087	0.0346063	0.979365	18.14

SNP	effect_allele HC	other_alleleHC	eaf.HC	beta.HC	se.HC	pval.HC	beta.SM	se.SM	pval.SM	F-statistics
rs11012732	G	A	0.331674	0.0200889	0.00211859	2.50E-21	0.0281329	0.0354145	0.426969	39.86
rs11030016	T	C	0.739706	0.018143	0.00227588	1.60E-15	0.0742217	0.0381328	0.0516072	24.47
rs11045163	G	A	0.430597	0.0114512	0.00201589	1.30E-08	0.0253497	0.0337036	0.451968	15.82
rs11075252	G	A	0.284481	-0.012562	0.00221467	1.40E-08	0.0308406	0.0370716	0.405453	13.10
rs1108548	G	A	0.27725	0.0156522	0.00222734	2.10E-12	0.0235374	0.0373586	0.528669	19.79
rs11107114	A	G	0.228885	0.0133238	0.00237525	2.00E-08	-0.017911	0.0397536	0.652313	11.11
rs11113445	G	A	0.391358	0.0127502	0.00203858	4.00E-10	-0.0177764	0.0341034	0.602191	18.64
rs11150461	G	C	0.727351	-0.0141564	0.00224714	3.00E-10	-0.0452069	0.0375291	0.228364	15.74
rs11150745	G	A	0.317702	-0.0202757	0.00214598	3.40E-21	0.0250219	0.0358459	0.485151	38.70
rs11164630	T	C	0.608331	-0.0138102	0.00204155	1.30E-11	0.0371737	0.034285	0.278251	21.81
rs11165643	T	C	0.590085	0.0185255	0.00202023	4.70E-20	0.0591106	0.0339213	0.0814067	40.68
rs11173522	A	C	0.214329	0.013694	0.00242935	1.70E-08	0.0578315	0.0405927	0.154251	10.70
rs1123295	G	A	0.444369	0.0119544	0.00200534	2.50E-09	-0.03743	0.0336505	0.266001	17.55
rs11263719	T	C	0.469657	0.0114989	0.00200822	1.00E-08	-0.0645783	0.0335518	0.0542625	16.33
rs112875651	A	G	0.391041	0.0182659	0.00206839	1.00E-18	-0.0478107	0.0346307	0.167406	37.14
rs113230003	A	G	0.260523	-0.014859	0.00229209	9.00E-11	-0.0808136	0.0383057	0.0348843	16.19
rs11513729	T	C	0.413028	-0.0166494	0.00205054	4.70E-16	-0.0615368	0.0344034	0.0736648	31.97
rs11664106	T	A	0.37403	0.016775	0.00211301	2.00E-15	-0.00284205	0.0352959	0.935823	29.51
rs11704728	T	C	0.196356	0.0148323	0.00252373	4.20E-09	0.0607684	0.0422053	0.149916	10.90
rs11751684	T	G	0.275389	0.0167319	0.0022233	5.20E-14	0.0302924	0.0373734	0.417633	22.60
rs11778934	G	C	0.536083	-0.0141306	0.00200504	1.80E-12	0.028525	0.0335573	0.395304	24.71
rs11779446	G	A	0.161259	-0.020178	0.00271964	1.20E-13	0.0630997	0.045444	0.16498	14.89
rs1182199	A	C	0.304395	-0.0246541	0.00216674	5.40E-30	0.0440869	0.0362729	0.224205	54.83
rs11882796	T	A	0.541285	-0.0113842	0.00200309	1.30E-08	-0.00561714	0.033599	0.867227	16.04

SNP	effect_allele HC	other_alleleHC	eaf.HC	beta.HC	se.HC	pval.HC	beta.SM	se.SM	pval.SM	F-statistics
rs11997077	G	A	0.380458	-0.0112873	0.00206597	4.70E-08	-0.0346876	0.0345799	0.315805	14.07
rs12072739	G	A	0.224461	0.0149904	0.00238686	3.40E-10	0.088556	0.0400173	0.0269017	13.73
rs12122361	G	A	0.265617	-0.013773	0.00226034	1.10E-09	0.0539381	0.0379918	0.155685	14.49
rs12128526	A	G	0.457416	0.0113684	0.00199597	1.20E-08	-0.0360213	0.033553	0.283018	16.10
rs12364470	G	T	0.164552	0.0178805	0.0026871	2.80E-11	0.00355296	0.044934	0.936977	12.17
rs12375196	A	C	0.424347	0.0167592	0.00202832	1.40E-16	0.0464441	0.0339235	0.170973	33.36
rs12427047	T	C	0.24273	-0.0163996	0.0023233	1.70E-12	0.00867913	0.038897	0.823434	18.32
rs12441543	A	G	0.2871	0.0152338	0.0022089	5.30E-12	-0.0356596	0.0369033	0.333894	19.47
rs12519997	A	G	0.559278	-0.0139984	0.00200434	2.90E-12	0.0112471	0.0335747	0.737636	24.05
rs12568411	A	G	0.166749	0.0164813	0.00269359	9.40E-10	0.0174595	0.0453175	0.700037	10.40
rs12569355	G	A	0.119536	0.0217444	0.00307709	1.60E-12	-0.0778025	0.0516333	0.131854	10.51
rs1260326	C	T	0.604267	0.0120369	0.00203008	3.00E-09	0.0207757	0.0341431	0.542863	16.81
rs12607512	G	A	0.44632	0.0117842	0.00200813	4.40E-09	-0.00280452	0.0336283	0.933535	17.02
rs12631813	G	C	0.50262	-0.0122712	0.00200027	8.50E-10	0.0572758	0.0335935	0.0882003	18.82
rs12762744	T	C	0.248261	0.0133166	0.00231189	8.40E-09	-0.0730919	0.0386584	0.0586624	12.38
rs12831185	G	A	0.170442	-0.0176382	0.00265532	3.10E-11	0.0266448	0.0444675	0.549041	12.48
rs1285992	G	A	0.710447	0.0203155	0.00220302	2.90E-20	-0.007766	0.0368211	0.832956	34.99
rs12880641	G	T	0.661578	-0.0149161	0.00210563	1.40E-12	-0.0462473	0.0351293	0.188011	22.47
rs12883788	T	C	0.459801	0.0143864	0.00200867	7.90E-13	0.0465636	0.0335496	0.165167	25.48
rs12921916	C	T	0.28756	0.0125955	0.00222773	1.60E-08	-0.0193883	0.0372143	0.602375	13.10
rs12939848	T	C	0.401034	0.0126399	0.00203785	5.60E-10	0.0539292	0.0341113	0.113883	18.48
rs1294438	T	C	0.354583	0.0186269	0.00210751	9.70E-19	0.323164	0.264973	0.222613	35.76
rs1294438	T	C	0.354583	0.0186269	0.00210751	9.70E-19	0.0138008	0.0353249	0.696032	35.76
rs1296328	C	A	0.559033	-0.015815	0.00201508	4.20E-15	0.0228682	0.0337378	0.497885	30.37

SNP	effect_allele HC	other_alleleHC	eaf.HC	beta.HC	se.HC	pval.HC	beta.SM	se.SM	pval.SM	F-statistics
rs13017207	A	G	0.393634	-0.0161548	0.00203426	2.00E-15	0.0393256	0.0340992	0.248799	30.11
rs13034765	C	G	0.370011	0.0133745	0.00206373	9.10E-11	0.0228319	0.0346382	0.509797	19.58
rs13107325	T	C	0.074895	0.0386647	0.0037857	1.70E-24	0.073001	0.0634155	0.249669	14.46
rs13148263	A	G	0.335162	0.0159436	0.00210506	3.60E-14	-0.05749	0.0352715	0.103117	25.57
rs13156484	A	G	0.472171	-0.0174918	0.00201454	3.90E-18	-0.0332929	0.033739	0.323752	37.58
rs13284988	C	T	0.305411	0.0120763	0.00217861	3.00E-08	0.068665	0.03647	0.0597297	13.04
rs13292699	C	A	0.433708	-0.0162732	0.00201482	6.70E-16	0.0486759	0.0337308	0.149	32.05
rs133015	G	C	0.439948	0.0129624	0.00201566	1.30E-10	-0.00950196	0.0336424	0.777607	20.38
rs13333747	C	T	0.182674	-0.0219722	0.00259208	2.30E-17	-0.0531535	0.043398	0.220654	21.46
rs13374459	C	T	0.411989	0.0126748	0.00202274	3.70E-10	0.0284722	0.0339264	0.401338	19.02
rs13389219	T	C	0.392395	0.0227595	0.00203433	4.70E-29	-0.0638539	0.0341754	0.0617035	59.69
rs143384	G	A	0.404382	0.0267503	0.00203131	1.30E-39	0.0286556	0.034013	0.399514	83.55
rs1446585	G	A	0.244506	-0.0128503	0.00226761	1.50E-08	0.011658	0.0398526	0.769883	11.86
rs1452082	A	C	0.557071	0.012586	0.00203982	6.80E-10	0.0032594	0.0342159	0.924108	18.79
rs1458156	T	C	0.488404	0.0126488	0.00199454	2.30E-10	-0.0180282	0.0333744	0.589071	20.10
rs1477290	C	T	0.136945	0.0298633	0.00292146	1.60E-24	0.0711121	0.0489282	0.146114	24.70
rs147730268	T	G	0.087233	-0.0506092	0.00361111	1.30E-44	-0.0172446	0.060468	0.775502	31.28
rs1480474	G	A	0.417444	-0.0145096	0.00202337	7.40E-13	0.0361382	0.0339588	0.287248	25.01
rs1481630	C	T	0.176871	-0.0153923	0.00261541	4.00E-09	-0.0776059	0.0438593	0.0768228	10.09
rs1502317	T	C	0.27657	-0.0151583	0.00223176	1.10E-11	0.0491089	0.0373947	0.189095	18.46
rs1514895	G	A	0.285289	0.0174697	0.00220175	2.10E-15	0.015251	0.0368624	0.679072	25.67
rs1538535	T	C	0.27456	0.0124387	0.0022369	2.70E-08	-0.0120427	0.0375128	0.748188	12.32
rs1573736	T	G	0.345277	-0.0127955	0.00211185	1.40E-09	-0.106951	0.0353459	0.00247948	16.60
rs1576655	C	A	0.59598	0.0161325	0.00207546	7.70E-15	0.007483	0.0347026	0.829274	29.10

SNP	effect_allele HC	other_alleleHC	eaf.HC	beta.HC	se.HC	pval.HC	beta.SM	se.SM	pval.SM	F-statistics
rs1609783	A	G	0.525344	0.0142717	0.0020044	1.10E-12	-0.00116479	0.0335936	0.972341	25.28
rs16868443	C	G	0.360418	0.0154349	0.0020756	1.00E-13	0.0315737	0.0347637	0.363753	25.50
rs170016	A	G	0.633948	-0.0116498	0.00209333	2.60E-08	-0.0431807	0.0350181	0.217539	14.37
rs17361789	G	T	0.32182	0.0126424	0.00214788	4.00E-09	-0.0292607	0.0360548	0.417042	15.12
rs17639546	A	G	0.14849	-0.0187703	0.0028004	2.00E-11	-0.00718632	0.0469538	0.878358	11.36
rs17733217	G	A	0.229127	-0.0158057	0.00237935	3.10E-11	0.0248053	0.0397177	0.532274	15.59
rs17766836	T	C	0.267888	0.0232323	0.00225241	6.10E-25	-5.60E-04	0.0376807	0.988151	41.73
rs17770336	T	C	0.322443	0.0191461	0.00212912	2.40E-19	-0.0101695	0.0356766	0.775609	35.34
rs1813212	G	A	0.445576	-0.0130082	0.00200772	9.20E-11	0.0472403	0.0336392	0.160222	20.74
rs1868069	A	G	0.229248	0.0150949	0.00236942	1.90E-10	0.00114985	0.0397427	0.976918	14.34
rs1934394	C	G	0.228157	0.0129993	0.00238257	4.90E-08	0.020278	0.039987	0.612076	10.48
rs1955695	G	A	0.62227	-0.0171244	0.00205855	8.90E-17	-0.00223819	0.0344747	0.948236	32.53
rs2022050	A	G	0.160924	-0.0208767	0.0027204	1.70E-14	0.00057966	0.0456185	0.989862	15.90
rs2023211	C	T	0.232254	0.0160386	0.00236158	1.10E-11	0.0434224	0.039576	0.272558	16.45
rs211434	A	G	0.681996	0.0131339	0.00214133	8.60E-10	-0.0166287	0.0358916	0.643147	16.32
rs2133292	T	C	0.373582	0.0132297	0.00206557	1.50E-10	-0.0523399	0.0345856	0.130193	19.20
rs2133561	T	A	0.611067	-0.0181895	0.00206386	1.20E-18	-0.0524033	0.0345815	0.129681	36.92
rs2151248	C	G	0.730659	-0.0149326	0.00224027	2.60E-11	0.0122549	0.0376284	0.744665	17.49
rs2159437	G	A	0.517573	0.0196241	0.00199751	8.90E-23	-0.0216019	0.0334089	0.517897	48.20
rs2172131	C	T	0.578698	-0.0136895	0.00202029	1.20E-11	0.00188983	0.0338009	0.955413	22.39
rs2178385	T	G	0.320337	0.0185474	0.00213295	3.40E-18	-0.0645834	0.0358403	0.0715484	32.93
rs2238435	G	C	0.613709	0.0259551	0.00205177	1.10E-36	-0.0120002	0.0343417	0.726763	75.89
rs2238689	C	T	0.399438	-0.0141655	0.00203741	3.60E-12	-0.0439205	0.0341412	0.19829	23.19
rs2253310	G	C	0.626098	0.019536	0.00205623	2.10E-21	0.0196752	0.0345224	0.568727	42.27

SNP	effect_allele HC	other_alleleHC	eaf.HC	beta.HC	se.HC	pval.HC	beta.SM	se.SM	pval.SM	F-statistics
rs2270894	G	C	0.203219	-0.0216431	0.00256793	3.50E-17	-0.00904152	0.0429251	0.833172	23.01
rs2288745	T	C	0.299451	0.0136628	0.0021768	3.50E-10	-0.0176398	0.0365374	0.629245	16.53
rs2307111	C	T	0.394977	-0.0285352	0.00203828	1.60E-44	-0.007442	0.0341518	0.8275	93.69
rs2371911	A	T	0.544326	0.0131057	0.0019989	5.50E-11	0.0793095	0.0334973	0.0179019	21.33
rs2384054	C	T	0.489428	0.0219057	0.0019869	2.90E-28	-0.0771278	0.0333928	0.020904	60.76
rs2479958	G	A	0.516982	-0.0129914	0.00201927	1.20E-10	-0.0120085	0.0337753	0.722184	20.67
rs2494196	A	C	0.286135	0.0312737	0.00219937	6.90E-46	-0.0449816	0.0369525	0.223497	82.61
rs2499468	A	C	0.651137	0.0131826	0.00209013	2.80E-10	-0.00670925	0.0350471	0.848185	18.07
rs253444	A	G	0.12923	-0.0198245	0.00297188	2.50E-11	-0.0623228	0.0499802	0.212416	10.01
rs2568958	A	G	0.603693	0.0206705	0.00202892	2.20E-24	0.00136011	0.0341105	0.968194	49.67
rs2577955	T	C	0.802767	-0.014667	0.00250536	4.80E-09	0.0401966	0.0417416	0.335554	10.85
rs25849	G	C	0.288829	0.019767	0.00220822	3.50E-19	-0.00296548	0.0369033	0.935952	32.92
rs2585526	G	A	0.557413	0.0122667	0.00201131	1.10E-09	0.0286433	0.0336327	0.394408	18.35
rs2593169	C	G	0.567965	-0.0116309	0.00201784	8.20E-09	0.0189119	0.0337461	0.575195	16.31
rs2606227	C	T	0.63145	-0.0124349	0.00208271	2.40E-09	0.0452389	0.0349246	0.195206	16.59
rs2642305	T	A	0.369566	0.0113043	0.00206693	4.50E-08	0.065695	0.0345714	0.0573971	13.94
rs2660241	C	T	0.364912	0.0139138	0.00207299	1.90E-11	0.00787858	0.0347691	0.820737	20.88
rs2678204	G	T	0.340172	0.0196748	0.00209913	7.10E-21	0.0111338	0.0352623	0.752199	39.44
rs273505	C	T	0.422021	0.0128735	0.00202114	1.90E-10	0.0130258	0.0337968	0.699931	19.79
rs2737263	T	G	0.28052	-0.0236589	0.00222017	1.60E-26	-0.104121	0.0371371	0.00505208	45.84
rs2744956	C	T	0.139254	0.0537577	0.00287012	2.80E-78	0.0423189	0.0481111	0.379072	84.11
rs2814350	A	G	0.304993	0.0138717	0.00220875	3.40E-10	-0.0525698	0.0368736	0.153963	16.72
rs2821226	G	A	0.527763	0.015138	0.00200431	4.30E-14	0.0493864	0.0336548	0.142256	28.44
rs28366156	C	T	0.130582	-0.0265761	0.00295158	2.20E-19	-0.0489375	0.0495786	0.323609	18.41

SNP	effect_allele HC	other_alleleHC	eaf.HC	beta.HC	se.HC	pval.HC	beta.SM	se.SM	pval.SM	F-statistics
rs28457680	A	T	0.136611	0.0195227	0.00291985	2.30E-11	0.0793335	0.0488347	0.104262	10.55
rs2861685	C	T	0.411983	-0.0147772	0.0020154	2.30E-13	0.0114506	0.0338335	0.735032	26.05
rs28711392	C	T	0.367255	-0.0125224	0.00208379	1.90E-09	-0.0500652	0.0349233	0.151694	16.78
rs28778940	A	G	0.330838	0.0151514	0.00212367	9.70E-13	0.00424121	0.0355035	0.904912	22.54
rs287837	G	A	0.523844	-0.013078	0.00200242	6.50E-11	-0.00607592	0.0335692	0.85637	21.28
rs28971796	G	A	0.620409	-0.0151466	0.00227581	2.80E-11	-0.00670867	0.0382384	0.860731	20.86
rs3012053	G	A	0.714878	-0.0149707	0.00220858	1.20E-11	0.0223286	0.0369449	0.545595	18.73
rs308911	G	A	0.714423	-0.0142761	0.00220578	9.70E-11	0.0210466	0.0369702	0.569163	17.09
rs310796	T	G	0.680961	0.0121086	0.00214394	1.60E-08	0.00921629	0.0358905	0.797341	13.86
rs3110942	A	G	0.481664	0.0127123	0.00199815	2.00E-10	0.0659103	0.0334365	0.0487001	20.21
rs3116600	G	A	0.215319	-0.0187688	0.00242539	1.00E-14	0.0311975	0.0405249	0.441397	20.24
rs314288	C	T	0.886256	-0.0227112	0.00313819	4.60E-13	0.081758	0.0526647	0.120561	10.56
rs329118	T	C	0.419386	-0.0163479	0.00201987	5.80E-16	-0.0275107	0.0338529	0.416417	31.90
rs33503	A	G	0.80589	-0.017501	0.00251417	3.40E-12	-0.0310801	0.0421606	0.461011	15.16
rs340025	C	T	0.57994	0.0127406	0.00202809	3.30E-10	0.000483694	0.0338572	0.988602	19.23
rs34223321	A	C	0.300092	0.0140924	0.00217285	8.80E-11	0.0435244	0.0363161	0.230727	17.67
rs34517439	A	C	0.121795	0.0454548	0.00307552	2.00E-49	0.073349	0.0516852	0.155856	46.73
rs34594435	T	C	0.195585	0.0215785	0.00251186	8.60E-18	-0.00601835	0.042074	0.886257	23.22
rs34629844	G	A	0.12828	0.0212952	0.00297607	8.30E-13	-0.0131317	0.0497406	0.791777	11.45
rs34656389	G	A	0.367473	0.011297	0.00206647	4.60E-08	0.00987535	0.0346989	0.77595	13.89
rs34765854	G	T	0.298258	0.0193318	0.00217163	5.50E-19	0.0402392	0.0363458	0.268242	33.17
rs34769775	T	C	0.29735	-0.0141127	0.00218344	1.00E-10	-0.0396885	0.0364939	0.276801	17.46
rs34772064	G	T	0.555918	-0.0118736	0.00200309	3.10E-09	0.0241004	0.0335852	0.473011	17.35
rs34811474	A	G	0.230735	-0.0197929	0.00236244	5.40E-17	-3.53E-04	0.0395603	0.992884	24.92

SNP	effect_allele HC	other_alleleHC	eaf.HC	beta.HC	se.HC	pval.HC	beta.SM	se.SM	pval.SM	F-statistics
rs34840745	T	C	0.262486	0.0166433	0.00227207	2.40E-13	-0.0259621	0.0379165	0.493523	20.78
rs34848742	G	T	0.788211	-0.0143597	0.00243566	3.70E-09	-0.00588591	0.0407858	0.885254	11.60
rs35506085	A	G	0.185085	-0.0204796	0.00258575	2.40E-15	-0.0574239	0.0432696	0.18447	18.92
rs35537311	T	C	0.388607	-0.0147642	0.00205013	6.00E-13	0.0313501	0.0342508	0.360029	24.65
rs35792595	A	T	0.297453	0.0137734	0.00219158	3.30E-10	-0.012805	0.0366379	0.726713	16.51
rs35882248	T	C	0.317213	0.015668	0.00213778	2.30E-13	0.0526144	0.0358573	0.142287	23.27
rs35910339	G	C	0.683708	-0.0127042	0.00214217	3.00E-09	-0.0343064	0.0358396	0.338456	15.21
rs35917007	G	A	0.533673	0.0147568	0.00199857	1.50E-13	0.0193236	0.0333955	0.562839	27.14
rs36140	C	A	0.635313	0.0148434	0.00207958	9.50E-13	0.0324323	0.0349337	0.353201	23.61
rs365352	A	G	0.24439	-0.0205385	0.0023126	6.60E-19	-0.0199389	0.0386922	0.606329	29.13
rs3737992	A	G	0.168963	-0.0215132	0.00265121	4.90E-16	0.0930934	0.0444388	0.0361826	18.49
rs3762988	T	C	0.388074	0.0126504	0.0020481	6.50E-10	0.00642393	0.0343316	0.851571	18.12
rs3807566	T	G	0.438301	-0.0149491	0.00201199	1.10E-13	0.0262914	0.0335797	0.433654	27.18
rs3810291	A	G	0.674991	0.0227348	0.00212876	1.30E-26	-0.0112247	0.0356062	0.752575	50.05
rs3811951	G	A	0.282058	0.0150182	0.00221148	1.10E-11	-0.0322266	0.0370477	0.384372	18.68
rs3814883	T	C	0.482403	0.0250685	0.00200044	5.00E-36	-0.0133553	0.0335021	0.690159	78.43
rs3826408	T	C	0.456783	0.0135888	0.00200083	1.10E-11	-0.0181588	0.0334584	0.587316	22.89
rs3845344	T	C	0.391164	0.0138703	0.00203631	9.70E-12	0.0427948	0.0340999	0.209485	22.10
rs3935190	A	G	0.536778	-0.0141131	0.00201245	2.30E-12	-0.00882247	0.0336142	0.792965	24.46
rs394608	C	T	0.537697	0.014611	0.00201006	3.60E-13	-0.0147296	0.033528	0.660429	26.27
rs40071	C	T	0.179489	-0.0174105	0.00260267	2.20E-11	-0.0229783	0.0435819	0.598025	13.18
rs4017425	T	C	0.470189	-0.0118377	0.00199708	3.10E-09	-0.0315945	0.0334968	0.345573	17.51
rs4240326	G	A	0.550142	-0.0279318	0.0020006	2.70E-44	-0.00919794	0.0335161	0.783751	96.50
rs4253755	A	G	0.128527	0.0211316	0.00300303	2.00E-12	0.0261489	0.0501726	0.602242	11.09

SNP	effect_allele HC	other_alleleHC	eaf.HC	beta.HC	se.HC	pval.HC	beta.SM	se.SM	pval.SM	F-statistics
rs429343	G	A	0.576577	-0.0121964	0.00201501	1.40E-09	-0.0395551	0.0338479	0.24256	17.89
rs4310395	G	A	0.442854	0.0122183	0.00200242	1.00E-09	-0.0417345	0.0335969	0.214157	18.37
rs4467770	A	G	0.731091	0.0158784	0.00225367	1.80E-12	0.0317257	0.0377791	0.401038	19.52
rs4476935	T	C	0.433157	-0.0128732	0.00201066	1.50E-10	-0.0271997	0.0336807	0.419336	20.13
rs4477562	T	C	0.128661	0.0292203	0.00300396	2.30E-22	0.0428385	0.0500546	0.392089	21.22
rs4486868	C	T	0.447992	-0.011105	0.00201104	3.40E-08	0.0439013	0.0336168	0.191576	15.08
rs4567604	T	G	0.193192	-0.0166838	0.00253879	5.00E-11	-0.0732669	0.0425397	0.085012	13.46
rs4589131	G	T	0.590569	-0.0117346	0.00202078	6.40E-09	-0.0539269	0.0339215	0.11189	16.31
rs4613074	C	T	0.18516	-0.0170225	0.00256625	3.30E-11	0.0183748	0.0430725	0.669669	13.28
rs463376	A	G	0.490305	0.0119932	0.0019975	1.90E-09	0.00485463	0.0333504	0.884265	18.02
rs4671328	G	T	0.551345	-0.0163116	0.00201469	5.70E-16	0.00365982	0.0338043	0.913786	32.43
rs4678016	T	C	0.366307	0.0115138	0.00206489	2.50E-08	0.0904116	0.0346994	0.00917234	14.43
rs475390	A	G	0.776432	-0.0140664	0.00238863	3.90E-09	0.0194575	0.0400383	0.626986	12.04
rs4790292	A	C	0.153689	-0.0261893	0.00277723	4.10E-21	-0.0276036	0.0462836	0.550908	23.13
rs4792716	G	A	0.562173	0.0145605	0.00201188	4.60E-13	0.0633194	0.0336054	0.0595374	25.79
rs4800490	C	A	0.495235	-0.023172	0.00199562	3.60E-31	-0.0341073	0.0333742	0.306798	67.42
rs4820346	G	C	0.69295	-0.0130489	0.00217064	1.80E-09	-0.0126348	0.0361669	0.726829	15.38
rs483465	G	A	0.749331	0.0154934	0.00229726	1.50E-11	0.0143158	0.038518	0.710142	17.09
rs4843158	C	G	0.68516	0.0218462	0.00214891	2.80E-24	0.00169709	0.0359219	0.962319	44.59
rs4870057	G	A	0.341223	0.0126926	0.00212695	2.40E-09	0.0137127	0.0356202	0.700261	16.01
rs4909309	C	T	0.393916	-0.0173539	0.00203923	1.70E-17	-0.0105143	0.0341461	0.758143	34.58
rs4962424	A	T	0.326831	0.0155846	0.00212749	2.40E-13	0.0201271	0.0355767	0.571571	23.61
rs4963975	A	G	0.244315	-0.0187917	0.00232365	6.10E-16	-0.0232178	0.038889	0.550488	24.15
rs4970946	G	A	0.217454	0.0141191	0.00240909	4.60E-09	-0.0049409	0.0404917	0.902881	11.69

SNP	effect_allele HC	other_alleleHC	eaf.HC	beta.HC	se.HC	pval.HC	beta.SM	se.SM	pval.SM	F-statistics
rs4976994	G	A	0.452549	0.0128387	0.00200503	1.50E-10	0.0141365	0.0335225	0.673243	20.32
rs522110	G	A	0.555042	0.0181191	0.0020069	1.70E-19	-0.0243668	0.033619	0.46858	40.27
rs543874	G	A	0.205218	0.0453641	0.00246182	8.00E-76	0.00368805	0.0412915	0.92883	110.79
rs551935	G	T	0.440778	-0.016925	0.00200492	3.10E-17	0.0183537	0.033579	0.584665	35.13
rs55726687	A	G	0.209736	0.0218167	0.00244465	4.50E-19	-0.0603978	0.040926	0.140003	26.40
rs56094641	G	A	0.404596	0.0616325	0.00203052	1.00E-200	0.0351196	0.0339399	0.300782	444.31
rs56097510	G	C	0.51052	0.0137125	0.00199793	6.70E-12	0.0599729	0.0333645	0.0722553	23.54
rs56288810	G	A	0.213142	0.0136147	0.00244829	2.70E-08	0.0607531	0.0409586	0.137999	10.37
rs56399737	T	C	0.449109	-0.0118202	0.00201255	4.30E-09	-0.0315113	0.0335896	0.348179	17.07
rs57222629	C	G	0.311673	0.0138153	0.0021564	1.50E-10	0.018947	0.0361456	0.600149	17.61
rs57235969	T	C	0.554199	0.0124617	0.00199957	4.60E-10	-0.0470572	0.0335362	0.160563	19.19
rs57636386	C	T	0.08382	-0.0350845	0.0036118	2.60E-22	-0.0747878	0.0601015	0.213368	14.49
rs57989773	C	T	0.245008	0.021874	0.00238057	4.00E-20	0.042091	0.0398831	0.291261	31.24
rs58584712	A	G	0.211251	0.0169765	0.0024357	3.20E-12	-0.00636708	0.0410012	0.876593	16.19
rs58862095	T	C	0.419278	-0.0181051	0.00202425	3.70E-19	0.0817249	0.0338539	0.0157765	38.96
rs60226453	T	C	0.177273	0.0162724	0.00260018	3.90E-10	0.0178599	0.043825	0.68362	11.42
rs6066104	T	C	0.324108	0.0192833	0.00213455	1.70E-19	-0.0280425	0.035742	0.4327	35.76
rs6142059	C	T	0.492562	0.0149966	0.00200021	6.50E-14	-0.0887738	0.0334376	0.00793305	28.10
rs614520	A	G	0.640637	-0.0130274	0.00212026	8.00E-10	0.000367197	0.0354456	0.991734	17.38
rs61941722	A	G	0.188737	0.0155992	0.00256577	1.20E-09	-0.0311207	0.0430116	0.469347	11.32
rs61969510	C	T	0.278956	0.0128445	0.00224311	1.00E-08	0.0322992	0.0375249	0.38938	13.19
rs62070648	A	G	0.268727	-0.0199439	0.0022501	7.80E-19	0.0327393	0.037591	0.383789	30.88
rs62107261	C	T	0.04832	-0.0808476	0.00465016	1.10E-67	-0.0733445	0.0776652	0.344982	27.80

SNP	effect_allele HC	other_alleleHC	eaf.HC	beta.HC	se.HC	pval.HC	beta.SM	se.SM	pval.SM	F-statistics
rs62301134	C	T	0.263286	-0.013638	0.00226745	1.80E-09	-0.0385149	0.0380472	0.311398	14.03
rs62396185	C	G	0.259989	-0.0346069	0.00227012	1.80E-52	-0.0538707	0.0381016	0.1574	89.44
rs62515438	G	T	0.227665	0.0150395	0.00237712	2.50E-10	0.0299356	0.0396707	0.450488	14.08
rs6438656	A	G	0.635867	-0.0119598	0.0020673	7.20E-09	-0.0641052	0.0346485	0.064291	15.50
rs6465828	T	G	0.482837	0.0163908	0.00199173	1.90E-16	-0.0164174	0.0333536	0.622561	33.82
rs6545714	A	G	0.60145	-0.0158267	0.00203362	7.10E-15	-0.0254351	0.0341471	0.456351	29.04
rs6567160	C	T	0.232716	0.0523537	0.00236068	5.70E-109	-0.0383816	0.0393246	0.329055	175.71
rs6575340	A	G	0.636028	0.0184545	0.00207818	6.70E-19	0.0267563	0.0347512	0.441336	36.51
rs6597975	G	C	0.543875	0.0122921	0.00201118	9.80E-10	0.0430125	0.033683	0.20161	18.53
rs6598540	G	A	0.276885	-0.0147876	0.00223088	3.40E-11	-0.0803884	0.0372481	0.0309136	17.60
rs6606686	C	G	0.680627	-0.0165692	0.00213651	8.80E-15	-0.0366462	0.0357642	0.305523	26.15
rs66679256	T	C	0.445805	0.0149901	0.00200438	7.50E-14	-0.0414005	0.0336518	0.218599	27.64
rs6669341	G	A	0.582713	-0.0151291	0.00201544	6.10E-14	-0.0259024	0.0337909	0.44335	27.41
rs667515	C	G	0.386143	-0.0141684	0.00205316	5.20E-12	0.0215473	0.0343392	0.530343	22.58
rs6744646	G	A	0.828323	0.0500537	0.00263558	2.00E-80	0.093848	0.0441896	0.0336907	102.60
rs6747657	A	G	0.281723	0.0129341	0.00220754	4.70E-09	-0.0171856	0.0370131	0.642426	13.89
rs675162	G	A	0.482059	0.0180079	0.00199495	1.80E-19	-0.0281913	0.0334435	0.399255	40.69
rs6821305	C	A	0.399287	0.0148966	0.00203236	2.30E-13	-0.0336	0.0340719	0.32406	25.77
rs6868125	T	C	0.521906	-0.0119493	0.00198836	1.90E-09	-0.00887753	0.0333826	0.790291	18.02
rs6946415	G	A	0.627099	0.0158826	0.00206409	1.40E-14	0.0328809	0.0346111	0.342107	27.69
rs6958365	T	C	0.411809	-0.0115375	0.00203582	1.50E-08	-0.00448398	0.0340599	0.895261	15.56
rs698147	G	A	0.543563	-0.0121448	0.00200109	1.30E-09	-0.00180711	0.0335252	0.957012	18.28
rs7002088	C	A	0.445199	0.0137173	0.00200556	7.90E-12	0.0819996	0.0336001	0.0146686	23.11

SNP	effect_allele HC	other_alleleHC	eaf.HC	beta.HC	se.HC	pval.HC	beta.SM	se.SM	pval.SM	F-statistics
rs7034554	G	A	0.373815	-0.012422	0.0020595	1.60E-09	0.0104707	0.0344211	0.76098	17.03
rs7038943	C	T	0.338791	-0.0121087	0.00210372	8.60E-09	0.0101689	0.035274	0.773131	14.84
rs705165	T	G	0.247646	0.0148091	0.00230698	1.40E-10	-0.0368996	0.0386354	0.339542	15.36
rs7094073	T	C	0.191266	0.0163482	0.00273496	2.30E-09	-0.0694328	0.0456507	0.12827	11.05
rs7111235	C	T	0.493399	0.0115415	0.00200364	8.40E-09	-0.0505289	0.0335592	0.132154	16.59
rs7124681	A	C	0.408387	0.0205032	0.00202231	3.70E-24	-0.0302404	0.0338821	0.372116	49.67
rs7132908	A	G	0.384469	0.0257741	0.00204901	2.80E-36	-0.00805489	0.0343471	0.814586	74.90
rs7171864	A	G	0.660205	0.0161986	0.00211572	1.90E-14	0.0123822	0.0354162	0.726624	26.30
rs7218014	C	T	0.197308	0.0207669	0.00251132	1.30E-16	-0.0520651	0.0421363	0.216594	21.66
rs7248205	T	C	0.600262	-0.0161199	0.00204315	3.00E-15	0.00864754	0.0341854	0.800299	29.87
rs7257083	A	G	0.288605	0.0162612	0.0022051	1.70E-13	-0.039295	0.03683	0.286003	22.33
rs72634826	A	G	0.259879	-0.0153317	0.00229923	2.60E-11	-0.0699561	0.0385192	0.069349	17.11
rs72656010	C	T	0.132181	-0.0252479	0.00295341	1.20E-17	-0.011202	0.0495258	0.821058	16.77
rs7274811	T	G	0.258589	-0.01951	0.00227858	1.10E-17	-0.0351674	0.0381054	0.356061	28.11
rs72756476	C	T	0.141428	-0.0192477	0.00287344	2.10E-11	0.120225	0.047894	0.0120651	10.90
rs72801843	A	T	0.301389	0.0186789	0.00217211	8.00E-18	0.037515	0.036366	0.302262	31.14
rs72959041	A	G	0.049137	-0.0662534	0.00466657	9.50E-46	-0.0610627	0.0782156	0.434981	18.84
rs73052033	C	T	0.184918	-0.0208103	0.00256783	5.30E-16	-0.0181331	0.0430904	0.67389	19.80
rs73175572	G	A	0.111726	0.0282075	0.00319248	1.00E-18	-0.134187	0.0535298	0.012184	15.50
rs73213484	T	A	0.141217	-0.0207006	0.00286036	4.60E-13	-0.00575697	0.0480495	0.904631	12.70
rs7365	G	A	0.482654	-0.0110939	0.00199385	2.60E-08	0.00329584	0.0334289	0.921462	15.46
rs7372674	A	C	0.357244	0.014356	0.00207575	4.60E-12	0.00419379	0.0347755	0.904011	21.97
rs743572	G	A	0.376473	0.0151185	0.00205743	2.00E-13	-0.00360873	0.0344662	0.916611	25.35
rs7442885	G	C	0.214027	-0.0198682	0.00243204	3.10E-16	-0.0411031	0.0409055	0.314979	22.45

SNP	effect_allele HC	other_alleleHC	eaf.HC	beta.HC	se.HC	pval.HC	beta.SM	se.SM	pval.SM	F-statistics
rs7460093	A	G	0.53118	0.0136639	0.0020144	1.20E-11	-0.0104707	0.0337105	0.7561	22.92
rs74749286	A	G	0.107637	0.0258963	0.00323591	1.20E-15	-0.00431084	0.054298	0.936721	12.30
rs7498665	G	A	0.399666	0.0317857	0.00203647	6.40E-55	0.0241258	0.0341061	0.479334	116.93
rs750090	C	T	0.35675	-0.0132955	0.00210095	2.50E-10	-0.00773369	0.0351398	0.825807	18.38
rs7519259	A	G	0.528388	0.0141441	0.00200058	1.50E-12	0.0799664	0.0335995	0.0173133	24.91
rs7523668	A	G	0.565827	-0.0137199	0.00200928	8.60E-12	-0.00484393	0.0337166	0.885764	22.91
rs756717	A	G	0.399077	-0.0140302	0.00206015	9.70E-12	0.0181077	0.0344458	0.599105	22.25
rs7570446	A	C	0.544459	0.0110158	0.00199439	3.30E-08	0.0106878	0.0335095	0.749766	15.13
rs7571496	G	A	0.260562	-0.0138156	0.00227445	1.20E-09	-0.0471952	0.0381818	0.216435	14.22
rs7582359	A	G	0.331712	-0.0135492	0.00212104	1.70E-10	0.0143504	0.0355292	0.686283	18.09
rs7632381	C	T	0.444325	0.0270429	0.00200044	1.20E-41	0.0081021	0.0335574	0.809214	90.26
rs765874	A	T	0.489353	-0.0168012	0.0019903	3.10E-17	0.0222324	0.0333399	0.504874	35.62
rs76647086	T	G	0.176857	-0.0202229	0.00260327	8.00E-15	0.0112921	0.0437395	0.796278	17.57
rs76798800	T	G	0.266025	0.0244232	0.00225319	2.20E-27	0.0454009	0.0378606	0.230465	45.89
rs7695177	G	C	0.470976	-0.0151527	0.00199491	3.10E-14	-0.0425493	0.0334166	0.202913	28.75
rs7696175	C	T	0.526478	0.0136068	0.00200383	1.10E-11	-0.0750407	0.0338	0.0264095	22.99
rs7707394	A	G	0.357269	-0.0184703	0.002075	5.50E-19	0.00675688	0.0347917	0.846012	36.39
rs7708584	G	A	0.572378	-0.013196	0.00201087	5.30E-11	-0.0319617	0.033636	0.341998	21.08
rs7740107	A	T	0.736289	-0.0236486	0.00225676	1.10E-25	-0.0374859	0.0378298	0.321731	42.65
rs7793674	C	A	0.144347	0.019239	0.00284373	1.30E-11	0.084266	0.0474468	0.0757304	11.31
rs779655	C	G	0.729184	0.0143261	0.00224052	1.60E-10	0.0122237	0.0375663	0.744886	16.15
rs7805441	T	C	0.502255	0.0126844	0.00200504	2.50E-10	0.0177525	0.0335753	0.596987	20.01
rs7864465	A	G	0.559379	-0.0110794	0.00200561	3.30E-08	0.012171	0.0335347	0.716653	15.04
rs7893571	T	G	0.665871	0.0138381	0.00211863	6.50E-11	0.0919474	0.0354019	0.00939745	18.98

SNP	effect_allele HC	other_alleleHC	eaf.HC	beta.HC	se.HC	pval.HC	beta.SM	se.SM	pval.SM	F-statistics
rs7903146	T	C	0.290662	-0.0214133	0.00219381	1.70E-22	-0.025742	0.0367052	0.483104	39.29
rs7944782	G	T	0.50981	0.0129576	0.0020033	9.90E-11	0.0637568	0.0335427	0.0573337	20.91
rs7952436	T	C	0.081996	-0.0354571	0.00363182	1.60E-22	0.0376648	0.0608917	0.53621	14.35
rs7996639	A	G	0.449371	0.0137138	0.00201868	1.10E-11	-0.0687406	0.0337725	0.04181	22.84
rs8030456	T	C	0.226311	-0.0247721	0.00237863	2.10E-25	-0.0987433	0.0398266	0.0131629	37.98
rs8132129	T	C	0.184901	-0.015922	0.00259474	8.40E-10	-0.0561456	0.0433425	0.195185	11.35
rs8133137	G	A	0.664097	0.0157777	0.00211541	8.80E-14	-0.00533721	0.0353258	0.879908	24.82
rs815163	C	T	0.563175	-0.0168978	0.00200258	3.20E-17	-0.00529718	0.0336466	0.874902	35.03
rs852042	G	A	0.758582	-0.0153695	0.00233046	4.30E-11	-0.0284438	0.0389941	0.465734	15.93
rs852983	A	G	0.459576	-0.0118657	0.00199745	2.80E-09	-0.00379523	0.0334158	0.909574	17.53
rs882378	C	A	0.306629	0.0152549	0.00216702	1.90E-12	-0.0374101	0.0362722	0.302367	21.07
rs897186	G	A	0.548592	-0.0160621	0.00199856	9.20E-16	-0.0253401	0.0334841	0.449182	31.99
rs9284814	A	G	0.884815	0.0230203	0.00311527	1.50E-13	0.0506205	0.0525538	0.335441	11.13
rs9378684	T	C	0.200589	0.018067	0.00250737	5.80E-13	0.0297651	0.0420215	0.478741	16.65
rs9385385	T	C	0.447856	0.012816	0.00201537	2.00E-10	0.0191813	0.0337232	0.569502	20.00
rs9512696	G	A	0.66154	0.0172114	0.00211114	3.60E-16	-0.0191036	0.0353463	0.588875	29.77
rs9513018	T	G	0.616147	-0.0112355	0.00205443	4.50E-08	-0.0283585	0.034362	0.409208	14.15
rs968379	T	C	0.229098	-0.0187833	0.00237244	2.40E-15	0.00764314	0.039765	0.84758	22.14
rs9764678	C	T	0.272455	0.0161285	0.00225012	7.60E-13	-0.0244116	0.0377255	0.517577	20.37
rs9788550	C	G	0.247466	-0.0214137	0.00232004	2.70E-20	-0.0465052	0.0388066	0.230768	31.73
rs9830592	A	C	0.582425	0.0138364	0.00201841	7.10E-12	0.0303345	0.0338121	0.369639	22.86
rs9843653	C	T	0.511657	0.0183267	0.00199203	3.60E-20	-0.0270878	0.0334392	0.417904	42.30
rs9845755	T	A	0.198555	0.027361	0.0024977	6.30E-28	-0.0185354	0.0419795	0.658826	38.19
rs9850529	A	G	0.339796	0.0114818	0.00209898	4.50E-08	-0.0543306	0.0352531	0.123278	13.43

SNP	effect_allele HC	other_alleleHC	eaf.HC	beta.HC	se.HC	pval.HC	beta.SM	se.SM	pval.SM	F-statistics
rs987237	G	A	0.17957	0.0344542	0.00259141	2.50E-40	-0.0974534	0.0434919	0.0250438	52.09
rs9876664	T	G	0.3754	-0.0143788	0.00205893	2.90E-12	0.00426609	0.0345426	0.901709	22.87
rs9951619	G	T	0.767352	0.0138525	0.00237634	5.60E-09	0.0106481	0.0397322	0.788702	12.13
rs9967287	T	G	0.251722	0.0141454	0.00230082	7.80E-10	0.0123967	0.0385461	0.747749	14.24
rs9967367	T	C	0.293494	-0.0174208	0.00220445	2.70E-15	-0.047937	0.0367551	0.192156	25.90
rs998584	A	C	0.482802	-0.0222736	0.00199467	5.90E-29	-0.0120676	0.0334845	0.718552	62.28

Abbreviations: SNP, single nucleotide polymorphism; se, standard error; HC, hip circumference; SM, sepsis mortality; pval, p-value.

Table S11. Detailed information about single-nucleotide polymorphisms of hip circumference on cholecystitis.

SNP	effect_allele HC	other_alleleHC	eaf. HC	beta.HC	se.HC	pval.HC	beta. cholecystitis	se. cholecystitis	pval. cholecystitis	F-statistics
rs1006399	A	G	0.458873	-0.0114857	0.00200117	9.50E-09	0.0154	0.0119	0.1957	16.36
rs10100245	A	G	0.564506	0.0205386	0.0020121	1.80E-24	-0.0154	0.0118	0.1924	51.24
rs10118701	G	A	0.32209	0.0168068	0.00213097	3.10E-15	0.0021	0.012	0.8635	27.17
rs10132514	T	C	0.273182	-0.0131415	0.00225463	5.60E-09	-0.0196	0.0143	0.17	13.49
rs1013402	G	A	0.318427	0.0293703	0.002136	5.10E-43	0.03	0.0124	0.0155099	82.08
rs10145154	T	C	0.22178	0.0253835	0.00240401	4.60E-26	0.0163	0.0137	0.2346	38.49
rs10153248	G	A	0.447835	-0.0158858	0.00200883	2.60E-15	-0.0085	0.0121	0.4806	30.93
rs10172196	A	G	0.305442	0.0174435	0.00216332	7.40E-16	0.006	0.0125	0.630099	27.59
rs10210468	C	T	0.464304	-0.0137575	0.00201939	9.60E-12	-0.0083	0.0118	0.4792	23.09
rs1023767	A	G	0.235192	-0.015354	0.00234795	6.20E-11	-0.0177	0.0121	0.1448	15.38
rs1037702	A	G	0.62178	-0.0116182	0.00205972	1.70E-08	-0.0041	0.0121	0.732799	14.97
rs10404726	T	C	0.465501	-0.0166459	0.00200329	9.60E-17	-0.0228	0.0118	0.0522504	34.36
rs10407871	C	T	0.155426	-0.0181966	0.00276505	4.70E-11	0.0142	0.0155	0.3611	11.37
rs10471636	A	G	0.508884	-0.0118317	0.00203156	5.70E-09	0.0038	0.0118	0.7471	16.95
rs1056441	C	T	0.675428	0.014638	0.002132	6.60E-12	-0.0191	0.0134	0.1549	20.67
rs1056720	T	C	0.234833	-0.0130647	0.00235122	2.80E-08	0.0081	0.0147	0.5811	11.10
rs10744145	A	C	0.513795	-0.0121183	0.00199668	1.30E-09	-0.0195	0.0118	0.0962299	18.40
rs10746833	G	A	0.581617	-0.0149421	0.0020362	2.20E-13	0.0251	0.0118	0.0333703	26.21
rs10773051	T	C	0.222167	0.0231417	0.00240285	5.90E-22	0.0079	0.0129	0.542199	32.06
rs10810598	A	T	0.639535	-0.0129254	0.00207775	4.90E-10	-0.0267	0.0123	0.0298703	17.84
rs10883553	A	C	0.445855	0.0151046	0.00200686	5.20E-14	6.00E-04	0.0119	0.9605	27.99
rs10938397	G	A	0.434366	0.02439	0.00201147	7.70E-34	0.0283	0.0118	0.0161998	72.26
rs10954284	A	T	0.488124	0.0193137	0.00199245	3.20E-22	-0.0034	0.0118	0.771999	46.96

SNP	effect_allele HC	other_alleleHC	eaf. HC	beta.HC	se.HC	pval.HC	beta. cholecystitis	se. cholecystitis	pval. cholecystitis	F-statistics
rs10987417	T	G	0.385638	0.0128078	0.00206992	6.10E-10	-5.00E-04	0.0118	0.9676	18.14
rs11012732	G	A	0.331674	0.0200889	0.00211859	2.50E-21	0.0243	0.0126	0.0552001	39.86
rs11030016	T	C	0.739706	0.018143	0.00227588	1.60E-15	-0.0105	0.0125	0.4046	24.47
rs11045163	G	A	0.430597	0.0114512	0.00201589	1.30E-08	0.0015	0.012	0.9039	15.82
rs11075252	G	A	0.284481	-0.012562	0.00221467	1.40E-08	-0.0139	0.0125	0.2665	13.10
rs1108548	G	A	0.27725	0.0156522	0.00222734	2.10E-12	0.0057	0.0127	0.652299	19.79
rs11107114	A	G	0.228885	0.0133238	0.00237525	2.00E-08	0.0022	0.0125	0.8583	11.11
rs11113445	G	A	0.391358	0.0127502	0.00203858	4.00E-10	0.0133	0.0121	0.2733	18.64
rs11150461	G	C	0.727351	-0.0141564	0.00224714	3.00E-10	0.0246	0.0144	0.0878699	15.74
rs11150745	G	A	0.317702	-0.0202757	0.00214598	3.40E-21	-0.0226	0.0131	0.0851001	38.70
rs11164630	T	C	0.608331	-0.0138102	0.00204155	1.30E-11	-0.0139	0.0123	0.2571	21.81
rs11165643	T	C	0.590085	0.0185255	0.00202023	4.70E-20	0.0103	0.012	0.3889	40.68
rs11173522	A	C	0.214329	0.013694	0.00242935	1.70E-08	-0.0114	0.0149	0.443	10.70
rs1123295	G	A	0.444369	0.0119544	0.00200534	2.50E-09	0.0221	0.0118	0.0618002	17.55
rs11263719	T	C	0.469657	0.0114989	0.00200822	1.00E-08	0.0116	0.012	0.3313	16.33
rs113230003	A	G	0.260523	-0.014859	0.00229209	9.00E-11	-0.0187	0.0144	0.1944	16.19
rs11513729	T	C	0.413028	-0.0166494	0.00205054	4.70E-16	0.0143	0.0121	0.2355	31.97
rs11664106	T	A	0.37403	0.016775	0.00211301	2.00E-15	-0.0086	0.0127	0.4962	29.51
rs11704728	T	C	0.196356	0.0148323	0.00252373	4.20E-09	0.0387	0.0143	0.00693107	10.90
rs11751684	T	G	0.275389	0.0167319	0.0022233	5.20E-14	0.009	0.0152	0.554201	22.60
rs11762444	A	G	0.216137	0.014599	0.00241918	1.60E-09	0.0119	0.0147	0.4211	12.34
rs11778934	G	C	0.536083	-0.0141306	0.00200504	1.80E-12	0.0211	0.0123	0.08569	24.71
rs11779446	G	A	0.161259	-0.020178	0.00271964	1.20E-13	0.0116	0.0168	0.4909	14.89
rs1182199	A	C	0.304395	-0.0246541	0.00216674	5.40E-30	-0.0173	0.0122	0.1571	54.83

SNP	effect_allele HC	other_alleleHC	eaf. HC	beta.HC	se.HC	pval.HC	beta. cholecystitis	se. cholecystitis	pval. cholecystitis	F-statistics
rs11882796	T	A	0.541285	-0.0113842	0.00200309	1.30E-08	-0.0116	0.0118	0.3254	16.04
rs11997077	G	A	0.380458	-0.0112873	0.00206597	4.70E-08	-0.0047	0.0118	0.6886	14.07
rs12072739	G	A	0.224461	0.0149904	0.00238686	3.40E-10	0.0042	0.0126	0.739399	13.73
rs12122361	G	A	0.265617	-0.013773	0.00226034	1.10E-09	-0.0014	0.0128	0.9129	14.49
rs12128526	A	G	0.457416	0.0113684	0.00199597	1.20E-08	0.0116	0.0118	0.326	16.10
rs12364470	G	T	0.164552	0.0178805	0.0026871	2.80E-11	0.0187	0.0141	0.184	12.17
rs12375196	A	C	0.424347	0.0167592	0.00202832	1.40E-16	-0.0026	0.0118	0.8274	33.36
rs12427047	T	C	0.24273	-0.0163996	0.0023233	1.70E-12	0.0046	0.0124	0.7112	18.32
rs12441543	A	G	0.2871	0.0152338	0.0022089	5.30E-12	-0.0117	0.013	0.3681	19.47
rs12462975	A	G	0.329669	0.0189074	0.00213714	9.00E-19	-0.0156	0.0127	0.2173	34.60
rs12519997	A	G	0.559278	-0.0139984	0.00200434	2.90E-12	-0.0036	0.0122	0.7692	24.05
rs12568411	A	G	0.166749	0.0164813	0.00269359	9.40E-10	-0.0232	0.014	0.097949	10.40
rs12569355	G	A	0.119536	0.0217444	0.00307709	1.60E-12	-0.0115	0.0233	0.6223	10.51
rs12607512	G	A	0.44632	0.0117842	0.00200813	4.40E-09	0.0209	0.0118	0.0750205	17.02
rs12631813	G	C	0.50262	-0.0122712	0.00200027	8.50E-10	-0.0121	0.0118	0.3023	18.82
rs12762744	T	C	0.248261	0.0133166	0.00231189	8.40E-09	0.0069	0.013	0.5947	12.38
rs12831185	G	A	0.170442	-0.0176382	0.00265532	3.10E-11	-0.0221	0.0186	0.2337	12.48
rs1285992	G	A	0.710447	0.0203155	0.00220302	2.90E-20	0.0226	0.0133	0.0900098	34.99
rs12880641	G	T	0.661578	-0.0149161	0.00210563	1.40E-12	-0.0138	0.0121	0.2556	22.47
rs12883788	T	C	0.459801	0.0143864	0.00200867	7.90E-13	0.0174	0.0119	0.1455	25.48
rs12921916	C	T	0.28756	0.0125955	0.00222773	1.60E-08	0.0112	0.0135	0.405	13.10
rs12939848	T	C	0.401034	0.0126399	0.00203785	5.60E-10	0.0043	0.0121	0.7224	18.48
rs1294438	T	C	0.354583	0.0186269	0.00210751	9.70E-19	-0.0124	0.0123	0.311	35.76
rs1296328	C	A	0.559033	-0.015815	0.00201508	4.20E-15	-0.0086	0.0118	0.463	30.37

SNP	effect_allele HC	other_alleleHC	eaf. HC	beta.HC	se.HC	pval.HC	beta. cholecystitis	se. cholecystitis	pval. cholecystitis	F-statistics
rs13017207	A	G	0.393634	-0.0161548	0.00203426	2.00E-15	-0.0155	0.012	0.1974	30.11
rs13034765	C	G	0.370011	0.0133745	0.00206373	9.10E-11	0.029	0.0125	0.0198098	19.58
rs13107325	T	C	0.074895	0.0386647	0.0037857	1.70E-24	0.0101	0.0496	0.8382	14.46
rs13148263	A	G	0.335162	0.0159436	0.00210506	3.60E-14	-0.0125	0.0118	0.2928	25.57
rs13156484	A	G	0.472171	-0.0174918	0.00201454	3.90E-18	-0.0024	0.0117	0.8402	37.58
rs13264909	T	A	0.428953	-0.0136063	0.00201731	1.50E-11	0.0191	0.0117	0.1041	22.29
rs13284988	C	T	0.305411	0.0120763	0.00217861	3.00E-08	0.0106	0.0133	0.4241	13.04
rs13292699	C	A	0.433708	-0.0162732	0.00201482	6.70E-16	-0.0139	0.0118	0.2391	32.05
rs133015	G	C	0.439948	0.0129624	0.00201566	1.30E-10	-0.011	0.0119	0.3568	20.38
rs13333747	C	T	0.182674	-0.0219722	0.00259208	2.30E-17	-0.0372	0.0154	0.01583	21.46
rs13389219	T	C	0.392395	0.0227595	0.00203433	4.70E-29	0.0081	0.0123	0.5112	59.69
rs143384	G	A	0.404382	0.0267503	0.00203131	1.30E-39	-0.0039	0.0119	0.742299	83.55
rs1446585	G	A	0.244506	-0.0128503	0.00226761	1.50E-08	7.00E-04	0.0124	0.958	11.86
rs1452082	A	C	0.557071	0.012586	0.00203982	6.80E-10	0.0056	0.012	0.6409	18.79
rs1458156	T	C	0.488404	0.0126488	0.00199454	2.30E-10	0.023	0.0118	0.0507002	20.10
rs1477290	C	T	0.136945	0.0298633	0.00292146	1.60E-24	0.0176	0.018	0.3284	24.70
rs147730268	T	G	0.087233	-0.0506092	0.00361111	1.30E-44	-0.0548	0.0213	0.0100399	31.28
rs1480474	G	A	0.417444	-0.0145096	0.00202337	7.40E-13	0.0264	0.012	0.0282397	25.01
rs1481630	C	T	0.176871	-0.0153923	0.00261541	4.00E-09	-0.0058	0.0167	0.73	10.09
rs1502317	T	C	0.27657	-0.0151583	0.00223176	1.10E-11	0.0031	0.0133	0.8145	18.46
rs1514895	G	A	0.285289	0.0174697	0.00220175	2.10E-15	0.0035	0.0133	0.7944	25.67
rs1538535	T	C	0.27456	0.0124387	0.0022369	2.70E-08	0.0064	0.0142	0.654201	12.32
rs1576655	C	A	0.59598	0.0161325	0.00207546	7.70E-15	0.0063	0.0126	0.615601	29.10
rs1609783	A	G	0.525344	0.0142717	0.0020044	1.10E-12	-0.0053	0.0118	0.6531	25.28

SNP	effect_allele HC	other_alleleHC	eaf. HC	beta.HC	se.HC	pval.HC	beta. cholecystitis	se. cholecystitis	pval. cholecystitis	F-statistics
rs16868443	C	G	0.360418	0.0154349	0.0020756	1.00E-13	0.0177	0.0133	0.1814	25.50
rs170016	A	G	0.633948	-0.0116498	0.00209333	2.60E-08	-0.0255	0.0126	0.0429299	14.37
rs1727901	T	C	0.736676	0.0204425	0.00226027	1.50E-19	0	0.0132	0.9972	31.74
rs17361789	G	T	0.32182	0.0126424	0.00214788	4.00E-09	0.0082	0.0132	0.537	15.12
rs17639546	A	G	0.14849	-0.0187703	0.0028004	2.00E-11	-0.0053	0.0171	0.757	11.36
rs17733217	G	A	0.229127	-0.0158057	0.00237935	3.10E-11	0.0122	0.0152	0.4223	15.59
rs17766836	T	C	0.267888	0.0232323	0.00225241	6.10E-25	-0.005	0.0135	0.7122	41.73
rs17770336	T	C	0.322443	0.0191461	0.00212912	2.40E-19	0.0131	0.0122	0.2799	35.34
rs1813212	G	A	0.445576	-0.0130082	0.00200772	9.20E-11	-0.0189	0.0119	0.1125	20.74
rs1868069	A	G	0.229248	0.0150949	0.00236942	1.90E-10	0.0045	0.0142	0.749	14.34
rs1934394	C	G	0.228157	0.0129993	0.00238257	4.90E-08	0.0081	0.0141	0.5645	10.48
rs1955695	G	A	0.62227	-0.0171244	0.00205855	8.90E-17	-7.00E-04	0.0129	0.9545	32.53
rs2022050	A	G	0.160924	-0.0208767	0.0027204	1.70E-14	0.0092	0.0142	0.517001	15.90
rs2023211	C	T	0.232254	0.0160386	0.00236158	1.10E-11	0.0017	0.0167	0.9185	16.45
rs2032251	A	T	0.507961	0.0114047	0.00199809	1.10E-08	-0.0168	0.0121	0.1641	16.29
rs211434	A	G	0.681996	0.0131339	0.00214133	8.60E-10	-0.0072	0.0124	0.561	16.32
rs2133292	T	C	0.373582	0.0132297	0.00206557	1.50E-10	0.0125	0.0127	0.3242	19.20
rs2133561	T	A	0.611067	-0.0181895	0.00206386	1.20E-18	-0.011	0.0123	0.3729	36.92
rs2151248	C	G	0.730659	-0.0149326	0.00224027	2.60E-11	0.0246	0.0134	0.0667406	17.49
rs2159437	G	A	0.517573	0.0196241	0.00199751	8.90E-23	-0.012	0.012	0.3199	48.20
rs2172131	C	T	0.578698	-0.0136895	0.00202029	1.20E-11	-0.0089	0.0117	0.4492	22.39
rs2178385	T	G	0.320337	0.0185474	0.00213295	3.40E-18	-0.0057	0.0129	0.6582	32.93
rs2238435	G	C	0.613709	0.0259551	0.00205177	1.10E-36	0.0212	0.012	0.0759102	75.89
rs2238689	C	T	0.399438	-0.0141655	0.00203741	3.60E-12	-0.0282	0.0118	0.0167699	23.19

SNP	effect_allele HC	other_alleleHC	eaf. HC	beta.HC	se.HC	pval.HC	beta. cholecystitis	se. cholecystitis	pval. cholecystitis	F-statistics
rs2253310	G	C	0.626098	0.019536	0.00205623	2.10E-21	0.0036	0.0119	0.762901	42.27
rs2270894	G	C	0.203219	-0.0216431	0.00256793	3.50E-17	0.0012	0.0134	0.9282	23.01
rs2288745	T	C	0.299451	0.0136628	0.0021768	3.50E-10	0.0087	0.0122	0.4754	16.53
rs2307111	C	T	0.394977	-0.0285352	0.00203828	1.60E-44	0.0226	0.0119	0.0566905	93.69
rs2371767	C	G	0.277705	0.0194135	0.00221849	2.10E-18	-0.0039	0.0142	0.780899	30.72
rs2371911	A	T	0.544326	0.0131057	0.0019989	5.50E-11	-0.0071	0.0118	0.5506	21.33
rs2384054	C	T	0.489428	0.0219057	0.0019869	2.90E-28	0.0083	0.0119	0.4839	60.76
rs2479958	G	A	0.516982	-0.0129914	0.00201927	1.20E-10	-0.0128	0.0118	0.278	20.67
rs2494196	A	C	0.286135	0.0312737	0.00219937	6.90E-46	-0.0057	0.0129	0.655799	82.61
rs2499468	A	C	0.651137	0.0131826	0.00209013	2.80E-10	-0.0124	0.0133	0.3506	18.07
rs253444	A	G	0.12923	-0.0198245	0.00297188	2.50E-11	-0.0075	0.0151	0.6215	10.01
rs2568958	A	G	0.603693	0.0206705	0.00202892	2.20E-24	0.0147	0.0123	0.2315	49.67
rs2577955	T	C	0.802767	-0.014667	0.00250536	4.80E-09	-6.00E-04	0.0158	0.9694	10.85
rs25849	G	C	0.288829	0.019767	0.00220822	3.50E-19	0.0068	0.0122	0.575999	32.92
rs2585526	G	A	0.557413	0.0122667	0.00201131	1.10E-09	0.0188	0.0118	0.1114	18.35
rs2593169	C	G	0.567965	-0.0116309	0.00201784	8.20E-09	0.0051	0.0118	0.666	16.31
rs2606227	C	T	0.63145	-0.0124349	0.00208271	2.40E-09	-0.0305	0.012	0.0106999	16.59
rs2642305	T	A	0.369566	0.0113043	0.00206693	4.50E-08	-0.0118	0.0137	0.3886	13.94
rs2660241	C	T	0.364912	0.0139138	0.00207299	1.90E-11	0.0095	0.0123	0.439	20.88
rs2678204	G	T	0.340172	0.0196748	0.00209913	7.10E-21	0.0081	0.0129	0.5312	39.44
rs273505	C	T	0.422021	0.0128735	0.00202114	1.90E-10	-0.0204	0.0118	0.0819993	19.79
rs2737263	T	G	0.28052	-0.0236589	0.00222017	1.60E-26	-9.00E-04	0.0127	0.9419	45.84
rs2744956	C	T	0.139254	0.0537577	0.00287012	2.80E-78	0.0283	0.0148	0.05595	84.11
rs2814350	A	G	0.304993	0.0138717	0.00220875	3.40E-10	-0.0101	0.0122	0.4102	16.72

SNP	effect_allele HC	other_alleleHC	eaf. HC	beta.HC	se.HC	pval.HC	beta. cholecystitis	se. cholecystitis	pval. cholecystitis	F-statistics
rs2821226	G	A	0.527763	0.015138	0.00200431	4.30E-14	0.0094	0.0119	0.4291	28.44
rs28366156	C	T	0.130582	-0.0265761	0.00295158	2.20E-19	0.0152	0.0228	0.5043	18.41
rs2861685	C	T	0.411983	-0.0147772	0.0020154	2.30E-13	-0.007	0.0119	0.5566	26.05
rs28778940	A	G	0.330838	0.0151514	0.00212367	9.70E-13	-0.0309	0.0125	0.0137	22.54
rs287837	G	A	0.523844	-0.013078	0.00200242	6.50E-11	-0.0057	0.0118	0.6275	21.28
rs2897968	A	G	0.607035	0.0122759	0.00204428	1.90E-09	0.0177	0.012	0.1395	17.20
rs3012053	G	A	0.714878	-0.0149707	0.00220858	1.20E-11	0.013	0.0125	0.2986	18.73
rs308911	G	A	0.714423	-0.0142761	0.00220578	9.70E-11	0.0113	0.0131	0.387	17.09
rs310796	T	G	0.680961	0.0121086	0.00214394	1.60E-08	0.0036	0.0133	0.787	13.86
rs3110942	A	G	0.481664	0.0127123	0.00199815	2.00E-10	7.00E-04	0.0118	0.9554	20.21
rs3116600	G	A	0.215319	-0.0187688	0.00242539	1.00E-14	0.017	0.0136	0.2088	20.24
rs314288	C	T	0.886256	-0.0227112	0.00313819	4.60E-13	-6.00E-04	0.0178	0.9722	10.56
rs329118	T	C	0.419386	-0.0163479	0.00201987	5.80E-16	0.0252	0.0119	0.0344001	31.90
rs33503	A	G	0.80589	-0.017501	0.00251417	3.40E-12	-0.0114	0.0149	0.4445	15.16
rs340025	C	T	0.57994	0.0127406	0.00202809	3.30E-10	0.0308	0.0126	0.0141198	19.23
rs34223321	A	C	0.300092	0.0140924	0.00217285	8.80E-11	0.0167	0.0123	0.173	17.67
rs34517439	A	C	0.121795	0.0454548	0.00307552	2.00E-49	0.0548	0.0177	0.00191201	46.73
rs34594435	T	C	0.195585	0.0215785	0.00251186	8.60E-18	0.043	0.0153	0.00487102	23.22
rs34629844	G	A	0.12828	0.0212952	0.00297607	8.30E-13	0.0101	0.0205	0.6221	11.45
rs34656389	G	A	0.367473	0.011297	0.00206647	4.60E-08	-0.0018	0.0118	0.8801	13.89
rs34765854	G	T	0.298258	0.0193318	0.00217163	5.50E-19	0.0166	0.0125	0.1854	33.17
rs34769775	T	C	0.29735	-0.0141127	0.00218344	1.00E-10	-0.021	0.0133	0.1141	17.46
rs34772064	G	T	0.555918	-0.0118736	0.00200309	3.10E-09	0.0048	0.0118	0.682299	17.35
rs34811474	A	G	0.230735	-0.0197929	0.00236244	5.40E-17	-0.0357	0.014	0.0109001	24.92

SNP	effect_allele HC	other_alleleHC	eaf. HC	beta.HC	se.HC	pval.HC	beta. cholecystitis	se. cholecystitis	pval. cholecystitis	F-statistics
rs34840745	T	C	0.262486	0.0166433	0.00227207	2.40E-13	-0.0049	0.0131	0.7085	20.78
rs34848742	G	T	0.788211	-0.0143597	0.00243566	3.70E-09	0.004	0.0153	0.7963	11.60
rs35506085	A	G	0.185085	-0.0204796	0.00258575	2.40E-15	-0.0049	0.0156	0.7523	18.92
rs35537311	T	C	0.388607	-0.0147642	0.00205013	6.00E-13	0.0042	0.012	0.7271	24.65
rs35792595	A	T	0.297453	0.0137734	0.00219158	3.30E-10	-0.0188	0.0134	0.1594	16.51
rs35882248	T	C	0.317213	0.015668	0.00213778	2.30E-13	0.0026	0.0127	0.8358	23.27
rs35910339	G	C	0.683708	-0.0127042	0.00214217	3.00E-09	-0.0073	0.0144	0.612999	15.21
rs35917007	G	A	0.533673	0.0147568	0.00199857	1.50E-13	0.0146	0.0119	0.2196	27.14
rs35957544	T	G	0.574337	-0.0192606	0.00202041	1.50E-21	-0.0397	0.012	0.000905899	44.44
rs36140	C	A	0.635313	0.0148434	0.00207958	9.50E-13	0.0392	0.0124	0.001513	23.61
rs365352	A	G	0.24439	-0.0205385	0.0023126	6.60E-19	-0.0091	0.0151	0.5475	29.13
rs3737992	A	G	0.168963	-0.0215132	0.00265121	4.90E-16	0.0056	0.0149	0.7072	18.49
rs3746759	G	T	0.205871	-0.0144809	0.00246759	4.40E-09	0.0263	0.0175	0.1338	11.26
rs3762988	T	C	0.388074	0.0126504	0.0020481	6.50E-10	6.00E-04	0.0123	0.9618	18.12
rs3807566	T	G	0.438301	-0.0149491	0.00201199	1.10E-13	0.0115	0.0121	0.3436	27.18
rs3810291	A	G	0.674991	0.0227348	0.00212876	1.30E-26	0.007	0.0122	0.5628	50.05
rs3811951	G	A	0.282058	0.0150182	0.00221148	1.10E-11	0.0059	0.0129	0.6466	18.68
rs3814883	T	C	0.482403	0.0250685	0.00200044	5.00E-36	0.0031	0.0119	0.7921	78.43
rs3826408	T	C	0.456783	0.0135888	0.00200083	1.10E-11	0.0288	0.0118	0.0146599	22.89
rs3845344	T	C	0.391164	0.0138703	0.00203631	9.70E-12	0.0207	0.0119	0.0802509	22.10
rs3935190	A	G	0.536778	-0.0141131	0.00201245	2.30E-12	-2.00E-04	0.0118	0.9886	24.46
rs394608	C	T	0.537697	0.014611	0.00201006	3.60E-13	0.006	0.0118	0.611399	26.27
rs40071	C	T	0.179489	-0.0174105	0.00260267	2.20E-11	-0.0035	0.0144	0.8099	13.18
rs4017425	T	C	0.470189	-0.0118377	0.00199708	3.10E-09	-0.0193	0.0121	0.1102	17.51

SNP	effect_allele HC	other_alleleHC	eaf. HC	beta.HC	se.HC	pval.HC	beta. cholecystitis	se. cholecystitis	pval. cholecystitis	F-statistics
rs41273794	T	C	0.287079	0.0191445	0.00220216	3.50E-18	0.0141	0.0136	0.2985	30.94
rs4240326	G	A	0.550142	-0.0279318	0.0020006	2.70E-44	-0.0189	0.0118	0.1085	96.50
rs4253755	A	G	0.128527	0.0211316	0.00300303	2.00E-12	-0.0153	0.0214	0.4767	11.09
rs429343	G	A	0.576577	-0.0121964	0.00201501	1.40E-09	-0.0072	0.0118	0.542199	17.89
rs4310395	G	A	0.442854	0.0122183	0.00200242	1.00E-09	0.0204	0.0118	0.0847208	18.37
rs4476935	T	C	0.433157	-0.0128732	0.00201066	1.50E-10	-0.003	0.012	0.8034	20.13
rs4477562	T	C	0.128661	0.0292203	0.00300396	2.30E-22	0.0361	0.017	0.03359	21.22
rs4486868	C	T	0.447992	-0.011105	0.00201104	3.40E-08	8.00E-04	0.0119	0.9494	15.08
rs4567604	T	G	0.193192	-0.0166838	0.00253879	5.00E-11	-0.0163	0.0155	0.2927	13.46
rs4589131	G	T	0.590569	-0.0117346	0.00202078	6.40E-09	-0.0038	0.0119	0.75	16.31
rs4613074	C	T	0.18516	-0.0170225	0.00256625	3.30E-11	0.007	0.0156	0.6549	13.28
rs463376	A	G	0.490305	0.0119932	0.0019975	1.90E-09	0.02	0.0117	0.0887892	18.02
rs4678016	T	C	0.366307	0.0115138	0.00206489	2.50E-08	0.008	0.0118	0.498901	14.43
rs475390	A	G	0.776432	-0.0140664	0.00238863	3.90E-09	0.0095	0.0141	0.5022	12.04
rs4790292	A	C	0.153689	-0.0261893	0.00277723	4.10E-21	0.0031	0.0157	0.8443	23.13
rs4792716	G	A	0.562173	0.0145605	0.00201188	4.60E-13	-6.00E-04	0.012	0.9571	25.79
rs4800490	C	A	0.495235	-0.023172	0.00199562	3.60E-31	-0.0322	0.0118	0.00620497	67.42
rs4820346	G	C	0.69295	-0.0130489	0.00217064	1.80E-09	-0.0321	0.012	0.00757897	15.38
rs483465	G	A	0.749331	0.0154934	0.00229726	1.50E-11	-0.0198	0.0163	0.2257	17.09
rs4843158	C	G	0.68516	0.0218462	0.00214891	2.80E-24	-0.0113	0.0127	0.3716	44.59
rs4870057	G	A	0.341223	0.0126926	0.00212695	2.40E-09	-0.0121	0.0139	0.3838	16.01
rs4909309	C	T	0.393916	-0.0173539	0.00203923	1.70E-17	-0.0107	0.0117	0.3625	34.58
rs4962424	A	T	0.326831	0.0155846	0.00212749	2.40E-13	0.0217	0.0131	0.0969996	23.61
rs4963975	A	G	0.244315	-0.0187917	0.00232365	6.10E-16	0.0143	0.0131	0.2719	24.15

SNP	effect_allele HC	other_alleleHC	eaf. HC	beta.HC	se.HC	pval.HC	beta. cholecystitis	se. cholecystitis	pval. cholecystitis	F-statistics
rs4976994	G	A	0.452549	0.0128387	0.00200503	1.50E-10	-0.0084	0.0118	0.4759	20.32
rs4985407	G	A	0.49763	0.0153026	0.00199743	1.80E-14	0.0038	0.012	0.754401	29.35
rs522110	G	A	0.555042	0.0181191	0.0020069	1.70E-19	0.0256	0.0118	0.03002	40.27
rs543874	G	A	0.205218	0.0453641	0.00246182	8.00E-76	0.0202	0.0154	0.189	110.79
rs551935	G	T	0.440778	-0.016925	0.00200492	3.10E-17	-0.0186	0.0124	0.1334	35.13
rs55726687	A	G	0.209736	0.0218167	0.00244465	4.50E-19	0.0096	0.0143	0.5011	26.40
rs56094641	G	A	0.404596	0.0616325	0.00203052	1.00E-200	0.0468	0.0119	8.17E-05	444.31
rs56097510	G	C	0.51052	0.0137125	0.00199793	6.70E-12	0.0035	0.0117	0.7641	23.54
rs56399737	T	C	0.449109	-0.0118202	0.00201255	4.30E-09	0.0048	0.0119	0.6848	17.07
rs57222629	C	G	0.311673	0.0138153	0.0021564	1.50E-10	-0.0016	0.0122	0.8958	17.61
rs57235969	T	C	0.554199	0.0124617	0.00199957	4.60E-10	0.0208	0.0118	0.07758	19.19
rs57636386	C	T	0.08382	-0.0350845	0.0036118	2.60E-22	-0.036	0.0271	0.184	14.49
rs57989773	C	T	0.245008	0.021874	0.00238057	4.00E-20	0.0342	0.0148	0.02067	31.24
rs58584712	A	G	0.211251	0.0169765	0.0024357	3.20E-12	-0.0011	0.0155	0.9436	16.19
rs58862095	T	C	0.419278	-0.0181051	0.00202425	3.70E-19	-0.0076	0.0118	0.5178	38.96
rs59227842	G	A	0.311488	0.0210227	0.00217036	3.40E-22	0.0114	0.0127	0.3681	40.25
rs60226453	T	C	0.177273	0.0162724	0.00260018	3.90E-10	-0.002	0.0159	0.8984	11.42
rs6066104	T	C	0.324108	0.0192833	0.00213455	1.70E-19	0.0098	0.0122	0.4238	35.76
rs6142059	C	T	0.492562	0.0149966	0.00200021	6.50E-14	0.0177	0.0118	0.1317	28.10
rs614520	A	G	0.640637	-0.0130274	0.00212026	8.00E-10	-0.0055	0.0125	0.660601	17.38
rs61941722	A	G	0.188737	0.0155992	0.00256577	1.20E-09	-0.0018	0.0151	0.9033	11.32
rs61969510	C	T	0.278956	0.0128445	0.00224311	1.00E-08	0.0311	0.0122	0.0107199	13.19
rs61992671	G	A	0.491978	-0.0151753	0.00208557	3.40E-13	0.0061	0.012	0.609399	26.47

SNP	effect_allele HC	other_alleleHC	eaf. HC	beta.HC	se.HC	pval.HC	beta. cholecystitis	se. cholecystitis	pval. cholecystitis	F-statistics
rs62070648	A	G	0.268727	-0.0199439	0.0022501	7.80E-19	0.0065	0.0131	0.6191	30.88
rs62107261	C	T	0.04832	-0.0808476	0.00465016	1.10E-67	-0.03	0.0415	0.4702	27.80
rs62301134	C	T	0.263286	-0.013638	0.00226745	1.80E-09	-0.0235	0.0131	0.0718307	14.03
rs62396185	C	G	0.259989	-0.0346069	0.00227012	1.80E-52	-0.0105	0.0121	0.3879	89.44
rs62425398	A	C	0.106309	0.0248634	0.00324644	1.90E-14	-0.0173	0.0224	0.4402	11.15
rs62515438	G	T	0.227665	0.0150395	0.00237712	2.50E-10	0.0312	0.0129	0.0153102	14.08
rs643499	C	T	0.398022	0.0119575	0.00204369	4.90E-09	0.0143	0.0118	0.2274	16.41
rs6438656	A	G	0.635867	-0.0119598	0.0020673	7.20E-09	-0.0156	0.0124	0.2095	15.50
rs6465828	T	G	0.482837	0.0163908	0.00199173	1.90E-16	0.0089	0.0119	0.4543	33.82
rs6545714	A	G	0.60145	-0.0158267	0.00203362	7.10E-15	-0.0141	0.012	0.2389	29.04
rs6567160	C	T	0.232716	0.0523537	0.00236068	5.70E-109	0.0235	0.0151	0.1205	175.71
rs6575340	A	G	0.636028	0.0184545	0.00207818	6.70E-19	-0.0027	0.0121	0.8221	36.51
rs6597975	G	C	0.543875	0.0122921	0.00201118	9.80E-10	-0.0202	0.0119	0.0896396	18.53
rs6598540	G	A	0.276885	-0.0147876	0.00223088	3.40E-11	-0.0152	0.0136	0.2645	17.60
rs6606686	C	G	0.680627	-0.0165692	0.00213651	8.80E-15	-0.0018	0.0126	0.8836	26.15
rs66679256	T	C	0.445805	0.0149901	0.00200438	7.50E-14	0.0064	0.0122	0.60	27.64
rs6669341	G	A	0.582713	-0.0151291	0.00201544	6.10E-14	-0.0037	0.0121	0.7597	27.41
rs667515	C	G	0.386143	-0.0141684	0.00205316	5.20E-12	0.006	0.0124	0.631401	22.58
rs6744646	G	A	0.828323	0.0500537	0.00263558	2.00E-80	0.02	0.0159	0.2074	102.60
rs6745626	T	C	0.589593	0.0139294	0.00202493	6.00E-12	0.0104	0.0121	0.3911	22.90
rs6747657	A	G	0.281723	0.0129341	0.00220754	4.70E-09	0.0305	0.0132	0.02069	13.89
rs675162	G	A	0.482059	0.0180079	0.00199495	1.80E-19	0.0123	0.0118	0.2941	40.69
rs6840236	C	T	0.464856	0.0169824	0.00199864	1.90E-17	0.012	0.0118	0.3098	35.92

SNP	effect_allele HC	other_alleleHC	eaf. HC	beta.HC	se.HC	pval.HC	beta. cholecystitis	se. cholecystitis	pval. cholecystitis	F-statistics
rs6868125	T	C	0.521906	-0.0119493	0.00198836	1.90E-09	0.0102	0.012	0.3931	18.02
rs6946415	G	A	0.627099	0.0158826	0.00206409	1.40E-14	0.0243	0.0125	0.0517905	27.69
rs6958365	T	C	0.411809	-0.0115375	0.00203582	1.50E-08	-0.0017	0.0122	0.8869	15.56
rs6973700	G	A	0.20616	-0.0157823	0.00246577	1.50E-10	-3.00E-04	0.0132	0.9809	13.41
rs698147	G	A	0.543563	-0.0121448	0.00200109	1.30E-09	-0.0113	0.0118	0.3361	18.28
rs7002088	C	A	0.445199	0.0137173	0.00200556	7.90E-12	-0.0175	0.0136	0.1989	23.11
rs7034554	G	A	0.373815	-0.012422	0.0020595	1.60E-09	-0.0353	0.0129	0.00635097	17.03
rs7038943	C	T	0.338791	-0.0121087	0.00210372	8.60E-09	-0.0213	0.012	0.0766708	14.84
rs705165	T	G	0.247646	0.0148091	0.00230698	1.40E-10	0.0011	0.0139	0.935	15.36
rs7094073	T	C	0.191266	0.0163482	0.00273496	2.30E-09	-0.022	0.017	0.1958	11.05
rs7111235	C	T	0.493399	0.0115415	0.00200364	8.40E-09	0.0221	0.0123	0.0727394	16.59
rs7124681	A	C	0.408387	0.0205032	0.00202231	3.70E-24	0.0203	0.0123	0.099901	49.67
rs7132908	A	G	0.384469	0.0257741	0.00204901	2.80E-36	0.0196	0.0121	0.1055	74.90
rs71413981	A	G	0.162848	0.021194	0.00271023	5.30E-15	0.0122	0.0172	0.4769	16.67
rs7171864	A	G	0.660205	0.0161986	0.00211572	1.90E-14	0.0123	0.0129	0.342	26.30
rs7218014	C	T	0.197308	0.0207669	0.00251132	1.30E-16	0.0242	0.0138	0.0792702	21.66
rs7248205	T	C	0.600262	-0.0161199	0.00204315	3.00E-15	0.0048	0.0121	0.6884	29.87
rs7257083	A	G	0.288605	0.0162612	0.0022051	1.70E-13	-0.0063	0.015	0.676	22.33
rs72634826	A	G	0.259879	-0.0153317	0.00229923	2.60E-11	-0.0029	0.0145	0.8431	17.11
rs72656010	C	T	0.132181	-0.0252479	0.00295341	1.20E-17	-0.0265	0.0177	0.1346	16.77
rs7274811	T	G	0.258589	-0.01951	0.00227858	1.10E-17	-0.0105	0.014	0.4515	28.11
rs72756476	C	T	0.141428	-0.0192477	0.00287344	2.10E-11	0.0278	0.0192	0.1472	10.90
rs73052033	C	T	0.184918	-0.0208103	0.00256783	5.30E-16	-0.0018	0.0159	0.9112	19.80
rs73175572	G	A	0.111726	0.0282075	0.00319248	1.00E-18	-4.00E-04	0.0183	0.9812	15.50

SNP	effect_allele HC	other_alleleHC	eaf. HC	beta.HC	se.HC	pval.HC	beta. cholecystitis	se. cholecystitis	pval. cholecystitis	F-statistics
rs73213484	T	A	0.141217	-0.0207006	0.00286036	4.60E-13	-0.0057	0.0166	0.731799	12.70
rs7365	G	A	0.482654	-0.0110939	0.00199385	2.60E-08	-0.0196	0.0117	0.0949205	15.46
rs7372674	A	C	0.357244	0.014356	0.00207575	4.60E-12	0.019	0.0128	0.137	21.97
rs743572	G	A	0.376473	0.0151185	0.00205743	2.00E-13	0.0027	0.0121	0.822	25.35
rs7442885	G	C	0.214027	-0.0198682	0.00243204	3.10E-16	-0.0237	0.0151	0.1166	22.45
rs7460093	A	G	0.53118	0.0136639	0.0020144	1.20E-11	-0.0039	0.0119	0.745799	22.92
rs74749286	A	G	0.107637	0.0258963	0.00323591	1.20E-15	0.0267	0.014	0.0575705	12.30
rs7498665	G	A	0.399666	0.0317857	0.00203647	6.40E-55	0.0203	0.0119	0.0885707	116.93
rs750090	C	T	0.35675	-0.0132955	0.00210095	2.50E-10	0.0015	0.012	0.8985	18.38
rs7519259	A	G	0.528388	0.0141441	0.00200058	1.50E-12	0.0086	0.0118	0.4661	24.91
rs756717	A	G	0.399077	-0.0140302	0.00206015	9.70E-12	-0.0179	0.012	0.1379	22.25
rs7570446	A	C	0.544459	0.0110158	0.00199439	3.30E-08	-0.0017	0.0119	0.8886	15.13
rs7571496	G	A	0.260562	-0.0138156	0.00227445	1.20E-09	-0.0246	0.0121	0.04171	14.22
rs7582359	A	G	0.331712	-0.0135492	0.00212104	1.70E-10	0.0105	0.0126	0.4022	18.09
rs7632381	C	T	0.444325	0.0270429	0.00200044	1.20E-41	0.0071	0.0118	0.547299	90.26
rs765874	A	T	0.489353	-0.0168012	0.0019903	3.10E-17	-0.007	0.0119	0.5571	35.62
rs76647086	T	G	0.176857	-0.0202229	0.00260327	8.00E-15	-0.022	0.0155	0.1563	17.57
rs76798800	T	G	0.266025	0.0244232	0.00225319	2.20E-27	-0.0063	0.0139	0.651	45.89
rs7695177	G	C	0.470976	-0.0151527	0.00199491	3.10E-14	0.0198	0.0118	0.0929095	28.75
rs7696175	C	T	0.526478	0.0136068	0.00200383	1.10E-11	-0.0032	0.0118	0.784599	22.99
rs7707394	A	G	0.357269	-0.0184703	0.002075	5.50E-19	0.0322	0.0121	0.00788007	36.39
rs7708584	G	A	0.572378	-0.013196	0.00201087	5.30E-11	-0.0126	0.012	0.2949	21.08
rs7740107	A	T	0.736289	-0.0236486	0.00225676	1.10E-25	-0.0106	0.0133	0.4222	42.65
rs7793674	C	A	0.144347	0.019239	0.00284373	1.30E-11	0.0042	0.0491	0.9319	11.31

SNP	effect_allele HC	other_alleleHC	eaf. HC	beta.HC	se.HC	pval.HC	beta. cholecystitis	se. cholecystitis	pval. cholecystitis	F-statistics
rs7793674	C	A	0.144347	0.019239	0.00284373	1.30E-11	0.0086	0.0222	0.697301	11.31
rs779655	C	G	0.729184	0.0143261	0.00224052	1.60E-10	-8.00E-04	0.0126	0.9493	16.15
rs7805441	T	C	0.502255	0.0126844	0.00200504	2.50E-10	4.00E-04	0.0118	0.9713	20.01
rs7864465	A	G	0.559379	-0.0110794	0.00200561	3.30E-08	0.0087	0.0119	0.462701	15.04
rs7893571	T	G	0.665871	0.0138381	0.00211863	6.50E-11	0.0057	0.0128	0.655101	18.98
rs7903146	T	C	0.290662	-0.0214133	0.00219381	1.70E-22	-0.0084	0.0147	0.569799	39.29
rs7944782	G	T	0.50981	0.0129576	0.0020033	9.90E-11	0.009	0.0118	0.4449	20.91
rs7952436	T	C	0.081996	-0.0354571	0.00363182	1.60E-22	-0.0705	0.0268	0.00842403	14.35
rs7996639	A	G	0.449371	0.0137138	0.00201868	1.10E-11	0.0157	0.0118	0.1825	22.84
rs8030456	T	C	0.226311	-0.0247721	0.00237863	2.10E-25	-0.0086	0.0159	0.5895	37.98
rs8132129	T	C	0.184901	-0.015922	0.00259474	8.40E-10	-0.0024	0.0143	0.865	11.35
rs8133137	G	A	0.664097	0.0157777	0.00211541	8.80E-14	0.0323	0.0119	0.00646294	24.82
rs815163	C	T	0.563175	-0.0168978	0.00200258	3.20E-17	-0.0126	0.0118	0.2848	35.03
rs852042	G	A	0.758582	-0.0153695	0.00233046	4.30E-11	-0.0169	0.0139	0.2228	15.93
rs852983	A	G	0.459576	-0.0118657	0.00199745	2.80E-09	0.0031	0.0117	0.7898	17.53
rs866006	G	T	0.618508	-0.0120595	0.00204995	4.00E-09	-0.0034	0.012	0.774901	16.33
rs897186	G	A	0.548592	-0.0160621	0.00199856	9.20E-16	-0.0178	0.0119	0.136	31.99
rs9284814	A	G	0.884815	0.0230203	0.00311527	1.50E-13	0.0257	0.0184	0.1621	11.13
rs9378684	T	C	0.200589	0.018067	0.00250737	5.80E-13	0.006	0.0132	0.646399	16.65
rs9385385	T	C	0.447856	0.012816	0.00201537	2.00E-10	-0.0019	0.0119	0.8732	20.00
rs9512696	G	A	0.66154	0.0172114	0.00211114	3.60E-16	0.0228	0.0125	0.0671506	29.77
rs9513018	T	G	0.616147	-0.0112355	0.00205443	4.50E-08	-0.0105	0.012	0.3831	14.15
rs968379	T	C	0.229098	-0.0187833	0.00237244	2.40E-15	-0.0055	0.014	0.6941	22.14
rs9764678	C	T	0.272455	0.0161285	0.00225012	7.60E-13	8.00E-04	0.0125	0.9499	20.37

SNP	effect_allele HC	other_alleleHC	eaf. HC	beta.HC	se.HC	pval.HC	beta. cholecystitis	se. cholecystitis	pval. cholecystitis	F-statistics
rs9788550	C	G	0.247466	-0.0214137	0.00232004	2.70E-20	-0.0113	0.0124	0.3599	31.73
rs9830592	A	C	0.582425	0.0138364	0.00201841	7.10E-12	0.0078	0.0122	0.5248	22.86
rs9843653	C	T	0.511657	0.0183267	0.00199203	3.60E-20	0.0275	0.012	0.0222802	42.30
rs9845755	T	A	0.198555	0.027361	0.0024977	6.30E-28	-0.0054	0.0155	0.727301	38.19
rs9850529	A	G	0.339796	0.0114818	0.00209898	4.50E-08	-0.02	0.013	0.125	13.43
rs987237	G	A	0.17957	0.0344542	0.00259141	2.50E-40	0.009	0.0144	0.5321	52.09
rs9876664	T	G	0.3754	-0.0143788	0.00205893	2.90E-12	-0.024	0.0118	0.0420001	22.87
rs9951619	G	T	0.767352	0.0138525	0.00237634	5.60E-09	0.0156	0.0126	0.2143	12.13
rs9967287	T	G	0.251722	0.0141454	0.00230082	7.80E-10	0.0121	0.0122	0.3197	14.24
rs9967367	T	C	0.293494	-0.0174208	0.00220445	2.70E-15	0.0014	0.0123	0.9098	25.90
rs998584	A	C	0.482802	-0.0222736	0.00199467	5.90E-29	3.00E-04	0.0118	0.982	62.28

Abbreviations: SNP, single nucleotide polymorphism; se, standard error; HC, hip circumference; pval, p-value.

Table S12. Detailed information about single-nucleotide polymorphisms of hip circumference on intestinal infections.

SNP	effect_allele HC	other_alleleHC	eaf. HC	beta.HC	se.HC	pval.HC	beta. II	se. II	pval. II	F-statistics
rs1006399	A	G	0.458873	-0.0114857	0.00200117	9.50E-09	0.0046	0.023	0.8416	16.36
rs10100245	A	G	0.564506	0.0205386	0.0020121	1.80E-24	0.0331	0.0226	0.1439	51.24
rs10118701	G	A	0.32209	0.0168068	0.00213097	3.10E-15	-0.0207	0.0233	0.3747	27.17
rs10132514	T	C	0.273182	-0.0131415	0.00225463	5.60E-09	-0.0278	0.0275	0.3121	13.49
rs1013402	G	A	0.318427	0.0293703	0.002136	5.10E-43	-0.0312	0.0239	0.1915	82.08
rs10145154	T	C	0.22178	0.0253835	0.00240401	4.60E-26	-0.0032	0.0265	0.9024	38.49
rs10153248	G	A	0.447835	-0.0158858	0.00200883	2.60E-15	-0.005	0.0233	0.8291	30.93
rs10172196	A	G	0.305442	0.0174435	0.00216332	7.40E-16	0.0097	0.0241	0.6866	27.59
rs10210468	C	T	0.464304	-0.0137575	0.00201939	9.60E-12	-2.00E-04	0.0228	0.9945	23.09
rs1023767	A	G	0.235192	-0.015354	0.00234795	6.20E-11	-0.03	0.0234	0.2005	15.38
rs1037702	A	G	0.62178	-0.0116182	0.00205972	1.70E-08	-0.0178	0.0233	0.4434	14.97
rs10404726	T	C	0.465501	-0.0166459	0.00200329	9.60E-17	0.0117	0.0226	0.6046	34.36
rs10407871	C	T	0.155426	-0.0181966	0.00276505	4.70E-11	-0.0193	0.03	0.519501	11.37
rs10471636	A	G	0.508884	-0.0118317	0.00203156	5.70E-09	0.0274	0.0228	0.2294	16.95
rs1047891	A	C	0.315753	0.0166025	0.00213661	7.80E-15	0.002	0.0243	0.9347	26.09
rs1056441	C	T	0.675428	0.014638	0.002132	6.60E-12	0.0161	0.0259	0.5346	20.67
rs1056720	T	C	0.234833	-0.0130647	0.00235122	2.80E-08	-0.0076	0.0285	0.7897	11.10
rs10744145	A	C	0.513795	-0.0121183	0.00199668	1.30E-09	0.0194	0.0227	0.3927	18.40
rs10746833	G	A	0.581617	-0.0149421	0.0020362	2.20E-13	-0.0165	0.0228	0.468	26.21
rs10773051	T	C	0.222167	0.0231417	0.00240285	5.90E-22	0.0031	0.0249	0.9021	32.06
rs10810598	A	T	0.639535	-0.0129254	0.00207775	4.90E-10	0.0097	0.0237	0.683	17.84
rs10883553	A	C	0.445855	0.0151046	0.00200686	5.20E-14	-0.0109	0.0229	0.6346	27.99
rs10938397	G	A	0.434366	0.02439	0.00201147	7.70E-34	0.0093	0.0226	0.6826	72.26

SNP	effect_allele HC	other_alleleHC	eaf. HC	beta.HC	se.HC	pval.HC	beta. II	se. II	pval. II	F-statistics
rs10954284	A	T	0.488124	0.0193137	0.00199245	3.20E-22	-0.0276	0.0226	0.2231	46.96
rs10987417	T	G	0.385638	0.0128078	0.00206992	6.10E-10	-0.0068	0.0228	0.7643	18.14
rs11012732	G	A	0.331674	0.0200889	0.00211859	2.50E-21	-3.00E-04	0.0243	0.9915	39.86
rs11030016	T	C	0.739706	0.018143	0.00227588	1.60E-15	0.0077	0.0242	0.7516	24.47
rs11045163	G	A	0.430597	0.0114512	0.00201589	1.30E-08	-0.0115	0.0232	0.6206	15.82
rs11075252	G	A	0.284481	-0.012562	0.00221467	1.40E-08	-0.012	0.0242	0.619901	13.10
rs1108548	G	A	0.27725	0.0156522	0.00222734	2.10E-12	0.0087	0.0245	0.721499	19.79
rs11107114	A	G	0.228885	0.0133238	0.00237525	2.00E-08	0.0379	0.024	0.1143	11.11
rs11113445	G	A	0.391358	0.0127502	0.00203858	4.00E-10	0.02	0.0233	0.3913	18.64
rs11150461	G	C	0.727351	-0.0141564	0.00224714	3.00E-10	-0.012	0.0278	0.665701	15.74
rs11150745	G	A	0.317702	-0.0202757	0.00214598	3.40E-21	-0.0225	0.0253	0.3741	38.70
rs11164630	T	C	0.608331	-0.0138102	0.00204155	1.30E-11	0.0512	0.0238	0.0311903	21.81
rs11165643	T	C	0.590085	0.0185255	0.00202023	4.70E-20	0.0166	0.0231	0.472799	40.68
rs11173522	A	C	0.214329	0.013694	0.00242935	1.70E-08	-0.022	0.0286	0.4417	10.70
rs1123295	G	A	0.444369	0.0119544	0.00200534	2.50E-09	0.0197	0.0229	0.3884	17.55
rs11263719	T	C	0.469657	0.0114989	0.00200822	1.00E-08	-0.0236	0.0231	0.3063	16.33
rs112875651	A	G	0.391041	0.0182659	0.00206839	1.00E-18	-0.0252	0.0233	0.2791	37.14
rs113230003	A	G	0.260523	-0.014859	0.00229209	9.00E-11	0.0282	0.0279	0.3118	16.19
rs11513729	T	C	0.413028	-0.0166494	0.00205054	4.70E-16	0.0279	0.0233	0.2319	31.97
rs11664106	T	A	0.37403	0.016775	0.00211301	2.00E-15	0.0032	0.0244	0.8946	29.51
rs11704728	T	C	0.196356	0.0148323	0.00252373	4.20E-09	0.0376	0.0275	0.1718	10.90
rs11751684	T	G	0.275389	0.0167319	0.0022233	5.20E-14	-0.0054	0.0293	0.8529	22.60
rs11762444	A	G	0.216137	0.014599	0.00241918	1.60E-09	-0.0325	0.0284	0.2524	12.34
rs11778934	G	C	0.536083	-0.0141306	0.00200504	1.80E-12	-0.0081	0.0237	0.733599	24.71

SNP	effect_allele HC	other_alleleHC	eaf. HC	beta.HC	se.HC	pval.HC	beta. II	se. II	pval. II	F-statistics
rs11779446	G	A	0.161259	-0.020178	0.00271964	1.20E-13	0.0052	0.0324	0.8732	14.89
rs1182199	A	C	0.304395	-0.0246541	0.00216674	5.40E-30	0.0023	0.0235	0.9233	54.83
rs11882796	T	A	0.541285	-0.0113842	0.00200309	1.30E-08	0.0807	0.0228	0.000401597	16.04
rs11997077	G	A	0.380458	-0.0112873	0.00206597	4.70E-08	0.0131	0.0228	0.5652	14.07
rs12072739	G	A	0.224461	0.0149904	0.00238686	3.40E-10	0.0203	0.0242	0.4017	13.73
rs12122361	G	A	0.265617	-0.013773	0.00226034	1.10E-09	0.014	0.0246	0.569399	14.49
rs12128526	A	G	0.457416	0.0113684	0.00199597	1.20E-08	-0.0168	0.0227	0.4596	16.10
rs12364470	G	T	0.164552	0.0178805	0.0026871	2.80E-11	-0.0044	0.0271	0.8721	12.17
rs12375196	A	C	0.424347	0.0167592	0.00202832	1.40E-16	-0.0047	0.0228	0.8383	33.36
rs12427047	T	C	0.24273	-0.0163996	0.0023233	1.70E-12	0.0215	0.0238	0.3681	18.32
rs12441543	A	G	0.2871	0.0152338	0.0022089	5.30E-12	0.0283	0.025	0.2581	19.47
rs12462975	A	G	0.329669	0.0189074	0.00213714	9.00E-19	-0.027	0.0244	0.2672	34.60
rs12519997	A	G	0.559278	-0.0139984	0.00200434	2.90E-12	-0.0359	0.0236	0.1287	24.05
rs12568411	A	G	0.166749	0.0164813	0.00269359	9.40E-10	-0.0436	0.0269	0.1051	10.40
rs12569355	G	A	0.119536	0.0217444	0.00307709	1.60E-12	-0.0026	0.0456	0.955	10.51
rs1260326	C	T	0.604267	0.0120369	0.00203008	3.00E-09	0.0292	0.0237	0.2175	16.81
rs12607512	G	A	0.44632	0.0117842	0.00200813	4.40E-09	-0.0011	0.0227	0.9606	17.02
rs12631813	G	C	0.50262	-0.0122712	0.00200027	8.50E-10	0.0307	0.0227	0.1753	18.82
rs12762744	T	C	0.248261	0.0133166	0.00231189	8.40E-09	0.0026	0.0249	0.9178	12.38
rs12831185	G	A	0.170442	-0.0176382	0.00265532	3.10E-11	-0.0294	0.0359	0.4121	12.48
rs1285992	G	A	0.710447	0.0203155	0.00220302	2.90E-20	-0.002	0.0257	0.9384	34.99
rs12880641	G	T	0.661578	-0.0149161	0.00210563	1.40E-12	-0.0249	0.0233	0.2853	22.47
rs12883788	T	C	0.459801	0.0143864	0.00200867	7.90E-13	-0.006	0.0229	0.7922	25.48
rs12921916	C	T	0.28756	0.0125955	0.00222773	1.60E-08	0.0106	0.0259	0.6806	13.10

SNP	effect_allele HC	other_alleleHC	eaf. HC	beta.HC	se.HC	pval.HC	beta. II	se. II	pval. II	F-statistics
rs12939848	T	C	0.401034	0.0126399	0.00203785	5.60E-10	0.0203	0.0233	0.3836	18.48
rs1294438	T	C	0.354583	0.0186269	0.00210751	9.70E-19	0.0154	0.0236	0.5142	35.76
rs1296328	C	A	0.559033	-0.015815	0.00201508	4.20E-15	0.0123	0.0227	0.5881	30.37
rs13017207	A	G	0.393634	-0.0161548	0.00203426	2.00E-15	-0.0073	0.0231	0.7519	30.11
rs13034765	C	G	0.370011	0.0133745	0.00206373	9.10E-11	-0.0333	0.024	0.1659	19.58
rs13107325	T	C	0.074895	0.0386647	0.0037857	1.70E-24	-0.1233	0.0965	0.2015	14.46
rs13148263	A	G	0.335162	0.0159436	0.00210506	3.60E-14	0.0094	0.0229	0.6797	25.57
rs13156484	A	G	0.472171	-0.0174918	0.00201454	3.90E-18	0.0238	0.0227	0.2947	37.58
rs13264909	T	A	0.428953	-0.0136063	0.00201731	1.50E-11	0.0055	0.0227	0.8076	22.29
rs13284988	C	T	0.305411	0.0120763	0.00217861	3.00E-08	-0.0194	0.0257	0.4511	13.04
rs13292699	C	A	0.433708	-0.0162732	0.00201482	6.70E-16	0.0462	0.0227	0.04132	32.05
rs133015	G	C	0.439948	0.0129624	0.00201566	1.30E-10	0.0447	0.023	0.0524204	20.38
rs13333747	C	T	0.182674	-0.0219722	0.00259208	2.30E-17	0.0235	0.0295	0.4256	21.46
rs13389219	T	C	0.392395	0.0227595	0.00203433	4.70E-29	-0.0097	0.0237	0.6819	59.69
rs143384	G	A	0.404382	0.0267503	0.00203131	1.30E-39	0.0192	0.0229	0.4001	83.55
rs1446585	G	A	0.244506	-0.0128503	0.00226761	1.50E-08	-0.0207	0.0238	0.3859	11.86
rs1452082	A	C	0.557071	0.012586	0.00203982	6.80E-10	0.0273	0.0231	0.2373	18.79
rs1458156	T	C	0.488404	0.0126488	0.00199454	2.30E-10	0.0117	0.0227	0.606001	20.10
rs1477290	C	T	0.136945	0.0298633	0.00292146	1.60E-24	-0.0251	0.0347	0.4691	24.70
rs147730268	T	G	0.087233	-0.0506092	0.00361111	1.30E-44	0.0397	0.0411	0.3335	31.28
rs1480474	G	A	0.417444	-0.0145096	0.00202337	7.40E-13	-0.031	0.0232	0.1809	25.01
rs1481630	C	T	0.176871	-0.0153923	0.00261541	4.00E-09	-0.0061	0.0322	0.849	10.09
rs1502317	T	C	0.27657	-0.0151583	0.00223176	1.10E-11	-0.0098	0.0257	0.7038	18.46
rs1514895	G	A	0.285289	0.0174697	0.00220175	2.10E-15	-0.0067	0.0256	0.792399	25.67

SNP	effect_allele HC	other_alleleHC	eaf. HC	beta.HC	se.HC	pval.HC	beta. II	se. II	pval. II	F-statistics
rs1538535	T	C	0.27456	0.0124387	0.0022369	2.70E-08	-0.0099	0.0274	0.717	12.32
rs1576655	C	A	0.59598	0.0161325	0.00207546	7.70E-15	-0.0019	0.0242	0.9387	29.10
rs1609783	A	G	0.525344	0.0142717	0.0020044	1.10E-12	-0.0178	0.0227	0.4319	25.28
rs16868443	C	G	0.360418	0.0154349	0.0020756	1.00E-13	-0.0349	0.0257	0.174	25.50
rs170016	A	G	0.633948	-0.0116498	0.00209333	2.60E-08	-0.0257	0.0242	0.2888	14.37
rs1727901	T	C	0.736676	0.0204425	0.00226027	1.50E-19	0.0342	0.0255	0.1801	31.74
rs17361789	G	T	0.32182	0.0126424	0.00214788	4.00E-09	0.0138	0.0256	0.5909	15.12
rs17639546	A	G	0.14849	-0.0187703	0.0028004	2.00E-11	-0.015	0.0327	0.646501	11.36
rs17733217	G	A	0.229127	-0.0158057	0.00237935	3.10E-11	0.0028	0.0293	0.9235	15.59
rs17766836	T	C	0.267888	0.0232323	0.00225241	6.10E-25	0.0075	0.0262	0.7756	41.73
rs17770336	T	C	0.322443	0.0191461	0.00212912	2.40E-19	-0.0355	0.0233	0.1273	35.34
rs1813212	G	A	0.445576	-0.0130082	0.00200772	9.20E-11	0.0448	0.0229	0.0504905	20.74
rs1868069	A	G	0.229248	0.0150949	0.00236942	1.90E-10	0.0011	0.0273	0.9686	14.34
rs1934394	C	G	0.228157	0.0129993	0.00238257	4.90E-08	-0.0193	0.0273	0.4799	10.48
rs1955695	G	A	0.62227	-0.0171244	0.00205855	8.90E-17	0.0306	0.0249	0.2195	32.53
rs2022050	A	G	0.160924	-0.0208767	0.0027204	1.70E-14	0.0064	0.0273	0.8148	15.90
rs2023211	C	T	0.232254	0.0160386	0.00236158	1.10E-11	-0.0391	0.0324	0.228	16.45
rs2032251	A	T	0.507961	0.0114047	0.00199809	1.10E-08	-0.0018	0.0234	0.9396	16.29
rs211434	A	G	0.681996	0.0131339	0.00214133	8.60E-10	0.0131	0.0239	0.5848	16.32
rs2133292	T	C	0.373582	0.0132297	0.00206557	1.50E-10	-0.0148	0.0245	0.546799	19.20
rs2133561	T	A	0.611067	-0.0181895	0.00206386	1.20E-18	0.0223	0.0236	0.346	36.92
rs2151248	C	G	0.730659	-0.0149326	0.00224027	2.60E-11	-0.0041	0.0258	0.8723	17.49
rs2159437	G	A	0.517573	0.0196241	0.00199751	8.90E-23	-0.0097	0.0233	0.677901	48.20
rs2172131	C	T	0.578698	-0.0136895	0.00202029	1.20E-11	-0.0077	0.0227	0.7335	22.39

SNP	effect_allele HC	other_alleleHC	eaf. HC	beta.HC	se.HC	pval.HC	beta. II	se. II	pval. II	F-statistics
rs2178385	T	G	0.320337	0.0185474	0.00213295	3.40E-18	-0.0389	0.0249	0.1184	32.93
rs2238435	G	C	0.613709	0.0259551	0.00205177	1.10E-36	0.0153	0.023	0.5046	75.89
rs2238689	C	T	0.399438	-0.0141655	0.00203741	3.60E-12	-0.0109	0.0228	0.6326	23.19
rs2253310	G	C	0.626098	0.019536	0.00205623	2.10E-21	0.0183	0.023	0.4253	42.27
rs2270894	G	C	0.203219	-0.0216431	0.00256793	3.50E-17	0.0011	0.0258	0.9673	23.01
rs2288745	T	C	0.299451	0.0136628	0.0021768	3.50E-10	0.0085	0.0234	0.7166	16.53
rs2307111	C	T	0.394977	-0.0285352	0.00203828	1.60E-44	-0.024	0.023	0.2951	93.69
rs2371767	C	G	0.277705	0.0194135	0.00221849	2.10E-18	0.0329	0.0273	0.2284	30.72
rs2371911	A	T	0.544326	0.0131057	0.0019989	5.50E-11	0.0233	0.0228	0.3064	21.33
rs2384054	C	T	0.489428	0.0219057	0.0019869	2.90E-28	-0.0024	0.0229	0.9183	60.76
rs2479958	G	A	0.516982	-0.0129914	0.00201927	1.20E-10	0.0194	0.0228	0.3931	20.67
rs2494196	A	C	0.286135	0.0312737	0.00219937	6.90E-46	-0.0265	0.0248	0.2854	82.61
rs2499468	A	C	0.651137	0.0131826	0.00209013	2.80E-10	-0.0047	0.0256	0.855	18.07
rs253444	A	G	0.12923	-0.0198245	0.00297188	2.50E-11	-0.0107	0.029	0.7133	10.01
rs2568958	A	G	0.603693	0.0206705	0.00202892	2.20E-24	0.0475	0.0237	0.04489	49.67
rs2577955	T	C	0.802767	-0.014667	0.00250536	4.80E-09	-0.0065	0.0304	0.8298	10.85
rs25849	G	C	0.288829	0.019767	0.00220822	3.50E-19	-0.0057	0.0235	0.8082	32.92
rs2585526	G	A	0.557413	0.0122667	0.00201131	1.10E-09	-0.0094	0.0227	0.6799	18.35
rs2593169	C	G	0.567965	-0.0116309	0.00201784	8.20E-09	-0.0161	0.0229	0.4812	16.31
rs2606227	C	T	0.63145	-0.0124349	0.00208271	2.40E-09	-0.0046	0.023	0.8417	16.59
rs2642305	T	A	0.369566	0.0113043	0.00206693	4.50E-08	0.0623	0.0265	0.0187599	13.94
rs2660241	C	T	0.364912	0.0139138	0.00207299	1.90E-11	-5.00E-04	0.0237	0.9823	20.88
rs2678204	G	T	0.340172	0.0196748	0.00209913	7.10E-21	0.0067	0.0248	0.7865	39.44
rs273505	C	T	0.422021	0.0128735	0.00202114	1.90E-10	-0.004	0.0226	0.8601	19.79

SNP	effect_allele HC	other_alleleHC	eaf. HC	beta.HC	se.HC	pval.HC	beta. II	se. II	pval. II	F-statistics
rs2737263	T	G	0.28052	-0.0236589	0.00222017	1.60E-26	0.0463	0.0245	0.0584696	45.84
rs2744956	C	T	0.139254	0.0537577	0.00287012	2.80E-78	0.0353	0.0284	0.2141	84.11
rs2814350	A	G	0.304993	0.0138717	0.00220875	3.40E-10	-0.0162	0.0234	0.4898	16.72
rs2821226	G	A	0.527763	0.015138	0.00200431	4.30E-14	-0.0157	0.0228	0.490999	28.44
rs28366156	C	T	0.130582	-0.0265761	0.00295158	2.20E-19	0.0046	0.0443	0.9176	18.41
rs2861685	C	T	0.411983	-0.0147772	0.0020154	2.30E-13	0.0486	0.023	0.0342799	26.05
rs28711392	C	T	0.367255	-0.0125224	0.00208379	1.90E-09	0.0289	0.0228	0.2042	16.78
rs28778940	A	G	0.330838	0.0151514	0.00212367	9.70E-13	-0.0111	0.0241	0.644401	22.54
rs287837	G	A	0.523844	-0.013078	0.00200242	6.50E-11	-0.004	0.0227	0.8618	21.28
rs2897968	A	G	0.607035	0.0122759	0.00204428	1.90E-09	-0.0279	0.0231	0.2261	17.20
rs3012053	G	A	0.714878	-0.0149707	0.00220858	1.20E-11	-0.01	0.024	0.6765	18.73
rs308911	G	A	0.714423	-0.0142761	0.00220578	9.70E-11	-0.006	0.0252	0.8126	17.09
rs310796	T	G	0.680961	0.0121086	0.00214394	1.60E-08	0.0309	0.0258	0.2307	13.86
rs3110942	A	G	0.481664	0.0127123	0.00199815	2.00E-10	0.0216	0.0228	0.343	20.21
rs3116600	G	A	0.215319	-0.0187688	0.00242539	1.00E-14	0.012	0.0259	0.6443	20.24
rs314288	C	T	0.886256	-0.0227112	0.00313819	4.60E-13	-0.0373	0.0344	0.2792	10.56
rs329118	T	C	0.419386	-0.0163479	0.00201987	5.80E-16	0.0025	0.023	0.9141	31.90
rs33503	A	G	0.80589	-0.017501	0.00251417	3.40E-12	-0.0308	0.0289	0.2872	15.16
rs340025	C	T	0.57994	0.0127406	0.00202809	3.30E-10	0.0083	0.0244	0.7331	19.23
rs34223321	A	C	0.300092	0.0140924	0.00217285	8.80E-11	-0.0068	0.0237	0.7733	17.67
rs34517439	A	C	0.121795	0.0454548	0.00307552	2.00E-49	2.00E-04	0.0342	0.9954	46.73
rs34594435	T	C	0.195585	0.0215785	0.00251186	8.60E-18	-0.0565	0.0296	0.0563806	23.22
rs34629844	G	A	0.12828	0.0212952	0.00297607	8.30E-13	-0.0163	0.0398	0.6817	11.45
rs34656389	G	A	0.367473	0.011297	0.00206647	4.60E-08	0.0417	0.0227	0.0659098	13.89

SNP	effect_allele HC	other_alleleHC	eaf. HC	beta.HC	se.HC	pval.HC	beta. II	se. II	pval. II	F-statistics
rs34765854	G	T	0.298258	0.0193318	0.00217163	5.50E-19	9.00E-04	0.024	0.9692	33.17
rs34769775	T	C	0.29735	-0.0141127	0.00218344	1.00E-10	-0.0114	0.0257	0.6565	17.46
rs34772064	G	T	0.555918	-0.0118736	0.00200309	3.10E-09	-0.0286	0.0226	0.206	17.35
rs34811474	A	G	0.230735	-0.0197929	0.00236244	5.40E-17	-0.0044	0.0269	0.869	24.92
rs34840745	T	C	0.262486	0.0166433	0.00227207	2.40E-13	-0.0235	0.0252	0.3514	20.78
rs34848742	G	T	0.788211	-0.0143597	0.00243566	3.70E-09	0.023	0.0295	0.4361	11.60
rs35506085	A	G	0.185085	-0.0204796	0.00258575	2.40E-15	-0.01	0.0301	0.740901	18.92
rs35537311	T	C	0.388607	-0.0147642	0.00205013	6.00E-13	-0.0166	0.0233	0.4751	24.65
rs35792595	A	T	0.297453	0.0137734	0.00219158	3.30E-10	0.0214	0.0257	0.4055	16.51
rs35882248	T	C	0.317213	0.015668	0.00213778	2.30E-13	-0.0257	0.0243	0.2905	23.27
rs35910339	G	C	0.683708	-0.0127042	0.00214217	3.00E-09	0.0043	0.0277	0.8759	15.21
rs35917007	G	A	0.533673	0.0147568	0.00199857	1.50E-13	-0.0256	0.0228	0.2625	27.14
rs35957544	T	G	0.574337	-0.0192606	0.00202041	1.50E-21	-0.0043	0.023	0.8532	44.44
rs36140	C	A	0.635313	0.0148434	0.00207958	9.50E-13	0.0368	0.0237	0.1212	23.61
rs365352	A	G	0.24439	-0.0205385	0.0023126	6.60E-19	-0.0228	0.0291	0.433	29.13
rs3737992	A	G	0.168963	-0.0215132	0.00265121	4.90E-16	-0.016	0.0288	0.579201	18.49
rs3746759	G	T	0.205871	-0.0144809	0.00246759	4.40E-09	0.0523	0.0337	0.1213	11.26
rs3762988	T	C	0.388074	0.0126504	0.0020481	6.50E-10	0.0034	0.0236	0.886	18.12
rs3807566	T	G	0.438301	-0.0149491	0.00201199	1.10E-13	0.0024	0.0233	0.9184	27.18
rs3810291	A	G	0.674991	0.0227348	0.00212876	1.30E-26	0.0262	0.0234	0.2643	50.05
rs3811951	G	A	0.282058	0.0150182	0.00221148	1.10E-11	-0.0278	0.025	0.2674	18.68
rs3814883	T	C	0.482403	0.0250685	0.00200044	5.00E-36	0.0021	0.0229	0.9259	78.43
rs3826408	T	C	0.456783	0.0135888	0.00200083	1.10E-11	0.0016	0.0226	0.943	22.89
rs3845344	T	C	0.391164	0.0138703	0.00203631	9.70E-12	0.0246	0.0229	0.2821	22.10

SNP	effect_allele HC	other_alleleHC	eaf. HC	beta.HC	se.HC	pval.HC	beta. II	se. II	pval. II	F-statistics
rs3935190	A	G	0.536778	-0.0141131	0.00201245	2.30E-12	0.0042	0.0227	0.8519	24.46
rs394608	C	T	0.537697	0.014611	0.00201006	3.60E-13	0.0306	0.0229	0.1818	26.27
rs40071	C	T	0.179489	-0.0174105	0.00260267	2.20E-11	0.0196	0.0278	0.4795	13.18
rs4017425	T	C	0.470189	-0.0118377	0.00199708	3.10E-09	0.0025	0.0233	0.9159	17.51
rs41273794	T	C	0.287079	0.0191445	0.00220216	3.50E-18	-0.0278	0.026	0.2865	30.94
rs4240326	G	A	0.550142	-0.0279318	0.0020006	2.70E-44	0.0329	0.0227	0.1472	96.50
rs4253755	A	G	0.128527	0.0211316	0.00300303	2.00E-12	0.0656	0.0414	0.1126	11.09
rs429343	G	A	0.576577	-0.0121964	0.00201501	1.40E-09	-0.0184	0.0227	0.4174	17.89
rs4310395	G	A	0.442854	0.0122183	0.00200242	1.00E-09	-0.0062	0.0228	0.7854	18.37
rs4467770	A	G	0.731091	0.0158784	0.00225367	1.80E-12	2.00E-04	0.0251	0.9939	19.52
rs4476935	T	C	0.433157	-0.0128732	0.00201066	1.50E-10	8.00E-04	0.0231	0.974	20.13
rs4477562	T	C	0.128661	0.0292203	0.00300396	2.30E-22	-0.002	0.0326	0.9501	21.22
rs4486868	C	T	0.447992	-0.011105	0.00201104	3.40E-08	0.0055	0.0228	0.8112	15.08
rs4567604	T	G	0.193192	-0.0166838	0.00253879	5.00E-11	-0.0326	0.0301	0.2795	13.46
rs4589131	G	T	0.590569	-0.0117346	0.00202078	6.40E-09	-0.0097	0.0229	0.6707	16.31
rs4613074	C	T	0.18516	-0.0170225	0.00256625	3.30E-11	0.0061	0.03	0.8385	13.28
rs463376	A	G	0.490305	0.0119932	0.0019975	1.90E-09	-0.0308	0.0227	0.1751	18.02
rs4678016	T	C	0.366307	0.0115138	0.00206489	2.50E-08	-0.0027	0.0227	0.9066	14.43
rs475390	A	G	0.776432	-0.0140664	0.00238863	3.90E-09	0.0244	0.0274	0.3736	12.04
rs4790292	A	C	0.153689	-0.0261893	0.00277723	4.10E-21	-0.015	0.0302	0.6195	23.13
rs4792716	G	A	0.562173	0.0145605	0.00201188	4.60E-13	-0.0192	0.0231	0.4042	25.79
rs4800490	C	A	0.495235	-0.023172	0.00199562	3.60E-31	-0.0027	0.0227	0.9055	67.42
rs4820346	G	C	0.69295	-0.0130489	0.00217064	1.80E-09	0.001	0.0232	0.966	15.38
rs483465	G	A	0.749331	0.0154934	0.00229726	1.50E-11	-0.008	0.0317	0.8019	17.09

SNP	effect_allele HC	other_alleleHC	eaf. HC	beta.HC	se.HC	pval.HC	beta. II	se. II	pval. II	F-statistics
rs4843158	C	G	0.68516	0.0218462	0.00214891	2.80E-24	0.0119	0.0245	0.6262	44.59
rs4870057	G	A	0.341223	0.0126926	0.00212695	2.40E-09	-6.00E-04	0.0269	0.9833	16.01
rs4909309	C	T	0.393916	-0.0173539	0.00203923	1.70E-17	-0.0031	0.0227	0.892	34.58
rs4962424	A	T	0.326831	0.0155846	0.00212749	2.40E-13	-0.0332	0.0253	0.1889	23.61
rs4963975	A	G	0.244315	-0.0187917	0.00232365	6.10E-16	-0.0094	0.0251	0.7076	24.15
rs4976994	G	A	0.452549	0.0128387	0.00200503	1.50E-10	-0.0081	0.0228	0.723601	20.32
rs4985407	G	A	0.49763	0.0153026	0.00199743	1.80E-14	-0.0084	0.0232	0.716901	29.35
rs522110	G	A	0.555042	0.0181191	0.0020069	1.70E-19	-0.0299	0.0227	0.1883	40.27
rs543874	G	A	0.205218	0.0453641	0.00246182	8.00E-76	-0.0229	0.0295	0.4369	110.79
rs551935	G	T	0.440778	-0.016925	0.00200492	3.10E-17	0.0022	0.0239	0.9254	35.13
rs55726687	A	G	0.209736	0.0218167	0.00244465	4.50E-19	0.0073	0.0277	0.7914	26.40
rs56094641	G	A	0.404596	0.0616325	0.00203052	1.00E-200	-0.0197	0.0229	0.3903	444.31
rs56097510	G	C	0.51052	0.0137125	0.00199793	6.70E-12	0.0219	0.0226	0.3338	23.54
rs56399737	T	C	0.449109	-0.0118202	0.00201255	4.30E-09	0.011	0.0229	0.6308	17.07
rs57222629	C	G	0.311673	0.0138153	0.0021564	1.50E-10	-0.0079	0.0235	0.737801	17.61
rs57235969	T	C	0.554199	0.0124617	0.00199957	4.60E-10	-0.0145	0.0227	0.5221	19.19
rs57636386	C	T	0.08382	-0.0350845	0.0036118	2.60E-22	0.0235	0.0521	0.651	14.49
rs57989773	C	T	0.245008	0.021874	0.00238057	4.00E-20	-0.0077	0.0282	0.7855	31.24
rs58584712	A	G	0.211251	0.0169765	0.0024357	3.20E-12	-0.0311	0.0297	0.2945	16.19
rs58862095	T	C	0.419278	-0.0181051	0.00202425	3.70E-19	0.0136	0.0227	0.5496	38.96
rs59227842	G	A	0.311488	0.0210227	0.00217036	3.40E-22	-0.0071	0.0244	0.7715	40.25
rs60226453	T	C	0.177273	0.0162724	0.00260018	3.90E-10	0.0714	0.0305	0.0190999	11.42
rs6066104	T	C	0.324108	0.0192833	0.00213455	1.70E-19	-0.0186	0.0235	0.4286	35.76

SNP	effect_allele HC	other_alleleHC	eaf. HC	beta.HC	se.HC	pval.HC	beta. II	se. II	pval. II	F-statistics
rs6142059	C	T	0.492562	0.0149966	0.00200021	6.50E-14	-0.0077	0.0227	0.7346	28.10
rs614520	A	G	0.640637	-0.0130274	0.00212026	8.00E-10	0.022	0.0243	0.3649	17.38
rs61941722	A	G	0.188737	0.0155992	0.00256577	1.20E-09	-0.0044	0.0291	0.8789	11.32
rs61969510	C	T	0.278956	0.0128445	0.00224311	1.00E-08	-0.0404	0.0234	0.0847598	13.19
rs61992671	G	A	0.491978	-0.0151753	0.00208557	3.40E-13	0.012	0.023	0.6002	26.47
rs62070648	A	G	0.268727	-0.0199439	0.0022501	7.80E-19	-0.0224	0.0252	0.3736	30.88
rs62107261	C	T	0.04832	-0.0808476	0.00465016	1.10E-67	0.0794	0.0795	0.3179	27.80
rs62301134	C	T	0.263286	-0.013638	0.00226745	1.80E-09	-0.0012	0.0252	0.9619	14.03
rs62396185	C	G	0.259989	-0.0346069	0.00227012	1.80E-52	0.0408	0.0233	0.0793798	89.44
rs62425398	A	C	0.106309	0.0248634	0.00324644	1.90E-14	-0.0532	0.0433	0.2191	11.15
rs62515438	G	T	0.227665	0.0150395	0.00237712	2.50E-10	0.0017	0.0247	0.9459	14.08
rs643499	C	T	0.398022	0.0119575	0.00204369	4.90E-09	0.0619	0.0228	0.00664906	16.41
rs6438656	A	G	0.635867	-0.0119598	0.0020673	7.20E-09	0.0018	0.024	0.9403	15.50
rs6465828	T	G	0.482837	0.0163908	0.00199173	1.90E-16	0.0077	0.0231	0.738501	33.82
rs6545714	A	G	0.60145	-0.0158267	0.00203362	7.10E-15	0.0077	0.023	0.739301	29.04
rs6567160	C	T	0.232716	0.0523537	0.00236068	5.70E-109	1.00E-04	0.0291	0.9975	175.71
rs6575340	A	G	0.636028	0.0184545	0.00207818	6.70E-19	0.0136	0.0235	0.5612	36.51
rs6597975	G	C	0.543875	0.0122921	0.00201118	9.80E-10	-0.0171	0.023	0.4564	18.53
rs6598540	G	A	0.276885	-0.0147876	0.00223088	3.40E-11	0.0043	0.0262	0.8693	17.60
rs6606686	C	G	0.680627	-0.0165692	0.00213651	8.80E-15	0.0316	0.0241	0.1913	26.15
rs66679256	T	C	0.445805	0.0149901	0.00200438	7.50E-14	-0.0059	0.0235	0.8019	27.64
rs6669341	G	A	0.582713	-0.0151291	0.00201544	6.10E-14	-0.025	0.0233	0.2838	27.41
rs667515	C	G	0.386143	-0.0141684	0.00205316	5.20E-12	0.0131	0.0239	0.582801	22.58

SNP	effect_allele HC	other_alleleHC	eaf. HC	beta.HC	se.HC	pval.HC	beta. II	se. II	pval. II	F-statistics
rs6744646	G	A	0.828323	0.0500537	0.00263558	2.00E-80	-0.023	0.0309	0.4562	102.60
rs6745626	T	C	0.589593	0.0139294	0.00202493	6.00E-12	-0.0201	0.0233	0.3886	22.90
rs6747657	A	G	0.281723	0.0129341	0.00220754	4.70E-09	-0.0478	0.0255	0.0611801	13.89
rs675162	G	A	0.482059	0.0180079	0.00199495	1.80E-19	0.0038	0.0226	0.8681	40.69
rs6840236	C	T	0.464856	0.0169824	0.00199864	1.90E-17	0.0039	0.0227	0.8649	35.92
rs6868125	T	C	0.521906	-0.0119493	0.00198836	1.90E-09	0.0515	0.0231	0.0258899	18.02
rs6946415	G	A	0.627099	0.0158826	0.00206409	1.40E-14	0.013	0.0241	0.5882	27.69
rs6958365	T	C	0.411809	-0.0115375	0.00203582	1.50E-08	-0.0294	0.0237	0.2144	15.56
rs6973700	G	A	0.20616	-0.0157823	0.00246577	1.50E-10	-0.0242	0.0255	0.3408	13.41
rs698147	G	A	0.543563	-0.0121448	0.00200109	1.30E-09	-0.0039	0.0226	0.8615	18.28
rs7002088	C	A	0.445199	0.0137173	0.00200556	7.90E-12	0.0399	0.0261	0.1259	23.11
rs7034554	G	A	0.373815	-0.012422	0.0020595	1.60E-09	-0.0259	0.025	0.3019	17.03
rs7038943	C	T	0.338791	-0.0121087	0.00210372	8.60E-09	-0.0234	0.0232	0.3123	14.84
rs705165	T	G	0.247646	0.0148091	0.00230698	1.40E-10	-0.0031	0.0267	0.9084	15.36
rs7094073	T	C	0.191266	0.0163482	0.00273496	2.30E-09	-0.0473	0.0327	0.1482	11.05
rs7111235	C	T	0.493399	0.0115415	0.00200364	8.40E-09	0.0217	0.0238	0.3612	16.59
rs7124681	A	C	0.408387	0.0205032	0.00202231	3.70E-24	-0.0598	0.0237	0.0116001	49.67
rs7132908	A	G	0.384469	0.0257741	0.00204901	2.80E-36	0.0147	0.0234	0.5284	74.90
rs71413981	A	G	0.162848	0.021194	0.00271023	5.30E-15	-0.0357	0.0329	0.2779	16.67
rs7171864	A	G	0.660205	0.0161986	0.00211572	1.90E-14	-0.0225	0.0249	0.3663	26.30
rs7218014	C	T	0.197308	0.0207669	0.00251132	1.30E-16	0.055	0.0266	0.0384902	21.66
rs7248205	T	C	0.600262	-0.0161199	0.00204315	3.00E-15	0.0015	0.0232	0.9486	29.87
rs7257083	A	G	0.288605	0.0162612	0.0022051	1.70E-13	0.0263	0.0289	0.3628	22.33
rs72634826	A	G	0.259879	-0.0153317	0.00229923	2.60E-11	0.0072	0.0278	0.7958	17.11

SNP	effect_allele HC	other_alleleHC	eaf. HC	beta.HC	se.HC	pval.HC	beta. II	se. II	pval. II	F-statistics
rs72656010	C	T	0.132181	-0.0252479	0.00295341	1.20E-17	-0.0018	0.0341	0.9572	16.77
rs7274811	T	G	0.258589	-0.01951	0.00227858	1.10E-17	-0.0101	0.0269	0.706601	28.11
rs72756476	C	T	0.141428	-0.0192477	0.00287344	2.10E-11	0.0459	0.0369	0.2138	10.90
rs72959041	A	G	0.049137	-0.0662534	0.00466657	9.50E-46	-0.0227	0.0464	0.6243	18.84
rs73052033	C	T	0.184918	-0.0208103	0.00256783	5.30E-16	0.038	0.0308	0.2168	19.80
rs73175572	G	A	0.111726	0.0282075	0.00319248	1.00E-18	0.0281	0.0354	0.4268	15.50
rs73213484	T	A	0.141217	-0.0207006	0.00286036	4.60E-13	-0.0439	0.032	0.1693	12.70
rs7365	G	A	0.482654	-0.0110939	0.00199385	2.60E-08	-0.0091	0.0227	0.6883	15.46
rs7372674	A	C	0.357244	0.014356	0.00207575	4.60E-12	0.0133	0.0246	0.5903	21.97
rs743572	G	A	0.376473	0.0151185	0.00205743	2.00E-13	-0.0275	0.0233	0.2379	25.35
rs7442885	G	C	0.214027	-0.0198682	0.00243204	3.10E-16	-0.031	0.0291	0.287	22.45
rs7460093	A	G	0.53118	0.0136639	0.0020144	1.20E-11	0.0151	0.0229	0.510199	22.92
rs74749286	A	G	0.107637	0.0258963	0.00323591	1.20E-15	0.068	0.0269	0.01163	12.30
rs7498665	G	A	0.399666	0.0317857	0.00203647	6.40E-55	0.0029	0.023	0.8999	116.93
rs750090	C	T	0.35675	-0.0132955	0.00210095	2.50E-10	7.00E-04	0.023	0.976	18.38
rs7519259	A	G	0.528388	0.0141441	0.00200058	1.50E-12	0.0087	0.0228	0.7021	24.91
rs7523668	A	G	0.565827	-0.0137199	0.00200928	8.60E-12	0.0209	0.0227	0.357	22.91
rs756717	A	G	0.399077	-0.0140302	0.00206015	9.70E-12	-0.0139	0.0232	0.5485	22.25
rs7570446	A	C	0.544459	0.0110158	0.00199439	3.30E-08	0.0618	0.0228	0.00672496	15.13
rs7571496	G	A	0.260562	-0.0138156	0.00227445	1.20E-09	-0.0217	0.0232	0.351	14.22
rs7582359	A	G	0.331712	-0.0135492	0.00212104	1.70E-10	0.0283	0.0242	0.2433	18.09
rs7632381	C	T	0.444325	0.0270429	0.00200044	1.20E-41	-0.0025	0.0228	0.9134	90.26
rs765874	A	T	0.489353	-0.0168012	0.0019903	3.10E-17	-3.00E-04	0.0231	0.9885	35.62
rs76647086	T	G	0.176857	-0.0202229	0.00260327	8.00E-15	-0.0015	0.0298	0.9591	17.57

SNP	effect_allele HC	other_alleleHC	eaf. HC	beta.HC	se.HC	pval.HC	beta. II	se. II	pval. II	F-statistics
rs76798800	T	G	0.266025	0.0244232	0.00225319	2.20E-27	-0.0138	0.0268	0.6077	45.89
rs7695177	G	C	0.470976	-0.0151527	0.00199491	3.10E-14	-0.0109	0.0227	0.6316	28.75
rs7696175	C	T	0.526478	0.0136068	0.00200383	1.10E-11	-0.0469	0.0228	0.0400498	22.99
rs7707394	A	G	0.357269	-0.0184703	0.002075	5.50E-19	-0.0095	0.0234	0.6851	36.39
rs7708584	G	A	0.572378	-0.013196	0.00201087	5.30E-11	0.0088	0.0231	0.702901	21.08
rs7740107	A	T	0.736289	-0.0236486	0.00225676	1.10E-25	0.0251	0.0256	0.3273	42.65
rs7793674	C	A	0.144347	0.019239	0.00284373	1.30E-11	0.1203	0.0967	0.2132	11.31
rs7793674	C	A	0.144347	0.019239	0.00284373	1.30E-11	0.0274	0.0436	0.5294	11.31
rs779655	C	G	0.729184	0.0143261	0.00224052	1.60E-10	-0.0392	0.0241	0.1043	16.15
rs7805441	T	C	0.502255	0.0126844	0.00200504	2.50E-10	0.0055	0.0226	0.8076	20.01
rs7864465	A	G	0.559379	-0.0110794	0.00200561	3.30E-08	-0.0408	0.023	0.0753494	15.04
rs7893571	T	G	0.665871	0.0138381	0.00211863	6.50E-11	0.0536	0.0247	0.0298003	18.98
rs7903146	T	C	0.290662	-0.0214133	0.00219381	1.70E-22	-0.0109	0.0284	0.7011	39.29
rs7944782	G	T	0.50981	0.0129576	0.0020033	9.90E-11	0.0219	0.0228	0.3372	20.91
rs7952436	T	C	0.081996	-0.0354571	0.00363182	1.60E-22	0.0352	0.0509	0.4897	14.35
rs7996639	A	G	0.449371	0.0137138	0.00201868	1.10E-11	0.001	0.0227	0.9643	22.84
rs8030456	T	C	0.226311	-0.0247721	0.00237863	2.10E-25	-0.041	0.0308	0.1828	37.98
rs8132129	T	C	0.184901	-0.015922	0.00259474	8.40E-10	-0.0472	0.0277	0.0883507	11.35
rs8133137	G	A	0.664097	0.0157777	0.00211541	8.80E-14	0.0045	0.0229	0.8455	24.82
rs815163	C	T	0.563175	-0.0168978	0.00200258	3.20E-17	0.0154	0.0227	0.4985	35.03
rs852042	G	A	0.758582	-0.0153695	0.00233046	4.30E-11	-0.0128	0.0267	0.6327	15.93
rs852983	A	G	0.459576	-0.0118657	0.00199745	2.80E-09	-0.0166	0.0227	0.4641	17.53
rs866006	G	T	0.618508	-0.0120595	0.00204995	4.00E-09	-0.0182	0.023	0.4298	16.33
rs897186	G	A	0.548592	-0.0160621	0.00199856	9.20E-16	0.0251	0.0229	0.2722	31.99

SNP	effect_allele HC	other_alleleHC	eaf. HC	beta.HC	se.HC	pval.HC	beta. II	se. II	pval. II	F-statistics
rs9284814	A	G	0.884815	0.0230203	0.00311527	1.50E-13	0.0356	0.0355	0.3158	11.13
rs9378684	T	C	0.200589	0.018067	0.00250737	5.80E-13	-0.0714	0.0253	0.00484105	16.65
rs9385385	T	C	0.447856	0.012816	0.00201537	2.00E-10	0.0031	0.0228	0.8911	20.00
rs9512696	G	A	0.66154	0.0172114	0.00211114	3.60E-16	-0.015	0.024	0.533301	29.77
rs9513018	T	G	0.616147	-0.0112355	0.00205443	4.50E-08	-0.0013	0.0232	0.9537	14.15
rs968379	T	C	0.229098	-0.0187833	0.00237244	2.40E-15	0.0045	0.027	0.868	22.14
rs9764678	C	T	0.272455	0.0161285	0.00225012	7.60E-13	0.0202	0.0242	0.4048	20.37
rs9788550	C	G	0.247466	-0.0214137	0.00232004	2.70E-20	-0.0203	0.0238	0.3938	31.73
rs9830592	A	C	0.582425	0.0138364	0.00201841	7.10E-12	0.0234	0.0235	0.3199	22.86
rs9843653	C	T	0.511657	0.0183267	0.00199203	3.60E-20	-0.0119	0.0232	0.6072	42.30
rs9845755	T	A	0.198555	0.027361	0.0024977	6.30E-28	-0.005	0.0299	0.8671	38.19
rs9850529	A	G	0.339796	0.0114818	0.00209898	4.50E-08	-0.0065	0.0252	0.7976	13.43
rs987237	G	A	0.17957	0.0344542	0.00259141	2.50E-40	0.0387	0.0276	0.1603	52.09
rs9876664	T	G	0.3754	-0.0143788	0.00205893	2.90E-12	-0.004	0.0228	0.8626	22.87
rs9951619	G	T	0.767352	0.0138525	0.00237634	5.60E-09	0.0069	0.0241	0.776401	12.13
rs9967287	T	G	0.251722	0.0141454	0.00230082	7.80E-10	-0.0272	0.0233	0.2433	14.24
rs9967367	T	C	0.293494	-0.0174208	0.00220445	2.70E-15	0.0313	0.0238	0.1888	25.90
rs998584	A	C	0.482802	-0.0222736	0.00199467	5.90E-29	-0.0265	0.0228	0.2443	62.28

Abbreviations: SNP, single nucleotide polymorphism; se, standard error; HC, hip circumference; II, intestinal infections; pval, p-value.

Table S13. Detailed information about single-nucleotide polymorphisms of hip circumference on infections of the skin and subcutaneous tissue.

SNP	effect_allele HC	other_alleleHC	eaf. HC	beta.HC	se.HC	pval.HC	beta. SSTI	se. SSTI	pval. SSTI	F-statistics
rs1006399	A	G	0.458873	-0.0114857	0.00200117	9.50E-09	-0.0141	0.0149	0.3462	16.36
rs10100245	A	G	0.564506	0.0205386	0.0020121	1.80E-24	-0.0039	0.0147	0.7925	51.24
rs10118701	G	A	0.32209	0.0168068	0.00213097	3.10E-15	0.0137	0.0151	0.3615	27.17
rs10132514	T	C	0.273182	-0.0131415	0.00225463	5.60E-09	-0.0323	0.0179	0.0713806	13.49
rs1013402	G	A	0.318427	0.0293703	0.002136	5.10E-43	0.0066	0.0155	0.672201	82.08
rs10145154	T	C	0.22178	0.0253835	0.00240401	4.60E-26	0.0042	0.0172	0.8084	38.49
rs10172196	A	G	0.305442	0.0174435	0.00216332	7.40E-16	-2.00E-04	0.0156	0.992	27.59
rs10210468	C	T	0.464304	-0.0137575	0.00201939	9.60E-12	0.0131	0.0148	0.3748	23.09
rs1023767	A	G	0.235192	-0.015354	0.00234795	6.20E-11	-0.019	0.0152	0.212	15.38
rs1037702	A	G	0.62178	-0.0116182	0.00205972	1.70E-08	-0.0198	0.0151	0.1907	14.97
rs10404726	T	C	0.465501	-0.0166459	0.00200329	9.60E-17	-0.0091	0.0147	0.5346	34.36
rs10407871	C	T	0.155426	-0.0181966	0.00276505	4.70E-11	-0.0133	0.0195	0.4946	11.37
rs10471636	A	G	0.508884	-0.0118317	0.00203156	5.70E-09	0.0104	0.0148	0.481	16.95
rs1047891	A	C	0.315753	0.0166025	0.00213661	7.80E-15	0.0105	0.0158	0.5053	26.09
rs1056720	T	C	0.234833	-0.0130647	0.00235122	2.80E-08	-0.0193	0.0184	0.2956	11.10
rs10744145	A	C	0.513795	-0.0121183	0.00199668	1.30E-09	0.0208	0.0147	0.1577	18.40
rs10746833	G	A	0.581617	-0.0149421	0.0020362	2.20E-13	0.0196	0.0148	0.185	26.21
rs10773051	T	C	0.222167	0.0231417	0.00240285	5.90E-22	-0.0092	0.0162	0.5686	32.06
rs10810598	A	T	0.639535	-0.0129254	0.00207775	4.90E-10	0.0119	0.0153	0.4378	17.84
rs10883553	A	C	0.445855	0.0151046	0.00200686	5.20E-14	-0.0127	0.0149	0.3962	27.99
rs10954284	A	T	0.488124	0.0193137	0.00199245	3.20E-22	0.0177	0.0147	0.2301	46.96
rs10987417	T	G	0.385638	0.0128078	0.00206992	6.10E-10	0.0101	0.0148	0.4974	18.14
rs11030016	T	C	0.739706	0.018143	0.00227588	1.60E-15	-0.0051	0.0157	0.746	24.47

SNP	effect_allele HC	other_alleleHC	eaf. HC	beta.HC	se.HC	pval.HC	beta. SSTI	se. SSTI	pval. SSTI	F-statistics
rs11045163	G	A	0.430597	0.0114512	0.00201589	1.30E-08	0.0106	0.0151	0.4821	15.82
rs11075252	G	A	0.284481	-0.012562	0.00221467	1.40E-08	0.004	0.0157	0.7982	13.10
rs1108548	G	A	0.27725	0.0156522	0.00222734	2.10E-12	0.0102	0.0159	0.519501	19.79
rs11107114	A	G	0.228885	0.0133238	0.00237525	2.00E-08	0.0163	0.0156	0.2985	11.11
rs11113445	G	A	0.391358	0.0127502	0.00203858	4.00E-10	0.0214	0.0152	0.1574	18.64
rs11150461	G	C	0.727351	-0.0141564	0.00224714	3.00E-10	0.015	0.018	0.4048	15.74
rs11164630	T	C	0.608331	-0.0138102	0.00204155	1.30E-11	0.0024	0.0154	0.8772	21.81
rs11165643	T	C	0.590085	0.0185255	0.00202023	4.70E-20	-0.0044	0.015	0.7693	40.68
rs11173522	A	C	0.214329	0.013694	0.00242935	1.70E-08	0.006	0.0186	0.7461	10.70
rs1123295	G	A	0.444369	0.0119544	0.00200534	2.50E-09	0.0107	0.0148	0.4719	17.55
rs11263719	T	C	0.469657	0.0114989	0.00200822	1.00E-08	-0.0158	0.015	0.2925	16.33
rs112875651	A	G	0.391041	0.0182659	0.00206839	1.00E-18	-0.0033	0.0151	0.8266	37.14
rs113230003	A	G	0.260523	-0.014859	0.00229209	9.00E-11	0.0065	0.018	0.7173	16.19
rs11664106	T	A	0.37403	0.016775	0.00211301	2.00E-15	-0.0153	0.0158	0.3337	29.51
rs11704728	T	C	0.196356	0.0148323	0.00252373	4.20E-09	0.028	0.0179	0.1177	10.90
rs11751684	T	G	0.275389	0.0167319	0.0022233	5.20E-14	-0.0167	0.0191	0.3811	22.60
rs11778934	G	C	0.536083	-0.0141306	0.00200504	1.80E-12	0.0041	0.0154	0.7896	24.71
rs11779446	G	A	0.161259	-0.020178	0.00271964	1.20E-13	0.0209	0.021	0.3203	14.89
rs1182199	A	C	0.304395	-0.0246541	0.00216674	5.40E-30	-0.0214	0.0153	0.1615	54.83
rs11882796	T	A	0.541285	-0.0113842	0.00200309	1.30E-08	5.00E-04	0.0148	0.9747	16.04
rs11997077	G	A	0.380458	-0.0112873	0.00206597	4.70E-08	-0.0142	0.0148	0.3375	14.07
rs12072739	G	A	0.224461	0.0149904	0.00238686	3.40E-10	0.0251	0.0158	0.112	13.73
rs12122361	G	A	0.265617	-0.013773	0.00226034	1.10E-09	-0.003	0.016	0.8515	14.49
rs12128526	A	G	0.457416	0.0113684	0.00199597	1.20E-08	-0.0096	0.0147	0.5153	16.10

SNP	effect_allele HC	other_alleleHC	eaf. HC	beta.HC	se.HC	pval.HC	beta. SSTI	se. SSTI	pval. SSTI	F-statistics
rs12364470	G	T	0.164552	0.0178805	0.0026871	2.80E-11	-0.0145	0.0176	0.4112	12.17
rs12375196	A	C	0.424347	0.0167592	0.00202832	1.40E-16	0.006	0.0148	0.6864	33.36
rs12427047	T	C	0.24273	-0.0163996	0.0023233	1.70E-12	-0.0211	0.0155	0.1748	18.32
rs12441543	A	G	0.2871	0.0152338	0.0022089	5.30E-12	-6.00E-04	0.0163	0.9708	19.47
rs12462975	A	G	0.329669	0.0189074	0.00213714	9.00E-19	8.00E-04	0.0158	0.9619	34.60
rs12519997	A	G	0.559278	-0.0139984	0.00200434	2.90E-12	0.0091	0.0153	0.554501	24.05
rs12568411	A	G	0.166749	0.0164813	0.00269359	9.40E-10	0.0122	0.0175	0.4864	10.40
rs12569355	G	A	0.119536	0.0217444	0.00307709	1.60E-12	0.0193	0.0294	0.5126	10.51
rs1260326	C	T	0.604267	0.0120369	0.00203008	3.00E-09	-0.0051	0.0154	0.738599	16.81
rs12607512	G	A	0.44632	0.0117842	0.00200813	4.40E-09	0.0127	0.0147	0.3866	17.02
rs12631813	G	C	0.50262	-0.0122712	0.00200027	8.50E-10	-0.0075	0.0147	0.6093	18.82
rs12762744	T	C	0.248261	0.0133166	0.00231189	8.40E-09	0.0253	0.0162	0.1181	12.38
rs1285992	G	A	0.710447	0.0203155	0.00220302	2.90E-20	0.0167	0.0167	0.3178	34.99
rs12883788	T	C	0.459801	0.0143864	0.00200867	7.90E-13	-0.0078	0.0149	0.6026	25.48
rs12921916	C	T	0.28756	0.0125955	0.00222773	1.60E-08	0.0047	0.0168	0.7782	13.10
rs12939848	T	C	0.401034	0.0126399	0.00203785	5.60E-10	-0.018	0.0151	0.2343	18.48
rs1294438	T	C	0.354583	0.0186269	0.00210751	9.70E-19	0.0138	0.0154	0.3697	35.76
rs1296328	C	A	0.559033	-0.015815	0.00201508	4.20E-15	-0.0255	0.0147	0.0829507	30.37
rs13017207	A	G	0.393634	-0.0161548	0.00203426	2.00E-15	0.0095	0.015	0.527499	30.11
rs13107325	T	C	0.074895	0.0386647	0.0037857	1.70E-24	0.0402	0.0619	0.5158	14.46
rs13148263	A	G	0.335162	0.0159436	0.00210506	3.60E-14	0.0181	0.0148	0.2213	25.57
rs13156484	A	G	0.472171	-0.0174918	0.00201454	3.90E-18	-0.0102	0.0147	0.4896	37.58
rs13284988	C	T	0.305411	0.0120763	0.00217861	3.00E-08	0.0234	0.0167	0.1607	13.04
rs13292699	C	A	0.433708	-0.0162732	0.00201482	6.70E-16	0.0023	0.0147	0.8776	32.05

SNP	effect_allele HC	other_alleleHC	eaf. HC	beta.HC	se.HC	pval.HC	beta. SSTI	se. SSTI	pval. SSTI	F-statistics
rs133015	G	C	0.439948	0.0129624	0.00201566	1.30E-10	0.0045	0.0149	0.7621	20.38
rs13333747	C	T	0.182674	-0.0219722	0.00259208	2.30E-17	-5.00E-04	0.0192	0.9778	21.46
rs13389219	T	C	0.392395	0.0227595	0.00203433	4.70E-29	-0.0025	0.0154	0.8696	59.69
rs143384	G	A	0.404382	0.0267503	0.00203131	1.30E-39	-0.0227	0.0148	0.127	83.55
rs1446585	G	A	0.244506	-0.0128503	0.00226761	1.50E-08	-0.0066	0.0154	0.668499	11.86
rs1452082	A	C	0.557071	0.012586	0.00203982	6.80E-10	-0.0192	0.015	0.2025	18.79
rs1458156	T	C	0.488404	0.0126488	0.00199454	2.30E-10	0.0118	0.0148	0.4237	20.10
rs1477290	C	T	0.136945	0.0298633	0.00292146	1.60E-24	-0.0387	0.0225	0.0855697	24.70
rs147730268	T	G	0.087233	-0.0506092	0.00361111	1.30E-44	-0.0169	0.0266	0.5251	31.28
rs1480474	G	A	0.417444	-0.0145096	0.00202337	7.40E-13	0.0058	0.0151	0.699401	25.01
rs1481630	C	T	0.176871	-0.0153923	0.00261541	4.00E-09	-0.0032	0.0209	0.8765	10.09
rs1502317	T	C	0.27657	-0.0151583	0.00223176	1.10E-11	-0.0196	0.0167	0.2392	18.46
rs1514895	G	A	0.285289	0.0174697	0.00220175	2.10E-15	0.0044	0.0166	0.793299	25.67
rs1538535	T	C	0.27456	0.0124387	0.0022369	2.70E-08	0.0324	0.0178	0.06868	12.32
rs1576655	C	A	0.59598	0.0161325	0.00207546	7.70E-15	0.0121	0.0157	0.4421	29.10
rs1609783	A	G	0.525344	0.0142717	0.0020044	1.10E-12	-0.0047	0.0147	0.751299	25.28
rs16868443	C	G	0.360418	0.0154349	0.0020756	1.00E-13	0.0131	0.0167	0.4329	25.50
rs170016	A	G	0.633948	-0.0116498	0.00209333	2.60E-08	-0.0198	0.0157	0.2076	14.37
rs17361789	G	T	0.32182	0.0126424	0.00214788	4.00E-09	0.0149	0.0166	0.3681	15.12
rs17639546	A	G	0.14849	-0.0187703	0.0028004	2.00E-11	-0.0109	0.0213	0.6073	11.36
rs17733217	G	A	0.229127	-0.0158057	0.00237935	3.10E-11	0.0099	0.0191	0.6055	15.59
rs17766836	T	C	0.267888	0.0232323	0.00225241	6.10E-25	0.0077	0.0169	0.6501	41.73
rs17770336	T	C	0.322443	0.0191461	0.00212912	2.40E-19	0.0167	0.0152	0.2723	35.34
rs1813212	G	A	0.445576	-0.0130082	0.00200772	9.20E-11	0.0073	0.0149	0.6264	20.74

SNP	effect_allele HC	other_alleleHC	eaf. HC	beta.HC	se.HC	pval.HC	beta. SSTI	se. SSTI	pval. SSTI	F-statistics
rs1868069	A	G	0.229248	0.0150949	0.00236942	1.90E-10	0.0099	0.0178	0.5771	14.34
rs1934394	C	G	0.228157	0.0129993	0.00238257	4.90E-08	0.0231	0.0177	0.192	10.48
rs1955695	G	A	0.62227	-0.0171244	0.00205855	8.90E-17	-0.0192	0.0162	0.2368	32.53
rs2022050	A	G	0.160924	-0.0208767	0.0027204	1.70E-14	0.013	0.0178	0.4635	15.90
rs2023211	C	T	0.232254	0.0160386	0.00236158	1.10E-11	-0.0071	0.0211	0.737099	16.45
rs2032251	A	T	0.507961	0.0114047	0.00199809	1.10E-08	-0.0118	0.0152	0.4376	16.29
rs211434	A	G	0.681996	0.0131339	0.00214133	8.60E-10	-0.0194	0.0156	0.2113	16.32
rs2133292	T	C	0.373582	0.0132297	0.00206557	1.50E-10	-0.0052	0.0159	0.745501	19.20
rs2159437	G	A	0.517573	0.0196241	0.00199751	8.90E-23	0.0094	0.0151	0.535	48.20
rs2172131	C	T	0.578698	-0.0136895	0.00202029	1.20E-11	0.0186	0.0147	0.2049	22.39
rs2178385	T	G	0.320337	0.0185474	0.00213295	3.40E-18	-0.0219	0.0162	0.1763	32.93
rs2238435	G	C	0.613709	0.0259551	0.00205177	1.10E-36	-0.0134	0.0149	0.3695	75.89
rs2238689	C	T	0.399438	-0.0141655	0.00203741	3.60E-12	-0.0208	0.0148	0.1596	23.19
rs2253310	G	C	0.626098	0.019536	0.00205623	2.10E-21	0.0156	0.0149	0.2961	42.27
rs2270894	G	C	0.203219	-0.0216431	0.00256793	3.50E-17	-0.0208	0.0167	0.2152	23.01
rs2288745	T	C	0.299451	0.0136628	0.0021768	3.50E-10	0.0025	0.0152	0.8699	16.53
rs2307111	C	T	0.394977	-0.0285352	0.00203828	1.60E-44	-0.0291	0.0149	0.0502296	93.69
rs2371767	C	G	0.277705	0.0194135	0.00221849	2.10E-18	0.0342	0.0177	0.0536599	30.72
rs2371911	A	T	0.544326	0.0131057	0.0019989	5.50E-11	0.0204	0.0148	0.1671	21.33
rs2384054	C	T	0.489428	0.0219057	0.0019869	2.90E-28	0.0157	0.0149	0.2911	60.76
rs2479958	G	A	0.516982	-0.0129914	0.00201927	1.20E-10	0.0083	0.0148	0.5721	20.67
rs2494196	A	C	0.286135	0.0312737	0.00219937	6.90E-46	-0.0071	0.0161	0.6579	82.61
rs2499468	A	C	0.651137	0.0131826	0.00209013	2.80E-10	0.0076	0.0167	0.6482	18.07
rs2568958	A	G	0.603693	0.0206705	0.00202892	2.20E-24	0.0034	0.0154	0.8246	49.67

SNP	effect_allele HC	other_alleleHC	eaf. HC	beta.HC	se.HC	pval.HC	beta. SSTI	se. SSTI	pval. SSTI	F-statistics
rs2577955	T	C	0.802767	-0.014667	0.00250536	4.80E-09	0.0083	0.0198	0.6761	10.85
rs25849	G	C	0.288829	0.019767	0.00220822	3.50E-19	-0.0242	0.0153	0.1124	32.92
rs2585526	G	A	0.557413	0.0122667	0.00201131	1.10E-09	0.0184	0.0148	0.2117	18.35
rs2593169	C	G	0.567965	-0.0116309	0.00201784	8.20E-09	0.0157	0.0148	0.2891	16.31
rs2606227	C	T	0.63145	-0.0124349	0.00208271	2.40E-09	-0.0213	0.0149	0.1532	16.59
rs2642305	T	A	0.369566	0.0113043	0.00206693	4.50E-08	0.0088	0.0171	0.6062	13.94
rs2660241	C	T	0.364912	0.0139138	0.00207299	1.90E-11	-0.0033	0.0154	0.8312	20.88
rs2678204	G	T	0.340172	0.0196748	0.00209913	7.10E-21	-0.0039	0.0161	0.8107	39.44
rs273505	C	T	0.422021	0.0128735	0.00202114	1.90E-10	0.0054	0.0147	0.713199	19.79
rs2744956	C	T	0.139254	0.0537577	0.00287012	2.80E-78	-0.01	0.0185	0.59	84.11
rs2814350	A	G	0.304993	0.0138717	0.00220875	3.40E-10	0.0211	0.0152	0.1657	16.72
rs2821226	G	A	0.527763	0.015138	0.00200431	4.30E-14	-0.0019	0.0148	0.8987	28.44
rs28366156	C	T	0.130582	-0.0265761	0.00295158	2.20E-19	0.0209	0.0288	0.4682	18.41
rs2861685	C	T	0.411983	-0.0147772	0.0020154	2.30E-13	0.0098	0.0149	0.5108	26.05
rs28711392	C	T	0.367255	-0.0125224	0.00208379	1.90E-09	-0.0027	0.0148	0.8535	16.78
rs28778940	A	G	0.330838	0.0151514	0.00212367	9.70E-13	-0.0198	0.0156	0.2068	22.54
rs287837	G	A	0.523844	-0.013078	0.00200242	6.50E-11	-0.0041	0.0147	0.7817	21.28
rs2897968	A	G	0.607035	0.0122759	0.00204428	1.90E-09	0.0064	0.015	0.6692	17.20
rs3012053	G	A	0.714878	-0.0149707	0.00220858	1.20E-11	0.0294	0.0156	0.0602504	18.73
rs308911	G	A	0.714423	-0.0142761	0.00220578	9.70E-11	-0.0321	0.0164	0.0501499	17.09
rs310796	T	G	0.680961	0.0121086	0.00214394	1.60E-08	0.004	0.0167	0.8099	13.86
rs3116600	G	A	0.215319	-0.0187688	0.00242539	1.00E-14	0.0193	0.0169	0.2538	20.24
rs314288	C	T	0.886256	-0.0227112	0.00313819	4.60E-13	-0.0254	0.0223	0.2548	10.56
rs329118	T	C	0.419386	-0.0163479	0.00201987	5.80E-16	0.0135	0.0149	0.365	31.90

SNP	effect_allele HC	other_alleleHC	eaf. HC	beta.HC	se.HC	pval.HC	beta. SSTI	se. SSTI	pval. SSTI	F-statistics
rs33503	A	G	0.80589	-0.017501	0.00251417	3.40E-12	0.0071	0.0187	0.7057	15.16
rs340025	C	T	0.57994	0.0127406	0.00202809	3.30E-10	-0.0127	0.0158	0.4228	19.23
rs34223321	A	C	0.300092	0.0140924	0.00217285	8.80E-11	-0.0028	0.0154	0.856	17.67
rs34594435	T	C	0.195585	0.0215785	0.00251186	8.60E-18	0.0111	0.0192	0.5636	23.22
rs34629844	G	A	0.12828	0.0212952	0.00297607	8.30E-13	-0.012	0.0258	0.6421	11.45
rs34656389	G	A	0.367473	0.011297	0.00206647	4.60E-08	0.0129	0.0148	0.3838	13.89
rs34769775	T	C	0.29735	-0.0141127	0.00218344	1.00E-10	-0.0035	0.0167	0.8324	17.46
rs34772064	G	T	0.555918	-0.0118736	0.00200309	3.10E-09	0.0027	0.0147	0.853	17.35
rs34840745	T	C	0.262486	0.0166433	0.00227207	2.40E-13	0.0045	0.0164	0.782299	20.78
rs34848742	G	T	0.788211	-0.0143597	0.00243566	3.70E-09	-0.0167	0.0192	0.3826	11.60
rs35506085	A	G	0.185085	-0.0204796	0.00258575	2.40E-15	0.0278	0.0195	0.1533	18.92
rs35537311	T	C	0.388607	-0.0147642	0.00205013	6.00E-13	-0.0065	0.0151	0.6673	24.65
rs35882248	T	C	0.317213	0.015668	0.00213778	2.30E-13	0.0013	0.0158	0.9336	23.27
rs35917007	G	A	0.533673	0.0147568	0.00199857	1.50E-13	0.015	0.0148	0.3104	27.14
rs35957544	T	G	0.574337	-0.0192606	0.00202041	1.50E-21	-0.0274	0.015	0.06718	44.44
rs365352	A	G	0.24439	-0.0205385	0.0023126	6.60E-19	-0.0074	0.0189	0.6951	29.13
rs3737992	A	G	0.168963	-0.0215132	0.00265121	4.90E-16	-0.0218	0.0187	0.2438	18.49
rs3762988	T	C	0.388074	0.0126504	0.0020481	6.50E-10	-0.0048	0.0154	0.754899	18.12
rs3807566	T	G	0.438301	-0.0149491	0.00201199	1.10E-13	-0.0227	0.0152	0.1344	27.18
rs3810291	A	G	0.674991	0.0227348	0.00212876	1.30E-26	-0.0031	0.0152	0.837	50.05
rs3811951	G	A	0.282058	0.0150182	0.00221148	1.10E-11	-0.0051	0.0162	0.755299	18.68
rs3814883	T	C	0.482403	0.0250685	0.00200044	5.00E-36	-0.0059	0.0149	0.6921	78.43
rs3826408	T	C	0.456783	0.0135888	0.00200083	1.10E-11	-0.0192	0.0147	0.193	22.89
rs3935190	A	G	0.536778	-0.0141131	0.00201245	2.30E-12	-0.0205	0.0148	0.1642	24.46

SNP	effect_allele HC	other_alleleHC	eaf. HC	beta.HC	se.HC	pval.HC	beta. SSTI	se. SSTI	pval. SSTI	F-statistics
rs394608	C	T	0.537697	0.014611	0.00201006	3.60E-13	0.0024	0.0148	0.8737	26.27
rs40071	C	T	0.179489	-0.0174105	0.00260267	2.20E-11	-0.0251	0.0181	0.1648	13.18
rs41273794	T	C	0.287079	0.0191445	0.00220216	3.50E-18	-0.0046	0.017	0.7862	30.94
rs4240326	G	A	0.550142	-0.0279318	0.0020006	2.70E-44	0.0056	0.0147	0.7047	96.50
rs4253755	A	G	0.128527	0.0211316	0.00300303	2.00E-12	0.0124	0.0268	0.6423	11.09
rs429343	G	A	0.576577	-0.0121964	0.00201501	1.40E-09	-0.02	0.0148	0.175	17.89
rs4310395	G	A	0.442854	0.0122183	0.00200242	1.00E-09	0.0011	0.0148	0.9419	18.37
rs4467770	A	G	0.731091	0.0158784	0.00225367	1.80E-12	-0.0086	0.0163	0.5989	19.52
rs4476935	T	C	0.433157	-0.0128732	0.00201066	1.50E-10	-0.0078	0.015	0.6058	20.13
rs4477562	T	C	0.128661	0.0292203	0.00300396	2.30E-22	0.0028	0.0213	0.8946	21.22
rs4486868	C	T	0.447992	-0.011105	0.00201104	3.40E-08	0.0054	0.0149	0.7183	15.08
rs4567604	T	G	0.193192	-0.0166838	0.00253879	5.00E-11	0.0062	0.0194	0.7484	13.46
rs4589131	G	T	0.590569	-0.0117346	0.00202078	6.40E-09	0.0048	0.0148	0.744201	16.31
rs4613074	C	T	0.18516	-0.0170225	0.00256625	3.30E-11	0.0045	0.0195	0.8189	13.28
rs463376	A	G	0.490305	0.0119932	0.0019975	1.90E-09	-0.0099	0.0147	0.5018	18.02
rs4678016	T	C	0.366307	0.0115138	0.00206489	2.50E-08	0.0065	0.0148	0.6615	14.43
rs475390	A	G	0.776432	-0.0140664	0.00238863	3.90E-09	0.0143	0.0177	0.4202	12.04
rs4790292	A	C	0.153689	-0.0261893	0.00277723	4.10E-21	-0.0142	0.0196	0.4703	23.13
rs4792716	G	A	0.562173	0.0145605	0.00201188	4.60E-13	0.0255	0.015	0.0885503	25.79
rs4800490	C	A	0.495235	-0.023172	0.00199562	3.60E-31	-0.0224	0.0147	0.1275	67.42
rs483465	G	A	0.749331	0.0154934	0.00229726	1.50E-11	-0.0096	0.0205	0.641	17.09
rs4843158	C	G	0.68516	0.0218462	0.00214891	2.80E-24	0.0021	0.0159	0.894	44.59
rs4870057	G	A	0.341223	0.0126926	0.00212695	2.40E-09	0.0244	0.0175	0.1618	16.01
rs4909309	C	T	0.393916	-0.0173539	0.00203923	1.70E-17	0.0183	0.0147	0.2136	34.58

SNP	effect_allele HC	other_alleleHC	eaf. HC	beta.HC	se.HC	pval.HC	beta. SSTI	se. SSTI	pval. SSTI	F-statistics
rs4962424	A	T	0.326831	0.0155846	0.00212749	2.40E-13	-1.00E-04	0.0164	0.9945	23.61
rs4963975	A	G	0.244315	-0.0187917	0.00232365	6.10E-16	-0.0126	0.0163	0.4388	24.15
rs4976994	G	A	0.452549	0.0128387	0.00200503	1.50E-10	0.0011	0.0148	0.9425	20.32
rs522110	G	A	0.555042	0.0181191	0.0020069	1.70E-19	-0.0157	0.0148	0.288	40.27
rs543874	G	A	0.205218	0.0453641	0.00246182	8.00E-76	0.0093	0.0192	0.6292	110.79
rs551935	G	T	0.440778	-0.016925	0.00200492	3.10E-17	0.0158	0.0155	0.3066	35.13
rs55726687	A	G	0.209736	0.0218167	0.00244465	4.50E-19	0.0304	0.0179	0.0898504	26.40
rs56094641	G	A	0.404596	0.0616325	0.00203052	1.00E-200	0.018	0.0149	0.2271	444.31
rs56097510	G	C	0.51052	0.0137125	0.00199793	6.70E-12	-2.00E-04	0.0147	0.9873	23.54
rs56399737	T	C	0.449109	-0.0118202	0.00201255	4.30E-09	-0.0179	0.0149	0.2283	17.07
rs57222629	C	G	0.311673	0.0138153	0.0021564	1.50E-10	-0.0033	0.0152	0.8266	17.61
rs57636386	C	T	0.08382	-0.0350845	0.0036118	2.60E-22	-0.0569	0.0337	0.0917191	14.49
rs58584712	A	G	0.211251	0.0169765	0.0024357	3.20E-12	0.0082	0.0194	0.672201	16.19
rs58862095	T	C	0.419278	-0.0181051	0.00202425	3.70E-19	-0.0177	0.0147	0.2285	38.96
rs59227842	G	A	0.311488	0.0210227	0.00217036	3.40E-22	0.0146	0.0159	0.3582	40.25
rs60226453	T	C	0.177273	0.0162724	0.00260018	3.90E-10	0.0031	0.0198	0.8775	11.42
rs6066104	T	C	0.324108	0.0192833	0.00213455	1.70E-19	0.0033	0.0153	0.8278	35.76
rs6142059	C	T	0.492562	0.0149966	0.00200021	6.50E-14	-0.0055	0.0147	0.7072	28.10
rs614520	A	G	0.640637	-0.0130274	0.00212026	8.00E-10	-0.0306	0.0157	0.0513405	17.38
rs61941722	A	G	0.188737	0.0155992	0.00256577	1.20E-09	0.0241	0.0189	0.2018	11.32
rs61969510	C	T	0.278956	0.0128445	0.00224311	1.00E-08	0.0048	0.0152	0.752501	13.19
rs61992671	G	A	0.491978	-0.0151753	0.00208557	3.40E-13	-0.0061	0.015	0.682801	26.47
rs62070648	A	G	0.268727	-0.0199439	0.0022501	7.80E-19	0.0262	0.0163	0.1089	30.88

SNP	effect_allele HC	other_alleleHC	eaf. HC	beta.HC	se.HC	pval.HC	beta. SSTI	se. SSTI	pval. SSTI	F-statistics
rs62107261	C	T	0.04832	-0.0808476	0.00465016	1.10E-67	-0.0344	0.0524	0.511	27.80
rs62301134	C	T	0.263286	-0.013638	0.00226745	1.80E-09	0.021	0.0163	0.1982	14.03
rs62396185	C	G	0.259989	-0.0346069	0.00227012	1.80E-52	0.0241	0.0151	0.1112	89.44
rs62425398	A	C	0.106309	0.0248634	0.00324644	1.90E-14	0.0442	0.028	0.1138	11.15
rs62515438	G	T	0.227665	0.0150395	0.00237712	2.50E-10	-0.0146	0.0161	0.3647	14.08
rs643499	C	T	0.398022	0.0119575	0.00204369	4.90E-09	0.0195	0.0148	0.1877	16.41
rs6438656	A	G	0.635867	-0.0119598	0.0020673	7.20E-09	-0.0186	0.0156	0.2318	15.50
rs6465828	T	G	0.482837	0.0163908	0.00199173	1.90E-16	4.00E-04	0.015	0.9804	33.82
rs6545714	A	G	0.60145	-0.0158267	0.00203362	7.10E-15	-0.0198	0.015	0.1862	29.04
rs6567160	C	T	0.232716	0.0523537	0.00236068	5.70E-109	0.034	0.019	0.0731206	175.71
rs6575340	A	G	0.636028	0.0184545	0.00207818	6.70E-19	0.0154	0.0152	0.3103	36.51
rs6597975	G	C	0.543875	0.0122921	0.00201118	9.80E-10	-0.0231	0.0149	0.1212	18.53
rs6598540	G	A	0.276885	-0.0147876	0.00223088	3.40E-11	0.0056	0.017	0.743401	17.60
rs6606686	C	G	0.680627	-0.0165692	0.00213651	8.80E-15	0.0131	0.0157	0.4047	26.15
rs66679256	T	C	0.445805	0.0149901	0.00200438	7.50E-14	1.00E-04	0.0153	0.9947	27.64
rs6669341	G	A	0.582713	-0.0151291	0.00201544	6.10E-14	-8.00E-04	0.0151	0.9567	27.41
rs667515	C	G	0.386143	-0.0141684	0.00205316	5.20E-12	0.0023	0.0155	0.8813	22.58
rs6744646	G	A	0.828323	0.0500537	0.00263558	2.00E-80	0.0364	0.02	0.0682905	102.60
rs6745626	T	C	0.589593	0.0139294	0.00202493	6.00E-12	0.0227	0.0151	0.1343	22.90
rs6747657	A	G	0.281723	0.0129341	0.00220754	4.70E-09	-0.0199	0.0165	0.2283	13.89
rs675162	G	A	0.482059	0.0180079	0.00199495	1.80E-19	0.0071	0.0147	0.6285	40.69
rs6840236	C	T	0.464856	0.0169824	0.00199864	1.90E-17	0.0086	0.0147	0.5615	35.92
rs6868125	T	C	0.521906	-0.0119493	0.00198836	1.90E-09	-0.0192	0.015	0.1989	18.02

SNP	effect_allele HC	other_alleleHC	eaf. HC	beta.HC	se.HC	pval.HC	beta. SSTI	se. SSTI	pval. SSTI	F-statistics
rs6946415	G	A	0.627099	0.0158826	0.00206409	1.40E-14	6.00E-04	0.0156	0.9718	27.69
rs6958365	T	C	0.411809	-0.0115375	0.00203582	1.50E-08	-0.0196	0.0153	0.2009	15.56
rs698147	G	A	0.543563	-0.0121448	0.00200109	1.30E-09	-0.0091	0.0147	0.5364	18.28
rs7002088	C	A	0.445199	0.0137173	0.00200556	7.90E-12	-0.0067	0.0169	0.6903	23.11
rs7034554	G	A	0.373815	-0.012422	0.0020595	1.60E-09	-0.0196	0.0162	0.2273	17.03
rs705165	T	G	0.247646	0.0148091	0.00230698	1.40E-10	-0.004	0.0174	0.8166	15.36
rs7111235	C	T	0.493399	0.0115415	0.00200364	8.40E-09	-0.0089	0.0154	0.5647	16.59
rs7124681	A	C	0.408387	0.0205032	0.00202231	3.70E-24	-0.0231	0.0154	0.1339	49.67
rs7132908	A	G	0.384469	0.0257741	0.00204901	2.80E-36	0.0054	0.0152	0.719701	74.90
rs71413981	A	G	0.162848	0.021194	0.00271023	5.30E-15	0.009	0.0215	0.676401	16.67
rs7171864	A	G	0.660205	0.0161986	0.00211572	1.90E-14	-0.0015	0.0161	0.9255	26.30
rs7218014	C	T	0.197308	0.0207669	0.00251132	1.30E-16	0.0236	0.0172	0.1706	21.66
rs7248205	T	C	0.600262	-0.0161199	0.00204315	3.00E-15	-0.0177	0.0151	0.2402	29.87
rs7257083	A	G	0.288605	0.0162612	0.0022051	1.70E-13	-0.012	0.0188	0.5213	22.33
rs72634826	A	G	0.259879	-0.0153317	0.00229923	2.60E-11	-0.0263	0.0181	0.1454	17.11
rs72656010	C	T	0.132181	-0.0252479	0.00295341	1.20E-17	0.0153	0.0221	0.4903	16.77
rs7274811	T	G	0.258589	-0.01951	0.00227858	1.10E-17	0.005	0.0175	0.7772	28.11
rs72756476	C	T	0.141428	-0.0192477	0.00287344	2.10E-11	0.0253	0.024	0.2913	10.90
rs72959041	A	G	0.049137	-0.0662534	0.00466657	9.50E-46	0.0482	0.0301	0.1098	18.84
rs73052033	C	T	0.184918	-0.0208103	0.00256783	5.30E-16	-0.003	0.0199	0.8792	19.80
rs73175572	G	A	0.111726	0.0282075	0.00319248	1.00E-18	-0.0144	0.0229	0.529	15.50
rs73213484	T	A	0.141217	-0.0207006	0.00286036	4.60E-13	0.0137	0.0208	0.5096	12.70
rs7365	G	A	0.482654	-0.0110939	0.00199385	2.60E-08	0.0187	0.0147	0.2032	15.46
rs7372674	A	C	0.357244	0.014356	0.00207575	4.60E-12	-0.0021	0.016	0.8951	21.97

SNP	effect_allele HC	other_alleleHC	eaf. HC	beta.HC	se.HC	pval.HC	beta. SSTI	se. SSTI	pval. SSTI	F-statistics
rs7442885	G	C	0.214027	-0.0198682	0.00243204	3.10E-16	-0.0134	0.0189	0.4801	22.45
rs7460093	A	G	0.53118	0.0136639	0.0020144	1.20E-11	0.0137	0.0149	0.356	22.92
rs74749286	A	G	0.107637	0.0258963	0.00323591	1.20E-15	-0.021	0.0175	0.2301	12.30
rs7498665	G	A	0.399666	0.0317857	0.00203647	6.40E-55	0.0146	0.0149	0.3271	116.93
rs7519259	A	G	0.528388	0.0141441	0.00200058	1.50E-12	0.0087	0.0148	0.5585	24.91
rs7523668	A	G	0.565827	-0.0137199	0.00200928	8.60E-12	0.0078	0.0147	0.598401	22.91
rs756717	A	G	0.399077	-0.0140302	0.00206015	9.70E-12	0.0145	0.0151	0.3364	22.25
rs7570446	A	C	0.544459	0.0110158	0.00199439	3.30E-08	0.0271	0.0148	0.0677096	15.13
rs7571496	G	A	0.260562	-0.0138156	0.00227445	1.20E-09	-0.0038	0.0151	0.7989	14.22
rs7582359	A	G	0.331712	-0.0135492	0.00212104	1.70E-10	-0.0149	0.0157	0.3423	18.09
rs7632381	C	T	0.444325	0.0270429	0.00200044	1.20E-41	-0.0182	0.0148	0.2183	90.26
rs765874	A	T	0.489353	-0.0168012	0.0019903	3.10E-17	0.0073	0.015	0.6243	35.62
rs76647086	T	G	0.176857	-0.0202229	0.00260327	8.00E-15	-0.0236	0.0194	0.2228	17.57
rs76798800	T	G	0.266025	0.0244232	0.00225319	2.20E-27	-0.0117	0.0174	0.5015	45.89
rs7695177	G	C	0.470976	-0.0151527	0.00199491	3.10E-14	-0.0093	0.0148	0.5306	28.75
rs7696175	C	T	0.526478	0.0136068	0.00200383	1.10E-11	0.0089	0.0148	0.547	22.99
rs7707394	A	G	0.357269	-0.0184703	0.002075	5.50E-19	-0.029	0.0152	0.0562199	36.39
rs7708584	G	A	0.572378	-0.013196	0.00201087	5.30E-11	-0.0082	0.015	0.5833	21.08
rs7740107	A	T	0.736289	-0.0236486	0.00225676	1.10E-25	-0.0255	0.0166	0.1243	42.65
rs7793674	C	A	0.144347	0.019239	0.00284373	1.30E-11	0.0247	0.028	0.3776	11.31
rs7793674	C	A	0.144347	0.019239	0.00284373	1.30E-11	-0.059	0.062	0.3416	11.31
rs779655	C	G	0.729184	0.0143261	0.00224052	1.60E-10	0.019	0.0157	0.2271	16.15
rs7805441	T	C	0.502255	0.0126844	0.00200504	2.50E-10	0.004	0.0147	0.784701	20.01
rs7864465	A	G	0.559379	-0.0110794	0.00200561	3.30E-08	0.0045	0.0149	0.763999	15.04

SNP	effect_allele HC	other_alleleHC	eaf. HC	beta.HC	se.HC	pval.HC	beta. SSTI	se. SSTI	pval. SSTI	F-statistics
rs7893571	T	G	0.665871	0.0138381	0.00211863	6.50E-11	-0.0055	0.016	0.7309	18.98
rs7903146	T	C	0.290662	-0.0214133	0.00219381	1.70E-22	-0.0011	0.0184	0.951	39.29
rs7944782	G	T	0.50981	0.0129576	0.0020033	9.90E-11	0.0173	0.0148	0.2429	20.91
rs7952436	T	C	0.081996	-0.0354571	0.00363182	1.60E-22	-0.0624	0.0334	0.0615503	14.35
rs7996639	A	G	0.449371	0.0137138	0.00201868	1.10E-11	0.0212	0.0148	0.1517	22.84
rs8030456	T	C	0.226311	-0.0247721	0.00237863	2.10E-25	-0.0044	0.0199	0.8243	37.98
rs8132129	T	C	0.184901	-0.015922	0.00259474	8.40E-10	-0.0199	0.0179	0.267	11.35
rs8133137	G	A	0.664097	0.0157777	0.00211541	8.80E-14	-0.0047	0.0149	0.750501	24.82
rs815163	C	T	0.563175	-0.0168978	0.00200258	3.20E-17	0.0054	0.0147	0.7133	35.03
rs852042	G	A	0.758582	-0.0153695	0.00233046	4.30E-11	-0.0093	0.0173	0.5935	15.93
rs852983	A	G	0.459576	-0.0118657	0.00199745	2.80E-09	-0.0018	0.0147	0.9006	17.53
rs866006	G	T	0.618508	-0.0120595	0.00204995	4.00E-09	-0.0199	0.015	0.1837	16.33
rs897186	G	A	0.548592	-0.0160621	0.00199856	9.20E-16	-0.004	0.0149	0.7887	31.99
rs9284814	A	G	0.884815	0.0230203	0.00311527	1.50E-13	0.032	0.023	0.1651	11.13
rs9378684	T	C	0.200589	0.018067	0.00250737	5.80E-13	0.0026	0.0165	0.8763	16.65
rs9385385	T	C	0.447856	0.012816	0.00201537	2.00E-10	-0.0175	0.0149	0.2375	20.00
rs9512696	G	A	0.66154	0.0172114	0.00211114	3.60E-16	0.0087	0.0156	0.578599	29.77
rs9513018	T	G	0.616147	-0.0112355	0.00205443	4.50E-08	-0.0167	0.0151	0.2691	14.15
rs968379	T	C	0.229098	-0.0187833	0.00237244	2.40E-15	5.00E-04	0.0175	0.977	22.14
rs9764678	C	T	0.272455	0.0161285	0.00225012	7.60E-13	-0.0044	0.0157	0.780601	20.37
rs9788550	C	G	0.247466	-0.0214137	0.00232004	2.70E-20	-0.0242	0.0154	0.1172	31.73
rs9830592	A	C	0.582425	0.0138364	0.00201841	7.10E-12	0.0033	0.0153	0.8264	22.86
rs9843653	C	T	0.511657	0.0183267	0.00199203	3.60E-20	-0.0017	0.015	0.9075	42.30
rs9845755	T	A	0.198555	0.027361	0.0024977	6.30E-28	-0.0146	0.0194	0.4506	38.19

SNP	effect_allele HC	other_alleleHC	eaf. HC	beta.HC	se.HC	pval.HC	beta. SSTI	se. SSTI	pval. SSTI	F-statistics
rs9850529	A	G	0.339796	0.0114818	0.00209898	4.50E-08	0.0089	0.0163	0.5851	13.43
rs987237	G	A	0.17957	0.0344542	0.00259141	2.50E-40	0.0248	0.018	0.168	52.09
rs9876664	T	G	0.3754	-0.0143788	0.00205893	2.90E-12	-0.0036	0.0148	0.8064	22.87
rs9967287	T	G	0.251722	0.0141454	0.00230082	7.80E-10	0.0252	0.0152	0.0964295	14.24
rs9967367	T	C	0.293494	-0.0174208	0.00220445	2.70E-15	-4.00E-04	0.0154	0.9783	25.90
rs998584	A	C	0.482802	-0.0222736	0.00199467	5.90E-29	2.00E-04	0.0148	0.9913	62.28

Abbreviations: SNP, single nucleotide polymorphism; se, standard error; HC, hip circumference; SSTI, infections of the skin and subcutaneous tissue; pval, p-value.

Table S14. Detailed information about single-nucleotide polymorphisms of hip circumference on acute lower respiratory infections.

SNP	effect_allele HC	other_alleleHC	eaf. HC	beta.HC	se.HC	pval.HC	beta.ALRI	se. ALRI	pval.ALRI	F-statistics
rs1006399	A	G	0.458873	-0.01149	0.002001	9.50E-09	-0.0147	0.0149	0.3239	16.36
rs10100245	A	G	0.564506	0.020539	0.002012	1.80E-24	0.0088	0.0147	0.5479	51.24
rs10118701	G	A	0.32209	0.016807	0.002131	3.10E-15	0.0234	0.015	0.119	27.17
rs10132514	T	C	0.273182	-0.01314	0.002255	5.60E-09	-0.0073	0.0178	0.6819	13.49
rs1013402	G	A	0.318427	0.02937	0.002136	5.10E-43	-0.0177	0.0155	0.2529	82.08
rs10145154	T	C	0.22178	0.025384	0.002404	4.60E-26	-0.0047	0.0171	0.7854	38.49
rs10153248	G	A	0.447835	-0.01589	0.002009	2.60E-15	-0.012	0.0151	0.4276	30.93
rs10172196	A	G	0.305442	0.017444	0.002163	7.40E-16	-0.0103	0.0156	0.5075	27.59
rs10210468	C	T	0.464304	-0.01376	0.002019	9.60E-12	-0.023	0.0147	0.1186	23.09
rs1023767	A	G	0.235192	-0.01535	0.002348	6.20E-11	0.0133	0.0151	0.3809	15.38
rs1037702	A	G	0.62178	-0.01162	0.00206	1.70E-08	-0.0031	0.015	0.8353	14.97
rs10404726	T	C	0.465501	-0.01665	0.002003	9.60E-17	-0.0016	0.0147	0.9135	34.36
rs10407871	C	T	0.155426	-0.0182	0.002765	4.70E-11	0.0094	0.0194	0.627899	11.37
rs10471636	A	G	0.508884	-0.01183	0.002032	5.70E-09	1.00E-04	0.0148	0.9937	16.95
rs1047891	A	C	0.315753	0.016603	0.002137	7.80E-15	0.0162	0.0157	0.3015	26.09
rs1056441	C	T	0.675428	0.014638	0.002132	6.60E-12	0.0182	0.0168	0.2769	20.67
rs1056720	T	C	0.234833	-0.01306	0.002351	2.80E-08	-0.0082	0.0184	0.657099	11.10
rs10744145	A	C	0.513795	-0.01212	0.001997	1.30E-09	-0.0092	0.0146	0.5295	18.40
rs10746833	G	A	0.581617	-0.01494	0.002036	2.20E-13	-0.0239	0.0147	0.1043	26.21
rs10773051	T	C	0.222167	0.023142	0.002403	5.90E-22	-0.0104	0.0161	0.5182	32.06
rs10810598	A	T	0.639535	-0.01293	0.002078	4.90E-10	0.0051	0.0153	0.7403	17.84
rs10883553	A	C	0.445855	0.015105	0.002007	5.20E-14	0.0034	0.0148	0.8197	27.99
rs10938397	G	A	0.434366	0.02439	0.002011	7.70E-34	0.0046	0.0147	0.754201	72.26

SNP	effect_allele HC	other_alleleHC	eaf. HC	beta.HC	se.HC	pval.HC	beta.ALRI	se. ALRI	pval.ALRI	F-statistics
rs10954284	A	T	0.488124	0.019314	0.001992	3.20E-22	0.0122	0.0147	0.4061	46.96
rs10987417	T	G	0.385638	0.012808	0.00207	6.10E-10	0.0173	0.0147	0.2405	18.14
rs11012732	G	A	0.331674	0.020089	0.002119	2.50E-21	0.0062	0.0158	0.6946	39.86
rs11030016	T	C	0.739706	0.018143	0.002276	1.60E-15	0.0062	0.0156	0.6897	24.47
rs11045163	G	A	0.430597	0.011451	0.002016	1.30E-08	0.0048	0.015	0.749501	15.82
rs11075252	G	A	0.284481	-0.01256	0.002215	1.40E-08	0.0092	0.0156	0.5569	13.10
rs1108548	G	A	0.27725	0.015652	0.002227	2.10E-12	-0.0105	0.0158	0.5078	19.79
rs11107114	A	G	0.228885	0.013324	0.002375	2.00E-08	0.0062	0.0156	0.691399	11.11
rs11113445	G	A	0.391358	0.01275	0.002039	4.00E-10	-0.0118	0.0151	0.4335	18.64
rs11150461	G	C	0.727351	-0.01416	0.002247	3.00E-10	-0.0023	0.018	0.899	15.74
rs11150745	G	A	0.317702	-0.02028	0.002146	3.40E-21	0.0014	0.0163	0.9323	38.70
rs11164630	T	C	0.608331	-0.01381	0.002042	1.30E-11	-0.0194	0.0153	0.2069	21.81
rs11165643	T	C	0.590085	0.018526	0.00202	4.70E-20	0.0073	0.015	0.626299	40.68
rs11173522	A	C	0.214329	0.013694	0.002429	1.70E-08	-0.0116	0.0185	0.5299	10.70
rs1123295	G	A	0.444369	0.011954	0.002005	2.50E-09	-0.0157	0.0148	0.2877	17.55
rs11263719	T	C	0.469657	0.011499	0.002008	1.00E-08	0.008	0.0149	0.5915	16.33
rs112875651	A	G	0.391041	0.018266	0.002068	1.00E-18	-0.0063	0.0151	0.6773	37.14
rs113230003	A	G	0.260523	-0.01486	0.002292	9.00E-11	0.0233	0.018	0.195	16.19
rs11513729	T	C	0.413028	-0.01665	0.002051	4.70E-16	0.0167	0.0151	0.2678	31.97
rs11664106	T	A	0.37403	0.016775	0.002113	2.00E-15	0.0122	0.0158	0.4403	29.51
rs11704728	T	C	0.196356	0.014832	0.002524	4.20E-09	0.0245	0.0178	0.1695	10.90
rs11751684	T	G	0.275389	0.016732	0.002223	5.20E-14	0.0147	0.019	0.4392	22.60
rs11762444	A	G	0.216137	0.014599	0.002419	1.60E-09	-0.0172	0.0184	0.3491	12.34
rs11778934	G	C	0.536083	-0.01413	0.002005	1.80E-12	0.0285	0.0153	0.06278	24.71

SNP	effect_allele HC	other_alleleHC	eaf. HC	beta.HC	se.HC	pval.HC	beta.ALRI	se. ALRI	pval.ALRI	F-statistics
rs11779446	G	A	0.161259	-0.02018	0.00272	1.20E-13	0.0178	0.0209	0.395	14.89
rs1182199	A	C	0.304395	-0.02465	0.002167	5.40E-30	-0.0059	0.0152	0.696299	54.83
rs11882796	T	A	0.541285	-0.01138	0.002003	1.30E-08	0.0067	0.0148	0.651799	16.04
rs11997077	G	A	0.380458	-0.01129	0.002066	4.70E-08	0.0112	0.0147	0.4461	14.07
rs12072739	G	A	0.224461	0.01499	0.002387	3.40E-10	0.0233	0.0157	0.1388	13.73
rs12122361	G	A	0.265617	-0.01377	0.00226	1.10E-09	-0.0046	0.0159	0.7715	14.49
rs12128526	A	G	0.457416	0.011368	0.001996	1.20E-08	-0.0049	0.0147	0.7364	16.10
rs12364470	G	T	0.164552	0.017881	0.002687	2.80E-11	-0.0087	0.0175	0.6201	12.17
rs12375196	A	C	0.424347	0.016759	0.002028	1.40E-16	-0.023	0.0147	0.1181	33.36
rs12427047	T	C	0.24273	-0.0164	0.002323	1.70E-12	-0.0069	0.0155	0.6557	18.32
rs12441543	A	G	0.2871	0.015234	0.002209	5.30E-12	-0.0022	0.0162	0.8928	19.47
rs12462975	A	G	0.329669	0.018907	0.002137	9.00E-19	-0.0513	0.0158	0.001163	34.60
rs12519997	A	G	0.559278	-0.014	0.002004	2.90E-12	-0.0066	0.0153	0.664	24.05
rs12568411	A	G	0.166749	0.016481	0.002694	9.40E-10	0.0125	0.0174	0.4743	10.40
rs12569355	G	A	0.119536	0.021744	0.003077	1.60E-12	-0.0354	0.0293	0.2274	10.51
rs1260326	C	T	0.604267	0.012037	0.00203	3.00E-09	-0.0145	0.0154	0.3451	16.81
rs12607512	G	A	0.44632	0.011784	0.002008	4.40E-09	0.0252	0.0147	0.086171	17.02
rs12631813	G	C	0.50262	-0.01227	0.002	8.50E-10	5.00E-04	0.0147	0.9745	18.82
rs12762744	T	C	0.248261	0.013317	0.002312	8.40E-09	0.0158	0.0161	0.3264	12.38
rs12831185	G	A	0.170442	-0.01764	0.002655	3.10E-11	-0.0143	0.0232	0.5376	12.48
rs1285992	G	A	0.710447	0.020316	0.002203	2.90E-20	0.0078	0.0166	0.6389	34.99
rs12880641	G	T	0.661578	-0.01492	0.002106	1.40E-12	-0.004	0.0151	0.790801	22.47
rs12883788	T	C	0.459801	0.014386	0.002009	7.90E-13	-0.0165	0.0149	0.2655	25.48
rs12921916	C	T	0.28756	0.012596	0.002228	1.60E-08	0.0142	0.0168	0.397	13.10

SNP	effect_allele HC	other_alleleHC	eaf. HC	beta.HC	se.HC	pval.HC	beta.ALRI	se. ALRI	pval.ALRI	F-statistics
rs12939848	T	C	0.401034	0.01264	0.002038	5.60E-10	-0.0256	0.0151	0.08901	18.48
rs1294438	T	C	0.354583	0.018627	0.002108	9.70E-19	-0.0205	0.0153	0.1797	35.76
rs1296328	C	A	0.559033	-0.01582	0.002015	4.20E-15	3.00E-04	0.0147	0.9821	30.37
rs13017207	A	G	0.393634	-0.01615	0.002034	2.00E-15	0.0112	0.015	0.4545	30.11
rs13034765	C	G	0.370011	0.013375	0.002064	9.10E-11	0.0153	0.0155	0.325	19.58
rs13107325	T	C	0.074895	0.038665	0.003786	1.70E-24	-0.046	0.0619	0.4568	14.46
rs13148263	A	G	0.335162	0.015944	0.002105	3.60E-14	-0.0106	0.0148	0.4735	25.57
rs13156484	A	G	0.472171	-0.01749	0.002015	3.90E-18	0.0241	0.0147	0.1008	37.58
rs13264909	T	A	0.428953	-0.01361	0.002017	1.50E-11	-0.0261	0.0147	0.07553	22.29
rs13284988	C	T	0.305411	0.012076	0.002179	3.00E-08	0.0145	0.0166	0.3827	13.04
rs13292699	C	A	0.433708	-0.01627	0.002015	6.70E-16	6.00E-04	0.0147	0.9673	32.05
rs133015	G	C	0.439948	0.012962	0.002016	1.30E-10	-0.0126	0.0149	0.3979	20.38
rs13333747	C	T	0.182674	-0.02197	0.002592	2.30E-17	0.0091	0.0192	0.635501	21.46
rs13389219	T	C	0.392395	0.02276	0.002034	4.70E-29	0.0025	0.0154	0.8696	59.69
rs143384	G	A	0.404382	0.02675	0.002031	1.30E-39	-0.0245	0.0148	0.097129	83.55
rs1446585	G	A	0.244506	-0.01285	0.002268	1.50E-08	0.0247	0.0154	0.1083	11.86
rs1452082	A	C	0.557071	0.012586	0.00204	6.80E-10	0.0156	0.015	0.2965	18.79
rs1458156	T	C	0.488404	0.012649	0.001995	2.30E-10	-0.0134	0.0147	0.3635	20.10
rs1477290	C	T	0.136945	0.029863	0.002921	1.60E-24	0.035	0.0224	0.1182	24.70
rs147730268	T	G	0.087233	-0.05061	0.003611	1.30E-44	-0.0392	0.0264	0.1385	31.28
rs1480474	G	A	0.417444	-0.01451	0.002023	7.40E-13	0.0103	0.015	0.4929	25.01
rs1481630	C	T	0.176871	-0.01539	0.002615	4.00E-09	0.0245	0.0208	0.2393	10.09
rs1502317	T	C	0.27657	-0.01516	0.002232	1.10E-11	0.0092	0.0166	0.5815	18.46
rs1514895	G	A	0.285289	0.01747	0.002202	2.10E-15	-0.0053	0.0166	0.750501	25.67

SNP	effect_allele HC	other_alleleHC	eaf. HC	beta.HC	se.HC	pval.HC	beta.ALRI	se. ALRI	pval.ALRI	F-statistics
rs1538535	T	C	0.27456	0.012439	0.002237	2.70E-08	0.0134	0.0177	0.4477	12.32
rs1576655	C	A	0.59598	0.016133	0.002075	7.70E-15	0.0066	0.0157	0.671699	29.10
rs1609783	A	G	0.525344	0.014272	0.002004	1.10E-12	-0.0109	0.0147	0.459501	25.28
rs16868443	C	G	0.360418	0.015435	0.002076	1.00E-13	-0.0234	0.0166	0.1585	25.50
rs170016	A	G	0.633948	-0.01165	0.002093	2.60E-08	0.0155	0.0157	0.3243	14.37
rs1727901	T	C	0.736676	0.020443	0.00226	1.50E-19	-0.0171	0.0165	0.2989	31.74
rs17361789	G	T	0.32182	0.012642	0.002148	4.00E-09	0.0024	0.0165	0.8826	15.12
rs17639546	A	G	0.14849	-0.01877	0.0028	2.00E-11	-0.0017	0.0212	0.9359	11.36
rs17733217	G	A	0.229127	-0.01581	0.002379	3.10E-11	-0.0129	0.019	0.4982	15.59
rs17766836	T	C	0.267888	0.023232	0.002252	6.10E-25	-0.0065	0.0169	0.702	41.73
rs17770336	T	C	0.322443	0.019146	0.002129	2.40E-19	6.00E-04	0.0151	0.9671	35.34
rs1813212	G	A	0.445576	-0.01301	0.002008	9.20E-11	-0.002	0.0148	0.8949	20.74
rs1868069	A	G	0.229248	0.015095	0.002369	1.90E-10	0.0103	0.0177	0.5605	14.34
rs1934394	C	G	0.228157	0.012999	0.002383	4.90E-08	-0.016	0.0176	0.3645	10.48
rs1955695	G	A	0.62227	-0.01712	0.002059	8.90E-17	-0.0073	0.0161	0.6532	32.53
rs2022050	A	G	0.160924	-0.02088	0.00272	1.70E-14	-0.004	0.0177	0.8212	15.90
rs2023211	C	T	0.232254	0.016039	0.002362	1.10E-11	0.0015	0.0209	0.9436	16.45
rs2032251	A	T	0.507961	0.011405	0.001998	1.10E-08	0.005	0.0151	0.7405	16.29
rs211434	A	G	0.681996	0.013134	0.002141	8.60E-10	0.0306	0.0155	0.04825	16.32
rs2133292	T	C	0.373582	0.01323	0.002066	1.50E-10	-0.004	0.0158	0.8001	19.20
rs2133561	T	A	0.611067	-0.01819	0.002064	1.20E-18	-0.0096	0.0153	0.5309	36.92
rs2151248	C	G	0.730659	-0.01493	0.00224	2.60E-11	0.0029	0.0167	0.8637	17.49
rs2159437	G	A	0.517573	0.019624	0.001998	8.90E-23	0.005	0.015	0.7411	48.20
rs2172131	C	T	0.578698	-0.01369	0.00202	1.20E-11	-0.0094	0.0146	0.5202	22.39

SNP	effect_allele HC	other_alleleHC	eaf. HC	beta.HC	se.HC	pval.HC	beta.ALRI	se. ALRI	pval.ALRI	F-statistics
rs2178385	T	G	0.320337	0.018547	0.002133	3.40E-18	-0.013	0.0161	0.419	32.93
rs2238435	G	C	0.613709	0.025955	0.002052	1.10E-36	-0.0231	0.0149	0.1203	75.89
rs2238689	C	T	0.399438	-0.01417	0.002037	3.60E-12	-0.0279	0.0147	0.0582	23.19
rs2253310	G	C	0.626098	0.019536	0.002056	2.10E-21	-0.0059	0.0148	0.692	42.27
rs2270894	G	C	0.203219	-0.02164	0.002568	3.50E-17	-0.0072	0.0167	0.666	23.01
rs2288745	T	C	0.299451	0.013663	0.002177	3.50E-10	4.00E-04	0.0152	0.9776	16.53
rs2307111	C	T	0.394977	-0.02854	0.002038	1.60E-44	-0.043	0.0148	0.003748	93.69
rs2371767	C	G	0.277705	0.019414	0.002218	2.10E-18	0.0575	0.0177	0.001142	30.72
rs2371911	A	T	0.544326	0.013106	0.001999	5.50E-11	-0.0056	0.0147	0.7036	21.33
rs2384054	C	T	0.489428	0.021906	0.001987	2.90E-28	0.0195	0.0148	0.1893	60.76
rs2479958	G	A	0.516982	-0.01299	0.002019	1.20E-10	-0.0064	0.0147	0.661301	20.67
rs2494196	A	C	0.286135	0.031274	0.002199	6.90E-46	0.0301	0.0161	0.06093	82.61
rs2499468	A	C	0.651137	0.013183	0.00209	2.80E-10	0.0102	0.0166	0.539299	18.07
rs253444	A	G	0.12923	-0.01982	0.002972	2.50E-11	-0.0359	0.0188	0.05653	10.01
rs2568958	A	G	0.603693	0.020671	0.002029	2.20E-24	-0.0053	0.0153	0.7309	49.67
rs2577955	T	C	0.802767	-0.01467	0.002505	4.80E-09	-0.0033	0.0197	0.8676	10.85
rs25849	G	C	0.288829	0.019767	0.002208	3.50E-19	0.0183	0.0152	0.2302	32.92
rs2585526	G	A	0.557413	0.012267	0.002011	1.10E-09	0.0054	0.0147	0.711101	18.35
rs2593169	C	G	0.567965	-0.01163	0.002018	8.20E-09	-0.0191	0.0148	0.1961	16.31
rs2606227	C	T	0.63145	-0.01243	0.002083	2.40E-09	2.00E-04	0.0149	0.9919	16.59
rs2642305	T	A	0.369566	0.011304	0.002067	4.50E-08	0.0054	0.0171	0.7508	13.94
rs2660241	C	T	0.364912	0.013914	0.002073	1.90E-11	-0.0284	0.0153	0.063879	20.88
rs2678204	G	T	0.340172	0.019675	0.002099	7.10E-21	-0.0029	0.0161	0.8567	39.44
rs273505	C	T	0.422021	0.012874	0.002021	1.90E-10	0.0064	0.0146	0.6594	19.79

SNP	effect_allele HC	other_alleleHC	eaf. HC	beta.HC	se.HC	pval.HC	beta.ALRI	se. ALRI	pval.ALRI	F-statistics
rs2737263	T	G	0.28052	-0.02366	0.00222	1.60E-26	0.0052	0.0158	0.742499	45.84
rs2744956	C	T	0.139254	0.053758	0.00287	2.80E-78	0.0175	0.0184	0.3412	84.11
rs2814350	A	G	0.304993	0.013872	0.002209	3.40E-10	0.0161	0.0152	0.2883	16.72
rs2821226	G	A	0.527763	0.015138	0.002004	4.30E-14	-0.0082	0.0148	0.581201	28.44
rs28366156	C	T	0.130582	-0.02658	0.002952	2.20E-19	-0.0034	0.0286	0.9061	18.41
rs2861685	C	T	0.411983	-0.01478	0.002015	2.30E-13	0.0237	0.0149	0.1107	26.05
rs28711392	C	T	0.367255	-0.01252	0.002084	1.90E-09	-0.0117	0.0147	0.4267	16.78
rs28778940	A	G	0.330838	0.015151	0.002124	9.70E-13	0.0061	0.0156	0.6968	22.54
rs287837	G	A	0.523844	-0.01308	0.002002	6.50E-11	-0.017	0.0147	0.2469	21.28
rs2897968	A	G	0.607035	0.012276	0.002044	1.90E-09	-0.0032	0.0149	0.8301	17.20
rs3012053	G	A	0.714878	-0.01497	0.002209	1.20E-11	-0.005	0.0156	0.746799	18.73
rs308911	G	A	0.714423	-0.01428	0.002206	9.70E-11	0.0117	0.0163	0.4747	17.09
rs310796	T	G	0.680961	0.012109	0.002144	1.60E-08	-0.0245	0.0166	0.1413	13.86
rs3110942	A	G	0.481664	0.012712	0.001998	2.00E-10	0.0011	0.0147	0.9395	20.21
rs3116600	G	A	0.215319	-0.01877	0.002425	1.00E-14	-0.0045	0.0169	0.791099	20.24
rs314288	C	T	0.886256	-0.02271	0.003138	4.60E-13	0.0061	0.0223	0.7828	10.56
rs329118	T	C	0.419386	-0.01635	0.00202	5.80E-16	-0.0273	0.0148	0.06558	31.90
rs33503	A	G	0.80589	-0.0175	0.002514	3.40E-12	-0.022	0.0186	0.2382	15.16
rs340025	C	T	0.57994	0.012741	0.002028	3.30E-10	0.0051	0.0157	0.7453	19.23
rs34223321	A	C	0.300092	0.014092	0.002173	8.80E-11	0.0048	0.0153	0.7538	17.67
rs34517439	A	C	0.121795	0.045455	0.003076	2.00E-49	0.0069	0.0221	0.754899	46.73
rs34594435	T	C	0.195585	0.021579	0.002512	8.60E-18	0.0068	0.0191	0.723501	23.22
rs34629844	G	A	0.12828	0.021295	0.002976	8.30E-13	-0.0406	0.0257	0.1134	11.45
rs34656389	G	A	0.367473	0.011297	0.002066	4.60E-08	0.0115	0.0147	0.4344	13.89

SNP	effect_allele HC	other_alleleHC	eaf. HC	beta.HC	se.HC	pval.HC	beta.ALRI	se. ALRI	pval.ALRI	F-statistics
rs34765854	G	T	0.298258	0.019332	0.002172	5.50E-19	0.0075	0.0156	0.6283	33.17
rs34769775	T	C	0.29735	-0.01411	0.002183	1.00E-10	-0.0033	0.0166	0.8441	17.46
rs34772064	G	T	0.555918	-0.01187	0.002003	3.10E-09	0.0142	0.0147	0.3324	17.35
rs34811474	A	G	0.230735	-0.01979	0.002362	5.40E-17	0.0121	0.0175	0.4897	24.92
rs34840745	T	C	0.262486	0.016643	0.002272	2.40E-13	0.0496	0.0163	0.00233	20.78
rs34848742	G	T	0.788211	-0.01436	0.002436	3.70E-09	-0.0426	0.0191	0.02559	11.60
rs35506085	A	G	0.185085	-0.02048	0.002586	2.40E-15	0.0104	0.0195	0.5924	18.92
rs35537311	T	C	0.388607	-0.01476	0.00205	6.00E-13	-0.006	0.015	0.6887	24.65
rs35792595	A	T	0.297453	0.013773	0.002192	3.30E-10	0.0272	0.0167	0.1029	16.51
rs35882248	T	C	0.317213	0.015668	0.002138	2.30E-13	8.00E-04	0.0158	0.9591	23.27
rs35910339	G	C	0.683708	-0.0127	0.002142	3.00E-09	-0.0011	0.0179	0.9523	15.21
rs35917007	G	A	0.533673	0.014757	0.001999	1.50E-13	0.0082	0.0148	0.578	27.14
rs35957544	T	G	0.574337	-0.01926	0.00202	1.50E-21	0.0082	0.0149	0.5844	44.44
rs36140	C	A	0.635313	0.014843	0.00208	9.50E-13	0.0123	0.0154	0.4245	23.61
rs365352	A	G	0.24439	-0.02054	0.002313	6.60E-19	0.0011	0.0188	0.9524	29.13
rs3737992	A	G	0.168963	-0.02151	0.002651	4.90E-16	-0.0628	0.0186	0.000753	18.49
rs3746759	G	T	0.205871	-0.01448	0.002468	4.40E-09	-0.0188	0.0219	0.3892	11.26
rs3762988	T	C	0.388074	0.01265	0.002048	6.50E-10	-0.0199	0.0153	0.1935	18.12
rs3807566	T	G	0.438301	-0.01495	0.002012	1.10E-13	-0.0024	0.0151	0.873	27.18
rs3810291	A	G	0.674991	0.022735	0.002129	1.30E-26	0.0091	0.0152	0.5486	50.05
rs3811951	G	A	0.282058	0.015018	0.002211	1.10E-11	0.0013	0.0162	0.9344	18.68
rs3814883	T	C	0.482403	0.025069	0.002	5.00E-36	0.0146	0.0148	0.3258	78.43
rs3826408	T	C	0.456783	0.013589	0.002001	1.10E-11	-0.0114	0.0147	0.4391	22.89
rs3845344	T	C	0.391164	0.01387	0.002036	9.70E-12	0.0152	0.0148	0.3052	22.10

SNP	effect_allele HC	other_alleleHC	eaf. HC	beta.HC	se.HC	pval.HC	beta.ALRI	se. ALRI	pval.ALRI	F-statistics
rs3935190	A	G	0.536778	-0.01411	0.002012	2.30E-12	0.0337	0.0147	0.02188	24.46
rs394608	C	T	0.537697	0.014611	0.00201	3.60E-13	0.0047	0.0148	0.7524	26.27
rs40071	C	T	0.179489	-0.01741	0.002603	2.20E-11	-0.0165	0.018	0.3588	13.18
rs4017425	T	C	0.470189	-0.01184	0.001997	3.10E-09	-0.0056	0.0151	0.7126	17.51
rs41273794	T	C	0.287079	0.019145	0.002202	3.50E-18	0.0022	0.0169	0.8957	30.94
rs4240326	G	A	0.550142	-0.02793	0.002001	2.70E-44	0.0143	0.0147	0.3305	96.50
rs4253755	A	G	0.128527	0.021132	0.003003	2.00E-12	-0.0216	0.0268	0.4201	11.09
rs429343	G	A	0.576577	-0.0122	0.002015	1.40E-09	-0.0085	0.0147	0.5623	17.89
rs4310395	G	A	0.442854	0.012218	0.002002	1.00E-09	0.0036	0.0147	0.8056	18.37
rs4467770	A	G	0.731091	0.015878	0.002254	1.80E-12	-0.0342	0.0162	0.03508	19.52
rs4476935	T	C	0.433157	-0.01287	0.002011	1.50E-10	-0.0019	0.015	0.8985	20.13
rs4477562	T	C	0.128661	0.02922	0.003004	2.30E-22	0.0208	0.0212	0.3254	21.22
rs4486868	C	T	0.447992	-0.01111	0.002011	3.40E-08	-0.0014	0.0148	0.9245	15.08
rs4567604	T	G	0.193192	-0.01668	0.002539	5.00E-11	-0.0406	0.0194	0.03589	13.46
rs4589131	G	T	0.590569	-0.01173	0.002021	6.40E-09	-0.0002	0.0148	0.9914	16.31
rs4613074	C	T	0.18516	-0.01702	0.002566	3.30E-11	0.0055	0.0194	0.778901	13.28
rs463376	A	G	0.490305	0.011993	0.001998	1.90E-09	-0.0197	0.0147	0.1789	18.02
rs4678016	T	C	0.366307	0.011514	0.002065	2.50E-08	0.0018	0.0147	0.902	14.43
rs475390	A	G	0.776432	-0.01407	0.002389	3.90E-09	-0.0057	0.0176	0.7449	12.04
rs4790292	A	C	0.153689	-0.02619	0.002777	4.10E-21	-0.0218	0.0196	0.2647	23.13
rs4792716	G	A	0.562173	0.014561	0.002012	4.60E-13	0.0326	0.0149	0.02878	25.79
rs4800490	C	A	0.495235	-0.02317	0.001996	3.60E-31	0.0104	0.0147	0.4794	67.42
rs4820346	G	C	0.69295	-0.01305	0.002171	1.80E-09	1.00E-04	0.015	0.9929	15.38
rs483465	G	A	0.749331	0.015493	0.002297	1.50E-11	0.042	0.0204	0.03982	17.09

SNP	effect_allele HC	other_alleleHC	eaf. HC	beta.HC	se.HC	pval.HC	beta.ALRI	se. ALRI	pval.ALRI	F-statistics
rs4843158	C	G	0.68516	0.021846	0.002149	2.80E-24	-0.006	0.0158	0.7047	44.59
rs4870057	G	A	0.341223	0.012693	0.002127	2.40E-09	0.0155	0.0174	0.3719	16.01
rs4909309	C	T	0.393916	-0.01735	0.002039	1.70E-17	0.0282	0.0147	0.05408	34.58
rs4962424	A	T	0.326831	0.015585	0.002127	2.40E-13	-0.0076	0.0163	0.641501	23.61
rs4963975	A	G	0.244315	-0.01879	0.002324	6.10E-16	0.0093	0.0163	0.5687	24.15
rs4976994	G	A	0.452549	0.012839	0.002005	1.50E-10	-0.0171	0.0148	0.2477	20.32
rs4985407	G	A	0.49763	0.015303	0.001997	1.80E-14	-0.0146	0.015	0.3317	29.35
rs522110	G	A	0.555042	0.018119	0.002007	1.70E-19	0.0153	0.0147	0.2978	40.27
rs543874	G	A	0.205218	0.045364	0.002462	8.00E-76	-0.0088	0.0191	0.6458	110.79
rs551935	G	T	0.440778	-0.01693	0.002005	3.10E-17	-0.0111	0.0154	0.4727	35.13
rs55726687	A	G	0.209736	0.021817	0.002445	4.50E-19	0.0072	0.0179	0.6863	26.40
rs56094641	G	A	0.404596	0.061633	0.002031	1E-200	0.0211	0.0148	0.1549	444.31
rs56097510	G	C	0.51052	0.013713	0.001998	6.70E-12	0.0144	0.0146	0.3251	23.54
rs56399737	T	C	0.449109	-0.01182	0.002013	4.30E-09	0.0096	0.0148	0.5153	17.07
rs57222629	C	G	0.311673	0.013815	0.002156	1.50E-10	-0.0059	0.0152	0.699	17.61
rs57235969	T	C	0.554199	0.012462	0.002	4.60E-10	0.0132	0.0147	0.3692	19.19
rs57636386	C	T	0.08382	-0.03508	0.003612	2.60E-22	-0.0277	0.0336	0.4106	14.49
rs57989773	C	T	0.245008	0.021874	0.002381	4.00E-20	-0.0085	0.0184	0.645	31.24
rs58584712	A	G	0.211251	0.016977	0.002436	3.20E-12	0.0382	0.0193	0.04773	16.19
rs58862095	T	C	0.419278	-0.01811	0.002024	3.70E-19	-0.0013	0.0147	0.9319	38.96
rs59227842	G	A	0.311488	0.021023	0.00217	3.40E-22	-0.0006	0.0158	0.9675	40.25
rs60226453	T	C	0.177273	0.016272	0.0026	3.90E-10	-0.0088	0.0198	0.657099	11.42
rs6066104	T	C	0.324108	0.019283	0.002135	1.70E-19	-0.003	0.0152	0.8447	35.76
rs6142059	C	T	0.492562	0.014997	0.002	6.50E-14	-0.0151	0.0147	0.3042	28.10

SNP	effect_allele HC	other_alleleHC	eaf. HC	beta.HC	se.HC	pval.HC	beta.ALRI	se. ALRI	pval.ALRI	F-statistics
rs614520	A	G	0.640637	-0.01303	0.00212	8.00E-10	-0.0134	0.0156	0.3917	17.38
rs61941722	A	G	0.188737	0.015599	0.002566	1.20E-09	0.0195	0.0188	0.2986	11.32
rs61969510	C	T	0.278956	0.012845	0.002243	1.00E-08	0.012	0.0152	0.4309	13.19
rs61992671	G	A	0.491978	-0.01518	0.002086	3.40E-13	0.0017	0.0149	0.91	26.47
rs62070648	A	G	0.268727	-0.01994	0.00225	7.80E-19	-0.0045	0.0163	0.7813	30.88
rs62107261	C	T	0.04832	-0.08085	0.00465	1.10E-67	0.0772	0.052	0.1376	27.80
rs62301134	C	T	0.263286	-0.01364	0.002267	1.80E-09	-0.0311	0.0163	0.05618	14.03
rs62396185	C	G	0.259989	-0.03461	0.00227	1.80E-52	-0.0168	0.0151	0.2666	89.44
rs62425398	A	C	0.106309	0.024863	0.003246	1.90E-14	-0.0305	0.0279	0.2755	11.15
rs62515438	G	T	0.227665	0.01504	0.002377	2.50E-10	0.0269	0.016	0.0933	14.08
rs643499	C	T	0.398022	0.011958	0.002044	4.90E-09	6.00E-04	0.0147	0.9664	16.41
rs6438656	A	G	0.635867	-0.01196	0.002067	7.20E-09	0.0184	0.0155	0.2352	15.50
rs6465828	T	G	0.482837	0.016391	0.001992	1.90E-16	4.00E-04	0.0149	0.9798	33.82
rs6545714	A	G	0.60145	-0.01583	0.002034	7.10E-15	0.008	0.0149	0.5937	29.04
rs6567160	C	T	0.232716	0.052354	0.002361	5.7E-109	0.0065	0.0189	0.728501	175.71
rs6575340	A	G	0.636028	0.018455	0.002078	6.70E-19	0.0408	0.0152	0.007042	36.51
rs6597975	G	C	0.543875	0.012292	0.002011	9.80E-10	-0.0297	0.0148	0.04552	18.53
rs6598540	G	A	0.276885	-0.01479	0.002231	3.40E-11	-0.0069	0.0169	0.6838	17.60
rs6606686	C	G	0.680627	-0.01657	0.002137	8.80E-15	0.0132	0.0157	0.3999	26.15
rs66679256	T	C	0.445805	0.01499	0.002004	7.50E-14	-0.0091	0.0152	0.5487	27.64
rs6669341	G	A	0.582713	-0.01513	0.002015	6.10E-14	-0.0092	0.0151	0.5424	27.41
rs667515	C	G	0.386143	-0.01417	0.002053	5.20E-12	0.0015	0.0155	0.9222	22.58
rs6744646	G	A	0.828323	0.050054	0.002636	2.00E-80	0.019	0.0199	0.3395	102.60
rs6745626	T	C	0.589593	0.013929	0.002025	6.00E-12	0.0202	0.0151	0.1811	22.90

SNP	effect_allele HC	other_alleleHC	eaf. HC	beta.HC	se.HC	pval.HC	beta.ALRI	se. ALRI	pval.ALRI	F-statistics
rs6747657	A	G	0.281723	0.012934	0.002208	4.70E-09	0.0414	0.0165	0.01186	13.89
rs675162	G	A	0.482059	0.018008	0.001995	1.80E-19	-0.029	0.0146	0.04783	40.69
rs6840236	C	T	0.464856	0.016982	0.001999	1.90E-17	-0.0108	0.0147	0.46	35.92
rs6868125	T	C	0.521906	-0.01195	0.001988	1.90E-09	0.0043	0.0149	0.7719	18.02
rs6946415	G	A	0.627099	0.015883	0.002064	1.40E-14	-0.0344	0.0156	0.02707	27.69
rs6958365	T	C	0.411809	-0.01154	0.002036	1.50E-08	0	0.0153	0.9993	15.56
rs6973700	G	A	0.20616	-0.01578	0.002466	1.50E-10	-0.0094	0.0165	0.5673	13.41
rs698147	G	A	0.543563	-0.01214	0.002001	1.30E-09	0.0119	0.0146	0.4179	18.28
rs7002088	C	A	0.445199	0.013717	0.002006	7.90E-12	-0.0215	0.0169	0.2027	23.11
rs7034554	G	A	0.373815	-0.01242	0.00206	1.60E-09	-0.0174	0.0161	0.2806	17.03
rs7038943	C	T	0.338791	-0.01211	0.002104	8.60E-09	0.011	0.015	0.4637	14.84
rs705165	T	G	0.247646	0.014809	0.002307	1.40E-10	0.0195	0.0173	0.2607	15.36
rs7094073	T	C	0.191266	0.016348	0.002735	2.30E-09	-0.0571	0.0212	0.007157	11.05
rs7111235	C	T	0.493399	0.011542	0.002004	8.40E-09	-0.0161	0.0153	0.2932	16.59
rs7124681	A	C	0.408387	0.020503	0.002022	3.70E-24	-0.0154	0.0153	0.314	49.67
rs7132908	A	G	0.384469	0.025774	0.002049	2.80E-36	-0.0243	0.0151	0.1073	74.90
rs71413981	A	G	0.162848	0.021194	0.00271	5.30E-15	0.005	0.0214	0.8157	16.67
rs7171864	A	G	0.660205	0.016199	0.002116	1.90E-14	0.0069	0.0161	0.6665	26.30
rs7218014	C	T	0.197308	0.020767	0.002511	1.30E-16	0.0341	0.0172	0.04698	21.66
rs7248205	T	C	0.600262	-0.01612	0.002043	3.00E-15	-0.0048	0.015	0.748101	29.87
rs7257083	A	G	0.288605	0.016261	0.002205	1.70E-13	-0.0025	0.0187	0.8944	22.33
rs72634826	A	G	0.259879	-0.01533	0.002299	2.60E-11	-0.0022	0.0181	0.9037	17.11
rs72656010	C	T	0.132181	-0.02525	0.002953	1.20E-17	-0.0127	0.0221	0.5653	16.77
rs7274811	T	G	0.258589	-0.01951	0.002279	1.10E-17	-0.0159	0.0175	0.3629	28.11

SNP	effect_allele HC	other_alleleHC	eaf. HC	beta.HC	se.HC	pval.HC	beta.ALRI	se. ALRI	pval.ALRI	F-statistics
rs72756476	C	T	0.141428	-0.01925	0.002873	2.10E-11	-0.0211	0.0239	0.3779	10.90
rs72959041	A	G	0.049137	-0.06625	0.004667	9.50E-46	-0.0011	0.0301	0.9718	18.84
rs73052033	C	T	0.184918	-0.02081	0.002568	5.30E-16	0.0157	0.0199	0.4303	19.80
rs73175572	G	A	0.111726	0.028208	0.003192	1.00E-18	-0.0263	0.0228	0.2484	15.50
rs73213484	T	A	0.141217	-0.0207	0.00286	4.60E-13	-0.0072	0.0207	0.7268	12.70
rs7365	G	A	0.482654	-0.01109	0.001994	2.60E-08	-0.0491	0.0146	0.000798	15.46
rs7372674	A	C	0.357244	0.014356	0.002076	4.60E-12	-0.0017	0.016	0.9167	21.97
rs743572	G	A	0.376473	0.015119	0.002057	2.00E-13	-0.0116	0.0151	0.4432	25.35
rs7442885	G	C	0.214027	-0.01987	0.002432	3.10E-16	4.00E-04	0.0188	0.9815	22.45
rs7460093	A	G	0.53118	0.013664	0.002014	1.20E-11	0.0318	0.0148	0.03231	22.92
rs74749286	A	G	0.107637	0.025896	0.003236	1.20E-15	-0.0157	0.0175	0.3691	12.30
rs7498665	G	A	0.399666	0.031786	0.002036	6.40E-55	0.0102	0.0149	0.494399	116.93
rs750090	C	T	0.35675	-0.0133	0.002101	2.50E-10	6.00E-04	0.0149	0.9699	18.38
rs7519259	A	G	0.528388	0.014144	0.002001	1.50E-12	0.019	0.0148	0.1993	24.91
rs7523668	A	G	0.565827	-0.01372	0.002009	8.60E-12	-0.0324	0.0147	0.02758	22.91
rs756717	A	G	0.399077	-0.01403	0.00206	9.70E-12	0.0161	0.015	0.2824	22.25
rs7570446	A	C	0.544459	0.011016	0.001994	3.30E-08	0.0051	0.0148	0.7278	15.13
rs7571496	G	A	0.260562	-0.01382	0.002274	1.20E-09	-0.0052	0.0151	0.7279	14.22
rs7582359	A	G	0.331712	-0.01355	0.002121	1.70E-10	-0.0206	0.0157	0.1886	18.09
rs7632381	C	T	0.444325	0.027043	0.002	1.20E-41	0.0013	0.0147	0.9313	90.26
rs765874	A	T	0.489353	-0.0168	0.00199	3.10E-17	0.0172	0.0149	0.2491	35.62
rs76647086	T	G	0.176857	-0.02022	0.002603	8.00E-15	0.0167	0.0193	0.3849	17.57
rs76798800	T	G	0.266025	0.024423	0.002253	2.20E-27	0.0168	0.0174	0.3344	45.89
rs7695177	G	C	0.470976	-0.01515	0.001995	3.10E-14	-0.015	0.0147	0.309	28.75

SNP	effect_allele HC	other_alleleHC	eaf. HC	beta.HC	se.HC	pval.HC	beta.ALRI	se. ALRI	pval.ALRI	F-statistics
rs7696175	C	T	0.526478	0.013607	0.002004	1.10E-11	-0.0225	0.0147	0.1269	22.99
rs7707394	A	G	0.357269	-0.01847	0.002075	5.50E-19	-0.0314	0.0151	0.03826	36.39
rs7708584	G	A	0.572378	-0.0132	0.002011	5.30E-11	0.0102	0.0149	0.4965	21.08
rs7740107	A	T	0.736289	-0.02365	0.002257	1.10E-25	-0.004	0.0165	0.811	42.65
rs7793674	C	A	0.144347	0.019239	0.002844	1.30E-11	0.0132	0.0278	0.635	11.31
rs7793674	C	A	0.144347	0.019239	0.002844	1.30E-11	-0.0184	0.062	0.7668	11.31
rs779655	C	G	0.729184	0.014326	0.002241	1.60E-10	0.0448	0.0157	0.004251	16.15
rs7805441	T	C	0.502255	0.012684	0.002005	2.50E-10	0.0421	0.0146	0.004025	20.01
rs7864465	A	G	0.559379	-0.01108	0.002006	3.30E-08	0.0079	0.0148	0.5954	15.04
rs7893571	T	G	0.665871	0.013838	0.002119	6.50E-11	-0.0161	0.0159	0.3134	18.98
rs7903146	T	C	0.290662	-0.02141	0.002194	1.70E-22	-0.0315	0.0183	0.085599	39.29
rs7944782	G	T	0.50981	0.012958	0.002003	9.90E-11	-0.033	0.0147	0.02502	20.91
rs7952436	T	C	0.081996	-0.03546	0.003632	1.60E-22	-0.0092	0.0332	0.7807	14.35
rs7996639	A	G	0.449371	0.013714	0.002019	1.10E-11	-0.0093	0.0147	0.526	22.84
rs8030456	T	C	0.226311	-0.02477	0.002379	2.10E-25	-0.0127	0.0199	0.5244	37.98
rs8132129	T	C	0.184901	-0.01592	0.002595	8.40E-10	-0.003	0.0179	0.8646	11.35
rs8133137	G	A	0.664097	0.015778	0.002115	8.80E-14	-0.025	0.0148	0.091829	24.82
rs815163	C	T	0.563175	-0.0169	0.002003	3.20E-17	-0.0125	0.0147	0.3929	35.03
rs852042	G	A	0.758582	-0.01537	0.00233	4.30E-11	0.0211	0.0173	0.2214	15.93
rs852983	A	G	0.459576	-0.01187	0.001997	2.80E-09	-0.0167	0.0146	0.2553	17.53
rs866006	G	T	0.618508	-0.01206	0.00205	4.00E-09	-0.0148	0.0149	0.3223	16.33
rs897186	G	A	0.548592	-0.01606	0.001999	9.20E-16	-0.0124	0.0148	0.405	31.99
rs9284814	A	G	0.884815	0.02302	0.003115	1.50E-13	-0.0333	0.023	0.1476	11.13
rs9378684	T	C	0.200589	0.018067	0.002507	5.80E-13	-0.0024	0.0164	0.8834	16.65

SNP	effect_allele HC	other_alleleHC	eaf. HC	beta.HC	se.HC	pval.HC	beta.ALRI	se. ALRI	pval.ALRI	F-statistics
rs9385385	T	C	0.447856	0.012816	0.002015	2.00E-10	-0.0169	0.0148	0.2535	20.00
rs9512696	G	A	0.66154	0.017211	0.002111	3.60E-16	-0.0031	0.0156	0.8419	29.77
rs9513018	T	G	0.616147	-0.01124	0.002054	4.50E-08	-0.0142	0.015	0.3426	14.15
rs968379	T	C	0.229098	-0.01878	0.002372	2.40E-15	-0.0024	0.0174	0.8883	22.14
rs9764678	C	T	0.272455	0.016129	0.00225	7.60E-13	0.0088	0.0156	0.5742	20.37
rs9788550	C	G	0.247466	-0.02141	0.00232	2.70E-20	-0.0039	0.0154	0.8003	31.73
rs9830592	A	C	0.582425	0.013836	0.002018	7.10E-12	0.0095	0.0152	0.5321	22.86

Abbreviations: SNP, single nucleotide polymorphism; se, standard error; HC, hip circumference; ALRI, acute lower respiratory infections; pval, p-value.

Table S15. Detailed information about single-nucleotide polymorphisms of waist circumference on sepsis susceptibility.

SNP	effect_allele WC	other_allele WC	eaf. WC	beta. WC	se. WC	pval. WC	beta.SS	se. SS	pval.SS	F-statistics
rs1013402	G	A	0.318423	0.025091	0.001917	3.90E-39	0.0169451	0.014668	0.247992	74.35
rs10150482	A	G	0.22035	0.021957	0.00217	4.50E-24	0.0146227	0.0165821	0.377865	35.20
rs10184230	T	C	0.647671	-0.01212	0.001869	8.90E-11	0.012766	0.0142937	0.371792	19.19
rs10185199	A	G	0.280698	-0.01145	0.002041	2.00E-08	-0.0382388	0.0156262	0.0144012	12.72
rs10236214	T	C	0.641963	0.013799	0.001876	1.90E-13	0.0123139	0.0143327	0.39026	24.88
rs10248298	A	C	0.366037	0.013255	0.001854	8.80E-13	0.0345463	0.0141734	0.0147928	23.72
rs1025065	G	T	0.639097	-0.01028	0.001866	3.60E-08	-0.00588473	0.0142642	0.679936	14.01
rs10257197	G	A	0.841665	-0.01548	0.002458	3.00E-10	0.000587339	0.0187768	0.975046	10.58
rs10269774	A	G	0.326456	0.01196	0.001908	3.60E-10	0.00398942	0.0145751	0.784303	17.28
rs1037702	A	G	0.621776	-0.01019	0.001849	3.60E-08	0.00270325	0.0141305	0.848286	14.28
rs10406327	G	C	0.478735	0.010362	0.001796	8.00E-09	-0.0365041	0.0136966	0.00769449	16.61
rs10423928	A	T	0.194353	-0.02656	0.002259	6.80E-32	-0.0169206	0.0172343	0.3262	43.26
rs10471636	A	G	0.508891	-0.01011	0.001824	3.00E-08	0.0060931	0.0139424	0.662096	15.36
rs10490869	T	A	0.209589	0.016339	0.002204	1.20E-13	0.0134009	0.0168669	0.426897	18.21
rs10499014	G	C	0.268616	-0.01328	0.002029	5.90E-11	-0.0099651	0.0155199	0.520817	16.84
rs1051613	A	G	0.544526	-0.00995	0.001797	3.10E-08	-0.0148436	0.0137621	0.280773	15.21
rs10732335	C	A	0.442949	-0.01474	0.001804	3.10E-16	0.00776084	0.0137747	0.573155	32.94
rs10757898	A	G	0.520113	-0.00997	0.001815	4.00E-08	-0.0229144	0.0138475	0.0979738	15.06
rs1078455	C	T	0.309501	0.011174	0.001948	9.70E-09	-0.0135877	0.0149031	0.361907	14.06
rs10787738	T	C	0.254549	0.015307	0.002086	2.10E-13	0.00493021	0.0159163	0.756746	20.44
rs10795418	G	A	0.664754	0.0127	0.001902	2.40E-11	0.0317837	0.0145218	0.0286194	19.88
rs10803762	A	G	0.677313	0.011945	0.001915	4.40E-10	0.0112317	0.0146726	0.443981	17.01
rs10827380	T	C	0.314309	0.01161	0.00193	1.80E-09	-0.0203189	0.0147477	0.168276	15.60

SNP	effect_allele WC	other_allele WC	eaf. WC	beta. WC	se. WC	pval. WC	beta.SS	se. SS	pval.SS	F-statistics
rs10835676	G	C	0.240436	0.012351	0.002098	4.00E-09	-0.0156592	0.0160163	0.32822	12.65
rs10887578	C	G	0.497514	0.010668	0.0018	3.10E-09	0.0267393	0.0137746	0.0522336	17.57
rs10938398	A	G	0.433578	0.022212	0.001808	1.00E-34	0.0191071	0.0138236	0.166908	74.18
rs10947793	G	A	0.371789	-0.01272	0.001861	8.10E-12	-0.0181871	0.0142128	0.200676	21.84
rs10992854	C	T	0.681971	-0.01125	0.001929	5.40E-09	0.0086781	0.0147405	0.556044	14.77
rs11012732	G	A	0.331675	0.019354	0.001901	2.40E-24	0.0201074	0.0145129	0.165904	45.95
rs1108548	G	A	0.277245	0.012172	0.002	1.20E-09	-0.00150637	0.0153137	0.921641	14.84
rs11099020	T	C	0.640565	-0.01124	0.001866	1.70E-09	0.00648544	0.0142713	0.649513	16.72
rs111258054	T	C	0.183665	0.015953	0.002341	9.50E-12	-0.0169102	0.0178604	0.343742	13.92
rs11150745	G	A	0.317708	-0.01608	0.001926	6.80E-17	-0.00505705	0.014695	0.730745	30.24
rs11162968	C	T	0.31619	0.012228	0.001924	2.10E-10	0.00960627	0.0147364	0.514482	17.47
rs11165493	A	G	0.343044	0.010758	0.001893	1.30E-08	0.01275	0.0144955	0.379086	14.55
rs1117619	G	C	0.250101	-0.01205	0.00206	5.00E-09	-0.0138935	0.0157716	0.378361	12.83
rs11196657	C	T	0.236715	0.012445	0.002106	3.40E-09	0.0126288	0.0160843	0.432358	12.62
rs11215381	C	T	0.525856	0.011176	0.001794	4.60E-10	0.00735096	0.013709	0.591811	19.36
rs11218510	A	G	0.40049	-0.01153	0.001829	2.90E-10	-0.0188612	0.0139758	0.177159	19.10
rs11223204	G	A	0.434214	0.012215	0.001809	1.50E-11	0.0114143	0.0138198	0.408837	22.41
rs11614326	A	G	0.545054	-0.01026	0.001817	1.60E-08	-0.0204621	0.0138741	0.140256	15.82
rs11636611	T	C	0.502804	0.010714	0.001793	2.30E-09	0.0164901	0.0136819	0.228106	17.86
rs11639596	C	A	0.250798	-0.01187	0.002078	1.10E-08	-0.0192311	0.0158895	0.226165	12.27
rs11653367	G	A	0.328129	-0.01517	0.001917	2.50E-15	-0.0015155	0.0146276	0.917483	27.60
rs11666480	G	C	0.536273	0.013289	0.001806	1.90E-13	0.0161371	0.0137841	0.241717	26.92
rs11675464	G	A	0.562997	0.011273	0.001799	3.70E-10	-0.00588376	0.0137735	0.669249	19.33
rs11704728	T	C	0.196347	0.013331	0.002264	3.90E-09	0.0229572	0.0172943	0.184362	10.94

SNP	effect_allele WC	other_allele WC	eaf. WC	beta. WC	se. WC	pval. WC	beta.SS	se. SS	pval.SS	F-statistics
rs11757278	C	T	0.303836	-0.01278	0.001944	4.90E-11	-0.00399458	0.0148653	0.788147	18.29
rs11767811	A	G	0.181372	-0.01503	0.002319	8.90E-11	-0.0192165	0.0177016	0.277665	12.48
rs11773362	T	C	0.336125	-0.01048	0.001894	3.20E-08	0.00436877	0.0145011	0.763208	13.66
rs11778934	G	C	0.536065	-0.01222	0.001799	1.10E-11	0.02861	0.0137512	0.0374757	22.93
rs11787216	T	C	0.369092	0.011505	0.001892	1.20E-09	-0.00262178	0.0144607	0.856129	17.23
rs1182199	A	C	0.3044	-0.01352	0.001945	3.60E-12	0.00991322	0.0148404	0.504142	20.47
rs11824092	C	T	0.635754	0.012491	0.001871	2.40E-11	0.0109714	0.014294	0.442753	20.65
rs11842871	T	G	0.259942	-0.01279	0.002044	4.00E-10	-0.0349672	0.0156418	0.0253852	15.05
rs1188209	G	A	0.551608	0.01024	0.001811	1.60E-08	-0.00402911	0.0138238	0.770699	15.81
rs11898037	C	T	0.367606	0.010569	0.001853	1.20E-08	0.0074145	0.0142001	0.601571	15.12
rs1191600	A	C	0.59357	-0.0108	0.001833	3.80E-09	-0.0421249	0.0140073	0.0026353	16.74
rs12001437	C	T	0.367701	0.010933	0.001855	3.80E-09	0.012298	0.0141753	0.385632	16.15
rs12072739	G	A	0.224447	0.016211	0.002143	3.90E-14	0.00546714	0.0164225	0.739205	19.92
rs12103006	G	A	0.569108	0.012478	0.001808	5.10E-12	0.0204937	0.0138072	0.137736	23.37
rs12140153	T	G	0.094226	-0.02659	0.003132	2.10E-17	0.0152555	0.0239456	0.524065	12.30
rs1218824	A	G	0.661499	0.012491	0.001893	4.20E-11	-0.00502243	0.0144656	0.728442	19.49
rs12375196	A	C	0.424364	0.013219	0.00182	3.80E-13	0.0168709	0.0138957	0.224705	25.76
rs12478299	C	T	0.252015	-0.01158	0.002062	1.90E-08	-0.0271573	0.0157828	0.085308	11.89
rs12877270	A	G	0.442217	0.011722	0.001816	1.10E-10	-0.00528489	0.013874	0.703262	20.57
rs12880641	G	T	0.661581	-0.01384	0.00189	2.40E-13	-0.0302364	0.0144006	0.0357586	24.04
rs12926311	C	G	0.353624	-0.01246	0.001877	3.20E-11	-0.0261205	0.014339	0.068511	20.13
rs1296328	C	A	0.559023	-0.01319	0.001809	3.10E-13	0.00412998	0.0138252	0.765147	26.22
rs12983532	T	C	0.251127	-0.015	0.002092	7.60E-13	0.00062966	0.0159602	0.96853	19.33
rs13033310	A	G	0.25275	0.012551	0.002068	1.30E-09	0.0187183	0.0158253	0.236886	13.92

SNP	effect_allele WC	other_allele WC	eaf. WC	beta. WC	se. WC	pval. WC	beta.SS	se. SS	pval.SS	F-statistics
rs13047416	G	C	0.37702	-0.0139	0.001854	6.60E-14	-0.0103652	0.0141525	0.463927	26.40
rs13163306	A	G	0.466047	-0.00989	0.001794	3.50E-08	0.0115473	0.0137018	0.399365	15.13
rs13182474	C	G	0.318883	-0.01172	0.001921	1.00E-09	-0.0199695	0.014668	0.173378	16.18
rs1320903	A	G	0.319725	0.017072	0.001917	5.20E-19	-0.0182786	0.0146846	0.213224	34.52
rs1321521	A	C	0.345235	0.014143	0.001879	5.10E-14	0.0114529	0.0143735	0.425563	25.62
rs1327259	G	A	0.387739	-0.01159	0.00184	3.10E-10	0.0102302	0.014081	0.46752	18.82
rs13288841	A	G	0.32166	0.01905	0.001912	2.20E-23	0.00510544	0.0146156	0.726852	43.31
rs13322435	G	A	0.404446	-0.01696	0.00183	1.90E-20	0.00613716	0.0140136	0.661427	41.38
rs13333747	C	T	0.182682	-0.02268	0.002326	1.90E-22	-0.00162624	0.0177542	0.927018	28.38
rs1336486	G	T	0.328585	0.012489	0.001908	6.00E-11	0.00215528	0.0145717	0.882415	18.90
rs13410783	G	A	0.36952	0.014106	0.00185	2.40E-14	0.0210698	0.0141784	0.137265	27.10
rs13420048	A	C	0.365011	-0.01335	0.00186	7.00E-13	-0.0183886	0.0142512	0.196942	23.89
rs13427822	G	A	0.271197	-0.01415	0.00203	3.20E-12	-0.0104937	0.0155546	0.499907	19.20
rs1346841	A	G	0.40501	-0.0106	0.001826	6.40E-09	-0.00492584	0.0139895	0.724755	16.25
rs1357079	C	T	0.570076	0.011183	0.001808	6.20E-10	0.0264409	0.0138436	0.0561371	18.76
rs1360201	T	C	0.48157	0.009794	0.001789	4.40E-08	0.0327822	0.0136642	0.0164343	14.96
rs1405261	A	T	0.434383	-0.00985	0.001805	4.80E-08	-0.0286274	0.0138342	0.0385168	14.64
rs1411432	C	A	0.186234	0.015004	0.002306	7.70E-11	0.0417265	0.0175894	0.0176799	12.83
rs1436348	G	A	0.582801	0.012493	0.001812	5.40E-12	0.00551949	0.0138615	0.690491	23.12
rs1441098	T	A	0.54446	-0.00991	0.001797	3.50E-08	-0.180053	0.296301	0.543408	15.08
rs1441098	T	A	0.54446	-0.00991	0.001797	3.50E-08	-0.0227379	0.0137579	0.0983875	15.08
rs1441264	A	G	0.593703	0.01501	0.001862	7.60E-16	-0.00859499	0.0142306	0.545857	31.35
rs1454687	G	C	0.515372	-0.01616	0.001787	1.50E-19	-0.010045	0.0136735	0.462563	40.86
rs1458156	T	C	0.488413	0.014604	0.001791	3.50E-16	-0.013501	0.0136873	0.323943	33.23

SNP	effect_allele WC	other_allele WC	eaf. WC	beta. WC	se. WC	pval. WC	beta.SS	se. SS	pval.SS	F-statistics
rs1502317	T	C	0.276578	-0.01737	0.002003	4.30E-18	0.00368617	0.0153094	0.809727	30.09
rs1559900	T	C	0.286025	0.012773	0.001979	1.10E-10	-0.011071	0.0151142	0.463868	17.02
rs156902	T	G	0.267387	-0.01382	0.002438	1.40E-08	0.0062392	0.0251099	0.803767	12.60
rs156902	T	G	0.267387	-0.01382	0.002438	1.40E-08	-0.0152546	0.0186701	0.413895	12.60
rs1570298	T	A	0.743189	0.012045	0.002045	3.80E-09	-0.000139129	0.0156358	0.9929	13.25
rs1582931	A	G	0.473264	-0.01385	0.001806	1.70E-14	-0.0178274	0.013796	0.196283	29.35
rs1609010	G	A	0.565725	0.014934	0.001806	1.30E-16	0.000489688	0.013782	0.971656	33.60
rs1609303	A	T	0.631343	0.015266	0.001858	2.10E-16	0.0318306	0.0142283	0.0252784	31.44
rs1625623	T	C	0.371988	0.010741	0.001873	9.80E-09	-0.0147444	0.0143183	0.303125	15.36
rs165656	C	G	0.517356	0.010304	0.001805	1.10E-08	-0.0129423	0.0137673	0.347179	16.27
rs1657930	A	G	0.802922	-0.01425	0.002248	2.30E-10	-0.0224452	0.0172151	0.192298	12.71
rs1711171	C	T	0.749579	0.017564	0.002065	1.80E-17	-0.0271122	0.0158017	0.0862025	27.15
rs17296856	C	A	0.280671	-0.01562	0.001996	5.00E-15	0.00829222	0.015218	0.585826	24.73
rs1731246	T	G	0.757325	-0.01184	0.002082	1.30E-08	-0.0323535	0.0159137	0.0420455	11.88
rs17446091	C	T	0.20186	0.014914	0.00223	2.30E-11	0.0177611	0.0170561	0.297719	14.42
rs1752169	A	C	0.250696	0.014308	0.002067	4.50E-12	0.00801755	0.015793	0.611688	18.00
rs17681738	T	C	0.328589	0.010542	0.001909	3.30E-08	-0.00379149	0.0146099	0.795238	13.46
rs1788808	G	A	0.494766	-0.02082	0.001793	3.60E-31	-0.0163371	0.0136997	0.233059	67.42
rs1834144	A	C	0.373194	-0.01462	0.001856	3.40E-15	0.00511551	0.0141753	0.718193	29.01
rs1861410	T	C	0.555467	-0.01601	0.001802	6.40E-19	0.0131704	0.0137943	0.339694	38.98
rs1902066	C	T	0.562316	0.010953	0.00181	1.40E-09	0.00132935	0.0138416	0.923489	18.02
rs2020942	T	C	0.394892	0.010847	0.001836	3.40E-09	0.0146118	0.0140079	0.296897	16.69
rs2133561	T	A	0.611063	-0.01224	0.001853	3.90E-11	-0.0143207	0.0141649	0.312017	20.76
rs215669	A	G	0.611654	-0.01251	0.001842	1.10E-11	-0.0106381	0.0140889	0.450207	21.90

SNP	effect_allele WC	other_allele WC	eaf. WC	beta. WC	se. WC	pval. WC	beta.SS	se. SS	pval.SS	F-statistics
rs2161097	T	C	0.437804	0.014277	0.0018	2.20E-15	0.0046993	0.0137477	0.732482	30.97
rs2172131	C	T	0.578709	-0.01217	0.001813	1.90E-11	-0.0123319	0.0138531	0.373363	21.99
rs217672	C	A	0.271758	0.012731	0.002018	2.80E-10	0.0169422	0.0154043	0.271405	15.76
rs2180454	C	T	0.771831	0.017982	0.002135	3.70E-17	0.00885425	0.0163059	0.587123	24.98
rs2183947	A	G	0.225006	-0.02208	0.002136	4.90E-25	-0.00664592	0.0163503	0.684397	37.25
rs2225909	C	T	0.774104	0.015797	0.002136	1.40E-13	0.00954515	0.016327	0.558799	19.12
rs2253310	G	C	0.626102	0.018205	0.001847	6.50E-23	0.0262588	0.0141292	0.0631001	45.48
rs2302209	T	C	0.288806	0.019833	0.001977	1.10E-23	0.0191297	0.0150921	0.204966	41.34
rs2306593	T	C	0.488298	-0.01529	0.001795	1.60E-17	-0.0267944	0.0137011	0.050508	36.27
rs2307111	C	T	0.394972	-0.02397	0.00183	3.30E-39	-0.00104154	0.013991	0.940657	82.04
rs2439823	G	A	0.545601	0.015525	0.001801	6.80E-18	0.0181334	0.0137653	0.187728	36.83
rs245767	G	A	0.730365	0.014598	0.002016	4.50E-13	0.00133485	0.0154014	0.930933	20.64
rs2470549	C	T	0.598143	-0.01195	0.001821	5.20E-11	-0.0122958	0.0139328	0.377504	20.72
rs2482704	T	G	0.426761	-0.01156	0.001806	1.50E-10	0.00262322	0.013808	0.849327	20.06
rs2568958	A	G	0.603683	0.016921	0.001822	1.60E-20	0.000632812	0.0139546	0.96383	41.28
rs2584205	A	G	0.733265	0.011159	0.002027	3.70E-08	0.0123365	0.015485	0.425641	11.86
rs2618039	T	A	0.381482	0.012078	0.00184	5.20E-11	0.0168799	0.0140799	0.230582	20.34
rs2678204	G	T	0.34017	0.015707	0.001885	7.90E-17	0.0290172	0.0144603	0.0447837	31.18
rs2696309	C	T	0.720419	0.011257	0.001993	1.60E-08	0.0274461	0.0152267	0.0714677	12.85
rs2725371	G	A	0.696098	-0.01545	0.001953	2.60E-15	-0.00528645	0.0149035	0.722806	26.48
rs2744938	G	A	0.147512	0.031969	0.002516	5.40E-37	0.029587	0.0192291	0.123888	40.61
rs28366156	C	T	0.130582	-0.01877	0.002651	1.50E-12	-0.0350368	0.0202505	0.0836007	11.37
rs28489620	A	G	0.29035	-0.01241	0.001989	4.40E-10	-0.0388031	0.0151437	0.010397	16.05
rs28580375	G	C	0.21874	0.012607	0.002168	6.00E-09	0.0144819	0.0165553	0.381703	11.56

SNP	effect_allele WC	other_allele WC	eaf. WC	beta. WC	se. WC	pval. WC	beta.SS	se. SS	pval.SS	F-statistics
rs2881479	T	A	0.14641	0.017833	0.002531	1.80E-12	-0.00409535	0.0193678	0.832535	12.41
rs2903738	T	A	0.221402	-0.01331	0.002157	6.80E-10	-0.00673907	0.0164453	0.681963	13.12
rs308911	G	A	0.714436	-0.01147	0.001981	6.90E-09	-0.004511	0.0151539	0.765947	13.70
rs3113509	T	C	0.731966	-0.01233	0.00202	1.00E-09	0.00136004	0.0154674	0.929932	14.62
rs319775	C	T	0.608753	0.010156	0.001834	3.00E-08	-0.0139067	0.0140039	0.320682	14.61
rs3212038	G	A	0.328517	0.012655	0.001911	3.50E-11	0.0100432	0.0145979	0.49146	19.35
rs34045288	T	C	0.334413	0.020375	0.001895	5.80E-27	0.0195746	0.0144824	0.176498	51.47
rs34140906	C	T	0.17033	-0.01794	0.002385	5.40E-14	-0.0157475	0.0181795	0.386368	15.99
rs34234296	A	G	0.392393	-0.01312	0.001848	1.30E-12	-0.0368777	0.0141483	0.00914724	24.03
rs34483452	A	C	0.136355	0.027048	0.002627	7.30E-25	0.0409749	0.0200678	0.041169	24.97
rs34517439	A	C	0.121789	0.030549	0.002762	1.90E-28	0.0487664	0.0211723	0.0212613	26.18
rs347551	G	C	0.472292	0.012631	0.00182	3.90E-12	-0.00495674	0.0139041	0.72147	24.01
rs34882821	T	G	0.338577	0.010624	0.001896	2.10E-08	0.0234868	0.0144864	0.104952	14.06
rs34994596	C	T	0.297289	-0.01456	0.001959	1.10E-13	-0.017376	0.0149417	0.244863	23.09
rs35243581	T	C	0.317318	0.017364	0.001923	1.70E-19	0.00935911	0.0146959	0.524222	35.35
rs35681682	C	T	0.407729	-0.01015	0.001773	1.00E-08	0.0171296	0.0135741	0.206974	15.85
rs35882248	T	C	0.317213	0.015709	0.00192	2.80E-16	0.0225208	0.0147046	0.125634	29.01
rs36061954	T	C	0.39881	0.011457	0.001827	3.60E-10	0.0168897	0.0139591	0.226303	18.87
rs36140	C	A	0.635311	0.011176	0.001867	2.10E-09	0.0129094	0.0142865	0.366203	16.61
rs36165342	C	T	0.478618	0.010712	0.001789	2.10E-09	0.0111882	0.0136793	0.413419	17.88
rs3764002	T	C	0.261552	-0.01608	0.002035	2.80E-15	0.0216749	0.0155392	0.163061	24.11
rs3768321	T	G	0.196525	0.017673	0.002248	3.80E-15	0.0338529	0.0172088	0.0491621	19.51
rs3806114	A	G	0.668366	-0.01081	0.00192	1.80E-08	0.0134432	0.014711	0.36081	14.06
rs3807566	T	G	0.438304	-0.01215	0.001806	1.70E-11	-0.0103842	0.0137874	0.451351	22.29

SNP	effect_allele WC	other_allele WC	eaf. WC	beta. WC	se. WC	pval. WC	beta.SS	se. SS	pval.SS	F-statistics
rs3814883	T	C	0.482382	0.023982	0.001795	1.10E-40	0.00926484	0.0137338	0.499929	89.12
rs3816760	A	G	0.307437	0.013354	0.001939	5.70E-12	0.00238713	0.0147994	0.871858	20.19
rs3826408	T	C	0.456788	0.011225	0.001796	4.10E-10	0.0029803	0.0137123	0.82794	19.39
rs3845344	T	C	0.391147	0.010737	0.001828	4.30E-09	0.0277623	0.0139968	0.0473151	16.42
rs3866805	A	C	0.355681	0.010421	0.00187	2.50E-08	0.0144363	0.014322	0.313464	14.23
rs3935190	A	G	0.536793	-0.01262	0.001806	2.80E-12	-0.0166636	0.0137787	0.226519	24.28
rs3936510	T	G	0.201255	0.013715	0.002227	7.30E-10	0.0123636	0.0170273	0.467775	12.20
rs3949781	A	T	0.538137	0.011512	0.001806	1.80E-10	0.000734336	0.0137947	0.957546	20.21
rs400031	G	A	0.756094	0.012086	0.002156	2.10E-08	0.0166679	0.0164956	0.312281	11.59
rs40067	A	G	0.170153	-0.01561	0.002387	6.10E-11	0.0156816	0.018238	0.389882	12.09
rs4017425	T	C	0.47017	-0.01013	0.001793	1.60E-08	-0.0111075	0.0137189	0.418142	15.90
rs4072917	A	G	0.474253	0.011719	0.001799	7.40E-11	-0.00512671	0.0137284	0.708823	21.15
rs4075353	A	G	0.344101	-0.01059	0.001892	2.20E-08	-0.00844098	0.0144508	0.559142	14.14
rs4290163	T	G	0.392697	0.011378	0.001833	5.40E-10	0.00184672	0.0140107	0.895136	18.38
rs429343	G	A	0.576576	-0.01245	0.001809	5.90E-12	-0.030448	0.0138442	0.0278542	23.12
rs429358	C	T	0.154146	-0.02714	0.002481	7.50E-28	0.0220053	0.0188995	0.24429	31.21
rs4419475	T	A	0.407376	0.011264	0.00182	6.00E-10	0.0145754	0.0139191	0.295029	18.50
rs4456769	T	C	0.333423	0.0134	0.0019	1.80E-12	0.00102665	0.0144801	0.943477	22.10
rs4469245	T	A	0.662728	-0.01152	0.001891	1.10E-09	0.000248055	0.0144442	0.986298	16.59
rs4525978	T	C	0.734555	-0.01138	0.00203	2.10E-08	0.0169147	0.0155436	0.276504	12.24
rs4527444	G	A	0.541317	0.010524	0.001794	4.50E-09	-0.0131348	0.0137253	0.338577	17.08
rs4552632	A	G	0.616568	-0.01017	0.001841	3.30E-08	-0.0104853	0.0140981	0.457038	14.42
rs4706004	G	A	0.217047	-0.01346	0.002168	5.40E-10	-0.0147495	0.0165794	0.373664	13.10
rs4742782	G	C	0.315942	0.012491	0.001922	8.00E-11	0.0253362	0.0147064	0.0849259	18.26

SNP	effect_allele WC	other_allele WC	eaf. WC	beta. WC	se. WC	pval. WC	beta.SS	se. SS	pval.SS	F-statistics
rs4790841	T	C	0.154472	-0.02126	0.002488	1.30E-17	-0.00828152	0.0189943	0.662837	19.07
rs484455	A	G	0.481363	-0.01153	0.001796	1.40E-10	-0.0218695	0.0137116	0.11072	20.58
rs4851283	G	C	0.684805	-0.0174	0.001935	2.50E-19	-0.0265403	0.0148068	0.0730634	34.88
rs4856720	C	G	0.53938	0.011434	0.001792	1.80E-10	0.00489518	0.0137056	0.720966	20.24
rs4876611	G	A	0.720239	0.015009	0.001995	5.30E-14	0.0402127	0.0152481	0.00835872	22.82
rs4900715	A	G	0.507101	-0.01143	0.001793	1.90E-10	-0.00535393	0.0136844	0.695617	20.30
rs4908672	T	C	0.393063	0.011397	0.00183	4.70E-10	-0.000612162	0.0140017	0.965127	18.50
rs520478	T	G	0.701359	-0.01251	0.001973	2.30E-10	-0.0360644	0.0150999	0.0169227	16.84
rs539515	C	A	0.204929	0.037822	0.002212	1.50E-65	0.0252615	0.0169191	0.135416	95.31
rs55726687	A	G	0.209735	0.019937	0.002195	1.10E-19	0.0139792	0.0167818	0.404846	27.34
rs557951	G	T	0.312953	0.012079	0.001932	4.00E-10	-0.00862872	0.0147735	0.559175	16.82
rs559231	T	G	0.393054	0.010752	0.001841	5.20E-09	0.0113252	0.0140572	0.420444	16.27
rs56094641	G	A	0.404591	0.057552	0.001822	1.00E-200	0.0229143	0.0139242	0.0998367	481.03
rs56803094	G	A	0.226708	-0.01277	0.002146	2.60E-09	-0.00703171	0.0163661	0.66745	12.42
rs57636386	C	T	0.083822	-0.03091	0.003241	1.50E-21	-0.0169019	0.0246731	0.493323	13.97
rs587271	T	C	0.686869	0.011805	0.002008	4.10E-09	0.0224101	0.0153599	0.144564	14.87
rs58862095	T	C	0.419271	-0.01658	0.001817	7.10E-20	-0.016511	0.0138805	0.234242	40.57
rs588660	A	G	0.584119	0.015528	0.001812	1.00E-17	0.0148011	0.0138752	0.286093	35.69
rs59068084	T	G	0.410212	0.01014	0.001819	2.50E-08	0.00957296	0.0138996	0.490998	15.03
rs59104534	T	C	0.298501	0.010719	0.001962	4.70E-08	0.00382776	0.0149819	0.798343	12.50
rs6069037	A	C	0.731389	-0.01113	0.00202	3.60E-08	0.0185431	0.0154007	0.228572	11.92
rs61223906	A	G	0.339313	-0.01087	0.001887	8.30E-09	-0.00160314	0.0143971	0.911337	14.89
rs61813324	T	C	0.135713	0.021976	0.002644	9.50E-17	0.0115144	0.0202551	0.569717	16.20

SNP	effect_allele WC	other_allele WC	eaf. WC	beta. WC	se. WC	pval. WC	beta.SS	se. SS	pval.SS	F-statistics
rs61903695	G	A	0.254942	0.013451	0.002055	5.90E-11	-0.00124158	0.0156949	0.936947	16.28
rs61969511	A	G	0.278938	0.011857	0.002013	3.80E-09	0.00244292	0.0153624	0.873654	13.96
rs62243489	G	T	0.259239	-0.01556	0.002049	3.00E-14	-0.0190249	0.0156721	0.224773	22.17
rs62261725	G	A	0.326101	-0.01487	0.001909	6.70E-15	-0.00674986	0.0146055	0.643978	26.68
rs6493498	C	T	0.54539	-0.01308	0.001805	4.20E-13	0.006436	0.0137731	0.640293	26.05
rs649458	A	T	0.860102	-0.01818	0.00257	1.50E-12	0.0112901	0.0196802	0.566187	12.05
rs6536575	C	T	0.51907	0.010843	0.00179	1.40E-09	-0.0156819	0.0136865	0.251881	18.32
rs6551304	G	A	0.831916	0.016827	0.002394	2.10E-12	0.0568664	0.0183312	0.0019211	13.82
rs6567160	C	T	0.232725	0.045164	0.002118	7.19E-101	-0.00995271	0.0161511	0.537746	162.41
rs6575340	A	G	0.636022	0.016158	0.001865	4.60E-18	0.0248041	0.0142379	0.0814892	34.76
rs6669341	G	A	0.58271	-0.01252	0.00181	4.50E-12	0.0118834	0.0138557	0.391085	23.28
rs6682438	C	T	0.673097	0.012477	0.001902	5.40E-11	-0.0186663	0.0145636	0.199944	18.94
rs6739755	G	A	0.603358	-0.01592	0.001829	3.10E-18	-0.0331821	0.0139864	0.017671	36.29
rs67609008	C	T	0.283612	0.011102	0.001992	2.50E-08	0.0214408	0.0152133	0.158732	12.62
rs6791983	A	C	0.750006	0.012315	0.00206	2.30E-09	-0.00863193	0.0157597	0.583883	13.40
rs6799080	A	G	0.353391	0.010231	0.001867	4.30E-08	0.0190321	0.0142876	0.182838	13.72
rs6846041	G	C	0.320508	0.012307	0.001915	1.30E-10	0.00632667	0.0146492	0.665831	18.00
rs6849518	T	C	0.12428	0.021864	0.00271	7.10E-16	-0.0129053	0.0207201	0.533389	14.17
rs6938973	C	T	0.601488	0.012011	0.001827	4.90E-11	0.0145299	0.0139711	0.298342	20.72
rs7034554	G	A	0.373813	-0.01126	0.001848	1.10E-09	-0.00246317	0.0141091	0.86141	17.39
rs703984	C	G	0.414809	-0.01147	0.00182	2.90E-10	-0.0151963	0.0138982	0.274216	19.30
rs704061	C	T	0.455057	0.014622	0.001797	4.00E-16	0.0242105	0.0137197	0.0776211	32.86
rs7070670	T	C	0.327941	-0.01203	0.001917	3.50E-10	0.0111143	0.0146321	0.447505	17.36

SNP	effect_allele WC	other_allele WC	eaf. WC	beta. WC	se. WC	pval. WC	beta.SS	se. SS	pval.SS	F-statistics
rs7115013	T	C	0.442738	-0.01064	0.001805	3.70E-09	0.00776694	0.0137827	0.573074	17.15
rs7132908	A	G	0.384454	0.021513	0.00184	1.40E-31	-0.0123432	0.0140582	0.379938	64.71
rs7169847	T	G	0.635678	-0.01027	0.001868	3.80E-08	0.0118828	0.0142544	0.404492	14.01
rs7171864	A	G	0.660202	0.012889	0.001899	1.10E-11	0.0159393	0.0144931	0.271428	20.67
rs7206608	G	C	0.321623	0.012	0.001916	3.80E-10	-0.00908609	0.014654	0.535231	17.11
rs7218014	C	T	0.197315	0.022409	0.002254	2.70E-23	-0.00739362	0.0171834	0.666994	31.31
rs7259070	C	T	0.596078	0.015381	0.001841	6.60E-17	0.0146818	0.014053	0.29614	33.60
rs72617140	C	A	0.213903	0.015207	0.002179	3.00E-12	0.0344789	0.0166831	0.0387623	16.38
rs72634826	A	G	0.259865	-0.0149	0.002065	5.40E-13	-0.044022	0.0157934	0.00531374	20.02
rs72892910	T	G	0.172259	0.03014	0.002371	5.20E-37	-0.00169934	0.0181597	0.925445	46.07
rs72976986	A	G	0.190131	-0.01576	0.002303	7.80E-12	-0.0149155	0.0175311	0.394879	14.42
rs73052033	C	T	0.184916	-0.02101	0.002305	7.90E-20	-0.00547851	0.0176534	0.756305	25.04
rs73068448	T	C	0.170691	-0.01534	0.002413	2.00E-10	-0.0334869	0.0184122	0.0689525	11.45
rs73142879	T	C	0.192309	-0.02419	0.002281	2.80E-26	-0.0271925	0.0174051	0.11821	34.95
rs7324067	C	T	0.761254	0.01213	0.002101	7.70E-09	0.00993517	0.0160158	0.535037	12.12
rs7372674	A	C	0.357239	0.011895	0.001863	1.70E-10	0.0199883	0.014256	0.160887	18.71
rs7377083	A	C	0.431061	0.01434	0.001819	3.20E-15	-0.0196487	0.0139013	0.157524	30.48
rs73985439	C	A	0.307294	0.012405	0.001939	1.60E-10	-9.31E-05	0.0148418	0.994996	17.42
rs7442885	G	C	0.214033	-0.02047	0.002183	6.80E-21	-0.0132707	0.0167453	0.428067	29.58
rs7498044	A	G	0.217312	-0.01515	0.002193	4.90E-12	-0.00475618	0.0167442	0.776372	16.24
rs7498665	G	A	0.399688	0.026616	0.001828	4.90E-48	0.0314728	0.0139758	0.0243254	101.79
rs7519259	A	G	0.528392	0.01267	0.001796	1.70E-12	0.021699	0.0137454	0.11442	24.80
rs7537581	A	C	0.531758	0.010736	0.001802	2.60E-09	-0.0207784	0.0138017	0.132195	17.67
rs756717	A	G	0.399071	-0.01072	0.001849	6.80E-09	0.0182678	0.0141272	0.195979	16.11

SNP	effect_allele WC	other_allele WC	eaf. WC	beta. WC	se. WC	pval. WC	beta.SS	se. SS	pval.SS	F-statistics
rs76286777	C	T	0.217783	0.023476	0.002162	1.80E-27	-0.0107514	0.0165641	0.516289	40.16
rs7630382	T	C	0.531552	0.013362	0.001793	9.30E-14	0.00542777	0.013713	0.692244	27.65
rs765876	G	A	0.489495	-0.00981	0.001788	4.10E-08	0.0125681	0.0136537	0.357316	15.04
rs7707394	A	G	0.357264	-0.01678	0.001863	2.10E-19	0.00785104	0.0142525	0.581735	37.28
rs7708584	G	A	0.572375	-0.0122	0.001805	1.40E-11	-0.0116579	0.0137972	0.398141	22.35
rs77165542	T	C	0.035495	-0.07007	0.004881	9.90E-47	-0.0437164	0.0374031	0.242487	14.11
rs784257	C	T	0.812569	0.016179	0.002306	2.30E-12	0.0414619	0.0175744	0.018313	14.99
rs7845090	A	G	0.709022	-0.01951	0.001979	6.30E-23	-0.00830167	0.0151334	0.583304	40.10
rs7925100	A	G	0.396115	0.014041	0.00183	1.70E-14	0.0270167	0.0139706	0.0531349	28.18
rs7933085	G	A	0.507544	0.010889	0.001799	1.40E-09	0.00876906	0.0137441	0.523457	18.32
rs7952436	T	C	0.081988	-0.02899	0.00326	5.90E-19	0.0361368	0.024885	0.146459	11.91
rs7966251	A	G	0.255065	-0.0116	0.002055	1.70E-08	-0.021869	0.0157167	0.16409	12.10
rs8013377	C	A	0.269606	-0.01661	0.002018	1.90E-16	0.0101347	0.0153995	0.510464	26.68
rs80243702	A	G	0.160621	0.015166	0.002456	6.70E-10	0.0185112	0.0187054	0.322361	10.28
rs8078135	T	C	0.489933	-0.01015	0.001797	1.60E-08	-0.0034027	0.0137111	0.804003	15.94
rs8097672	T	A	0.145231	0.01678	0.002554	5.00E-11	0.00581426	0.0194939	0.765503	10.72
rs8112818	G	A	0.400336	-0.01592	0.001833	3.80E-18	-0.0158413	0.0139812	0.257194	36.22
rs815163	C	T	0.563195	-0.01314	0.001798	2.70E-13	0.00707068	0.0137755	0.607754	26.27
rs8192675	C	T	0.288645	0.015992	0.001969	4.50E-16	0.0184505	0.0150647	0.220669	27.10
rs852042	G	A	0.758579	-0.0116	0.002092	3.00E-08	-0.0345422	0.0159599	0.0304411	11.26
rs852983	A	G	0.459562	-0.00983	0.001793	4.20E-08	-0.00502062	0.0136969	0.713954	14.93
rs862227	G	A	0.457951	-0.01098	0.001791	8.80E-10	0.016064	0.0136953	0.240813	18.65
rs862320	T	C	0.409625	-0.01803	0.001822	4.40E-23	-0.0347836	0.0139165	0.0124388	47.36
rs876605	G	A	0.739809	-0.01116	0.002036	4.20E-08	-0.0174313	0.0155522	0.262362	11.57

SNP	effect_allele WC	other_allele WC	eaf. WC	beta. WC	se. WC	pval. WC	beta.SS	se. SS	pval.SS	F-statistics
rs879620	T	C	0.613194	0.019443	0.001842	4.90E-26	0.0233107	0.0140868	0.097967	52.85
rs883403	C	T	0.154427	-0.01767	0.002475	9.50E-13	0.0018406	0.0189039	0.922435	13.31
rs923994	G	A	0.783206	-0.01301	0.002175	2.20E-09	-0.00737467	0.0166218	0.657278	12.16
rs9289630	C	G	0.389039	0.014476	0.001839	3.50E-15	0.00827711	0.0140763	0.556519	29.46
rs9294260	A	G	0.476584	0.013178	0.001799	2.40E-13	-0.000703818	0.0137633	0.959216	26.76
rs9308964	T	C	0.565114	-0.00996	0.001806	3.50E-08	-0.0225184	0.0138218	0.103271	14.95
rs9316661	C	T	0.801292	-0.01561	0.002247	3.80E-12	-0.0225339	0.0171615	0.189166	15.36
rs9378676	C	A	0.234005	0.013032	0.002114	7.00E-10	0.000950179	0.0161526	0.953092	13.63
rs945211	C	G	0.615598	0.010079	0.001836	4.10E-08	0.0258845	0.0140522	0.0654712	14.26
rs9568867	A	G	0.129253	0.022614	0.00269	4.20E-17	0.0242988	0.0204959	0.235801	15.91
rs9584870	C	T	0.366117	-0.01081	0.001892	1.10E-08	0.0158302	0.0144359	0.27282	15.16
rs9814758	G	T	0.355886	-0.01116	0.001875	2.70E-09	0.00362886	0.0143339	0.80014	16.22
rs9835772	T	A	0.243635	0.01213	0.002081	5.60E-09	0.0132744	0.0159197	0.404375	12.52
rs9843653	C	T	0.511657	0.019579	0.001788	6.70E-28	0.0138883	0.013692	0.310423	59.92
rs9888533	T	C	0.538081	0.010833	0.001826	3.00E-09	0.0103987	0.0139334	0.455479	17.50
rs9902846	T	C	0.316036	0.013376	0.00193	4.20E-12	0.0080229	0.0147288	0.585955	20.77
rs9916444	G	C	0.34174	0.011474	0.00189	1.30E-09	0.0112908	0.0144518	0.43464	16.58

Abbreviations: SNP, single nucleotide polymorphism; se, standard error; WC, waist circumference; SS, sepsis susceptibility; pval, p-value.

Table S16. Detailed information about single-nucleotide polymorphisms of waist circumference on sepsis mortality.

SNP	effect_allele WC	other_allele WC	eaf. WC	beta. WC	se. WC	pval. WC	beta.SM	se.SM	pval.SM	F-statistics
rs1013402	G	A	0.318423	0.025091	0.001917	3.90E-39	0.00560092	0.035741	0.875474	74.35
rs10150482	A	G	0.22035	0.021957	0.00217	4.50E-24	0.0354298	0.0405068	0.381758	35.20
rs10184230	T	C	0.647671	-0.01212	0.001869	8.90E-11	0.0235824	0.0348743	0.498906	19.19
rs10185199	A	G	0.280698	-0.01145	0.002041	2.00E-08	-0.0178804	0.0381483	0.639278	12.72
rs10236214	T	C	0.641963	0.013799	0.001876	1.90E-13	0.0275846	0.0350274	0.43098	24.88
rs10248298	A	C	0.366037	0.013255	0.001854	8.80E-13	-0.00539081	0.0345949	0.87617	23.72
rs1025065	G	T	0.639097	-0.01028	0.001866	3.60E-08	-0.0408199	0.0347341	0.23991	14.01
rs10257197	G	A	0.841665	-0.01548	0.002458	3.00E-10	-0.00946757	0.0458541	0.836423	10.58
rs10269774	A	G	0.326456	0.01196	0.001908	3.60E-10	-0.0278605	0.0355506	0.433225	17.28
rs1037702	A	G	0.621776	-0.01019	0.001849	3.60E-08	-0.0237928	0.0344567	0.489871	14.28
rs10406327	G	C	0.478735	0.010362	0.001796	8.00E-09	-0.0146628	0.033409	0.660744	16.61
rs10423928	A	T	0.194353	-0.02656	0.002259	6.80E-32	-0.0416247	0.0421491	0.323369	43.26
rs10471636	A	G	0.508891	-0.01011	0.001824	3.00E-08	0.00483721	0.0340038	0.886879	15.36
rs10490869	T	A	0.209589	0.016339	0.002204	1.20E-13	-0.0332685	0.0411306	0.418601	18.21
rs10499014	G	C	0.268616	-0.01328	0.002029	5.90E-11	0.0388572	0.0379488	0.305865	16.84
rs1051613	A	G	0.544526	-0.00995	0.001797	3.10E-08	0.0100473	0.0336418	0.765204	15.21
rs10732335	C	A	0.442949	-0.01474	0.001804	3.10E-16	0.044697	0.0336579	0.184184	32.94
rs10757898	A	G	0.520113	-0.00997	0.001815	4.00E-08	-0.024079	0.0337847	0.47602	15.06
rs1078455	C	T	0.309501	0.011174	0.001948	9.70E-09	0.0233638	0.0364428	0.521453	14.06
rs10787738	T	C	0.254549	0.015307	0.002086	2.10E-13	0.0584859	0.0389494	0.133204	20.44
rs10795418	G	A	0.664754	0.0127	0.001902	2.40E-11	0.0894295	0.0354265	0.0115907	19.88
rs10803762	A	G	0.677313	0.011945	0.001915	4.40E-10	0.0296923	0.0358373	0.407369	17.01
rs10827380	T	C	0.314309	0.01161	0.00193	1.80E-09	-0.0106584	0.0360145	0.76727	15.60

SNP	effect_allele WC	other_allele WC	eaf. WC	beta. WC	se. WC	pval. WC	beta.SM	se.SM	pval.SM	F-statistics
rs10835676	G	C	0.240436	0.012351	0.002098	4.00E-09	0.00864539	0.0390888	0.824958	12.65
rs10887578	C	G	0.497514	0.010668	0.0018	3.10E-09	-0.022374	0.0335544	0.504902	17.57
rs10938398	A	G	0.433578	0.022212	0.001808	1.00E-34	0.071239	0.033715	0.0346027	74.18
rs10947793	G	A	0.371789	-0.01272	0.001861	8.10E-12	-0.00373587	0.0347146	0.9143	21.84
rs10992854	C	T	0.681971	-0.01125	0.001929	5.40E-09	0.0176674	0.0359897	0.623495	14.77
rs11012732	G	A	0.331675	0.019354	0.001901	2.40E-24	0.0281329	0.0354145	0.426969	45.95
rs1108548	G	A	0.277245	0.012172	0.002	1.20E-09	0.0235374	0.0373586	0.528669	14.84
rs11099020	T	C	0.640565	-0.01124	0.001866	1.70E-09	-0.017363	0.0349063	0.618895	16.72
rs111258054	T	C	0.183665	0.015953	0.002341	9.50E-12	-0.0111591	0.0436096	0.798038	13.92
rs11150745	G	A	0.317708	-0.01608	0.001926	6.80E-17	0.0250219	0.0358459	0.485151	30.24
rs11162968	C	T	0.31619	0.012228	0.001924	2.10E-10	0.0152999	0.0359514	0.670419	17.47
rs11165493	A	G	0.343044	0.010758	0.001893	1.30E-08	0.0521473	0.0354252	0.14101	14.55
rs1117619	G	C	0.250101	-0.01205	0.00206	5.00E-09	-0.0200979	0.0385051	0.601701	12.83
rs11196657	C	T	0.236715	0.012445	0.002106	3.40E-09	0.0112778	0.0392244	0.773715	12.62
rs11215381	C	T	0.525856	0.011176	0.001794	4.60E-10	0.0372829	0.0334113	0.264475	19.36
rs11218510	A	G	0.40049	-0.01153	0.001829	2.90E-10	0.0028574	0.0340936	0.933207	19.10
rs11223204	G	A	0.434214	0.012215	0.001809	1.50E-11	0.0251925	0.0336919	0.454624	22.41
rs11614326	A	G	0.545054	-0.01026	0.001817	1.60E-08	-0.0416692	0.0338469	0.218282	15.82
rs11636611	T	C	0.502804	0.010714	0.001793	2.30E-09	0.0399716	0.0334096	0.231537	17.86
rs11639596	C	A	0.250798	-0.01187	0.002078	1.10E-08	0.043415	0.0388168	0.26337	12.27
rs11653367	G	A	0.328129	-0.01517	0.001917	2.50E-15	0.0563547	0.0356504	0.113933	27.60
rs11666480	G	C	0.536273	0.013289	0.001806	1.90E-13	0.0243349	0.0336416	0.46946	26.92
rs11675464	G	A	0.562997	0.011273	0.001799	3.70E-10	-0.044811	0.033596	0.182264	19.33
rs11704728	T	C	0.196347	0.013331	0.002264	3.90E-09	0.0607684	0.0422053	0.149916	10.94

SNP	effect_allele WC	other_allele WC	eaf. WC	beta. WC	se. WC	pval. WC	beta.SM	se.SM	pval.SM	F-statistics
rs11757278	C	T	0.303836	-0.01278	0.001944	4.90E-11	0.00279284	0.0363511	0.938759	18.29
rs11767811	A	G	0.181372	-0.01503	0.002319	8.90E-11	-0.0449387	0.0433163	0.299524	12.48
rs11773362	T	C	0.336125	-0.01048	0.001894	3.20E-08	-0.0231483	0.0354031	0.513209	13.66
rs11778934	G	C	0.536065	-0.01222	0.001799	1.10E-11	0.028525	0.0335573	0.395304	22.93
rs11787216	T	C	0.369092	0.011505	0.001892	1.20E-09	-0.00268984	0.035263	0.939197	17.23
rs1182199	A	C	0.3044	-0.01352	0.001945	3.60E-12	0.0440869	0.0362729	0.224205	20.47
rs11824092	C	T	0.635754	0.012491	0.001871	2.40E-11	0.0567546	0.0349268	0.104172	20.65
rs11842871	T	G	0.259942	-0.01279	0.002044	4.00E-10	-0.0462848	0.0381732	0.225323	15.05
rs1188209	G	A	0.551608	0.01024	0.001811	1.60E-08	0.034015	0.0337778	0.313924	15.81
rs11898037	C	T	0.367606	0.010569	0.001853	1.20E-08	0.0305124	0.0346384	0.378381	15.12
rs1191600	A	C	0.59357	-0.0108	0.001833	3.80E-09	-0.0130713	0.0341787	0.702134	16.74
rs12001437	C	T	0.367701	0.010933	0.001855	3.80E-09	0.0248882	0.0345504	0.471313	16.15
rs12072739	G	A	0.224447	0.016211	0.002143	3.90E-14	0.088556	0.0400173	0.0269017	19.92
rs12103006	G	A	0.569108	0.012478	0.001808	5.10E-12	0.0502276	0.033695	0.136052	23.37
rs12140153	T	G	0.094226	-0.02659	0.003132	2.10E-17	0.0302506	0.0582246	0.603377	12.30
rs1218824	A	G	0.661499	0.012491	0.001893	4.20E-11	-0.0198154	0.0353287	0.574876	19.49
rs12375196	A	C	0.424364	0.013219	0.00182	3.80E-13	0.0464441	0.0339235	0.170973	25.76
rs12478299	C	T	0.252015	-0.01158	0.002062	1.90E-08	-0.0572335	0.0385656	0.137794	11.89
rs12877270	A	G	0.442217	0.011722	0.001816	1.10E-10	-0.0587425	0.0338506	0.0826799	20.57
rs12880641	G	T	0.661581	-0.01384	0.00189	2.40E-13	-0.0462473	0.0351293	0.188011	24.04
rs12926311	C	G	0.353624	-0.01246	0.001877	3.20E-11	-0.085158	0.0349383	0.0147941	20.13
rs1296328	C	A	0.559023	-0.01319	0.001809	3.10E-13	0.0228682	0.0337378	0.497885	26.22
rs12983532	T	C	0.251127	-0.015	0.002092	7.60E-13	-0.0985003	0.0389773	0.0115003	19.33
rs13033310	A	G	0.25275	0.012551	0.002068	1.30E-09	-0.0156823	0.0386556	0.684968	13.92

SNP	effect_allele WC	other_allele WC	eaf. WC	beta. WC	se. WC	pval. WC	beta.SM	se.SM	pval.SM	F-statistics
rs13047416	G	C	0.37702	-0.0139	0.001854	6.60E-14	-0.0470903	0.0345388	0.172756	26.40
rs13163306	A	G	0.466047	-0.00989	0.001794	3.50E-08	0.0425005	0.0334308	0.203623	15.13
rs13182474	C	G	0.318883	-0.01172	0.001921	1.00E-09	-0.0027104	0.0358246	0.939692	16.18
rs1320903	A	G	0.319725	0.017072	0.001917	5.20E-19	-0.0632196	0.0358724	0.07801	34.52
rs1321521	A	C	0.345235	0.014143	0.001879	5.10E-14	0.0540446	0.0351536	0.124198	25.62
rs1327259	G	A	0.387739	-0.01159	0.00184	3.10E-10	0.0899075	0.0343545	0.00886931	18.82
rs13288841	A	G	0.32166	0.01905	0.001912	2.20E-23	-0.012816	0.0357115	0.719688	43.31
rs13322435	G	A	0.404446	-0.01696	0.00183	1.90E-20	-0.0261967	0.0342402	0.444221	41.38
rs13333747	C	T	0.182682	-0.02268	0.002326	1.90E-22	-0.0531535	0.043398	0.220654	28.38
rs1336486	G	T	0.328585	0.012489	0.001908	6.00E-11	-0.0331611	0.0355631	0.3511	18.90
rs13410783	G	A	0.36952	0.014106	0.00185	2.40E-14	0.0626339	0.0346224	0.0704417	27.10
rs13420048	A	C	0.365011	-0.01335	0.00186	7.00E-13	0.010412	0.0347676	0.764578	23.89
rs13427822	G	A	0.271197	-0.01415	0.00203	3.20E-12	-0.0544364	0.037959	0.151548	19.20
rs1346841	A	G	0.40501	-0.0106	0.001826	6.40E-09	-0.0439708	0.0341504	0.197897	16.25
rs1357079	C	T	0.570076	0.011183	0.001808	6.20E-10	-0.00438447	0.0337318	0.896582	18.76
rs1360201	T	C	0.48157	0.009794	0.001789	4.40E-08	0.0216521	0.0333598	0.516307	14.96
rs1405261	A	T	0.434383	-0.00985	0.001805	4.80E-08	-0.039511	0.0337686	0.24198	14.64
rs1411432	C	A	0.186234	0.015004	0.002306	7.70E-11	0.0836486	0.0429018	0.0512035	12.83
rs1436348	G	A	0.582801	0.012493	0.001812	5.40E-12	0.0277657	0.0338099	0.411515	23.12
rs1441098	T	A	0.54446	-0.00991	0.001797	3.50E-08	-0.949589	0.738654	0.198594	15.08
rs1441098	T	A	0.54446	-0.00991	0.001797	3.50E-08	-0.0453754	0.033573	0.176522	15.08
rs1441264	A	G	0.593703	0.01501	0.001862	7.60E-16	0.0122693	0.0346988	0.723644	31.35
rs1454687	G	C	0.515372	-0.01616	0.001787	1.50E-19	-0.0160744	0.0333768	0.630086	40.86
rs1458156	T	C	0.488413	0.014604	0.001791	3.50E-16	-0.0180282	0.0333744	0.589071	33.23

SNP	effect_allele WC	other_allele WC	eaf. WC	beta. WC	se. WC	pval. WC	beta.SM	se.SM	pval.SM	F-statistics
rs1502317	T	C	0.276578	-0.01737	0.002003	4.30E-18	0.0491089	0.0373947	0.189095	30.09
rs1559900	T	C	0.286025	0.012773	0.001979	1.10E-10	-0.0192645	0.0368413	0.601041	17.02
rs156902	T	G	0.267387	-0.01382	0.002438	1.40E-08	-0.0286217	0.045607	0.530285	12.60
rs156902	T	G	0.267387	-0.01382	0.002438	1.40E-08	0.0091387	0.0613875	0.881657	12.60
rs1570298	T	A	0.743189	0.012045	0.002045	3.80E-09	0.048224	0.0381831	0.206601	13.25
rs1582931	A	G	0.473264	-0.01385	0.001806	1.70E-14	-0.0313812	0.0336846	0.351535	29.35
rs1609010	G	A	0.565725	0.014934	0.001806	1.30E-16	-0.00465167	0.0336659	0.890105	33.60
rs1609303	A	T	0.631343	0.015266	0.001858	2.10E-16	-0.00776544	0.0347175	0.82301	31.44
rs1625623	T	C	0.371988	0.010741	0.001873	9.80E-09	0.027003	0.0349604	0.439885	15.36
rs165656	C	G	0.517356	0.010304	0.001805	1.10E-08	0.0578881	0.033596	0.084877	16.27
rs1657930	A	G	0.802922	-0.01425	0.002248	2.30E-10	-0.0161058	0.0419063	0.700735	12.71
rs1711171	C	T	0.749579	0.017564	0.002065	1.80E-17	0.0123046	0.0385798	0.749773	27.15
rs17296856	C	A	0.280671	-0.01562	0.001996	5.00E-15	-0.00289368	0.0371556	0.937923	24.73
rs1731246	T	G	0.757325	-0.01184	0.002082	1.30E-08	-0.0228044	0.0388793	0.557509	11.88
rs17446091	C	T	0.20186	0.014914	0.00223	2.30E-11	0.0154987	0.0416306	0.709676	14.42
rs1752169	A	C	0.250696	0.014308	0.002067	4.50E-12	-0.0684624	0.0384506	0.0749894	18.00
rs17681738	T	C	0.328589	0.010542	0.001909	3.30E-08	-0.0306784	0.035649	0.389474	13.46
rs1788808	G	A	0.494766	-0.02082	0.001793	3.60E-31	-0.03116	0.0334161	0.351086	67.42
rs1834144	A	C	0.373194	-0.01462	0.001856	3.40E-15	0.00215979	0.0345895	0.950212	29.01
rs1861410	T	C	0.555467	-0.01601	0.001802	6.40E-19	0.001564	0.0336785	0.96296	38.98
rs1902066	C	T	0.562316	0.010953	0.00181	1.40E-09	-0.00482351	0.0337581	0.886381	18.02
rs2020942	T	C	0.394892	0.010847	0.001836	3.40E-09	0.0325811	0.0341847	0.340545	16.69
rs2133561	T	A	0.611063	-0.01224	0.001853	3.90E-11	-0.0524033	0.0345815	0.129681	20.76
rs215669	A	G	0.611654	-0.01251	0.001842	1.10E-11	-0.0212551	0.0343938	0.536581	21.90

SNP	effect_allele WC	other_allele WC	eaf. WC	beta. WC	se. WC	pval. WC	beta.SM	se.SM	pval.SM	F-statistics
rs2161097	T	C	0.437804	0.014277	0.0018	2.20E-15	0.0103745	0.0335611	0.757229	30.97
rs2172131	C	T	0.578709	-0.01217	0.001813	1.90E-11	0.00188983	0.0338009	0.955413	21.99
rs217672	C	A	0.271758	0.012731	0.002018	2.80E-10	0.0194905	0.0376544	0.604728	15.76
rs2180454	C	T	0.771831	0.017982	0.002135	3.70E-17	0.0613039	0.0397894	0.123387	24.98
rs2183947	A	G	0.225006	-0.02208	0.002136	4.90E-25	-0.0221545	0.0399236	0.578949	37.25
rs2225909	C	T	0.774104	0.015797	0.002136	1.40E-13	-0.0145354	0.039788	0.714873	19.12
rs2253310	G	C	0.626102	0.018205	0.001847	6.50E-23	0.0196752	0.0345224	0.568727	45.48
rs2302209	T	C	0.288806	0.019833	0.001977	1.10E-23	0.0402232	0.036843	0.274944	41.34
rs2306593	T	C	0.488298	-0.01529	0.001795	1.60E-17	-0.0233838	0.0334176	0.484087	36.27
rs2307111	C	T	0.394972	-0.02397	0.00183	3.30E-39	-0.007442	0.0341518	0.8275	82.04
rs2439823	G	A	0.545601	0.015525	0.001801	6.80E-18	-0.0167662	0.0336073	0.61786	36.83
rs245767	G	A	0.730365	0.014598	0.002016	4.50E-13	0.00480757	0.0376501	0.898393	20.64
rs2470549	C	T	0.598143	-0.01195	0.001821	5.20E-11	0.0383651	0.0340144	0.259358	20.72
rs2482704	T	G	0.426761	-0.01156	0.001806	1.50E-10	-0.0215965	0.0337197	0.521867	20.06
rs2568958	A	G	0.603683	0.016921	0.001822	1.60E-20	0.00136011	0.0341105	0.968194	41.28
rs2584205	A	G	0.733265	0.011159	0.002027	3.70E-08	-0.00164125	0.0377645	0.965335	11.86
rs2618039	T	A	0.381482	0.012078	0.00184	5.20E-11	-0.00368701	0.0343829	0.914603	20.34
rs2678204	G	T	0.34017	0.015707	0.001885	7.90E-17	0.0111338	0.0352623	0.752199	31.18
rs2696309	C	T	0.720419	0.011257	0.001993	1.60E-08	0.0643356	0.037141	0.0832377	12.85
rs2725371	G	A	0.696098	-0.01545	0.001953	2.60E-15	0.0146524	0.0363531	0.686906	26.48
rs2744938	G	A	0.147512	0.031969	0.002516	5.40E-37	0.0411976	0.0469313	0.380038	40.61
rs28366156	C	T	0.130582	-0.01877	0.002651	1.50E-12	-0.0489375	0.0495786	0.323609	11.37
rs28489620	A	G	0.29035	-0.01241	0.001989	4.40E-10	-0.0639655	0.0369845	0.0837163	16.05
rs28580375	G	C	0.21874	0.012607	0.002168	6.00E-09	0.0456897	0.0403158	0.25709	11.56

SNP	effect_allele WC	other_allele WC	eaf. WC	beta. WC	se. WC	pval. WC	beta.SM	se.SM	pval.SM	F-statistics
rs2881479	T	A	0.14641	0.017833	0.002531	1.80E-12	0.0289444	0.047405	0.541479	12.41
rs2903738	T	A	0.221402	-0.01331	0.002157	6.80E-10	0.0219335	0.040171	0.585065	13.12
rs308911	G	A	0.714436	-0.01147	0.001981	6.90E-09	0.0210466	0.0369702	0.569163	13.70
rs3113509	T	C	0.731966	-0.01233	0.00202	1.00E-09	0.0385167	0.0378218	0.308501	14.62
rs319775	C	T	0.608753	0.010156	0.001834	3.00E-08	-0.0112419	0.0341682	0.742144	14.61
rs3212038	G	A	0.328517	0.012655	0.001911	3.50E-11	-0.0185683	0.0355762	0.601718	19.35
rs34045288	T	C	0.334413	0.020375	0.001895	5.80E-27	0.0424922	0.0353097	0.228817	51.47
rs34140906	C	T	0.17033	-0.01794	0.002385	5.40E-14	-0.134807	0.0443386	0.00236271	15.99
rs34234296	A	G	0.392393	-0.01312	0.001848	1.30E-12	-0.0711551	0.0345116	0.0392292	24.03
rs34483452	A	C	0.136355	0.027048	0.002627	7.30E-25	0.0733945	0.0490038	0.134204	24.97
rs34517439	A	C	0.121789	0.030549	0.002762	1.90E-28	0.073349	0.0516852	0.155856	26.18
rs347551	G	C	0.472292	0.012631	0.00182	3.90E-12	-0.025047	0.0339562	0.460742	24.01
rs34882821	T	G	0.338577	0.010624	0.001896	2.10E-08	0.0585303	0.0353862	0.0981183	14.06
rs34994596	C	T	0.297289	-0.01456	0.001959	1.10E-13	-0.0388912	0.0364714	0.286266	23.09
rs35243581	T	C	0.317318	0.017364	0.001923	1.70E-19	0.0365231	0.0358766	0.308668	35.35
rs35681682	C	T	0.407729	-0.01015	0.001773	1.00E-08	0.0197077	0.0331545	0.55223	15.85
rs35882248	T	C	0.317213	0.015709	0.00192	2.80E-16	0.0526144	0.0358573	0.142287	29.01
rs36061954	T	C	0.39881	0.011457	0.001827	3.60E-10	-0.00931603	0.034083	0.784596	18.87
rs36140	C	A	0.635311	0.011176	0.001867	2.10E-09	0.0324323	0.0349337	0.353201	16.61
rs36165342	C	T	0.478618	0.010712	0.001789	2.10E-09	0.0179762	0.0334044	0.590481	17.88
rs3764002	T	C	0.261552	-0.01608	0.002035	2.80E-15	0.0225889	0.0379322	0.551504	24.11
rs3768321	T	G	0.196525	0.017673	0.002248	3.80E-15	0.0412135	0.0419112	0.325434	19.51
rs3806114	A	G	0.668366	-0.01081	0.00192	1.80E-08	-0.0112459	0.0358483	0.753742	14.06
rs3807566	T	G	0.438304	-0.01215	0.001806	1.70E-11	0.0262914	0.0335797	0.433654	22.29

SNP	effect_allele WC	other_allele WC	eaf. WC	beta. WC	se. WC	pval. WC	beta.SM	se.SM	pval.SM	F-statistics
rs3814883	T	C	0.482382	0.023982	0.001795	1.10E-40	-0.0133553	0.0335021	0.690159	89.12
rs3816760	A	G	0.307437	0.013354	0.001939	5.70E-12	-0.0442561	0.0361675	0.221086	20.19
rs3826408	T	C	0.456788	0.011225	0.001796	4.10E-10	-0.0181588	0.0334584	0.587316	19.39
rs3845344	T	C	0.391147	0.010737	0.001828	4.30E-09	0.0427948	0.0340999	0.209485	16.42
rs3866805	A	C	0.355681	0.010421	0.00187	2.50E-08	-0.036095	0.0349551	0.301786	14.23
rs3935190	A	G	0.536793	-0.01262	0.001806	2.80E-12	-0.00882247	0.0336142	0.792965	24.28
rs3936510	T	G	0.201255	0.013715	0.002227	7.30E-10	0.0641379	0.0416311	0.123408	12.20
rs3949781	A	T	0.538137	0.011512	0.001806	1.80E-10	0.0121781	0.0336047	0.717058	20.21
rs400031	G	A	0.756094	0.012086	0.002156	2.10E-08	0.0150941	0.0402601	0.707724	11.59
rs40067	A	G	0.170153	-0.01561	0.002387	6.10E-11	0.00028524	0.0444916	0.994885	12.09
rs4017425	T	C	0.47017	-0.01013	0.001793	1.60E-08	-0.0315945	0.0334968	0.345573	15.90
rs4072917	A	G	0.474253	0.011719	0.001799	7.40E-11	0.021698	0.033517	0.51739	21.15
rs4075353	A	G	0.344101	-0.01059	0.001892	2.20E-08	0.0272341	0.0353474	0.441022	14.14
rs4290163	T	G	0.392697	0.011378	0.001833	5.40E-10	-0.0195131	0.0342374	0.568722	18.38
rs429343	G	A	0.576576	-0.01245	0.001809	5.90E-12	-0.0395551	0.0338479	0.24256	23.12
rs429358	C	T	0.154146	-0.02714	0.002481	7.50E-28	0.0706299	0.0461037	0.125528	31.21
rs4419475	T	A	0.407376	0.011264	0.00182	6.00E-10	-0.00433242	0.0340197	0.898663	18.50
rs4456769	T	C	0.333423	0.0134	0.0019	1.80E-12	0.0254283	0.0353229	0.471597	22.10
rs4469245	T	A	0.662728	-0.01152	0.001891	1.10E-09	0.0165214	0.0352906	0.639674	16.59
rs4525978	T	C	0.734555	-0.01138	0.00203	2.10E-08	-0.0108042	0.037991	0.776113	12.24
rs4527444	G	A	0.541317	0.010524	0.001794	4.50E-09	-0.0316423	0.0334839	0.344659	17.08
rs4552632	A	G	0.616568	-0.01017	0.001841	3.30E-08	-0.0499942	0.0344269	0.146451	14.42
rs4706004	G	A	0.217047	-0.01346	0.002168	5.40E-10	0.0265074	0.0404825	0.512605	13.10
rs4742782	G	C	0.315942	0.012491	0.001922	8.00E-11	0.0177373	0.0358633	0.620895	18.26

SNP	effect_allele WC	other_allele WC	eaf. WC	beta. WC	se. WC	pval. WC	beta.SM	se.SM	pval.SM	F-statistics
rs4790841	T	C	0.154472	-0.02126	0.002488	1.30E-17	-0.0281816	0.0462146	0.541995	19.07
rs484455	A	G	0.481363	-0.01153	0.001796	1.40E-10	0.0271085	0.0334449	0.41763	20.58
rs4851283	G	C	0.684805	-0.0174	0.001935	2.50E-19	0.029849	0.036141	0.408858	34.88
rs4856720	C	G	0.53938	0.011434	0.001792	1.80E-10	0.016306	0.0334662	0.626089	20.24
rs4876611	G	A	0.720239	0.015009	0.001995	5.30E-14	0.101413	0.03718	0.00637911	22.82
rs4900715	A	G	0.507101	-0.01143	0.001793	1.90E-10	0.0287371	0.0334452	0.390215	20.30
rs4908672	T	C	0.393063	0.011397	0.00183	4.70E-10	-0.0220951	0.0342016	0.518263	18.50
rs520478	T	G	0.701359	-0.01251	0.001973	2.30E-10	0.0346853	0.0368996	0.347221	16.84
rs539515	C	A	0.204929	0.037822	0.002212	1.50E-65	0.00559748	0.041315	0.89223	95.31
rs55726687	A	G	0.209735	0.019937	0.002195	1.10E-19	-0.0603978	0.040926	0.140003	27.34
rs557951	G	T	0.312953	0.012079	0.001932	4.00E-10	0.0158815	0.036011	0.6592	16.82
rs559231	T	G	0.393054	0.010752	0.001841	5.20E-09	0.00785735	0.0342753	0.818681	16.27
rs56094641	G	A	0.404591	0.057552	0.001822	1.00E-200	0.0351196	0.0339399	0.300782	481.03
rs56803094	G	A	0.226708	-0.01277	0.002146	2.60E-09	-0.0944776	0.0398762	0.017823	12.42
rs57636386	C	T	0.083822	-0.03091	0.003241	1.50E-21	-0.0747878	0.0601015	0.213368	13.97
rs587271	T	C	0.686869	0.011805	0.002008	4.10E-09	0.0278518	0.0375158	0.457843	14.87
rs58862095	T	C	0.419271	-0.01658	0.001817	7.10E-20	0.0817249	0.0338539	0.0157765	40.57
rs588660	A	G	0.584119	0.015528	0.001812	1.00E-17	0.0614519	0.0338685	0.0696113	35.69
rs59068084	T	G	0.410212	0.01014	0.001819	2.50E-08	-0.00782858	0.0339694	0.817734	15.03
rs59104534	T	C	0.298501	0.010719	0.001962	4.70E-08	0.0253616	0.0365295	0.487509	12.50
rs6069037	A	C	0.731389	-0.01113	0.00202	3.60E-08	-0.00236215	0.0375499	0.949841	11.92
rs61223906	A	G	0.339313	-0.01087	0.001887	8.30E-09	-0.0139258	0.0352178	0.692534	14.89
rs61813324	T	C	0.135713	0.021976	0.002644	9.50E-17	0.0154191	0.049427	0.755073	16.20

SNP	effect_allele WC	other_allele WC	eaf. WC	beta. WC	se. WC	pval. WC	beta.SM	se.SM	pval.SM	F-statistics
rs61903695	G	A	0.254942	0.013451	0.002055	5.90E-11	0.0569912	0.038358	0.137339	16.28
rs61969511	A	G	0.278938	0.011857	0.002013	3.80E-09	0.0321689	0.0375234	0.391279	13.96
rs62243489	G	T	0.259239	-0.01556	0.002049	3.00E-14	-0.0335151	0.0383045	0.381594	22.17
rs62261725	G	A	0.326101	-0.01487	0.001909	6.70E-15	-0.00806379	0.0356691	0.821145	26.68
rs6493498	C	T	0.54539	-0.01308	0.001805	4.20E-13	0.0177188	0.0336027	0.597985	26.05
rs649458	A	T	0.860102	-0.01818	0.00257	1.50E-12	0.00683995	0.0480461	0.886794	12.05
rs6536575	C	T	0.51907	0.010843	0.00179	1.40E-09	0.00230459	0.0334203	0.945023	18.32
rs6551304	G	A	0.831916	0.016827	0.002394	2.10E-12	0.0385923	0.0448131	0.389137	13.82
rs6567160	C	T	0.232725	0.045164	0.002118	7.19E-101	-0.0383816	0.0393246	0.329055	162.41
rs6575340	A	G	0.636022	0.016158	0.001865	4.60E-18	0.0267563	0.0347512	0.441336	34.76
rs6669341	G	A	0.58271	-0.01252	0.00181	4.50E-12	-0.0259024	0.0337909	0.44335	23.28
rs6682438	C	T	0.673097	0.012477	0.001902	5.40E-11	-0.0773785	0.0355391	0.0294598	18.94
rs6739755	G	A	0.603358	-0.01592	0.001829	3.10E-18	-0.0250168	0.0341971	0.464445	36.29
rs67609008	C	T	0.283612	0.011102	0.001992	2.50E-08	0.0561796	0.0371439	0.130411	12.62
rs6791983	A	C	0.750006	0.012315	0.00206	2.30E-09	-0.0213417	0.0384339	0.578701	13.40
rs6799080	A	G	0.353391	0.010231	0.001867	4.30E-08	0.00599276	0.0348794	0.863584	13.72
rs6846041	G	C	0.320508	0.012307	0.001915	1.30E-10	0.0102119	0.0357491	0.775143	18.00
rs6849518	T	C	0.12428	0.021864	0.00271	7.10E-16	0.0203997	0.0504781	0.686117	14.17
rs6938973	C	T	0.601488	0.012011	0.001827	4.90E-11	0.061804	0.0341067	0.0699745	20.72
rs7034554	G	A	0.373813	-0.01126	0.001848	1.10E-09	0.0104707	0.0344211	0.76098	17.39
rs703984	C	G	0.414809	-0.01147	0.00182	2.90E-10	-0.0493399	0.0338894	0.145417	19.30
rs704061	C	T	0.455057	0.014622	0.001797	4.00E-16	0.0244648	0.0335006	0.465219	32.86
rs7070670	T	C	0.327941	-0.01203	0.001917	3.50E-10	0.0237653	0.035728	0.50594	17.36

SNP	effect_allele WC	other_allele WC	eaf. WC	beta. WC	se. WC	pval. WC	beta.SM	se.SM	pval.SM	F-statistics
rs7115013	T	C	0.442738	-0.01064	0.001805	3.70E-09	0.0144506	0.0336473	0.667578	17.15
rs7132908	A	G	0.384454	0.021513	0.00184	1.40E-31	-0.00805489	0.0343471	0.814586	64.71
rs7169847	T	G	0.635678	-0.01027	0.001868	3.80E-08	0.0169616	0.0348491	0.62646	14.01
rs7171864	A	G	0.660202	0.012889	0.001899	1.10E-11	0.0123822	0.0354162	0.726624	20.67
rs7206608	G	C	0.321623	0.012	0.001916	3.80E-10	-0.0291263	0.0358036	0.415931	17.11
rs7218014	C	T	0.197315	0.022409	0.002254	2.70E-23	-0.0520651	0.0421363	0.216594	31.31
rs7259070	C	T	0.596078	0.015381	0.001841	6.60E-17	0.0309083	0.0343096	0.367661	33.60
rs72617140	C	A	0.213903	0.015207	0.002179	3.00E-12	0.0142813	0.0407668	0.726099	16.38
rs72634826	A	G	0.259865	-0.0149	0.002065	5.40E-13	-0.0699561	0.0385192	0.069349	20.02
rs72892910	T	G	0.172259	0.03014	0.002371	5.20E-37	-0.104811	0.0443088	0.0180078	46.07
rs72976986	A	G	0.190131	-0.01576	0.002303	7.80E-12	-0.0453821	0.0427732	0.288693	14.42
rs73052033	C	T	0.184916	-0.02101	0.002305	7.90E-20	-0.0181331	0.0430904	0.67389	25.04
rs73068448	T	C	0.170691	-0.01534	0.002413	2.00E-10	-0.104484	0.0449027	0.019971	11.45
rs73142879	T	C	0.192309	-0.02419	0.002281	2.80E-26	0.00631023	0.0424291	0.881771	34.95
rs7324067	C	T	0.761254	0.01213	0.002101	7.70E-09	0.0685586	0.0391539	0.0799448	12.12
rs7372674	A	C	0.357239	0.011895	0.001863	1.70E-10	0.00419379	0.0347755	0.904011	18.71
rs7377083	A	C	0.431061	0.01434	0.001819	3.20E-15	0.0140704	0.0339746	0.678767	30.48
rs73985439	C	A	0.307294	0.012405	0.001939	1.60E-10	-0.0307663	0.0362236	0.395691	17.42
rs7442885	G	C	0.214033	-0.02047	0.002183	6.80E-21	-0.0411031	0.0409055	0.314979	29.58
rs7498044	A	G	0.217312	-0.01515	0.002193	4.90E-12	0.0605524	0.0408844	0.138589	16.24
rs7498665	G	A	0.399688	0.026616	0.001828	4.90E-48	0.0241258	0.0341061	0.479334	101.79
rs7519259	A	G	0.528392	0.01267	0.001796	1.70E-12	0.0799664	0.0335995	0.0173133	24.80
rs7537581	A	C	0.531758	0.010736	0.001802	2.60E-09	0.0377406	0.0336954	0.262692	17.67
rs756717	A	G	0.399071	-0.01072	0.001849	6.80E-09	0.0181077	0.0344458	0.599105	16.11

SNP	effect_allele WC	other_allele WC	eaf. WC	beta. WC	se. WC	pval. WC	beta.SM	se.SM	pval.SM	F-statistics
rs76286777	C	T	0.217783	0.023476	0.002162	1.80E-27	-0.0294427	0.0404462	0.466646	40.16
rs7630382	T	C	0.531552	0.013362	0.001793	9.30E-14	-0.0243243	0.0334897	0.467641	27.65
rs765876	G	A	0.489495	-0.00981	0.001788	4.10E-08	0.0221219	0.0333376	0.506964	15.04
rs7707394	A	G	0.357264	-0.01678	0.001863	2.10E-19	0.00675688	0.0347917	0.846012	37.28
rs7708584	G	A	0.572375	-0.0122	0.001805	1.40E-11	-0.0319617	0.033636	0.341998	22.35
rs77165542	T	C	0.035495	-0.07007	0.004881	9.90E-47	-0.0957122	0.0908096	0.291888	14.11
rs784257	C	T	0.812569	0.016179	0.002306	2.30E-12	-0.00850319	0.0428753	0.842792	14.99
rs7845090	A	G	0.709022	-0.01951	0.001979	6.30E-23	0.0387171	0.0369301	0.294458	40.10
rs7925100	A	G	0.396115	0.014041	0.00183	1.70E-14	0.046496	0.0341006	0.172727	28.18
rs7933085	G	A	0.507544	0.010889	0.001799	1.40E-09	0.065093	0.0335591	0.0524216	18.32
rs7952436	T	C	0.081988	-0.02899	0.00326	5.90E-19	0.0376648	0.0608917	0.53621	11.91
rs7966251	A	G	0.255065	-0.0116	0.002055	1.70E-08	0.0642822	0.0384044	0.0941651	12.10
rs8013377	C	A	0.269606	-0.01661	0.002018	1.90E-16	0.0724776	0.037686	0.0544553	26.68
rs80243702	A	G	0.160621	0.015166	0.002456	6.70E-10	0.0981149	0.0457025	0.0318083	10.28
rs8078135	T	C	0.489933	-0.01015	0.001797	1.60E-08	-0.0240099	0.0334488	0.472874	15.94
rs8097672	T	A	0.145231	0.01678	0.002554	5.00E-11	-0.0446381	0.0475021	0.347367	10.72
rs8112818	G	A	0.400336	-0.01592	0.001833	3.80E-18	-0.0211437	0.0342169	0.536621	36.22
rs815163	C	T	0.563195	-0.01314	0.001798	2.70E-13	-0.00529718	0.0336466	0.874902	26.27
rs8192675	C	T	0.288645	0.015992	0.001969	4.50E-16	0.0112581	0.0367381	0.759268	27.10
rs852042	G	A	0.758579	-0.0116	0.002092	3.00E-08	-0.0284438	0.0389941	0.465734	11.26
rs852983	A	G	0.459562	-0.00983	0.001793	4.20E-08	-0.00379523	0.0334158	0.909574	14.93
rs862227	G	A	0.457951	-0.01098	0.001791	8.80E-10	0.0226141	0.0334026	0.498395	18.65
rs862320	T	C	0.409625	-0.01803	0.001822	4.40E-23	-0.0381562	0.0339525	0.261092	47.36
rs876605	G	A	0.739809	-0.01116	0.002036	4.20E-08	-0.0515328	0.0379679	0.174694	11.57

SNP	effect_allele WC	other_allele WC	eaf. WC	beta. WC	se. WC	pval. WC	beta.SM	se.SM	pval.SM	F-statistics
rs879620	T	C	0.613194	0.019443	0.001842	4.90E-26	-0.0102857	0.0343592	0.764668	52.85
rs883403	C	T	0.154427	-0.01767	0.002475	9.50E-13	0.0154989	0.0461965	0.737249	13.31
rs923994	G	A	0.783206	-0.01301	0.002175	2.20E-09	-0.00093424	0.0405584	0.981623	12.16
rs9289630	C	G	0.389039	0.014476	0.001839	3.50E-15	0.0200714	0.0343132	0.558583	29.46
rs9294260	A	G	0.476584	0.013178	0.001799	2.40E-13	0.00645661	0.0335778	0.847516	26.76
rs9308964	T	C	0.565114	-0.00996	0.001806	3.50E-08	-0.0159998	0.0337093	0.635044	14.95
rs9316661	C	T	0.801292	-0.01561	0.002247	3.80E-12	0.00500727	0.0418274	0.904711	15.36
rs9378676	C	A	0.234005	0.013032	0.002114	7.00E-10	0.00790289	0.0394176	0.841096	13.63
rs945211	C	G	0.615598	0.010079	0.001836	4.10E-08	0.0153512	0.0343291	0.654748	14.26
rs9568867	A	G	0.129253	0.022614	0.00269	4.20E-17	0.0447834	0.0499561	0.37001	15.91
rs9584870	C	T	0.366117	-0.01081	0.001892	1.10E-08	0.0271387	0.0352378	0.441207	15.16
rs9814758	G	T	0.355886	-0.01116	0.001875	2.70E-09	-0.0106427	0.0350062	0.761111	16.22
rs9835772	T	A	0.243635	0.01213	0.002081	5.60E-09	0.0262616	0.0388062	0.498573	12.52
rs9843653	C	T	0.511657	0.019579	0.001788	6.70E-28	-0.0270878	0.0334392	0.417904	59.92
rs9888533	T	C	0.538081	0.010833	0.001826	3.00E-09	0.00630954	0.034045	0.852971	17.50
rs9902846	T	C	0.316036	0.013376	0.00193	4.20E-12	0.070614	0.035933	0.0493958	20.77
rs9916444	G	C	0.34174	0.011474	0.00189	1.30E-09	0.0490083	0.0352538	0.164482	16.58

Abbreviations: SNP, single nucleotide polymorphism; se, standard error; pval, p-value; WC, waist circumference; SM, sepsis mortality.

Table S17. Detailed information about single-nucleotide polymorphisms of waist circumference on cholecystitis.

SNP	effect_allele WC	other_allele WC	eaf. WC	beta. WC	se. WC	pval. WC	beta. cholecystitis	se. cholecystitis	pval. cholecystitis	F-statistics
rs10150482	A	G	0.22035	0.021957	0.00217	4.50E-24	0.0197	0.0137	0.1528	35.20
rs10248298	A	C	0.366037	0.013255	0.001854	8.80E-13	0.0145	0.0121	0.2304	23.72
rs1025065	G	T	0.639097	-0.01028	0.001866	3.60E-08	0.0156	0.0129	0.2239	14.01
rs10257197	G	A	0.841665	-0.01548	0.002458	3.00E-10	0.0207	0.0148	0.1629	10.58
rs10269774	A	G	0.326456	0.01196	0.001908	3.60E-10	5.00E-04	0.0127	0.9669	17.28
rs1037702	A	G	0.621776	-0.01019	0.001849	3.60E-08	-0.0041	0.0121	0.732799	14.28
rs10406327	G	C	0.478735	0.010362	0.001796	8.00E-09	0.0098	0.0118	0.4065	16.61
rs10471636	A	G	0.508891	-0.01011	0.001824	3.00E-08	0.0038	0.0118	0.7471	15.36
rs10490869	T	A	0.209589	0.016339	0.002204	1.20E-13	5.00E-04	0.0142	0.9742	18.21
rs10499014	G	C	0.268616	-0.01328	0.002029	5.90E-11	-0.0232	0.0134	0.08209	16.84
rs1051613	A	G	0.544526	-0.00995	0.001797	3.10E-08	0.0155	0.0118	0.1907	15.21
rs1056441	C	T	0.675421	0.012706	0.001913	3.10E-11	-0.0191	0.0134	0.1549	19.33
rs10732335	C	A	0.442949	-0.01474	0.001804	3.10E-16	-0.0181	0.0118	0.1233	32.94
rs10757898	A	G	0.520113	-0.00997	0.001815	4.00E-08	-0.0135	0.0118	0.2539	15.06
rs1078455	C	T	0.309501	0.011174	0.001948	9.70E-09	-0.0071	0.0129	0.579399	14.06
rs10787738	T	C	0.254549	0.015307	0.002086	2.10E-13	0.014	0.0135	0.3006	20.44
rs10795418	G	A	0.664754	0.0127	0.001902	2.40E-11	0.0098	0.0127	0.4411	19.88
rs10803762	A	G	0.677313	0.011945	0.001915	4.40E-10	0.0133	0.0129	0.3029	17.01
rs10827380	T	C	0.314309	0.01161	0.00193	1.80E-09	0.0042	0.012	0.7289	15.60
rs10835676	G	C	0.240436	0.012351	0.002098	4.00E-09	0.0254	0.0132	0.05481	12.65
rs10887578	C	G	0.497514	0.010668	0.0018	3.10E-09	-0.0133	0.0118	0.2594	17.57
rs10992854	C	T	0.681971	-0.01125	0.001929	5.40E-09	0.0155	0.0124	0.2108	14.77
rs11012732	G	A	0.331675	0.019354	0.001901	2.40E-24	0.0243	0.0126	0.0552	45.95

SNP	effect_allele WC	other_allele WC	eaf. WC	beta. WC	se. WC	pval. WC	beta. cholecystitis	se. cholecystitis	pval. cholecystitis	F-statistics
rs1108548	G	A	0.277245	0.012172	0.002	1.20E-09	0.0057	0.0127	0.652299	14.84
rs11099020	T	C	0.640565	-0.01124	0.001866	1.70E-09	-0.0072	0.0121	0.5528	16.72
rs1111817	G	C	0.365048	-0.01056	0.001881	2.00E-08	-0.018	0.0123	0.1434	14.61
rs111258054	T	C	0.183665	0.015953	0.002341	9.50E-12	0.0218	0.0132	0.096919	13.92
rs11150745	G	A	0.317708	-0.01608	0.001926	6.80E-17	-0.0226	0.0131	0.0851	30.24
rs11162968	C	T	0.31619	0.012228	0.001924	2.10E-10	0.0203	0.0137	0.1379	17.47
rs11165493	A	G	0.343044	0.010758	0.001893	1.30E-08	0.0199	0.0121	0.1006	14.55
rs1117619	G	C	0.250101	-0.01205	0.00206	5.00E-09	0.0111	0.0133	0.4059	12.83
rs11196657	C	T	0.236715	0.012445	0.002106	3.40E-09	0.0106	0.0143	0.4578	12.62
rs11215381	C	T	0.525856	0.011176	0.001794	4.60E-10	-8.00E-04	0.0118	0.9434	19.36
rs11218510	A	G	0.40049	-0.01153	0.001829	2.90E-10	-0.0025	0.0122	0.8398	19.10
rs11223204	G	A	0.434214	0.012215	0.001809	1.50E-11	-0.0107	0.0124	0.3878	22.41
rs11614326	A	G	0.545054	-0.01026	0.001817	1.60E-08	0.0082	0.0119	0.4891	15.82
rs11636611	T	C	0.502804	0.010714	0.001793	2.30E-09	0.0204	0.0117	0.082689	17.86
rs11639596	C	A	0.250798	-0.01187	0.002078	1.10E-08	-0.0099	0.0128	0.4369	12.27
rs11653367	G	A	0.328129	-0.01517	0.001917	2.50E-15	-0.0162	0.0123	0.1878	27.60
rs11666480	G	C	0.536273	0.013289	0.001806	1.90E-13	-0.0143	0.0119	0.2291	26.92
rs11675464	G	A	0.562997	0.011273	0.001799	3.70E-10	0.0203	0.0118	0.08522	19.33
rs11757278	C	T	0.303836	-0.01278	0.001944	4.90E-11	7.00E-04	0.012	0.9517	18.29
rs11767811	A	G	0.181372	-0.01503	0.002319	8.90E-11	-0.0045	0.0145	0.754699	12.48
rs11773362	T	C	0.336125	-0.01048	0.001894	3.20E-08	0.0087	0.0121	0.473301	13.66
rs11778934	G	C	0.536065	-0.01222	0.001799	1.10E-11	0.0211	0.0123	0.08569	22.93
rs11787216	T	C	0.369092	0.011505	0.001892	1.20E-09	0.0043	0.0123	0.723401	17.23
rs1182199	A	C	0.3044	-0.01352	0.001945	3.60E-12	-0.0173	0.0122	0.1571	20.47

SNP	effect_allele WC	other_allele WC	eaf. WC	beta. WC	se. WC	pval. WC	beta. cholecystitis	se. cholecystitis	pval. cholecystitis	F-statistics
rs1183668	G	C	0.37014	-0.01206	0.001862	9.30E-11	-0.0041	0.0125	0.742499	19.56
rs11842871	T	G	0.259942	-0.01279	0.002044	4.00E-10	0.0196	0.0126	0.1185	15.05
rs1188209	G	A	0.551608	0.01024	0.001811	1.60E-08	-0.0158	0.0118	0.1792	15.81
rs11898037	C	T	0.367606	0.010569	0.001853	1.20E-08	-0.0018	0.0118	0.8806	15.12
rs1191600	A	C	0.59357	-0.0108	0.001833	3.80E-09	-0.0136	0.0119	0.2535	16.74
rs12001437	C	T	0.367701	0.010933	0.001855	3.80E-09	0.0202	0.0123	0.1002	16.15
rs12072739	G	A	0.224447	0.016211	0.002143	3.90E-14	0.0042	0.0126	0.739399	19.92
rs12140153	T	G	0.094226	-0.02659	0.003132	2.10E-17	-0.0347	0.0219	0.1137	12.30
rs1218824	A	G	0.661499	0.012491	0.001893	4.20E-11	0.0228	0.0125	0.06727	19.49
rs12287076	C	G	0.707082	0.021064	0.001972	1.30E-26	-0.0021	0.0139	0.8804	47.25
rs12375196	A	C	0.424364	0.013219	0.00182	3.80E-13	-0.0026	0.0118	0.8274	25.76
rs12462975	A	G	0.329671	0.01754	0.001918	5.90E-20	-0.0156	0.0127	0.2173	36.97
rs12463617	C	A	0.828064	0.043192	0.002366	1.80E-74	0.0202	0.0159	0.2037	94.93
rs12478299	C	T	0.252015	-0.01158	0.002062	1.90E-08	-0.0209	0.0135	0.1211	11.89
rs12877270	A	G	0.442217	0.011722	0.001816	1.10E-10	0.0159	0.0118	0.1763	20.57
rs12880641	G	T	0.661581	-0.01384	0.00189	2.40E-13	-0.0138	0.0121	0.2556	24.04
rs12926311	C	G	0.353624	-0.01246	0.001877	3.20E-11	0.0958	0.1888	0.612099	20.13
rs12926311	C	G	0.353624	-0.01246	0.001877	3.20E-11	-0.0109	0.0129	0.399	20.13
rs1296328	C	A	0.559023	-0.01319	0.001809	3.10E-13	-0.0086	0.0118	0.463	26.22
rs12983532	T	C	0.251127	-0.015	0.002092	7.60E-13	-0.0193	0.014	0.1685	19.33
rs13033310	A	G	0.25275	0.012551	0.002068	1.30E-09	-0.0236	0.0137	0.08437	13.92
rs13163306	A	G	0.466047	-0.00989	0.001794	3.50E-08	-0.0092	0.0117	0.4315	15.13
rs13182474	C	G	0.318883	-0.01172	0.001921	1.00E-09	0.0098	0.0141	0.4873	16.18
rs1320903	A	G	0.319725	0.017072	0.001917	5.20E-19	-0.0058	0.0129	0.6543	34.52

SNP	effect_allele WC	other_allele WC	eaf. WC	beta. WC	se. WC	pval. WC	beta. cholecystitis	se. cholecystitis	pval. cholecystitis	F-statistics
rs1321521	A	C	0.345235	0.014143	0.001879	5.10E-14	-0.0185	0.0119	0.1187	25.62
rs13264909	T	A	0.428948	-0.01228	0.00181	1.20E-11	0.0191	0.0117	0.1041	22.55
rs13288841	A	G	0.32166	0.01905	0.001912	2.20E-23	0.0129	0.0122	0.2889	43.31
rs1336486	G	T	0.328585	0.012489	0.001908	6.00E-11	0.0108	0.0119	0.367	18.90
rs13410783	G	A	0.36952	0.014106	0.00185	2.40E-14	0.0125	0.0121	0.301	27.10
rs1346841	A	G	0.40501	-0.0106	0.001826	6.40E-09	-0.0063	0.0127	0.6195	16.25
rs1357079	C	T	0.570076	0.011183	0.001808	6.20E-10	-0.0079	0.012	0.5085	18.76
rs1360201	T	C	0.48157	0.009794	0.001789	4.40E-08	0.0192	0.0118	0.103	14.96
rs1405261	A	T	0.434383	-0.00985	0.001805	4.80E-08	0.0057	0.0118	0.630901	14.64
rs1436348	G	A	0.582801	0.012493	0.001812	5.40E-12	0.0077	0.0122	0.5279	23.12
rs1441098	T	A	0.54446	-0.00991	0.001797	3.50E-08	0	0.0118	0.9994	15.08
rs1441264	A	G	0.593703	0.01501	0.001862	7.60E-16	0.0068	0.0125	0.5851	31.35
rs1454687	G	C	0.515372	-0.01616	0.001787	1.50E-19	-0.0101	0.0118	0.3925	40.86
rs1458156	T	C	0.488413	0.014604	0.001791	3.50E-16	0.023	0.0118	0.0507	33.23
rs1502317	T	C	0.276578	-0.01737	0.002003	4.30E-18	0.0031	0.0133	0.8145	30.09
rs1559900	T	C	0.286025	0.012773	0.001979	1.10E-10	-0.0179	0.0135	0.1853	17.02
rs156902	T	G	0.267387	-0.01382	0.002438	1.40E-08	0.0336	0.0225	0.1348	12.60
rs1570298	T	A	0.743189	0.012045	0.002045	3.80E-09	-0.013	0.0139	0.3487	13.25
rs1582931	A	G	0.473264	-0.01385	0.001806	1.70E-14	-0.0019	0.0117	0.8688	29.35
rs1609010	G	A	0.565725	0.014934	0.001806	1.30E-16	-0.0148	0.0118	0.2081	33.60
rs1609303	A	T	0.631343	0.015266	0.001858	2.10E-16	0.0079	0.0123	0.5183	31.44
rs1625623	T	C	0.371988	0.010741	0.001873	9.80E-09	-0.0075	0.0122	0.5414	15.36
rs165656	C	G	0.517356	0.010304	0.001805	1.10E-08	-0.0049	0.0118	0.6785	16.27
rs1657930	A	G	0.802922	-0.01425	0.002248	2.30E-10	0.0169	0.0163	0.3018	12.71

SNP	effect_allele WC	other_allele WC	eaf. WC	beta. WC	se. WC	pval. WC	beta. cholecystitis	se. cholecystitis	pval. cholecystitis	F-statistics
rs1711171	C	T	0.749579	0.017564	0.002065	1.80E-17	-0.0199	0.0163	0.2225	27.15
rs17296856	C	A	0.280671	-0.01562	0.001996	5.00E-15	6.00E-04	0.0126	0.9621	24.73
rs1731246	T	G	0.757325	-0.01184	0.002082	1.30E-08	0.0161	0.0138	0.2462	11.88
rs17446091	C	T	0.20186	0.014914	0.00223	2.30E-11	0.0077	0.0167	0.6451	14.42
rs1752169	A	C	0.250696	0.014308	0.002067	4.50E-12	-0.0125	0.0121	0.3023	18.00
rs17681738	T	C	0.328589	0.010542	0.001909	3.30E-08	-0.0193	0.0126	0.1252	13.46
rs1834144	A	C	0.373194	-0.01462	0.001856	3.40E-15	-0.0085	0.0118	0.4717	29.01
rs2020942	T	C	0.394892	0.010847	0.001836	3.40E-09	0.0216	0.0119	0.06861	16.69
rs2074881	T	C	0.168487	-0.01476	0.002402	8.00E-10	-0.0306	0.0175	0.079939	10.58
rs2133561	T	A	0.611063	-0.01224	0.001853	3.90E-11	-0.011	0.0123	0.3729	20.76
rs215669	A	G	0.611654	-0.01251	0.001842	1.10E-11	-0.0215	0.0131	0.1002	21.90
rs2161097	T	C	0.437804	0.014277	0.0018	2.20E-15	0.0051	0.0121	0.6736	30.97
rs2172131	C	T	0.578709	-0.01217	0.001813	1.90E-11	-0.0089	0.0117	0.4492	21.99
rs2180454	C	T	0.771831	0.017982	0.002135	3.70E-17	0.0078	0.0125	0.5325	24.98
rs2225909	C	T	0.774104	0.015797	0.002136	1.40E-13	-0.0115	0.0152	0.4507	19.12
rs2253310	G	C	0.626102	0.018205	0.001847	6.50E-23	0.0036	0.0119	0.762901	45.48
rs2302209	T	C	0.288806	0.019833	0.001977	1.10E-23	-0.018	0.0135	0.1822	41.34
rs2306593	T	C	0.488298	-0.01529	0.001795	1.60E-17	-0.0142	0.0118	0.2287	36.27
rs2376885	A	G	0.324117	-0.01062	0.001913	2.80E-08	-0.0049	0.0121	0.684699	13.52
rs2439823	G	A	0.545601	0.015525	0.001801	6.80E-18	0.0218	0.0118	0.065089	36.83
rs245767	G	A	0.730365	0.014598	0.002016	4.50E-13	0	0.0126	0.998	20.64
rs2470549	C	T	0.598143	-0.01195	0.001821	5.20E-11	-0.0159	0.0121	0.1883	20.72
rs2482704	T	G	0.426761	-0.01156	0.001806	1.50E-10	0.0103	0.0121	0.3972	20.06
rs2568958	A	G	0.603683	0.016921	0.001822	1.60E-20	0.0147	0.0123	0.2315	41.28

SNP	effect_allele WC	other_allele WC	eaf. WC	beta. WC	se. WC	pval. WC	beta. cholecystitis	se. cholecystitis	pval. cholecystitis	F-statistics
rs2584205	A	G	0.733265	0.011159	0.002027	3.70E-08	0.0227	0.0136	0.0944	11.86
rs2678204	G	T	0.34017	0.015707	0.001885	7.90E-17	0.0081	0.0129	0.5312	31.18
rs2696309	C	T	0.720419	0.011257	0.001993	1.60E-08	-0.0027	0.0125	0.8308	12.85
rs2725371	G	A	0.696098	-0.01545	0.001953	2.60E-15	-0.0074	0.0134	0.5783	26.48
rs28350	G	A	0.820658	-0.01394	0.002337	2.50E-09	-0.0133	0.0151	0.3787	10.47
rs28366156	C	T	0.130582	-0.01877	0.002651	1.50E-12	0.0152	0.0228	0.5043	11.37
rs28375268	T	G	0.645058	-0.01308	0.001878	3.30E-12	-0.0068	0.0124	0.585	22.22
rs28489620	A	G	0.29035	-0.01241	0.001989	4.40E-10	-0.0065	0.0129	0.6122	16.05
rs28580375	G	C	0.21874	0.012607	0.002168	6.00E-09	0.0078	0.014	0.5772	11.56
rs2861692	C	T	0.275371	-0.01665	0.001998	7.70E-17	0.0076	0.013	0.5582	27.73
rs2903738	T	A	0.221402	-0.01331	0.002157	6.80E-10	0.0017	0.0138	0.9013	13.12
rs308911	G	A	0.714436	-0.01147	0.001981	6.90E-09	0.0113	0.0131	0.387	13.70
rs3113509	T	C	0.731966	-0.01233	0.00202	1.00E-09	-0.0172	0.0131	0.1891	14.62
rs319775	C	T	0.608753	0.010156	0.001834	3.00E-08	0.0122	0.012	0.3111	14.61
rs3212038	G	A	0.328517	0.012655	0.001911	3.50E-11	0.0042	0.0119	0.728201	19.35
rs34045288	T	C	0.334413	0.020375	0.001895	5.80E-27	0.018	0.0121	0.1381	51.47
rs34140906	C	T	0.17033	-0.01794	0.002385	5.40E-14	-0.0227	0.0148	0.1243	15.99
rs34234296	A	G	0.392393	-0.01312	0.001848	1.30E-12	-0.0155	0.0128	0.2254	24.03
rs34483452	A	C	0.136355	0.027048	0.002627	7.30E-25	0.0177	0.018	0.3253	24.97
rs347551	G	C	0.472292	0.012631	0.00182	3.90E-12	0.0179	0.0119	0.1335	24.01
rs34882821	T	G	0.338577	0.010624	0.001896	2.10E-08	0.0032	0.0129	0.8053	14.06
rs34994596	C	T	0.297289	-0.01456	0.001959	1.10E-13	-0.021	0.0133	0.1145	23.09
rs35023999	C	A	0.508297	-0.01134	0.00179	2.40E-10	-0.017	0.0118	0.1483	20.07
rs35243581	T	C	0.317318	0.017364	0.001923	1.70E-19	0.0102	0.0127	0.4228	35.35

SNP	effect_allele WC	other_allele WC	eaf. WC	beta. WC	se. WC	pval. WC	beta. cholecystitis	se. cholecystitis	pval. cholecystitis	F-statistics
rs35681682	C	T	0.407729	-0.01015	0.001773	1.00E-08	-0.0159	0.0118	0.178	15.85
rs35882248	T	C	0.317213	0.015709	0.00192	2.80E-16	0.0026	0.0127	0.8358	29.01
rs36061954	T	C	0.39881	0.011457	0.001827	3.60E-10	-0.0081	0.012	0.5005	18.87
rs36165342	C	T	0.478618	0.010712	0.001789	2.10E-09	-0.0224	0.012	0.06138	17.88
rs3764002	T	C	0.261552	-0.01608	0.002035	2.80E-15	-0.0238	0.0124	0.05499	24.11
rs3768321	T	G	0.196525	0.017673	0.002248	3.80E-15	0.001	0.0159	0.9506	19.51
rs3784692	T	C	0.601752	0.018582	0.001827	2.60E-24	0.0156	0.0123	0.2026	49.61
rs3806114	A	G	0.668366	-0.01081	0.00192	1.80E-08	0.0103	0.0137	0.4542	14.06
rs3807566	T	G	0.438304	-0.01215	0.001806	1.70E-11	0.0115	0.0121	0.3436	22.29
rs3814883	T	C	0.482382	0.023982	0.001795	1.10E-40	0.0031	0.0119	0.7921	89.12
rs3816760	A	G	0.307437	0.013354	0.001939	5.70E-12	0.004	0.0133	0.7663	20.19
rs3845344	T	C	0.391147	0.010737	0.001828	4.30E-09	0.0207	0.0119	0.080251	16.42
rs3866805	A	C	0.355681	0.010421	0.00187	2.50E-08	-0.0031	0.0124	0.8034	14.23
rs3935190	A	G	0.536793	-0.01262	0.001806	2.80E-12	-2.00E-04	0.0118	0.9886	24.28
rs3936510	T	G	0.201255	0.013715	0.002227	7.30E-10	0.0131	0.017	0.4422	12.20
rs3949781	A	T	0.538137	0.011512	0.001806	1.80E-10	0.0132	0.0118	0.2611	20.21
rs40067	A	G	0.170153	-0.01561	0.002387	6.10E-11	-0.0023	0.0144	0.8719	12.09
rs4017425	T	C	0.47017	-0.01013	0.001793	1.60E-08	-0.0193	0.0121	0.1102	15.90
rs4072917	A	G	0.474253	0.011719	0.001799	7.40E-11	0.017	0.0118	0.1503	21.15
rs4075353	A	G	0.344101	-0.01059	0.001892	2.20E-08	-0.0204	0.0123	0.097771	14.14
rs4290163	T	G	0.392697	0.011378	0.001833	5.40E-10	0.0032	0.012	0.7938	18.38
rs429343	G	A	0.576576	-0.01245	0.001809	5.90E-12	-0.0072	0.0118	0.542199	23.12
rs4419475	T	A	0.407376	0.011264	0.00182	6.00E-10	-0.0013	0.0118	0.909	18.50
rs4456769	T	C	0.333423	0.0134	0.0019	1.80E-12	-0.0099	0.0124	0.4227	22.10

SNP	effect_allele WC	other_allele WC	eaf. WC	beta. WC	se. WC	pval. WC	beta. cholecystitis	se. cholecystitis	pval. cholecystitis	F-statistics
rs4469245	T	A	0.662728	-0.01152	0.001891	1.10E-09	-0.0039	0.0128	0.758501	16.59
rs4525978	T	C	0.734555	-0.01138	0.00203	2.10E-08	0.0028	0.014	0.8415	12.24
rs4527444	G	A	0.541317	0.010524	0.001794	4.50E-09	0.0075	0.0117	0.5243	17.08
rs4552632	A	G	0.616568	-0.01017	0.001841	3.30E-08	1.00E-04	0.0124	0.993	14.42
rs4689465	C	T	0.525445	-0.01087	0.00179	1.30E-09	-0.0111	0.0118	0.3443	18.39
rs4706004	G	A	0.217047	-0.01346	0.002168	5.40E-10	0.0133	0.0134	0.3189	13.10
rs4718964	T	G	0.413175	0.012248	0.001822	1.80E-11	-0.007	0.012	0.560899	21.91
rs4742782	G	C	0.315942	0.012491	0.001922	8.00E-11	0.0015	0.012	0.8985	18.26
rs484455	A	G	0.481363	-0.01153	0.001796	1.40E-10	-0.0065	0.0121	0.59	20.58
rs4851283	G	C	0.684805	-0.0174	0.001935	2.50E-19	-0.019	0.0142	0.181	34.88
rs4856720	C	G	0.53938	0.011434	0.001792	1.80E-10	0.0068	0.0118	0.564999	20.24
rs4876611	G	A	0.720239	0.015009	0.001995	5.30E-14	0.001	0.0127	0.9368	22.82
rs4900715	A	G	0.507101	-0.01143	0.001793	1.90E-10	0.0153	0.0119	0.1996	20.30
rs4908672	T	C	0.393063	0.011397	0.00183	4.70E-10	0.015	0.0119	0.205	18.50
rs520478	T	G	0.701359	-0.01251	0.001973	2.30E-10	-0.0127	0.0139	0.36	16.84
rs539515	C	A	0.204929	0.037822	0.002212	1.50E-65	0.0183	0.0154	0.2349	95.31
rs55726687	A	G	0.209735	0.019937	0.002195	1.10E-19	0.0096	0.0143	0.5011	27.34
rs557951	G	T	0.312953	0.012079	0.001932	4.00E-10	0.0113	0.0133	0.3947	16.82
rs559231	T	G	0.393054	0.010752	0.001841	5.20E-09	0.0228	0.0119	0.05478	16.27
rs56803094	G	A	0.226708	-0.01277	0.002146	2.60E-09	-0.0132	0.0132	0.3164	12.42
rs57636386	C	T	0.083822	-0.03091	0.003241	1.50E-21	-0.036	0.0271	0.184	13.97
rs587271	T	C	0.686869	0.011805	0.002008	4.10E-09	0.0106	0.0128	0.4048	14.87
rs58862095	T	C	0.419271	-0.01658	0.001817	7.10E-20	-0.0076	0.0118	0.5178	40.57
rs588660	A	G	0.584119	0.015528	0.001812	1.00E-17	0.01	0.012	0.4042	35.69

SNP	effect_allele WC	other_allele WC	eaf. WC	beta. WC	se. WC	pval. WC	beta. cholecystitis	se. cholecystitis	pval. cholecystitis	F-statistics
rs59068084	T	G	0.410212	0.01014	0.001819	2.50E-08	0.0179	0.0121	0.1365	15.03
rs59104534	T	C	0.298501	0.010719	0.001962	4.70E-08	0.0046	0.0125	0.712	12.50
rs6069037	A	C	0.731389	-0.01113	0.00202	3.60E-08	-0.0056	0.0137	0.681999	11.92
rs61223906	A	G	0.339313	-0.01087	0.001887	8.30E-09	-0.0078	0.0122	0.522701	14.89
rs61813324	T	C	0.135713	0.021976	0.002644	9.50E-17	0.012	0.0163	0.4614	16.20
rs61903695	G	A	0.254942	0.013451	0.002055	5.90E-11	4.00E-04	0.0137	0.979	16.28
rs61992671	G	A	0.491969	-0.013	0.001872	3.80E-12	0.0061	0.012	0.609399	24.12
rs62243489	G	T	0.259239	-0.01556	0.002049	3.00E-14	-0.0076	0.013	0.5553	22.17
rs62261725	G	A	0.326101	-0.01487	0.001909	6.70E-15	-0.0144	0.0122	0.2398	26.68
rs6493498	C	T	0.54539	-0.01308	0.001805	4.20E-13	-0.0068	0.012	0.5701	26.05
rs649458	A	T	0.860102	-0.01818	0.00257	1.50E-12	0.0191	0.0185	0.3003	12.05
rs6551304	G	A	0.831916	0.016827	0.002394	2.10E-12	0.0065	0.0169	0.6999	13.82
rs6567160	C	T	0.232725	0.045164	0.002118	7.19E-101	0.0235	0.0151	0.1205	162.41
rs6575340	A	G	0.636022	0.016158	0.001865	4.60E-18	-0.0027	0.0121	0.8221	34.76
rs6669341	G	A	0.58271	-0.01252	0.00181	4.50E-12	-0.0037	0.0121	0.7597	23.28
rs6682438	C	T	0.673097	0.012477	0.001902	5.40E-11	-0.0015	0.0124	0.9049	18.94
rs6739755	G	A	0.603358	-0.01592	0.001829	3.10E-18	-0.0132	0.012	0.2713	36.29
rs67609008	C	T	0.283612	0.011102	0.001992	2.50E-08	-0.0237	0.0153	0.1209	12.62
rs6791983	A	C	0.750006	0.012315	0.00206	2.30E-09	0.0169	0.014	0.2271	13.40
rs6799080	A	G	0.353391	0.010231	0.001867	4.30E-08	0.008	0.0119	0.4986	13.72
rs6846041	G	C	0.320508	0.012307	0.001915	1.30E-10	0.0142	0.0134	0.2921	18.00
rs6849518	T	C	0.12428	0.021864	0.00271	7.10E-16	-0.0143	0.0157	0.3635	14.17
rs6938973	C	T	0.601488	0.012011	0.001827	4.90E-11	-0.001	0.0126	0.9376	20.72

SNP	effect_allele WC	other_allele WC	eaf. WC	beta. WC	se. WC	pval. WC	beta. cholecystitis	se. cholecystitis	pval. cholecystitis	F-statistics
rs703984	C	G	0.414809	-0.01147	0.00182	2.90E-10	-0.0146	0.0121	0.2256	19.30
rs704061	C	T	0.455057	0.014622	0.001797	4.00E-16	6.00E-04	0.0121	0.9586	32.86
rs7070670	T	C	0.327941	-0.01203	0.001917	3.50E-10	-0.0168	0.0131	0.2013	17.36
rs7115013	T	C	0.442738	-0.01064	0.001805	3.70E-09	0.0025	0.012	0.8348	17.15
rs7132908	A	G	0.384454	0.021513	0.00184	1.40E-31	0.0196	0.0121	0.1055	64.71
rs7169847	T	G	0.635678	-0.01027	0.001868	3.80E-08	0.0124	0.0118	0.2943	14.01
rs7171864	A	G	0.660202	0.012889	0.001899	1.10E-11	0.0123	0.0129	0.342	20.67
rs7206608	G	C	0.321623	0.012	0.001916	3.80E-10	0.0158	0.0123	0.1991	17.11
rs7218014	C	T	0.197315	0.022409	0.002254	2.70E-23	0.0242	0.0138	0.07927	31.31
rs7259070	C	T	0.596078	0.015381	0.001841	6.60E-17	0.0105	0.0119	0.3759	33.60
rs72634826	A	G	0.259865	-0.0149	0.002065	5.40E-13	-0.0029	0.0145	0.8431	20.02
rs72892910	T	G	0.172259	0.03014	0.002371	5.20E-37	0.0074	0.0146	0.6136	46.07
rs72976986	A	G	0.190131	-0.01576	0.002303	7.80E-12	0.0036	0.0161	0.8245	14.42
rs73052033	C	T	0.184916	-0.02101	0.002305	7.90E-20	-0.0018	0.0159	0.9112	25.04
rs73068448	T	C	0.170691	-0.01534	0.002413	2.00E-10	-0.0094	0.0176	0.5924	11.45
rs73142879	T	C	0.192309	-0.02419	0.002281	2.80E-26	6.00E-04	0.0161	0.9727	34.95
rs7324067	C	T	0.761254	0.01213	0.002101	7.70E-09	0.0141	0.0132	0.2851	12.12
rs735033	G	A	0.605139	-0.01027	0.001843	2.50E-08	0.0151	0.0118	0.2027	14.83
rs7372674	A	C	0.357239	0.011895	0.001863	1.70E-10	0.019	0.0128	0.137	18.71
rs7377083	A	C	0.431061	0.01434	0.001819	3.20E-15	0.003	0.0118	0.7969	30.48
rs73985439	C	A	0.307294	0.012405	0.001939	1.60E-10	-0.0131	0.0128	0.3058	17.42
rs7442885	G	C	0.214033	-0.02047	0.002183	6.80E-21	-0.0237	0.0151	0.1166	29.58
rs7498044	A	G	0.217312	-0.01515	0.002193	4.90E-12	-0.0026	0.0136	0.8465	16.24
rs7498665	G	A	0.399688	0.026616	0.001828	4.90E-48	0.0203	0.0119	0.088571	101.79

SNP	effect_allele WC	other_allele WC	eaf. WC	beta. WC	se. WC	pval. WC	beta. cholecystitis	se. cholecystitis	pval. cholecystitis	F-statistics
rs7519259	A	G	0.528392	0.01267	0.001796	1.70E-12	0.0086	0.0118	0.4661	24.80
rs7537581	A	C	0.531758	0.010736	0.001802	2.60E-09	0.0055	0.0119	0.6438	17.67
rs7539903	A	T	0.615583	-0.01072	0.001836	5.30E-09	-0.0104	0.012	0.3874	16.14
rs756717	A	G	0.399071	-0.01072	0.001849	6.80E-09	-0.0179	0.012	0.1379	16.11
rs76286777	C	T	0.217783	0.023476	0.002162	1.80E-27	-0.0186	0.0171	0.2762	40.16
rs7630382	T	C	0.531552	0.013362	0.001793	9.30E-14	-0.0213	0.0118	0.071261	27.65
rs765876	G	A	0.489495	-0.00981	0.001788	4.10E-08	0.007	0.0119	0.5592	15.04
rs7708584	G	A	0.572375	-0.0122	0.001805	1.40E-11	-0.0126	0.012	0.2949	22.35
rs77165542	T	C	0.035495	-0.07007	0.004881	9.90E-47	-0.0545	0.045	0.2256	14.11
rs7752202	T	C	0.145139	0.017701	0.002532	2.70E-12	0.0123	0.0191	0.5176	12.13
rs784257	C	T	0.812569	0.016179	0.002306	2.30E-12	0.0213	0.0157	0.1737	14.99
rs7966251	A	G	0.255065	-0.0116	0.002055	1.70E-08	0.0011	0.0131	0.9349	12.10
rs80243702	A	G	0.160621	0.015166	0.002456	6.70E-10	0.0236	0.02	0.2374	10.28
rs8078135	T	C	0.489933	-0.01015	0.001797	1.60E-08	-0.0015	0.0118	0.8976	15.94
rs8097672	T	A	0.145231	0.01678	0.002554	5.00E-11	0.0203	0.0154	0.1895	10.72
rs815163	C	T	0.563195	-0.01314	0.001798	2.70E-13	-0.0126	0.0118	0.2848	26.27
rs8192675	C	T	0.288645	0.015992	0.001969	4.50E-16	0.0027	0.0133	0.8367	27.10
rs852042	G	A	0.758579	-0.0116	0.002092	3.00E-08	-0.0169	0.0139	0.2228	11.26
rs852983	A	G	0.459562	-0.00983	0.001793	4.20E-08	0.0031	0.0117	0.7898	14.93
rs862227	G	A	0.457951	-0.01098	0.001791	8.80E-10	-0.0063	0.0119	0.5961	18.65
rs862320	T	C	0.409625	-0.01803	0.001822	4.40E-23	-0.0176	0.0119	0.1377	47.36
rs876605	G	A	0.739809	-0.01116	0.002036	4.20E-08	-0.0216	0.0134	0.1087	11.57
rs879620	T	C	0.613194	0.019443	0.001842	4.90E-26	0.0194	0.012	0.1054	52.85
rs883403	C	T	0.154427	-0.01767	0.002475	9.50E-13	-0.0102	0.0141	0.4675	13.31

SNP	effect_allele WC	other_allele WC	eaf. WC	beta. WC	se. WC	pval. WC	beta. cholecystitis	se. cholecystitis	pval. cholecystitis	F-statistics
rs894736	G	A	0.362765	0.015737	0.001868	3.60E-17	-0.0119	0.0118	0.3103	32.81
rs923994	G	A	0.783206	-0.01301	0.002175	2.20E-09	-0.0221	0.0142	0.1199	12.16
rs9289630	C	G	0.389039	0.014476	0.001839	3.50E-15	0.0206	0.0126	0.1036	29.46
rs9294260	A	G	0.476584	0.013178	0.001799	2.40E-13	0.0056	0.0118	0.6363	26.76
rs9316661	C	T	0.801292	-0.01561	0.002247	3.80E-12	-0.0056	0.0148	0.704099	15.36
rs9378676	C	A	0.234005	0.013032	0.002114	7.00E-10	0.0011	0.0128	0.9304	13.63
rs9673839	G	A	0.490971	0.010906	0.001799	1.30E-09	6.00E-04	0.0118	0.9582	18.38
rs9814758	G	T	0.355886	-0.01116	0.001875	2.70E-09	0.0178	0.0126	0.1571	16.22
rs9835772	T	A	0.243635	0.01213	0.002081	5.60E-09	-0.0042	0.014	0.7636	12.52
rs9888533	T	C	0.538081	0.010833	0.001826	3.00E-09	-0.0097	0.012	0.4201	17.50
rs9902846	T	C	0.316036	0.013376	0.00193	4.20E-12	-6.00E-04	0.0128	0.9647	20.77
rs9916444	G	C	0.34174	0.011474	0.00189	1.30E-09	-0.0211	0.0129	0.1017	16.58

Abbreviations: SNP, single nucleotide polymorphism; se, standard error; pval, p-value; WC, waist circumference.

Table S18. Detailed information about single-nucleotide polymorphisms of waist circumference on intestinal infections.

SNP	effect_allele WC	other_allele WC	eaf. WC	beta. WC	se. WC	pval. WC	beta. II	se. II	pval. II	F-statistics
rs1013402	G	A	0.318423	0.0250906	0.00191723	3.90E-39	-0.0312	0.0239	0.1915	74.35
rs10150482	A	G	0.22035	0.0219574	0.00216957	4.50E-24	0.0057	0.0266	0.8289	35.20
rs10184230	T	C	0.647671	-0.0121164	0.00186862	8.90E-11	0.022	0.0244	0.3671	19.19
rs10236214	T	C	0.641963	0.013799	0.00187561	1.90E-13	0.0074	0.0246	0.7634	24.88
rs10248298	A	C	0.366037	0.0132553	0.00185427	8.80E-13	-0.0074	0.0232	0.751299	23.72
rs1025065	G	T	0.639097	-0.010284	0.00186623	3.60E-08	-0.0205	0.0248	0.4088	14.01
rs10257197	G	A	0.841665	-0.0154813	0.00245771	3.00E-10	0.0255	0.0284	0.3695	10.58
rs10269774	A	G	0.326456	0.0119597	0.00190774	3.60E-10	0.0035	0.0246	0.8857	17.28
rs1037702	A	G	0.621776	-0.0101871	0.00184913	3.60E-08	-0.0178	0.0233	0.4434	14.28
rs10406327	G	C	0.478735	0.0103615	0.00179628	8.00E-09	-0.0275	0.0227	0.2257	16.61
rs10423928	A	T	0.194353	-0.0265551	0.00225944	6.80E-32	-0.0102	0.026	0.6941	43.26
rs10471636	A	G	0.508891	-0.010111	0.00182385	3.00E-08	0.0274	0.0228	0.2294	15.36
rs10490869	T	A	0.209589	0.0163389	0.00220375	1.20E-13	8.00E-04	0.0274	0.9774	18.21
rs10499014	G	C	0.268616	-0.0132827	0.00202893	5.90E-11	-0.0618	0.0258	0.0165402	16.84
rs1051613	A	G	0.544526	-0.00995199	0.00179742	3.10E-08	-0.0014	0.0228	0.9495	15.21
rs1056441	C	T	0.675421	0.0127063	0.00191349	3.10E-11	0.0161	0.0259	0.5346	19.33
rs10732335	C	A	0.442949	-0.0147414	0.00180443	3.10E-16	0.0373	0.0227	0.0998206	32.94
rs10757898	A	G	0.520113	-0.00996701	0.00181479	4.00E-08	0.0198	0.0227	0.3838	15.06
rs1078455	C	T	0.309501	0.0111735	0.00194801	9.70E-09	0.0105	0.0249	0.673501	14.06
rs10787738	T	C	0.254549	0.0153066	0.00208556	2.10E-13	-0.0078	0.0261	0.765301	20.44
rs10795418	G	A	0.664754	0.0127004	0.00190172	2.40E-11	0.0446	0.0245	0.0693202	19.88

SNP	effect_allele WC	other_allele WC	eaf. WC	beta. WC	se. WC	pval. WC	beta. II	se. II	pval. II	F-statistics
rs10803762	A	G	0.677313	0.0119451	0.00191493	4.40E-10	0.0372	0.0249	0.1346	17.01
rs10827380	T	C	0.314309	0.0116104	0.0019302	1.80E-09	0.0124	0.0232	0.5936	15.60
rs10835676	G	C	0.240436	0.0123512	0.00209843	4.00E-09	0.0345	0.0255	0.175	12.65
rs10887578	C	G	0.497514	0.0106684	0.00179976	3.10E-09	0.0209	0.0227	0.3584	17.57
rs10938398	A	G	0.433578	0.0222115	0.00180756	1.00E-34	0.009	0.0226	0.6912	74.18
rs10947793	G	A	0.371789	-0.0127212	0.0018605	8.10E-12	-0.0042	0.0233	0.8574	21.84
rs10992854	C	T	0.681971	-0.0112545	0.00192897	5.40E-09	-0.0384	0.0237	0.1055	14.77
rs11012732	G	A	0.331675	0.0193536	0.00190117	2.40E-24	-3.00E-04	0.0243	0.9915	45.95
rs1108548	G	A	0.277245	0.0121715	0.00200003	1.20E-09	0.0087	0.0245	0.721499	14.84
rs11099020	T	C	0.640565	-0.0112449	0.00186604	1.70E-09	-0.0218	0.0232	0.3476	16.72
rs1111817	G	C	0.365048	-0.0105597	0.00188105	2.00E-08	0.008	0.0237	0.7356	14.61
rs111258054	T	C	0.183665	0.0159528	0.00234105	9.50E-12	-0.009	0.0254	0.723601	13.92
rs11150745	G	A	0.317708	-0.0160848	0.00192604	6.80E-17	-0.0225	0.0253	0.3741	30.24
rs11162968	C	T	0.31619	0.0122283	0.00192412	2.10E-10	-0.0175	0.0263	0.5053	17.47
rs11165493	A	G	0.343044	0.0107576	0.00189335	1.30E-08	-0.0167	0.0233	0.4736	14.55
rs1117619	G	C	0.250101	-0.0120513	0.00206049	5.00E-09	0.0252	0.0257	0.3266	12.83
rs11196657	C	T	0.236715	0.0124451	0.00210599	3.40E-09	0.0256	0.0275	0.3529	12.62
rs11215381	C	T	0.525856	0.0111759	0.00179363	4.60E-10	-1.00E-04	0.0227	0.9961	19.36
rs11218510	A	G	0.40049	-0.0115323	0.00182872	2.90E-10	-0.0375	0.0236	0.1118	19.10
rs11223204	G	A	0.434214	0.0122154	0.00180899	1.50E-11	6.00E-04	0.024	0.9801	22.41
rs11614326	A	G	0.545054	-0.0102606	0.0018167	1.60E-08	-0.0049	0.0229	0.8304	15.82
rs11636611	T	C	0.502804	0.0107144	0.00179296	2.30E-09	0.006	0.0226	0.7921	17.86
rs11639596	C	A	0.250798	-0.011872	0.00207809	1.10E-08	-0.0186	0.0247	0.4516	12.27
rs11653367	G	A	0.328129	-0.0151666	0.00191685	2.50E-15	0.0414	0.0237	0.0802897	27.60

SNP	effect_allele WC	other_allele WC	eaf. WC	beta. WC	se. WC	pval. WC	beta. II	se. II	pval. II	F-statistics
rs11666480	G	C	0.536273	0.0132885	0.00180641	1.90E-13	0.0242	0.0229	0.2898	26.92
rs11675464	G	A	0.562997	0.0112729	0.00179869	3.70E-10	-0.0102	0.0228	0.6531	19.33
rs11704728	T	C	0.196347	0.0133307	0.00226433	3.90E-09	0.0376	0.0275	0.1718	10.94
rs11757278	C	T	0.303836	-0.012784	0.00194427	4.90E-11	0.0094	0.0232	0.684499	18.29
rs11767811	A	G	0.181372	-0.0150345	0.00231875	8.90E-11	-0.0231	0.028	0.4093	12.48
rs11773362	T	C	0.336125	-0.0104772	0.0018937	3.20E-08	-0.0166	0.0234	0.4778	13.66
rs11778934	G	C	0.536065	-0.0122168	0.00179945	1.10E-11	-0.0081	0.0237	0.733599	22.93
rs11787216	T	C	0.369092	0.011505	0.0018915	1.20E-09	-0.0117	0.0236	0.6182	17.23
rs1182199	A	C	0.3044	-0.0135196	0.00194469	3.60E-12	0.0023	0.0235	0.9233	20.47
rs11824092	C	T	0.635754	0.012491	0.00187069	2.40E-11	-0.0245	0.0227	0.2797	20.65
rs1183668	G	C	0.37014	-0.0120589	0.00186172	9.30E-11	0.0221	0.0241	0.3604	19.56
rs11842871	T	G	0.259942	-0.0127878	0.00204439	4.00E-10	0.0629	0.0242	0.0092841	15.05
rs1188209	G	A	0.551608	0.0102398	0.00181136	1.60E-08	-0.0428	0.0227	0.0595004	15.81
rs11898037	C	T	0.367606	0.0105694	0.00185324	1.20E-08	0.0144	0.0229	0.529	15.12
rs1191600	A	C	0.59357	-0.0108005	0.00183345	3.80E-09	0.0438	0.023	0.0567597	16.74
rs12001437	C	T	0.367701	0.0109331	0.00185543	3.80E-09	0.0123	0.0237	0.6032	16.15
rs12072739	G	A	0.224447	0.0162106	0.00214325	3.90E-14	0.0203	0.0242	0.4017	19.92
rs12140153	T	G	0.094226	-0.0265936	0.00313243	2.10E-17	-0.0074	0.0427	0.8618	12.30
rs1218824	A	G	0.661499	0.012491	0.00189332	4.20E-11	-0.015	0.024	0.533	19.49
rs12287076	C	G	0.707082	0.0210638	0.00197238	1.30E-26	-0.0323	0.027	0.2321	47.25
rs12375196	A	C	0.424364	0.0132187	0.00182044	3.80E-13	-0.0047	0.0228	0.8383	25.76
rs12462975	A	G	0.329671	0.01754	0.00191777	5.90E-20	-0.027	0.0244	0.2672	36.97
rs12463617	C	A	0.828064	0.0431919	0.00236577	1.80E-74	-0.0241	0.031	0.4353	94.93
rs12478299	C	T	0.252015	-0.0115796	0.00206169	1.90E-08	-0.0472	0.0258	0.0673798	11.89

SNP	effect_allele WC	other_allele WC	eaf. WC	beta. WC	se. WC	pval. WC	beta. II	se. II	pval. II	F-statistics
rs12877270	A	G	0.442217	0.0117224	0.00181553	1.10E-10	-0.002	0.0227	0.9304	20.57
rs12880641	G	T	0.661581	-0.0138449	0.00188965	2.40E-13	-0.0249	0.0233	0.2853	24.04
rs12926311	C	G	0.353624	-0.0124576	0.00187714	3.20E-11	-0.0225	0.0249	0.3669	20.13
rs12926311	C	G	0.353624	-0.0124576	0.00187714	3.20E-11	0.0453	0.378	0.9047	20.13
rs1296328	C	A	0.559023	-0.0131912	0.00180908	3.10E-13	0.0123	0.0227	0.5881	26.22
rs12983532	T	C	0.251127	-0.0149964	0.00209191	7.60E-13	0.0253	0.0271	0.3498	19.33
rs13033310	A	G	0.25275	0.0125512	0.00206781	1.30E-09	-0.0079	0.0265	0.7666	13.92
rs13047416	G	C	0.37702	-0.0139009	0.00185438	6.60E-14	0.0075	0.0227	0.7411	26.40
rs13163306	A	G	0.466047	-0.00988959	0.00179351	3.50E-08	0.0194	0.0227	0.3925	15.13
rs13182474	C	G	0.318883	-0.0117225	0.00192056	1.00E-09	-0.0369	0.0273	0.1775	16.18
rs1320903	A	G	0.319725	0.017072	0.00191652	5.20E-19	-0.0393	0.0249	0.1144	34.52
rs1321521	A	C	0.345235	0.0141428	0.00187867	5.10E-14	-0.0141	0.0228	0.5378	25.62
rs13264909	T	A	0.428948	-0.0122831	0.00181048	1.20E-11	0.0055	0.0227	0.8076	22.55
rs1327259	G	A	0.387739	-0.0115864	0.0018403	3.10E-10	-0.0164	0.0228	0.4721	18.82
rs13288841	A	G	0.32166	0.0190501	0.00191229	2.20E-23	-0.0355	0.0233	0.1276	43.31
rs13322435	G	A	0.404446	-0.0169561	0.00182964	1.90E-20	0.0107	0.024	0.6566	41.38
rs13333747	C	T	0.182682	-0.022678	0.00232636	1.90E-22	0.0235	0.0295	0.4256	28.38
rs1336486	G	T	0.328585	0.012489	0.00190819	6.00E-11	0.0136	0.023	0.5539	18.90
rs13410783	G	A	0.36952	0.0141058	0.0018498	2.40E-14	0.0371	0.0233	0.1118	27.10
rs13420048	A	C	0.365011	-0.0133504	0.00185957	7.00E-13	0.0458	0.0229	0.0451398	23.89
rs13427822	G	A	0.271197	-0.0141495	0.00203048	3.20E-12	-0.0066	0.0248	0.7906	19.20
rs1346841	A	G	0.40501	-0.0106038	0.00182614	6.40E-09	-0.0185	0.0244	0.4483	16.25
rs1357079	C	T	0.570076	0.011183	0.00180784	6.20E-10	0.0168	0.023	0.4646	18.76

SNP	effect_allele WC	other_allele WC	eaf. WC	beta. WC	se. WC	pval. WC	beta. II	se. II	pval. II	F-statistics
rs1360201	T	C	0.48157	0.00979421	0.00178924	4.40E-08	0.0532	0.0227	0.0190498	14.96
rs1405261	A	T	0.434383	-0.00985187	0.0018049	4.80E-08	0.0065	0.0227	0.774901	14.64
rs1411432	C	A	0.186234	0.0150036	0.00230632	7.70E-11	-0.0299	0.0298	0.3146	12.83
rs1436348	G	A	0.582801	0.0124929	0.00181185	5.40E-12	0.0235	0.0235	0.3171	23.12
rs1441098	T	A	0.54446	-0.00990642	0.00179682	3.50E-08	-0.0036	0.0228	0.874	15.08
rs1441264	A	G	0.593703	0.0150095	0.00186203	7.60E-16	0	0.0242	0.9992	31.35
rs1454687	G	C	0.515372	-0.0161615	0.00178705	1.50E-19	0.0308	0.0228	0.1756	40.86
rs1458156	T	C	0.488413	0.0146036	0.00179105	3.50E-16	0.0117	0.0227	0.606001	33.23
rs1502317	T	C	0.276578	-0.0173687	0.0020032	4.30E-18	-0.0098	0.0257	0.7038	30.09
rs1559900	T	C	0.286025	0.0127734	0.00197882	1.10E-10	0.0346	0.0259	0.182	17.02
rs156902	T	G	0.267387	-0.0138228	0.00243775	1.40E-08	-0.0586	0.043	0.1731	12.60
rs1570298	T	A	0.743189	0.0120451	0.00204452	3.80E-09	0.0183	0.0268	0.494899	13.25
rs1582931	A	G	0.473264	-0.0138548	0.00180588	1.70E-14	0.0219	0.0227	0.3342	29.35
rs1609010	G	A	0.565725	0.0149337	0.00180594	1.30E-16	0.0355	0.0226	0.1164	33.60
rs1609303	A	T	0.631343	0.0152662	0.00185779	2.10E-16	-0.0245	0.0236	0.2993	31.44
rs1625623	T	C	0.371988	0.0107407	0.00187307	9.80E-09	0.0075	0.0236	0.7514	15.36
rs165656	C	G	0.517356	0.0103041	0.00180539	1.10E-08	-0.0423	0.0227	0.0624698	16.27
rs1657930	A	G	0.802922	-0.0142481	0.00224816	2.30E-10	8.00E-04	0.0314	0.9803	12.71
rs1711171	C	T	0.749579	0.0175635	0.00206542	1.80E-17	-0.0089	0.0317	0.7783	27.15
rs17296856	C	A	0.280671	-0.0156228	0.00199629	5.00E-15	-0.0055	0.0243	0.8197	24.73
rs1731246	T	G	0.757325	-0.0118366	0.0020818	1.30E-08	-0.0252	0.0266	0.344	11.88
rs17446091	C	T	0.20186	0.0149141	0.00222979	2.30E-11	0.0215	0.0322	0.5032	14.42

SNP	effect_allele WC	other_allele WC	eaf. WC	beta. WC	se. WC	pval. WC	beta. II	se. II	pval. II	F-statistics
rs1752169	A	C	0.250696	0.0143082	0.00206705	4.50E-12	-0.0208	0.0233	0.372	18.00
rs17681738	T	C	0.328589	0.0105421	0.00190864	3.30E-08	-0.009	0.0243	0.712	13.46
rs1834144	A	C	0.373194	-0.0146152	0.00185609	3.40E-15	-0.0254	0.0228	0.264	29.01
rs1861410	T	C	0.555467	-0.016009	0.00180199	6.40E-19	-0.0019	0.0231	0.9355	38.98
rs1902066	C	T	0.562316	0.0109526	0.00181038	1.40E-09	0.006	0.0227	0.790801	18.02
rs2020942	T	C	0.394892	0.0108469	0.00183573	3.40E-09	0.0527	0.0229	0.0213201	16.69
rs2074881	T	C	0.168487	-0.0147612	0.00240193	8.00E-10	-0.0413	0.0335	0.2177	10.58
rs2133561	T	A	0.611063	-0.0122441	0.00185284	3.90E-11	0.0223	0.0236	0.346	20.76
rs215669	A	G	0.611654	-0.0125095	0.00184246	1.10E-11	-0.0321	0.0253	0.2046	21.90
rs2161097	T	C	0.437804	0.0142773	0.00180004	2.20E-15	0.02	0.0233	0.391	30.97
rs2172131	C	T	0.578709	-0.0121742	0.00181299	1.90E-11	-0.0077	0.0227	0.7335	21.99
rs2180454	C	T	0.771831	0.0179823	0.00213539	3.70E-17	0.0366	0.0241	0.1289	24.98
rs2183947	A	G	0.225006	-0.0220783	0.00213628	4.90E-25	-0.0123	0.0268	0.646501	37.25
rs2225909	C	T	0.774104	0.015797	0.00213642	1.40E-13	0.0258	0.0294	0.3797	19.12
rs2253310	G	C	0.626102	0.018205	0.00184715	6.50E-23	0.0183	0.023	0.4253	45.48
rs2302209	T	C	0.288806	0.0198329	0.00197713	1.10E-23	-0.0448	0.0259	0.0835199	41.34
rs2306593	T	C	0.488298	-0.0152919	0.00179509	1.60E-17	-0.0178	0.0227	0.432	36.27
rs2307111	C	T	0.394972	-0.0239722	0.0018299	3.30E-39	-0.024	0.023	0.2951	82.04
rs2376885	A	G	0.324117	-0.0106223	0.00191252	2.80E-08	-0.0071	0.0232	0.7597	13.52
rs2439823	G	A	0.545601	0.0155254	0.00180145	6.80E-18	-0.0352	0.0227	0.1212	36.83
rs245767	G	A	0.730365	0.0145976	0.00201635	4.50E-13	-0.0381	0.0241	0.1145	20.64
rs2470549	C	T	0.598143	-0.0119541	0.00182105	5.20E-11	-0.0288	0.0233	0.2164	20.72
rs2470946	T	G	0.401445	0.011687	0.00182473	1.50E-10	0.05	0.0232	0.0313899	19.71
rs2482704	T	G	0.426761	-0.0115642	0.00180618	1.50E-10	0.0044	0.0235	0.8497	20.06

SNP	effect_allele WC	other_allele WC	eaf. WC	beta. WC	se. WC	pval. WC	beta. II	se. II	pval. II	F-statistics
rs2568958	A	G	0.603683	0.0169206	0.00182184	1.60E-20	0.0475	0.0237	0.04489	41.28
rs2584205	A	G	0.733265	0.0111592	0.00202662	3.70E-08	-0.0355	0.0262	0.1751	11.86
rs2618039	T	A	0.381482	0.0120777	0.00183977	5.20E-11	-0.0125	0.0227	0.5824	20.34
rs2678204	G	T	0.34017	0.0157074	0.00188488	7.90E-17	0.0067	0.0248	0.7865	31.18
rs2696309	C	T	0.720419	0.0112571	0.00199327	1.60E-08	-0.0336	0.024	0.1623	12.85
rs2725371	G	A	0.696098	-0.0154452	0.00195255	2.60E-15	-0.0296	0.0259	0.2517	26.48
rs2744938	G	A	0.147512	0.0319686	0.00251588	5.40E-37	0.0328	0.0282	0.2443	40.61
rs28350	G	A	0.820658	-0.0139376	0.0023374	2.50E-09	-0.026	0.0291	0.372	10.47
rs28366156	C	T	0.130582	-0.0187659	0.00265145	1.50E-12	0.0046	0.0443	0.9176	11.37
rs28375268	T	G	0.645058	-0.0130789	0.00187766	3.30E-12	-0.0079	0.0238	0.7403	22.22
rs28489620	A	G	0.29035	-0.0124131	0.00198899	4.40E-10	-0.0123	0.0247	0.6193	16.05
rs28580375	G	C	0.21874	0.0126069	0.00216777	6.00E-09	0.0116	0.0268	0.664401	11.56
rs2861692	C	T	0.275371	-0.0166517	0.00199772	7.70E-17	0.0409	0.0249	0.101	27.73
rs2903738	T	A	0.221402	-0.0133061	0.00215663	6.80E-10	0.0174	0.0266	0.5114	13.12
rs308911	G	A	0.714436	-0.0114749	0.00198055	6.90E-09	-0.006	0.0252	0.8126	13.70
rs3113509	T	C	0.731966	-0.0123292	0.00201975	1.00E-09	-0.019	0.0252	0.4514	14.62
rs319775	C	T	0.608753	0.0101556	0.0018335	3.00E-08	0.0387	0.0231	0.0939096	14.61
rs3212038	G	A	0.328517	0.0126546	0.00191072	3.50E-11	-0.0144	0.023	0.5306	19.35
rs34045288	T	C	0.334413	0.0203746	0.00189485	5.80E-27	0.0141	0.0233	0.5466	51.47
rs34140906	C	T	0.17033	-0.0179391	0.00238486	5.40E-14	-0.0599	0.0285	0.0355304	15.99
rs34234296	A	G	0.392393	-0.0131214	0.00184833	1.30E-12	0.0229	0.0247	0.3531	24.03
rs34483452	A	C	0.136355	0.0270483	0.0026269	7.30E-25	-0.0266	0.0348	0.4435	24.97
rs34517439	A	C	0.121789	0.0305491	0.00276165	1.90E-28	2.00E-04	0.0342	0.9954	26.18
rs347551	G	C	0.472292	0.0126307	0.00181978	3.90E-12	0.0058	0.0229	0.7989	24.01

SNP	effect_allele WC	other_allele WC	eaf. WC	beta. WC	se. WC	pval. WC	beta. II	se. II	pval. II	F-statistics
rs34882821	T	G	0.338577	0.0106237	0.00189644	2.10E-08	-0.0171	0.0249	0.4917	14.06
rs34994596	C	T	0.297289	-0.014559	0.00195866	1.10E-13	-0.0126	0.0257	0.6244	23.09
rs35023999	C	A	0.508297	-0.0113409	0.00178977	2.40E-10	0.012	0.0227	0.5961	20.07
rs35243581	T	C	0.317318	0.0173643	0.00192251	1.70E-19	-0.0111	0.0244	0.6487	35.35
rs35681682	C	T	0.407729	-0.0101534	0.00177268	1.00E-08	0.0188	0.0228	0.409	15.85
rs35882248	T	C	0.317213	0.0157088	0.00191956	2.80E-16	-0.0257	0.0243	0.2905	29.01
rs36061954	T	C	0.39881	0.0114574	0.00182669	3.60E-10	0.0298	0.0233	0.2003	18.87
rs36140	C	A	0.635311	0.0111762	0.001867	2.10E-09	0.0368	0.0237	0.1212	16.61
rs36165342	C	T	0.478618	0.0107118	0.00178943	2.10E-09	0.0247	0.0231	0.2858	17.88
rs3764002	T	C	0.261552	-0.0160772	0.00203505	2.80E-15	0.0042	0.0239	0.861	24.11
rs3768321	T	G	0.196525	0.0176726	0.00224849	3.80E-15	-0.0082	0.0307	0.788199	19.51
rs3784692	T	C	0.601752	0.0185824	0.00182662	2.60E-24	0.0338	0.0236	0.1516	49.61
rs3806114	A	G	0.668366	-0.0108144	0.00192005	1.80E-08	0.0188	0.0264	0.4773	14.06
rs3807566	T	G	0.438304	-0.012149	0.00180583	1.70E-11	0.0024	0.0233	0.9184	22.29
rs3814883	T	C	0.482382	0.0239816	0.00179535	1.10E-40	0.0021	0.0229	0.9259	89.12
rs3816760	A	G	0.307437	0.0133538	0.00193927	5.70E-12	0.0016	0.0258	0.9496	20.19
rs3826408	T	C	0.456788	0.0112249	0.00179579	4.10E-10	0.0016	0.0226	0.943	19.39
rs3845344	T	C	0.391147	0.0107371	0.00182845	4.30E-09	0.0246	0.0229	0.2821	16.42
rs3866805	A	C	0.355681	0.010421	0.00187021	2.50E-08	-0.0103	0.0239	0.6656	14.23
rs3935190	A	G	0.536793	-0.0126195	0.00180625	2.80E-12	0.0042	0.0227	0.8519	24.28
rs3936510	T	G	0.201255	0.0137147	0.0022268	7.30E-10	0.0706	0.0328	0.03125	12.20
rs3949781	A	T	0.538137	0.0115117	0.00180564	1.80E-10	-0.0234	0.0227	0.3021	20.21
rs40067	A	G	0.170153	-0.0156125	0.00238663	6.10E-11	0.0132	0.0278	0.6363	12.09
rs4017425	T	C	0.47017	-0.0101269	0.00179274	1.60E-08	0.0025	0.0233	0.9159	15.90

SNP	effect_allele WC	other_allele WC	eaf. WC	beta. WC	se. WC	pval. WC	beta. II	se. II	pval. II	F-statistics
rs4072917	A	G	0.474253	0.0117189	0.0017994	7.40E-11	0.0194	0.0229	0.397	21.15
rs4075353	A	G	0.344101	-0.0105885	0.00189213	2.20E-08	0.0182	0.0236	0.4418	14.14
rs4290163	T	G	0.392697	0.0113777	0.00183305	5.40E-10	-0.028	0.0233	0.229	18.38
rs429343	G	A	0.576576	-0.0124509	0.00180931	5.90E-12	-0.0184	0.0227	0.4174	23.12
rs429358	C	T	0.154146	-0.0271422	0.00248126	7.50E-28	-0.0835	0.0292	0.004271	31.21
rs4419475	T	A	0.407376	0.0112635	0.00181962	6.00E-10	0.0493	0.0226	0.0293002	18.50
rs4456769	T	C	0.333423	0.0134001	0.0019003	1.80E-12	0.0183	0.0238	0.4421	22.10
rs4469245	T	A	0.662728	-0.0115211	0.00189126	1.10E-09	0.0428	0.0248	0.0844092	16.59
rs4525978	T	C	0.734555	-0.0113759	0.00203018	2.10E-08	0.045	0.027	0.0963607	12.24
rs4527444	G	A	0.541317	0.0105243	0.00179433	4.50E-09	-0.0094	0.0226	0.678	17.08
rs4552632	A	G	0.616568	-0.0101695	0.00184128	3.30E-08	0.0496	0.0239	0.0383301	14.42
rs4689465	C	T	0.525445	-0.0108689	0.00178976	1.30E-09	-0.0086	0.0226	0.703301	18.39
rs4706004	G	A	0.217047	-0.0134593	0.00216831	5.40E-10	-0.0485	0.0256	0.0581393	13.10
rs4718964	T	G	0.413175	0.0122483	0.00182202	1.80E-11	0.0247	0.0232	0.2851	21.91
rs4742782	G	C	0.315942	0.0124912	0.00192172	8.00E-11	-0.0237	0.0233	0.3097	18.26
rs484455	A	G	0.481363	-0.0115336	0.00179638	1.40E-10	-0.0248	0.0233	0.2879	20.58
rs4851283	G	C	0.684805	-0.0173961	0.00193525	2.50E-19	0.0078	0.0276	0.7758	34.88
rs4856720	C	G	0.53938	0.0114342	0.0017918	1.80E-10	-0.0253	0.0227	0.2643	20.24
rs4876611	G	A	0.720239	0.0150085	0.0019947	5.30E-14	-0.0463	0.0245	0.0584804	22.82
rs4900715	A	G	0.507101	-0.0114279	0.00179331	1.90E-10	-0.0636	0.023	0.00573997	20.30
rs4908672	T	C	0.393063	0.0113966	0.00183009	4.70E-10	-0.0089	0.0228	0.6959	18.50
rs520478	T	G	0.701359	-0.0125051	0.00197262	2.30E-10	0.0759	0.0268	0.00467498	16.84
rs539515	C	A	0.204929	0.0378223	0.00221181	1.50E-65	-0.0215	0.0295	0.4662	95.31
rs55726687	A	G	0.209735	0.0199369	0.00219529	1.10E-19	0.0073	0.0277	0.7914	27.34

SNP	effect_allele WC	other_allele WC	eaf. WC	beta. WC	se. WC	pval. WC	beta. II	se. II	pval. II	F-statistics
rs557951	G	T	0.312953	0.0120789	0.00193163	4.00E-10	0.0254	0.0257	0.323	16.82
rs559231	T	G	0.393054	0.0107518	0.00184107	5.20E-09	-0.0061	0.023	0.7903	16.27
rs56094641	G	A	0.404591	0.057552	0.00182234	1.00E-200	-0.0197	0.0229	0.3903	481.03
rs56803094	G	A	0.226708	-0.0127745	0.00214599	2.60E-09	0.009	0.0254	0.724299	12.42
rs57636386	C	T	0.083822	-0.0309067	0.00324088	1.50E-21	0.0235	0.0521	0.651	13.97
rs587271	T	C	0.686869	0.0118054	0.0020079	4.10E-09	0.0301	0.0245	0.2199	14.87
rs58862095	T	C	0.419271	-0.0165812	0.00181678	7.10E-20	0.0136	0.0227	0.5496	40.57
rs588660	A	G	0.584119	0.0155276	0.00181174	1.00E-17	0.0177	0.0231	0.4442	35.69
rs59068084	T	G	0.410212	0.0101403	0.00181938	2.50E-08	0.0427	0.0232	0.0657794	15.03
rs59104534	T	C	0.298501	0.0107189	0.00196197	4.70E-08	-0.0171	0.0242	0.4786	12.50
rs6001877	A	G	0.339906	-0.010579	0.00189657	2.40E-08	-0.0165	0.0234	0.482	13.96
rs6069037	A	C	0.731389	-0.011125	0.00202001	3.60E-08	0.0478	0.0265	0.0705895	11.92
rs61223906	A	G	0.339313	-0.0108726	0.00188655	8.30E-09	1.00E-04	0.0235	0.9965	14.89
rs61813324	T	C	0.135713	0.0219762	0.00264443	9.50E-17	-0.0232	0.0313	0.4582	16.20
rs61903695	G	A	0.254942	0.0134514	0.00205497	5.90E-11	0.0108	0.0263	0.681201	16.28
rs61969511	A	G	0.278938	0.011857	0.0020127	3.80E-09	-0.0404	0.0234	0.0847598	13.96
rs61992671	G	A	0.491969	-0.0130005	0.00187164	3.80E-12	0.012	0.023	0.6002	24.12
rs62243489	G	T	0.259239	-0.0155648	0.00204876	3.00E-14	-0.0182	0.0249	0.4655	22.17
rs62261725	G	A	0.326101	-0.0148698	0.00190872	6.70E-15	-0.0113	0.0236	0.633299	26.68
rs6493498	C	T	0.54539	-0.013082	0.001805	4.20E-13	-0.0207	0.023	0.3697	26.05
rs649458	A	T	0.860102	-0.018179	0.00256951	1.50E-12	0.006	0.0357	0.8659	12.05
rs6536575	C	T	0.51907	0.0108432	0.00178995	1.40E-09	-0.0143	0.023	0.533	18.32
rs6551304	G	A	0.831916	0.016827	0.00239416	2.10E-12	0.0215	0.0327	0.5105	13.82

SNP	effect_allele WC	other_allele WC	eaf. WC	beta. WC	se. WC	pval. WC	beta. II	se. II	pval. II	F-statistics
rs6567160	C	T	0.232725	0.0451644	0.00211824	7.19E-101	1.00E-04	0.0291	0.9975	162.41
rs6575340	A	G	0.636022	0.0161584	0.00186502	4.60E-18	0.0136	0.0235	0.5612	34.76
rs6669341	G	A	0.58271	-0.0125214	0.0018097	4.50E-12	-0.025	0.0233	0.2838	23.28
rs6682438	C	T	0.673097	0.0124771	0.00190203	5.40E-11	-0.0104	0.024	0.663599	18.94
rs6693294	G	A	0.688535	-0.0170126	0.00192843	1.10E-18	0.027	0.0233	0.2466	33.38
rs6739755	G	A	0.603358	-0.0159241	0.00182885	3.10E-18	0.0067	0.023	0.770699	36.29
rs67609008	C	T	0.283612	0.011102	0.00199233	2.50E-08	-0.0253	0.0295	0.3913	12.62
rs6791983	A	C	0.750006	0.0123148	0.00205988	2.30E-09	-0.0074	0.0267	0.7828	13.40
rs6799080	A	G	0.353391	0.0102307	0.00186721	4.30E-08	-0.0014	0.0228	0.9503	13.72
rs6846041	G	C	0.320508	0.0123073	0.00191467	1.30E-10	-0.0239	0.0259	0.3577	18.00
rs6849518	T	C	0.12428	0.0218636	0.00270973	7.10E-16	-0.0166	0.0303	0.5847	14.17
rs6938973	C	T	0.601488	0.0120106	0.001827	4.90E-11	-0.0101	0.0243	0.6783	20.72
rs7034554	G	A	0.373813	-0.0112644	0.00184813	1.10E-09	-0.0259	0.025	0.3019	17.39
rs703984	C	G	0.414809	-0.011472	0.00181967	2.90E-10	-0.0879	0.0233	0.000156498	19.30
rs704061	C	T	0.455057	0.0146223	0.00179659	4.00E-16	-0.0211	0.0234	0.3664	32.86
rs7070670	T	C	0.327941	-0.0120318	0.00191737	3.50E-10	0.0057	0.0253	0.8209	17.36
rs7115013	T	C	0.442738	-0.0106394	0.00180461	3.70E-09	0.0593	0.0231	0.0101501	17.15
rs7132908	A	G	0.384454	0.0215129	0.00183998	1.40E-31	0.0147	0.0234	0.5284	64.71
rs7169847	T	G	0.635678	-0.0102703	0.00186771	3.80E-08	0.0338	0.0227	0.136	14.01
rs7171864	A	G	0.660202	0.0128894	0.00189898	1.10E-11	-0.0225	0.0249	0.3663	20.67
rs7206608	G	C	0.321623	0.0120004	0.00191641	3.80E-10	0.019	0.0236	0.4206	17.11
rs7218014	C	T	0.197315	0.0224089	0.00225393	2.70E-23	0.055	0.0266	0.0384902	31.31
rs7259070	C	T	0.596078	0.0153811	0.00184133	6.60E-17	0.0343	0.0228	0.1327	33.60

SNP	effect_allele WC	other_allele WC	eaf. WC	beta. WC	se. WC	pval. WC	beta. II	se. II	pval. II	F-statistics
rs72634826	A	G	0.259865	-0.0148957	0.00206456	5.40E-13	0.0072	0.0278	0.7958	20.02
rs72892910	T	G	0.172259	0.0301404	0.00237148	5.20E-37	0.0423	0.028	0.1311	46.07
rs72976986	A	G	0.190131	-0.0157587	0.00230334	7.80E-12	-0.0159	0.0312	0.6112	14.42
rs73052033	C	T	0.184916	-0.0210096	0.00230513	7.90E-20	0.038	0.0308	0.2168	25.04
rs73068448	T	C	0.170691	-0.0153436	0.00241309	2.00E-10	0.0353	0.0341	0.2997	11.45
rs73142879	T	C	0.192309	-0.0241942	0.00228107	2.80E-26	0.0158	0.031	0.609	34.95
rs7324067	C	T	0.761254	0.0121297	0.00210064	7.70E-09	-0.0274	0.0254	0.281	12.12
rs735033	G	A	0.605139	-0.0102658	0.00184312	2.50E-08	0.0276	0.0228	0.2273	14.83
rs7372674	A	C	0.357239	0.0118947	0.00186339	1.70E-10	0.0133	0.0246	0.5903	18.71
rs7377083	A	C	0.431061	0.0143396	0.00181913	3.20E-15	-0.018	0.0228	0.4306	30.48
rs73985439	C	A	0.307294	0.0124046	0.00193905	1.60E-10	0.0096	0.0246	0.697999	17.42
rs7442885	G	C	0.214033	-0.0204724	0.00218339	6.80E-21	-0.031	0.0291	0.287	29.58
rs7498044	A	G	0.217312	-0.0151498	0.00219298	4.90E-12	-0.0448	0.0262	0.08684	16.24
rs7498665	G	A	0.399688	0.026616	0.00182769	4.90E-48	0.0029	0.023	0.8999	101.79
rs7519259	A	G	0.528392	0.0126703	0.00179634	1.70E-12	0.0087	0.0228	0.7021	24.80
rs7537581	A	C	0.531758	0.0107362	0.00180248	2.60E-09	0.0481	0.0229	0.0354699	17.67
rs7539903	A	T	0.615583	-0.0107184	0.00183572	5.30E-09	-0.0141	0.0231	0.5418	16.14
rs756717	A	G	0.399071	-0.0107152	0.00184896	6.80E-09	-0.0139	0.0232	0.5485	16.11
rs76286777	C	T	0.217783	0.0234759	0.0021623	1.80E-27	0.0394	0.0332	0.2351	40.16
rs7630382	T	C	0.531552	0.013362	0.00179336	9.30E-14	-0.0027	0.0228	0.9046	27.65
rs765876	G	A	0.489495	-9.81E-03	0.00178785	4.10E-08	4.00E-04	0.0231	0.9862	15.04
rs7707394	A	G	0.357264	-0.0167827	0.00186285	2.10E-19	-0.0095	0.0234	0.6851	37.28
rs7708584	G	A	0.572375	-0.0121976	0.00180534	1.40E-11	0.0088	0.0231	0.702901	22.35
rs77165542	T	C	0.035495	-0.0700663	0.00488088	9.90E-47	0.0489	0.0859	0.569101	14.11

SNP	effect_allele WC	other_allele WC	eaf. WC	beta. WC	se. WC	pval. WC	beta. II	se. II	pval. II	F-statistics
rs7752202	T	C	0.145139	0.0177008	0.00253183	2.70E-12	0.0189	0.0368	0.6081	12.13
rs784257	C	T	0.812569	0.0161785	0.00230615	2.30E-12	-0.0145	0.0302	0.631499	14.99
rs7845090	A	G	0.709022	-0.0195133	0.00197942	6.30E-23	0.0125	0.0257	0.6272	40.10
rs7925100	A	G	0.396115	0.0140407	0.00182953	1.70E-14	-0.0123	0.025	0.6222	28.18
rs7952436	T	C	0.081988	-0.0289945	0.00326001	5.90E-19	0.0352	0.0509	0.4897	11.91
rs7966251	A	G	0.255065	-0.0115986	0.00205522	1.70E-08	0.0044	0.0252	0.8625	12.10
rs8013377	C	A	0.269606	-0.0166104	0.002018	1.90E-16	0.0108	0.0246	0.6605	26.68
rs80243702	A	G	0.160621	0.0151663	0.00245648	6.70E-10	-0.0461	0.0386	0.232	10.28
rs8078135	T	C	0.489933	-0.0101458	0.00179655	1.60E-08	-0.0324	0.0226	0.1519	15.94
rs8097672	T	A	0.145231	0.0167802	0.00255407	5.00E-11	0.0094	0.03	0.754899	10.72
rs815163	C	T	0.563195	-0.0131383	0.00179812	2.70E-13	0.0154	0.0227	0.4985	26.27
rs8192675	C	T	0.288645	0.0159923	0.00196858	4.50E-16	-0.0073	0.0256	0.7769	27.10
rs852042	G	A	0.758579	-0.0115951	0.00209164	3.00E-08	-0.0128	0.0267	0.6327	11.26
rs852983	A	G	0.459562	-9.83E-03	0.00179325	4.20E-08	-0.0166	0.0227	0.4641	14.93
rs862227	G	A	0.457951	-0.0109798	0.00179141	8.80E-10	0.0229	0.0228	0.3163	18.65
rs862320	T	C	0.409625	-0.0180264	0.0018218	4.40E-23	-0.0085	0.0228	0.709499	47.36
rs876605	G	A	0.739809	-0.0111634	0.00203598	4.20E-08	0.0152	0.026	0.5583	11.57
rs879620	T	C	0.613194	0.0194431	0.00184223	4.90E-26	0.0142	0.023	0.5381	52.85
rs883403	C	T	0.154427	-0.0176676	0.00247511	9.50E-13	0.0424	0.0271	0.1178	13.31
rs894736	G	A	0.362765	0.0157371	0.00186806	3.60E-17	0.0296	0.0227	0.1918	32.81
rs923994	G	A	0.783206	-0.0130094	0.0021745	2.20E-09	-0.016	0.0272	0.5571	12.16
rs9289630	C	G	0.389039	0.0144758	0.00183898	3.50E-15	-8.00E-04	0.0244	0.9727	29.46
rs9294260	A	G	0.476584	0.0131781	0.00179925	2.40E-13	0.0092	0.0227	0.6866	26.76
rs9316661	C	T	0.801292	-0.0156101	0.00224739	3.80E-12	0.0162	0.0285	0.5701	15.36

SNP	effect_allele WC	other_allele WC	eaf. WC	beta. WC	se. WC	pval. WC	beta. II	se. II	pval. II	F-statistics
rs9378676	C	A	0.234005	0.0130316	0.00211353	7.00E-10	-0.0506	0.0247	0.0400203	13.63
rs945211	C	G	0.615598	0.0100787	0.0018363	4.10E-08	0.003	0.0236	0.8994	14.26
rs9568867	A	G	0.129253	0.022614	0.00268971	4.20E-17	-0.0019	0.0326	0.954	15.91
rs9584870	C	T	0.366117	-0.0108139	0.00189234	1.10E-08	-0.0438	0.0241	0.0693601	15.16
rs9673839	G	A	0.490971	0.0109063	0.00179863	1.30E-09	-0.0188	0.0227	0.409	18.38
rs9814758	G	T	0.355886	-0.0111552	0.00187521	2.70E-09	0.0135	0.0243	0.577	16.22
rs9835772	T	A	0.243635	0.0121296	0.00208107	5.60E-09	0.0533	0.0269	0.0474602	12.52
rs9843653	C	T	0.511657	0.0195793	0.00178819	6.70E-28	-0.0119	0.0232	0.6072	59.92
rs9888533	T	C	0.538081	0.0108325	0.00182556	3.00E-09	0.0361	0.0231	0.1182	17.50
rs9902846	T	C	0.316036	0.0133756	0.00192994	4.20E-12	0.0039	0.0246	0.8745	20.77
rs9916444	G	C	0.34174	0.0114738	0.0018902	1.30E-09	0.0114	0.0248	0.647099	16.58

Abbreviations: SNP, single nucleotide polymorphism; se, standard error; pval, p-value; WC, waist circumference; II, intestinal infections.

Table S19. Detailed information about single-nucleotide polymorphisms of waist circumference on infections of the skin and subcutaneous tissue.

SNP	effect_allele WC	other_allele WC	eaf. WC	beta. WC	se. WC	pval. WC	beta. SSTI	se. SSTI	pval. SSTI	F-statistics
rs1013402	G	A	0.318423	0.0250906	0.00191723	3.90E-39	0.0066	0.0155	0.672201	74.35
rs10150482	A	G	0.22035	0.0219574	0.00216957	4.50E-24	0.0000	0.0172	0.9998	35.20
rs10184230	T	C	0.647671	-0.0121164	0.00186862	8.90E-11	0.0099	0.0159	0.533499	19.19
rs10236214	T	C	0.641963	0.013799	0.00187561	1.90E-13	0.0076	0.016	0.636999	24.88
rs10248298	A	C	0.366037	0.0132553	0.00185427	8.80E-13	0.0176	0.0151	0.242	23.72
rs1025065	G	T	0.639097	-0.010284	0.00186623	3.60E-08	0.0097	0.0161	0.5467	14.01
rs10257197	G	A	0.841665	-0.0154813	0.00245771	3.00E-10	-0.0069	0.0185	0.709499	10.58
rs10269774	A	G	0.326456	0.0119597	0.00190774	3.60E-10	0.0073	0.0159	0.6453	17.28
rs1037702	A	G	0.621776	-0.0101871	0.00184913	3.60E-08	-0.0198	0.0151	0.1907	14.28
rs10406327	G	C	0.478735	0.0103615	0.00179628	8.00E-09	0.0106	0.0147	0.4726	16.61
rs10423928	A	T	0.194353	-0.0265551	0.00225944	6.80E-32	-0.0245	0.0169	0.1474	43.26
rs10471636	A	G	0.508891	-0.010111	0.00182385	3.00E-08	0.0104	0.0148	0.481	15.36
rs10490869	T	A	0.209589	0.0163389	0.00220375	1.20E-13	-0.0369	0.0177	0.0376002	18.21
rs10499014	G	C	0.268616	-0.0132827	0.00202893	5.90E-11	-0.0077	0.0167	0.644899	16.84
rs1051613	A	G	0.544526	-9.95E-03	0.00179742	3.10E-08	-0.005	0.0148	0.737199	15.21
rs1056441	C	T	0.675421	0.0127063	0.00191349	3.10E-11	0.0497	0.0168	0.003176	19.33
rs10732335	C	A	0.442949	-0.0147414	0.00180443	3.10E-16	0.0068	0.0147	0.6425	32.94
rs10757898	A	G	0.520113	-9.97E-03	0.00181479	4.00E-08	-0.011	0.0148	0.4573	15.06
rs1078455	C	T	0.309501	0.0111735	0.00194801	9.70E-09	0.0083	0.0161	0.608001	14.06
rs10787738	T	C	0.254549	0.0153066	0.00208556	2.10E-13	-0.0079	0.0169	0.639501	20.44
rs10795418	G	A	0.664754	0.0127004	0.00190172	2.40E-11	-0.0108	0.0159	0.4996	19.88
rs10803762	A	G	0.677313	0.0119451	0.00191493	4.40E-10	-8.00E-04	0.0162	0.9627	17.01
rs10827380	T	C	0.314309	0.0116104	0.0019302	1.80E-09	-0.0139	0.015	0.357	15.60

SNP	effect_allele WC	other_allele WC	eaf. WC	beta. WC	se. WC	pval. WC	beta. SSTI	se. SSTI	pval. SSTI	F-statistics
rs10835676	G	C	0.240436	0.0123512	0.00209843	4.00E-09	-0.0252	0.0165	0.1272	12.65
rs10887578	C	G	0.497514	0.0106684	0.00179976	3.10E-09	-0.0309	0.0148	0.0361202	17.57
rs10938398	A	G	0.433578	0.0222115	0.00180756	1.00E-34	0.0339	0.0147	0.0212902	74.18
rs10947793	G	A	0.371789	-0.0127212	0.0018605	8.10E-12	0.006	0.0151	0.6936	21.84
rs10992854	C	T	0.681971	-0.0112545	0.00192897	5.40E-09	-0.0198	0.0155	0.2003	14.77
rs11012732	G	A	0.331675	0.0193536	0.00190117	2.40E-24	0.0311	0.0158	0.0490897	45.95
rs1108548	G	A	0.277245	0.0121715	0.00200003	1.20E-09	0.0102	0.0159	0.519501	14.84
rs11099020	T	C	0.640565	-0.0112449	0.00186604	1.70E-09	0.0031	0.0151	0.8353	16.72
rs1111817	G	C	0.365048	-0.0105597	0.00188105	2.00E-08	0.0106	0.0154	0.490999	14.61
rs111258054	T	C	0.183665	0.0159528	0.00234105	9.50E-12	-0.015	0.0165	0.3623	13.92
rs11150745	G	A	0.317708	-0.0160848	0.00192604	6.80E-17	-0.0473	0.0164	0.00399098	30.24
rs11162968	C	T	0.31619	0.0122283	0.00192412	2.10E-10	0.0047	0.0171	0.784	17.47
rs11165493	A	G	0.343044	0.0107576	0.00189335	1.30E-08	-0.0038	0.0152	0.8025	14.55
rs1117619	G	C	0.250101	-0.0120513	0.00206049	5.00E-09	0.0054	0.0166	0.747799	12.83
rs11196657	C	T	0.236715	0.0124451	0.00210599	3.40E-09	0.0156	0.0179	0.383	12.62
rs11215381	C	T	0.525856	0.0111759	0.00179363	4.60E-10	-0.0249	0.0147	0.0909599	19.36
rs11218510	A	G	0.40049	-0.0115323	0.00182872	2.90E-10	-0.0353	0.0153	0.0213098	19.10
rs11223204	G	A	0.434214	0.0122154	0.00180899	1.50E-11	0.0068	0.0156	0.6605	22.41
rs11614326	A	G	0.545054	-0.0102606	0.0018167	1.60E-08	0.0079	0.0148	0.595	15.82
rs11636611	T	C	0.502804	0.0107144	0.00179296	2.30E-09	0.0124	0.0147	0.4001	17.86
rs11639596	C	A	0.250798	-0.011872	0.00207809	1.10E-08	0.0024	0.016	0.8806	12.27
rs11653367	G	A	0.328129	-0.0151666	0.00191685	2.50E-15	-0.0341	0.0154	0.0268999	27.60
rs11666480	G	C	0.536273	0.0132885	0.00180641	1.90E-13	-0.0147	0.0149	0.3213	26.92
rs11675464	G	A	0.562997	0.0112729	0.00179869	3.70E-10	0.0389	0.0148	0.00856604	19.33

SNP	effect_allele WC	other_allele WC	eaf. WC	beta. WC	se. WC	pval. WC	beta. SSTI	se. SSTI	pval. SSTI	F-statistics
rs11704728	T	C	0.196347	0.0133307	0.00226433	3.90E-09	0.028	0.0179	0.1177	10.94
rs11757278	C	T	0.303836	-0.012784	0.00194427	4.90E-11	-0.0151	0.0151	0.3157	18.29
rs11767811	A	G	0.181372	-0.0150345	0.00231875	8.90E-11	-0.0118	0.0181	0.514801	12.48
rs11773362	T	C	0.336125	-0.0104772	0.0018937	3.20E-08	0.0041	0.0151	0.784401	13.66
rs11778934	G	C	0.536065	-0.0122168	0.00179945	1.10E-11	0.0041	0.0154	0.7896	22.93
rs11787216	T	C	0.369092	0.011505	0.0018915	1.20E-09	0.0142	0.0153	0.3532	17.23
rs1182199	A	C	0.3044	-0.0135196	0.00194469	3.60E-12	-0.0214	0.0153	0.1615	20.47
rs11824092	C	T	0.635754	0.012491	0.00187069	2.40E-11	0.0083	0.0148	0.5723	20.65
rs1183668	G	C	0.37014	-0.0120589	0.00186172	9.30E-11	-0.0059	0.0156	0.7077	19.56
rs11842871	T	G	0.259942	-0.0127878	0.00204439	4.00E-10	0.0026	0.0157	0.8711	15.05
rs1188209	G	A	0.551608	0.0102398	0.00181136	1.60E-08	0.0133	0.0148	0.3671	15.81
rs11898037	C	T	0.367606	0.0105694	0.00185324	1.20E-08	0.0133	0.0148	0.3709	15.12
rs1191600	A	C	0.59357	-0.0108005	0.00183345	3.80E-09	-0.0104	0.0149	0.4851	16.74
rs12001437	C	T	0.367701	0.0109331	0.00185543	3.80E-09	0.0374	0.0154	0.0151001	16.15
rs12072739	G	A	0.224447	0.0162106	0.00214325	3.90E-14	0.0251	0.0158	0.112	19.92
rs12140153	T	G	0.094226	-0.0265936	0.00313243	2.10E-17	-0.0131	0.0275	0.6332	12.30
rs1218824	A	G	0.661499	0.012491	0.00189332	4.20E-11	0.0086	0.0156	0.581	19.49
rs12287076	C	G	0.707082	0.0210638	0.00197238	1.30E-26	0.0054	0.0175	0.755499	47.25
rs12375196	A	C	0.424364	0.0132187	0.00182044	3.80E-13	0.006	0.0148	0.6864	25.76
rs12462975	A	G	0.329671	0.01754	0.00191777	5.90E-20	8.00E-04	0.0158	0.9619	36.97
rs12463617	C	A	0.828064	0.0431919	0.00236577	1.80E-74	0.0355	0.02	0.0762009	94.93
rs12478299	C	T	0.252015	-0.0115796	0.00206169	1.90E-08	-0.0047	0.0168	0.781101	11.89
rs12877270	A	G	0.442217	0.0117224	0.00181553	1.10E-10	0.0206	0.0147	0.1627	20.57
rs12880641	G	T	0.661581	-0.0138449	0.00188965	2.40E-13	-0.034	0.0152	0.0249597	24.04

SNP	effect_allele WC	other_allele WC	eaf. WC	beta. WC	se. WC	pval. WC	beta. SSTI	se. SSTI	pval. SSTI	F-statistics
rs12926311	C	G	0.353624	-0.0124576	0.00187714	3.20E-11	-0.0011	0.0162	0.9482	20.13
rs12926311	C	G	0.353624	-0.0124576	0.00187714	3.20E-11	0.0835	0.2478	0.7361	20.13
rs1296328	C	A	0.559023	-0.0131912	0.00180908	3.10E-13	-0.0255	0.0147	0.0829507	26.22
rs12983532	T	C	0.251127	-0.0149964	0.00209191	7.60E-13	0.0057	0.0175	0.7438	19.33
rs13033310	A	G	0.25275	0.0125512	0.00206781	1.30E-09	0.016	0.0172	0.3528	13.92
rs13047416	G	C	0.37702	-0.0139009	0.00185438	6.60E-14	4.00E-04	0.0147	0.9786	26.40
rs13163306	A	G	0.466047	-9.89E-03	0.00179351	3.50E-08	8.00E-04	0.0147	0.9579	15.13
rs13182474	C	G	0.318883	-0.0117225	0.00192056	1.00E-09	0.0127	0.0177	0.4739	16.18
rs1320903	A	G	0.319725	0.017072	0.00191652	5.20E-19	-0.0229	0.0162	0.1573	34.52
rs1321521	A	C	0.345235	0.0141428	0.00187867	5.10E-14	0.0016	0.0148	0.916	25.62
rs13264909	T	A	0.428948	-0.0122831	0.00181048	1.20E-11	0.0292	0.0147	0.0476102	22.55
rs1327259	G	A	0.387739	-0.0115864	0.0018403	3.10E-10	-0.021	0.0148	0.1558	18.82
rs13288841	A	G	0.32166	0.0190501	0.00191229	2.20E-23	0.0167	0.0152	0.2715	43.31
rs13322435	G	A	0.404446	-0.0169561	0.00182964	1.90E-20	-0.0107	0.0156	0.4923	41.38
rs13333747	C	T	0.182682	-0.022678	0.00232636	1.90E-22	-5.00E-04	0.0192	0.9778	28.38
rs1336486	G	T	0.328585	0.012489	0.00190819	6.00E-11	0.0126	0.0149	0.4003	18.90
rs13410783	G	A	0.36952	0.0141058	0.0018498	2.40E-14	-0.0014	0.0151	0.9278	27.10
rs13420048	A	C	0.365011	-0.0133504	0.00185957	7.00E-13	-0.0309	0.0149	0.03748	23.89
rs13427822	G	A	0.271197	-0.0141495	0.00203048	3.20E-12	-0.0255	0.0161	0.1131	19.20
rs1346841	A	G	0.40501	-0.0106038	0.00182614	6.40E-09	-0.0288	0.0159	0.0687907	16.25
rs1357079	C	T	0.570076	0.011183	0.00180784	6.20E-10	0.0099	0.015	0.5079	18.76
rs1360201	T	C	0.48157	0.0097942	0.00178924	4.40E-08	0.0129	0.0147	0.3834	14.96
rs1405261	A	T	0.434383	-9.85E-03	0.0018049	4.80E-08	-0.0028	0.0148	0.8512	14.64
rs1411432	C	A	0.186234	0.0150036	0.00230632	7.70E-11	0.0158	0.0193	0.4119	12.83

SNP	effect_allele WC	other_allele WC	eaf. WC	beta. WC	se. WC	pval. WC	beta. SSTI	se. SSTI	pval. SSTI	F-statistics
rs1436348	G	A	0.582801	0.0124929	0.00181185	5.40E-12	0.0034	0.0153	0.8255	23.12
rs1441098	T	A	0.54446	-9.91E-03	0.00179682	3.50E-08	-0.0135	0.0148	0.362	15.08
rs1441264	A	G	0.593703	0.0150095	0.00186203	7.60E-16	0.0151	0.0157	0.3344	31.35
rs1454687	G	C	0.515372	-0.0161615	0.00178705	1.50E-19	-0.0121	0.0148	0.4146	40.86
rs1458156	T	C	0.488413	0.0146036	0.00179105	3.50E-16	0.0118	0.0148	0.4237	33.23
rs1502317	T	C	0.276578	-0.0173687	0.0020032	4.30E-18	-0.0196	0.0167	0.2392	30.09
rs1559900	T	C	0.286025	0.0127734	0.00197882	1.10E-10	0.0047	0.0169	0.7796	17.02
rs156902	T	G	0.267387	-0.0138228	0.00243775	1.40E-08	-0.0201	0.0281	0.4748	12.60
rs1570298	T	A	0.743189	0.0120451	0.00204452	3.80E-09	-0.0087	0.0174	0.6176	13.25
rs1582931	A	G	0.473264	-0.0138548	0.00180588	1.70E-14	-0.0108	0.0147	0.4625	29.35
rs1609010	G	A	0.565725	0.0149337	0.00180594	1.30E-16	-0.004	0.0147	0.787501	33.60
rs1609303	A	T	0.631343	0.0152662	0.00185779	2.10E-16	0.02	0.0153	0.1922	31.44
rs1625623	T	C	0.371988	0.0107407	0.00187307	9.80E-09	0.0031	0.0153	0.8406	15.36
rs165656	C	G	0.517356	0.0103041	0.00180539	1.10E-08	0.0374	0.0148	0.01119	16.27
rs1657930	A	G	0.802922	-0.0142481	0.00224816	2.30E-10	-0.0171	0.0204	0.4027	12.71
rs1711171	C	T	0.749579	0.0175635	0.00206542	1.80E-17	-0.0079	0.0205	0.699401	27.15
rs17296856	C	A	0.280671	-0.0156228	0.00199629	5.00E-15	0.0075	0.0157	0.6342	24.73
rs1731246	T	G	0.757325	-0.0118366	0.0020818	1.30E-08	-0.0068	0.0173	0.6962	11.88
rs17446091	C	T	0.20186	0.0149141	0.00222979	2.30E-11	-0.0124	0.021	0.553	14.42
rs1752169	A	C	0.250696	0.0143082	0.00206705	4.50E-12	-0.0143	0.0152	0.3445	18.00
rs17681738	T	C	0.328589	0.0105421	0.00190864	3.30E-08	0.0043	0.0158	0.7866	13.46
rs1834144	A	C	0.373194	-0.0146152	0.00185609	3.40E-15	0.0139	0.0148	0.3469	29.01
rs1861410	T	C	0.555467	-0.016009	0.00180199	6.40E-19	0.0014	0.015	0.928	38.98
rs1902066	C	T	0.562316	0.0109526	0.00181038	1.40E-09	-0.0026	0.0148	0.8596	18.02

SNP	effect_allele WC	other_allele WC	eaf. WC	beta. WC	se. WC	pval. WC	beta. SSTI	se. SSTI	pval. SSTI	F-statistics
rs2020942	T	C	0.394892	0.0108469	0.00183573	3.40E-09	0.0164	0.0149	0.2686	16.69
rs2074881	T	C	0.168487	-0.0147612	0.00240193	8.00E-10	0.0054	0.0218	0.8055	10.58
rs2133561	T	A	0.611063	-0.0122441	0.00185284	3.90E-11	-0.0343	0.0154	0.0257798	20.76
rs215669	A	G	0.611654	-0.0125095	0.00184246	1.10E-11	-0.022	0.0164	0.1796	21.90
rs2161097	T	C	0.437804	0.0142773	0.00180004	2.20E-15	-0.0343	0.0151	0.02329	30.97
rs2172131	C	T	0.578709	-0.0121742	0.00181299	1.90E-11	0.0186	0.0147	0.2049	21.99
rs2180454	C	T	0.771831	0.0179823	0.00213539	3.70E-17	0.0184	0.0157	0.2412	24.98
rs2183947	A	G	0.225006	-0.0220783	0.00213628	4.90E-25	0.01	0.0174	0.5665	37.25
rs2225909	C	T	0.774104	0.015797	0.00213642	1.40E-13	0.0359	0.0191	0.0600606	19.12
rs2253310	G	C	0.626102	0.018205	0.00184715	6.50E-23	0.0156	0.0149	0.2961	45.48
rs2302209	T	C	0.288806	0.0198329	0.00197713	1.10E-23	0.0189	0.0168	0.2612	41.34
rs2306593	T	C	0.488298	-0.0152919	0.00179509	1.60E-17	-0.0042	0.0147	0.777499	36.27
rs2307111	C	T	0.394972	-0.0239722	0.0018299	3.30E-39	-0.0291	0.0149	0.0502296	82.04
rs2376885	A	G	0.324117	-0.0106223	0.00191252	2.80E-08	-0.0192	0.0151	0.2026	13.52
rs2439823	G	A	0.545601	0.0155254	0.00180145	6.80E-18	-0.0144	0.0148	0.3279	36.83
rs245767	G	A	0.730365	0.0145976	0.00201635	4.50E-13	0.0152	0.0157	0.3317	20.64
rs2470549	C	T	0.598143	-0.0119541	0.00182105	5.20E-11	0.0209	0.0151	0.1682	20.72
rs2470946	T	G	0.401445	0.011687	0.00182473	1.50E-10	0.0167	0.0151	0.2704	19.71
rs2482704	T	G	0.426761	-0.0115642	0.00180618	1.50E-10	0.0087	0.0152	0.566301	20.06
rs2568958	A	G	0.603683	0.0169206	0.00182184	1.60E-20	0.0034	0.0154	0.8246	41.28
rs2584205	A	G	0.733265	0.0111592	0.00202662	3.70E-08	0.0187	0.017	0.2711	11.86
rs2618039	T	A	0.381482	0.0120777	0.00183977	5.20E-11	0.0287	0.0148	0.0520895	20.34
rs2678204	G	T	0.34017	0.0157074	0.00188488	7.90E-17	-0.0039	0.0161	0.8107	31.18
rs2696309	C	T	0.720419	0.0112571	0.00199327	1.60E-08	-0.0145	0.0156	0.3538	12.85

SNP	effect_allele WC	other_allele WC	eaf. WC	beta. WC	se. WC	pval. WC	beta. SSTI	se. SSTI	pval. SSTI	F-statistics
rs2725371	G	A	0.696098	-0.0154452	0.00195255	2.60E-15	-0.0186	0.0168	0.2676	26.48
rs2744938	G	A	0.147512	0.0319686	0.00251588	5.40E-37	-0.0115	0.0183	0.530401	40.61
rs28350	G	A	0.820658	-0.0139376	0.0023374	2.50E-09	0.004	0.0189	0.8322	10.47
rs28366156	C	T	0.130582	-0.0187659	0.00265145	1.50E-12	0.0209	0.0288	0.4682	11.37
rs28375268	T	G	0.645058	-0.0130789	0.00187766	3.30E-12	0.0082	0.0155	0.5954	22.22
rs28489620	A	G	0.29035	-0.0124131	0.00198899	4.40E-10	-0.016	0.0161	0.3206	16.05
rs28580375	G	C	0.21874	0.0126069	0.00216777	6.00E-09	0.031	0.0174	0.0756798	11.56
rs2861692	C	T	0.275371	-0.0166517	0.00199772	7.70E-17	0.0196	0.0162	0.2258	27.73
rs2903738	T	A	0.221402	-0.0133061	0.00215663	6.80E-10	-0.006	0.0172	0.7278	13.12
rs308911	G	A	0.714436	-0.0114749	0.00198055	6.90E-09	-0.0321	0.0164	0.0501499	13.70
rs3113509	T	C	0.731966	-0.0123292	0.00201975	1.00E-09	-0.0233	0.0163	0.1544	14.62
rs319775	C	T	0.608753	0.0101556	0.0018335	3.00E-08	-0.0213	0.015	0.1554	14.61
rs3212038	G	A	0.328517	0.0126546	0.00191072	3.50E-11	-0.0053	0.0149	0.724201	19.35
rs34045288	T	C	0.334413	0.0203746	0.00189485	5.80E-27	0.0321	0.0152	0.0345502	51.47
rs34140906	C	T	0.17033	-0.0179391	0.00238486	5.40E-14	0.0084	0.0185	0.649201	15.99
rs34234296	A	G	0.392393	-0.0131214	0.00184833	1.30E-12	0.0174	0.016	0.2763	24.03
rs34483452	A	C	0.136355	0.0270483	0.0026269	7.30E-25	-0.0391	0.0225	0.0831209	24.97
rs34517439	A	C	0.121789	0.0305491	0.00276165	1.90E-28	0.0459	0.0222	0.0383902	26.18
rs347551	G	C	0.472292	0.0126307	0.00181978	3.90E-12	0.0212	0.0149	0.1542	24.01
rs34882821	T	G	0.338577	0.0106237	0.00189644	2.10E-08	0.0209	0.0162	0.1966	14.06
rs34994596	C	T	0.297289	-0.014559	0.00195866	1.10E-13	-0.0031	0.0167	0.8535	23.09
rs35023999	C	A	0.508297	-0.0113409	0.00178977	2.40E-10	0.0034	0.0147	0.8177	20.07
rs35243581	T	C	0.317318	0.0173643	0.00192251	1.70E-19	0.0136	0.0159	0.3906	35.35
rs35681682	C	T	0.407729	-0.0101534	0.00177268	1.00E-08	0.0029	0.0148	0.8433	15.85

SNP	effect_allele WC	other_allele WC	eaf. WC	beta. WC	se. WC	pval. WC	beta. SSTI	se. SSTI	pval. SSTI	F-statistics
rs35882248	T	C	0.317213	0.0157088	0.00191956	2.80E-16	0.0013	0.0158	0.9336	29.01
rs36061954	T	C	0.39881	0.0114574	0.00182669	3.60E-10	-0.0032	0.0151	0.8323	18.87
rs36140	C	A	0.635311	0.0111762	0.001867	2.10E-09	0.0381	0.0154	0.0137101	16.61
rs36165342	C	T	0.478618	0.0107118	0.00178943	2.10E-09	-0.0064	0.015	0.67	17.88
rs3764002	T	C	0.261552	-0.0160772	0.00203505	2.80E-15	0.02	0.0155	0.198	24.11
rs3768321	T	G	0.196525	0.0176726	0.00224849	3.80E-15	-0.0039	0.0199	0.8451	19.51
rs3784692	T	C	0.601752	0.0185824	0.00182662	2.60E-24	-0.0074	0.0153	0.628801	49.61
rs3806114	A	G	0.668366	-0.0108144	0.00192005	1.80E-08	-0.0043	0.0172	0.803	14.06
rs3807566	T	G	0.438304	-0.012149	0.00180583	1.70E-11	-0.0227	0.0152	0.1344	22.29
rs3814883	T	C	0.482382	0.0239816	0.00179535	1.10E-40	-0.0059	0.0149	0.6921	89.12
rs3816760	A	G	0.307437	0.0133538	0.00193927	5.70E-12	-0.0082	0.0168	0.6234	20.19
rs3826408	T	C	0.456788	0.0112249	0.00179579	4.10E-10	-0.0192	0.0147	0.193	19.39
rs3845344	T	C	0.391147	0.0107371	0.00182845	4.30E-09	0.0343	0.0148	0.0209002	16.42
rs3866805	A	C	0.355681	0.010421	0.00187021	2.50E-08	-0.0089	0.0155	0.566301	14.23
rs3935190	A	G	0.536793	-0.0126195	0.00180625	2.80E-12	-0.0205	0.0148	0.1642	24.28
rs3936510	T	G	0.201255	0.0137147	0.0022268	7.30E-10	0.0071	0.0212	0.738599	12.20
rs3949781	A	T	0.538137	0.0115117	0.00180564	1.80E-10	0.0089	0.0148	0.5454	20.21
rs40067	A	G	0.170153	-0.0156125	0.00238663	6.10E-11	-0.029	0.0181	0.1087	12.09
rs4017425	T	C	0.47017	-0.0101269	0.00179274	1.60E-08	-0.0402	0.0151	0.00791407	15.90
rs4072917	A	G	0.474253	0.0117189	0.0017994	7.40E-11	-0.0049	0.0148	0.741401	21.15
rs4075353	A	G	0.344101	-0.0105885	0.00189213	2.20E-08	-0.0098	0.0154	0.5214	14.14
rs4290163	T	G	0.392697	0.0113777	0.00183305	5.40E-10	0.033	0.0151	0.0289601	18.38
rs429343	G	A	0.576576	-0.0124509	0.00180931	5.90E-12	-0.02	0.0148	0.175	23.12
rs429358	C	T	0.154146	-0.0271422	0.00248126	7.50E-28	-0.0152	0.0191	0.4262	31.21

SNP	effect_allele WC	other_allele WC	eaf. WC	beta. WC	se. WC	pval. WC	beta. SSTI	se. SSTI	pval. SSTI	F-statistics
rs4419475	T	A	0.407376	0.0112635	0.00181962	6.00E-10	0.0123	0.0147	0.4044	18.50
rs4456769	T	C	0.333423	0.0134001	0.0019003	1.80E-12	0.0079	0.0154	0.6081	22.10
rs4469245	T	A	0.662728	-0.0115211	0.00189126	1.10E-09	0.0064	0.0161	0.6887	16.59
rs4525978	T	C	0.734555	-0.0113759	0.00203018	2.10E-08	0.0119	0.0176	0.4987	12.24
rs4527444	G	A	0.541317	0.0105243	0.00179433	4.50E-09	-0.0156	0.0147	0.2872	17.08
rs4552632	A	G	0.616568	-0.0101695	0.00184128	3.30E-08	-0.0126	0.0155	0.4155	14.42
rs4689465	C	T	0.525445	-0.0108689	0.00178976	1.30E-09	-0.0199	0.0147	0.1754	18.39
rs4706004	G	A	0.217047	-0.0134593	0.00216831	5.40E-10	-0.0362	0.0167	0.0301898	13.10
rs4718964	T	G	0.413175	0.0122483	0.00182202	1.80E-11	-0.0102	0.015	0.4959	21.91
rs4742782	G	C	0.315942	0.0124912	0.00192172	8.00E-11	0.0147	0.0151	0.3296	18.26
rs484455	A	G	0.481363	-0.0115336	0.00179638	1.40E-10	-0.0072	0.0151	0.636099	20.58
rs4851283	G	C	0.684805	-0.0173961	0.00193525	2.50E-19	0.0139	0.0178	0.4351	34.88
rs4856720	C	G	0.53938	0.0114342	0.0017918	1.80E-10	-0.0102	0.0147	0.4872	20.24
rs4876611	G	A	0.720239	0.0150085	0.0019947	5.30E-14	0.0397	0.0159	0.0127201	22.82
rs4900715	A	G	0.507101	-0.0114279	0.00179331	1.90E-10	-0.0202	0.0149	0.1754	20.30
rs4908672	T	C	0.393063	0.0113966	0.00183009	4.70E-10	-0.0043	0.0148	0.7712	18.50
rs520478	T	G	0.701359	-0.0125051	0.00197262	2.30E-10	-0.0046	0.0174	0.7903	16.84
rs539515	C	A	0.204929	0.0378223	0.00221181	1.50E-65	0.0081	0.0192	0.6723	95.31
rs55726687	A	G	0.209735	0.0199369	0.00219529	1.10E-19	0.0304	0.0179	0.0898504	27.34
rs557951	G	T	0.312953	0.0120789	0.00193163	4.00E-10	-0.0058	0.0167	0.726599	16.82
rs559231	T	G	0.393054	0.0107518	0.00184107	5.20E-09	0.0309	0.0149	0.0378704	16.27
rs56094641	G	A	0.404591	0.057552	0.00182234	1.00E-200	0.018	0.0149	0.2271	481.03
rs56803094	G	A	0.226708	-0.0127745	0.00214599	2.60E-09	0.001	0.0165	0.9511	12.42

SNP	effect_allele WC	other_allele WC	eaf. WC	beta. WC	se. WC	pval. WC	beta. SSTI	se. SSTI	pval. SSTI	F-statistics
rs57636386	C	T	0.083822	-0.0309067	0.00324088	1.50E-21	-0.0569	0.0337	0.0917191	13.97
rs587271	T	C	0.686869	0.0118054	0.0020079	4.10E-09	0.0059	0.016	0.7098	14.87
rs58862095	T	C	0.419271	-0.0165812	0.00181678	7.10E-20	-0.0177	0.0147	0.2285	40.57
rs588660	A	G	0.584119	0.0155276	0.00181174	1.00E-17	-0.0089	0.015	0.5532	35.69
rs59068084	T	G	0.410212	0.0101403	0.00181938	2.50E-08	9.00E-04	0.0151	0.9521	15.03
rs59104534	T	C	0.298501	0.0107189	0.00196197	4.70E-08	0.006	0.0157	0.7032	12.50
rs6001877	A	G	0.339906	-0.010579	0.00189657	2.40E-08	-0.005	0.0152	0.7422	13.96
rs6069037	A	C	0.731389	-0.011125	0.00202001	3.60E-08	-0.0018	0.0172	0.9149	11.92
rs61223906	A	G	0.339313	-0.0108726	0.00188655	8.30E-09	-0.0029	0.0152	0.8512	14.89
rs61813324	T	C	0.135713	0.0219762	0.00264443	9.50E-17	0.0186	0.0204	0.3621	16.20
rs61903695	G	A	0.254942	0.0134514	0.00205497	5.90E-11	-0.0285	0.0171	0.0959489	16.28
rs61969511	A	G	0.278938	0.011857	0.0020127	3.80E-09	0.0048	0.0152	0.752501	13.96
rs61992671	G	A	0.491969	-0.0130005	0.00187164	3.80E-12	-0.0061	0.015	0.682801	24.12
rs62243489	G	T	0.259239	-0.0155648	0.00204876	3.00E-14	0.0332	0.0162	0.0407803	22.17
rs62261725	G	A	0.326101	-0.0148698	0.00190872	6.70E-15	0.0064	0.0153	0.6766	26.68
rs6493498	C	T	0.54539	-0.013082	0.001805	4.20E-13	0.0177	0.015	0.2363	26.05
rs649458	A	T	0.860102	-0.018179	0.00256951	1.50E-12	-0.0406	0.0231	0.0786901	12.05
rs6536575	C	T	0.51907	0.0108432	0.00178995	1.40E-09	0.0175	0.0149	0.2387	18.32
rs6551304	G	A	0.831916	0.016827	0.00239416	2.10E-12	0.0176	0.0213	0.4086	13.82
rs6567160	C	T	0.232725	0.0451644	0.00211824	7.19E-101	0.034	0.019	0.0731206	162.41
rs6575340	A	G	0.636022	0.0161584	0.00186502	4.60E-18	0.0154	0.0152	0.3103	34.76
rs6669341	G	A	0.58271	-0.0125214	0.0018097	4.50E-12	-8.00E-04	0.0151	0.9567	23.28
rs6682438	C	T	0.673097	0.0124771	0.00190203	5.40E-11	0.0059	0.0156	0.7031	18.94

SNP	effect_allele WC	other_allele WC	eaf. WC	beta. WC	se. WC	pval. WC	beta. SSTI	se. SSTI	pval. SSTI	F-statistics
rs6693294	G	A	0.688535	-0.0170126	0.00192843	1.10E-18	-0.0075	0.0152	0.6215	33.38
rs6739755	G	A	0.603358	-0.0159241	0.00182885	3.10E-18	-0.0195	0.015	0.192	36.29
rs67609008	C	T	0.283612	0.011102	0.00199233	2.50E-08	-0.0173	0.0191	0.3659	12.62
rs6791983	A	C	0.750006	0.0123148	0.00205988	2.30E-09	0.0088	0.0174	0.612901	13.40
rs6799080	A	G	0.353391	0.0102307	0.00186721	4.30E-08	-0.0046	0.0148	0.7568	13.72
rs6846041	G	C	0.320508	0.0123073	0.00191467	1.30E-10	0.0053	0.0169	0.755499	18.00
rs6849518	T	C	0.12428	0.0218636	0.00270973	7.10E-16	0.0234	0.0197	0.2339	14.17
rs6938973	C	T	0.601488	0.0120106	0.001827	4.90E-11	-0.0073	0.0157	0.6447	20.72
rs7034554	G	A	0.373813	-0.0112644	0.00184813	1.10E-09	-0.0196	0.0162	0.2273	17.39
rs703984	C	G	0.414809	-0.011472	0.00181967	2.90E-10	0.0244	0.0151	0.1062	19.30
rs704061	C	T	0.455057	0.0146223	0.00179659	4.00E-16	0.0227	0.0151	0.1334	32.86
rs7070670	T	C	0.327941	-0.0120318	0.00191737	3.50E-10	0.012	0.0164	0.4657	17.36
rs7115013	T	C	0.442738	-0.0106394	0.00180461	3.70E-09	-0.0042	0.015	0.779301	17.15
rs7132908	A	G	0.384454	0.0215129	0.00183998	1.40E-31	0.0054	0.0152	0.719701	64.71
rs7169847	T	G	0.635678	-0.0102703	0.00186771	3.80E-08	-0.0185	0.0147	0.2096	14.01
rs7171864	A	G	0.660202	0.0128894	0.00189898	1.10E-11	-0.0015	0.0161	0.9255	20.67
rs7206608	G	C	0.321623	0.0120004	0.00191641	3.80E-10	0.026	0.0153	0.0894994	17.11
rs7218014	C	T	0.197315	0.0224089	0.00225393	2.70E-23	0.0236	0.0172	0.1706	31.31
rs7259070	C	T	0.596078	0.0153811	0.00184133	6.60E-17	-0.0105	0.0149	0.4787	33.60
rs72634826	A	G	0.259865	-0.0148957	0.00206456	5.40E-13	-0.0263	0.0181	0.1454	20.02
rs72892910	T	G	0.172259	0.0301404	0.00237148	5.20E-37	0.0291	0.0183	0.1107	46.07
rs72976986	A	G	0.190131	-0.0157587	0.00230334	7.80E-12	0.0013	0.0202	0.9504	14.42
rs73052033	C	T	0.184916	-0.0210096	0.00230513	7.90E-20	-0.003	0.0199	0.8792	25.04
rs73068448	T	C	0.170691	-0.0153436	0.00241309	2.00E-10	-1.00E-04	0.0221	0.9977	11.45

SNP	effect_allele WC	other_allele WC	eaf. WC	beta. WC	se. WC	pval. WC	beta. SSTI	se. SSTI	pval. SSTI	F-statistics
rs73142879	T	C	0.192309	-0.0241942	0.00228107	2.80E-26	0.0088	0.0202	0.6635	34.95
rs7324067	C	T	0.761254	0.0121297	0.00210064	7.70E-09	-0.0046	0.0165	0.781	12.12
rs735033	G	A	0.605139	-0.0102658	0.00184312	2.50E-08	-0.0087	0.0148	0.554799	14.83
rs7372674	A	C	0.357239	0.0118947	0.00186339	1.70E-10	-0.0021	0.016	0.8951	18.71
rs7377083	A	C	0.431061	0.0143396	0.00181913	3.20E-15	0.0255	0.0148	0.0854004	30.48
rs73985439	C	A	0.307294	0.0124046	0.00193905	1.60E-10	0.0032	0.016	0.8403	17.42
rs7442885	G	C	0.214033	-0.0204724	0.00218339	6.80E-21	-0.0134	0.0189	0.4801	29.58
rs7498044	A	G	0.217312	-0.0151498	0.00219298	4.90E-12	-0.004	0.017	0.8133	16.24
rs7498665	G	A	0.399688	0.026616	0.00182769	4.90E-48	0.0146	0.0149	0.3271	101.79
rs7519259	A	G	0.528392	0.0126703	0.00179634	1.70E-12	0.0087	0.0148	0.5585	24.80
rs7537581	A	C	0.531758	0.0107362	0.00180248	2.60E-09	-0.0224	0.0148	0.1305	17.67
rs7539903	A	T	0.615583	-0.0107184	0.00183572	5.30E-09	0.0258	0.015	0.0849493	16.14
rs756717	A	G	0.399071	-0.0107152	0.00184896	6.80E-09	0.0145	0.0151	0.3364	16.11
rs76286777	C	T	0.217783	0.0234759	0.0021623	1.80E-27	-0.0019	0.0214	0.9292	40.16
rs7630382	T	C	0.531552	0.013362	0.00179336	9.30E-14	-2.00E-04	0.0148	0.9883	27.65
rs765876	G	A	0.489495	-9.81E-03	0.00178785	4.10E-08	-0.0073	0.015	0.6242	15.04
rs7707394	A	G	0.357264	-0.0167827	0.00186285	2.10E-19	-0.029	0.0152	0.0562199	37.28
rs7708584	G	A	0.572375	-0.0121976	0.00180534	1.40E-11	-0.0082	0.015	0.5833	22.35
rs77165542	T	C	0.035495	-0.0700663	0.00488088	9.90E-47	-0.0255	0.0569	0.6544	14.11
rs7752202	T	C	0.145139	0.0177008	0.00253183	2.70E-12	-0.0454	0.024	0.0581206	12.13
rs784257	C	T	0.812569	0.0161785	0.00230615	2.30E-12	0.0143	0.0196	0.4673	14.99
rs7845090	A	G	0.709022	-0.0195133	0.00197942	6.30E-23	-0.0335	0.0167	0.0448405	40.10
rs7925100	A	G	0.396115	0.0140407	0.00182953	1.70E-14	0.0261	0.0162	0.1072	28.18
rs7952436	T	C	0.081988	-0.0289945	0.00326001	5.90E-19	-0.0624	0.0334	0.0615503	11.91

SNP	effect_allele WC	other_allele WC	eaf. WC	beta. WC	se. WC	pval. WC	beta. SSTI	se. SSTI	pval. SSTI	F-statistics
rs7966251	A	G	0.255065	-0.0115986	0.00205522	1.70E-08	-0.0302	0.0164	0.0654199	12.10
rs8013377	C	A	0.269606	-0.0166104	0.002018	1.90E-16	-0.0056	0.016	0.7262	26.68
rs80243702	A	G	0.160621	0.0151663	0.00245648	6.70E-10	-0.0181	0.025	0.4683	10.28
rs8078135	T	C	0.489933	-0.0101458	0.00179655	1.60E-08	-0.0273	0.0147	0.0631801	15.94
rs8097672	T	A	0.145231	0.0167802	0.00255407	5.00E-11	0.005	0.0194	0.7949	10.72
rs815163	C	T	0.563195	-0.0131383	0.00179812	2.70E-13	0.0054	0.0147	0.7133	26.27
rs8192675	C	T	0.288645	0.0159923	0.00196858	4.50E-16	0.0036	0.0166	0.8288	27.10
rs852042	G	A	0.758579	-0.0115951	0.00209164	3.00E-08	-0.0093	0.0173	0.5935	11.26
rs852983	A	G	0.459562	-9.83E-03	0.00179325	4.20E-08	-0.0018	0.0147	0.9006	14.93
rs862227	G	A	0.457951	-0.0109798	0.00179141	8.80E-10	0.0101	0.0148	0.4967	18.65
rs862320	T	C	0.409625	-0.0180264	0.0018218	4.40E-23	-0.0134	0.0148	0.3665	47.36
rs876605	G	A	0.739809	-0.0111634	0.00203598	4.20E-08	0.0296	0.0168	0.0789205	11.57
rs879620	T	C	0.613194	0.0194431	0.00184223	4.90E-26	-0.0136	0.0149	0.3622	52.85
rs883403	C	T	0.154427	-0.0176676	0.00247511	9.50E-13	0.0047	0.0176	0.7886	13.31
rs894736	G	A	0.362765	0.0157371	0.00186806	3.60E-17	-0.0075	0.0147	0.6111	32.81
rs923994	G	A	0.783206	-0.0130094	0.0021745	2.20E-09	-0.0173	0.0177	0.3281	12.16
rs9289630	C	G	0.389039	0.0144758	0.00183898	3.50E-15	-0.0209	0.0158	0.1866	29.46
rs9294260	A	G	0.476584	0.0131781	0.00179925	2.40E-13	0.0078	0.0147	0.5962	26.76
rs9316661	C	T	0.801292	-0.0156101	0.00224739	3.80E-12	-0.0187	0.0186	0.3136	15.36
rs9378676	C	A	0.234005	0.0130316	0.00211353	7.00E-10	5.00E-04	0.016	0.975	13.63
rs945211	C	G	0.615598	0.0100787	0.0018363	4.10E-08	-0.0058	0.0153	0.704301	14.26
rs9568867	A	G	0.129253	0.022614	0.00268971	4.20E-17	0.0027	0.0213	0.897	15.91
rs9584870	C	T	0.366117	-0.0108139	0.00189234	1.10E-08	-0.0126	0.0157	0.4234	15.16
rs9673839	G	A	0.490971	0.0109063	0.00179863	1.30E-09	0.0122	0.0148	0.4101	18.38

SNP	effect_allele WC	other_allele WC	eaf. WC	beta. WC	se. WC	pval. WC	beta. SSTI	se. SSTI	pval. SSTI	F-statistics
rs9814758	G	T	0.355886	-0.0111552	0.00187521	2.70E-09	-3.00E-04	0.0158	0.9867	16.22
rs9835772	T	A	0.243635	0.0121296	0.00208107	5.60E-09	-0.0141	0.0175	0.4198	12.52
rs9843653	C	T	0.511657	0.0195793	0.00178819	6.70E-28	-0.0017	0.015	0.9075	59.92
rs9888533	T	C	0.538081	0.0108325	0.00182556	3.00E-09	-0.0028	0.015	0.8542	17.50
rs9902846	T	C	0.316036	0.0133756	0.00192994	4.20E-12	-0.0028	0.016	0.863	20.77
rs9916444	G	C	0.34174	0.0114738	0.0018902	1.30E-09	0.035	0.0161	0.0299502	16.58

Abbreviations: SNP, single nucleotide polymorphism; se, standard error; pval, p-value; WC, waist circumference; SSTI, infections of the skin and subcutaneous tissue.

Table S20. Detailed information about single-nucleotide polymorphisms of waist circumference on acute lower respiratory infections.

SNP	effect_allele WC	other_allele WC	eaf. WC	beta. WC	se. WC	pval. WC	beta.ALRI	se. ALRI	pval.ALRI	F-statistics
rs1013402	G	A	0.318423	0.0250906	0.00191723	3.90E-39	-0.0177	0.0155	0.2529	74.35
rs10150482	A	G	0.22035	0.0219574	0.00216957	4.50E-24	-0.0044	0.0172	0.7975	35.20
rs10184230	T	C	0.647671	-0.0121164	0.00186862	8.90E-11	-0.0254	0.0158	0.1082	19.19
rs10236214	T	C	0.641963	0.013799	0.00187561	1.90E-13	-0.0212	0.0159	0.1837	24.88
rs10248298	A	C	0.366037	0.0132553	0.00185427	8.80E-13	0.0081	0.015	0.589899	23.72
rs1025065	G	T	0.639097	-0.010284	0.00186623	3.60E-08	-0.0033	0.016	0.8374	14.01
rs10257197	G	A	0.841665	-0.0154813	0.00245771	3.00E-10	0.0079	0.0184	0.666801	10.58
rs10269774	A	G	0.326456	0.0119597	0.00190774	3.60E-10	-0.0165	0.0159	0.2989	17.28
rs1037702	A	G	0.621776	-0.0101871	0.00184913	3.60E-08	-0.0031	0.015	0.8353	14.28
rs10406327	G	C	0.478735	0.0103615	0.00179628	8.00E-09	7.00E-04	0.0147	0.9611	16.61
rs10423928	A	T	0.194353	-0.0265551	0.00225944	6.80E-32	-0.0242	0.0168	0.1496	43.26
rs10471636	A	G	0.508891	-0.010111	0.00182385	3.00E-08	1.00E-04	0.0148	0.9937	15.36
rs10490869	T	A	0.209589	0.0163389	0.00220375	1.20E-13	-0.0046	0.0177	0.7957	18.21
rs10499014	G	C	0.268616	-0.0132827	0.00202893	5.90E-11	0.0093	0.0167	0.578	16.84
rs1051613	A	G	0.544526	-9.95E-03	0.00179742	3.10E-08	0.0139	0.0147	0.3451	15.21
rs1056441	C	T	0.675421	0.0127063	0.00191349	3.10E-11	0.0182	0.0168	0.2769	19.33
rs10732335	C	A	0.442949	-0.0147414	0.00180443	3.10E-16	-0.0035	0.0147	0.8113	32.94
rs10757898	A	G	0.520113	-9.97E-03	0.00181479	4.00E-08	-0.0111	0.0147	0.4489	15.06
rs1078455	C	T	0.309501	0.0111735	0.00194801	9.70E-09	0.0143	0.0161	0.3739	14.06
rs10787738	T	C	0.254549	0.0153066	0.00208556	2.10E-13	0.0122	0.0169	0.4696	20.44
rs10795418	G	A	0.664754	0.0127004	0.00190172	2.40E-11	-0.0167	0.0159	0.2919	19.88
rs10803762	A	G	0.677313	0.0119451	0.00191493	4.40E-10	0.0326	0.0161	0.0432803	17.01
rs10827380	T	C	0.314309	0.0116104	0.0019302	1.80E-09	0.0448	0.015	0.00278202	15.60

SNP	effect_allele WC	other_allele WC	eaf. WC	beta. WC	se. WC	pval. WC	beta.ALRI	se. ALRI	pval.ALRI	F-statistics
rs10835676	G	C	0.240436	0.0123512	0.00209843	4.00E-09	0.0071	0.0165	0.6675	12.65
rs10887578	C	G	0.497514	0.0106684	0.00179976	3.10E-09	-0.0045	0.0147	0.757801	17.57
rs10938398	A	G	0.433578	0.0222115	0.00180756	1.00E-34	0.0045	0.0147	0.7565	74.18
rs10947793	G	A	0.371789	-0.0127212	0.0018605	8.10E-12	0.011	0.0151	0.4644	21.84
rs10992854	C	T	0.681971	-0.0112545	0.00192897	5.40E-09	-0.0049	0.0154	0.750501	14.77
rs11012732	G	A	0.331675	0.0193536	0.00190117	2.40E-24	0.0062	0.0158	0.6946	45.95
rs1108548	G	A	0.277245	0.0121715	0.00200003	1.20E-09	-0.0105	0.0158	0.5078	14.84
rs11099020	T	C	0.640565	-0.0112449	0.00186604	1.70E-09	-0.0076	0.015	0.612999	16.72
rs1111817	G	C	0.365048	-0.0105597	0.00188105	2.00E-08	-0.0065	0.0153	0.6705	14.61
rs111258054	T	C	0.183665	0.0159528	0.00234105	9.50E-12	-0.0199	0.0164	0.2259	13.92
rs11150745	G	A	0.317708	-0.0160848	0.00192604	6.80E-17	0.0014	0.0163	0.9323	30.24
rs11162968	C	T	0.31619	0.0122283	0.00192412	2.10E-10	0.0133	0.0171	0.4342	17.47
rs11165493	A	G	0.343044	0.0107576	0.00189335	1.30E-08	-0.0174	0.0151	0.2493	14.55
rs1117619	G	C	0.250101	-0.0120513	0.00206049	5.00E-09	0.0162	0.0166	0.33	12.83
rs11196657	C	T	0.236715	0.0124451	0.00210599	3.40E-09	0.0427	0.0178	0.0164801	12.62
rs11215381	C	T	0.525856	0.0111759	0.00179363	4.60E-10	0.0038	0.0147	0.794301	19.36
rs11218510	A	G	0.40049	-0.0115323	0.00182872	2.90E-10	0.0068	0.0153	0.654201	19.10
rs11223204	G	A	0.434214	0.0122154	0.00180899	1.50E-11	-0.0114	0.0155	0.4641	22.41
rs11614326	A	G	0.545054	-0.0102606	0.0018167	1.60E-08	-0.0189	0.0148	0.2007	15.82
rs11636611	T	C	0.502804	0.0107144	0.00179296	2.30E-09	-6.00E-04	0.0146	0.9647	17.86
rs11639596	C	A	0.250798	-0.011872	0.00207809	1.10E-08	2.00E-04	0.0159	0.989	12.27
rs11653367	G	A	0.328129	-0.0151666	0.00191685	2.50E-15	0.023	0.0153	0.1332	27.60
rs11666480	G	C	0.536273	0.0132885	0.00180641	1.90E-13	-0.0094	0.0148	0.5252	26.92
rs11675464	G	A	0.562997	0.0112729	0.00179869	3.70E-10	0.0116	0.0147	0.4323	19.33

SNP	effect_allele WC	other_allele WC	eaf. WC	beta. WC	se. WC	pval. WC	beta.ALRI	se. ALRI	pval.ALRI	F-statistics
rs11704728	T	C	0.196347	0.0133307	0.00226433	3.90E-09	0.0245	0.0178	0.1695	10.94
rs11757278	C	T	0.303836	-0.012784	0.00194427	4.90E-11	-0.0021	0.015	0.8906	18.29
rs11767811	A	G	0.181372	-0.0150345	0.00231875	8.90E-11	-0.0118	0.018	0.515	12.48
rs11773362	T	C	0.336125	-0.0104772	0.0018937	3.20E-08	-0.0185	0.0151	0.2213	13.66
rs11778934	G	C	0.536065	-0.0122168	0.00179945	1.10E-11	0.0285	0.0153	0.0627798	22.93
rs11787216	T	C	0.369092	0.011505	0.0018915	1.20E-09	0.0345	0.0153	0.0238902	17.23
rs1182199	A	C	0.3044	-0.0135196	0.00194469	3.60E-12	-0.0059	0.0152	0.696299	20.47
rs11824092	C	T	0.635754	0.012491	0.00187069	2.40E-11	0.0142	0.0147	0.3342	20.65
rs1183668	G	C	0.37014	-0.0120589	0.00186172	9.30E-11	0.005	0.0156	0.7492	19.56
rs11842871	T	G	0.259942	-0.0127878	0.00204439	4.00E-10	0.0118	0.0157	0.4533	15.05
rs1188209	G	A	0.551608	0.0102398	0.00181136	1.60E-08	-0.0303	0.0147	0.0391399	15.81
rs11898037	C	T	0.367606	0.0105694	0.00185324	1.20E-08	0.0017	0.0148	0.906	15.12
rs1191600	A	C	0.59357	-0.0108005	0.00183345	3.80E-09	-0.0255	0.0149	0.0862601	16.74
rs12001437	C	T	0.367701	0.0109331	0.00185543	3.80E-09	0.0144	0.0153	0.3483	16.15
rs12072739	G	A	0.224447	0.0162106	0.00214325	3.90E-14	0.0233	0.0157	0.1388	19.92
rs12140153	T	G	0.094226	-0.0265936	0.00313243	2.10E-17	0.0098	0.0274	0.7206	12.30
rs1218824	A	G	0.661499	0.012491	0.00189332	4.20E-11	-0.003	0.0156	0.8485	19.49
rs12287076	C	G	0.707082	0.0210638	0.00197238	1.30E-26	-0.0234	0.0174	0.1793	47.25
rs12375196	A	C	0.424364	0.0132187	0.00182044	3.80E-13	-0.023	0.0147	0.1181	25.76
rs12462975	A	G	0.329671	0.01754	0.00191777	5.90E-20	-0.0513	0.0158	0.001163	36.97
rs12463617	C	A	0.828064	0.0431919	0.00236577	1.80E-74	0.0212	0.02	0.2881	94.93
rs12478299	C	T	0.252015	-0.0115796	0.00206169	1.90E-08	0.0119	0.0167	0.4779	11.89
rs12877270	A	G	0.442217	0.0117224	0.00181553	1.10E-10	-0.0112	0.0147	0.4448	20.57
rs12880641	G	T	0.661581	-0.0138449	0.00188965	2.40E-13	-0.004	0.0151	0.790801	24.04

SNP	effect_allele WC	other_allele WC	eaf. WC	beta. WC	se. WC	pval. WC	beta.ALRI	se. ALRI	pval.ALRI	F-statistics
rs12926311	C	G	0.353624	-0.0124576	0.00187714	3.20E-11	-0.0104	0.0161	0.518	20.13
rs12926311	C	G	0.353624	-0.0124576	0.00187714	3.20E-11	-0.0105	0.2437	0.9656	20.13
rs1296328	C	A	0.559023	-0.0131912	0.00180908	3.10E-13	3.00E-04	0.0147	0.9821	26.22
rs12983532	T	C	0.251127	-0.0149964	0.00209191	7.60E-13	0.0222	0.0175	0.204	19.33
rs13033310	A	G	0.25275	0.0125512	0.00206781	1.30E-09	0.0011	0.0171	0.9494	13.92
rs13047416	G	C	0.37702	-0.0139009	0.00185438	6.60E-14	0.013	0.0147	0.3753	26.40
rs13163306	A	G	0.466047	-9.89E-03	0.00179351	3.50E-08	-0.0091	0.0147	0.535499	15.13
rs13182474	C	G	0.318883	-0.0117225	0.00192056	1.00E-09	0.0121	0.0177	0.4942	16.18
rs1320903	A	G	0.319725	0.017072	0.00191652	5.20E-19	-0.0132	0.0161	0.4133	34.52
rs1321521	A	C	0.345235	0.0141428	0.00187867	5.10E-14	-0.0154	0.0148	0.2969	25.62
rs13264909	T	A	0.428948	-0.0122831	0.00181048	1.20E-11	-0.0261	0.0147	0.0755301	22.55
rs1327259	G	A	0.387739	-0.0115864	0.0018403	3.10E-10	-0.0061	0.0148	0.6811	18.82
rs13288841	A	G	0.32166	0.0190501	0.00191229	2.20E-23	3.00E-04	0.0151	0.9833	43.31
rs13322435	G	A	0.404446	-0.0169561	0.00182964	1.90E-20	0.0035	0.0155	0.8239	41.38
rs13333747	C	T	0.182682	-0.022678	0.00232636	1.90E-22	0.0091	0.0192	0.635501	28.38
rs1336486	G	T	0.328585	0.012489	0.00190819	6.00E-11	-0.0147	0.0149	0.3246	18.90
rs13410783	G	A	0.36952	0.0141058	0.0018498	2.40E-14	-0.0118	0.0151	0.4329	27.10
rs13420048	A	C	0.365011	-0.0133504	0.00185957	7.00E-13	-0.0041	0.0148	0.7795	23.89
rs13427822	G	A	0.271197	-0.0141495	0.00203048	3.20E-12	0.0176	0.016	0.2712	19.20
rs1346841	A	G	0.40501	-0.0106038	0.00182614	6.40E-09	0.026	0.0158	0.0989191	16.25
rs1357079	C	T	0.570076	0.011183	0.00180784	6.20E-10	-0.0041	0.0149	0.7835	18.76
rs1360201	T	C	0.48157	0.0097942	0.00178924	4.40E-08	-0.0073	0.0147	0.6214	14.96
rs1405261	A	T	0.434383	-9.85E-03	0.0018049	4.80E-08	0.0108	0.0147	0.4634	14.64
rs1411432	C	A	0.186234	0.0150036	0.00230632	7.70E-11	0.014	0.0192	0.466	12.83

SNP	effect_allele WC	other_allele WC	eaf. WC	beta. WC	se. WC	pval. WC	beta.ALRI	se. ALRI	pval.ALRI	F-statistics
rs1436348	G	A	0.582801	0.0124929	0.00181185	5.40E-12	0.009	0.0152	0.5517	23.12
rs1441098	T	A	0.54446	-9.91E-03	0.00179682	3.50E-08	-0.0155	0.0148	0.2939	15.08
rs1441264	A	G	0.593703	0.0150095	0.00186203	7.60E-16	0.0071	0.0156	0.6514	31.35
rs1454687	G	C	0.515372	-0.0161615	0.00178705	1.50E-19	-0.0104	0.0148	0.479001	40.86
rs1458156	T	C	0.488413	0.0146036	0.00179105	3.50E-16	-0.0134	0.0147	0.3635	33.23
rs1502317	T	C	0.276578	-0.0173687	0.0020032	4.30E-18	0.0092	0.0166	0.5815	30.09
rs1559900	T	C	0.286025	0.0127734	0.00197882	1.10E-10	0.0151	0.0168	0.3698	17.02
rs156902	T	G	0.267387	-0.0138228	0.00243775	1.40E-08	-0.0305	0.0279	0.2743	12.60
rs1570298	T	A	0.743189	0.0120451	0.00204452	3.80E-09	-4.00E-04	0.0173	0.9818	13.25
rs1582931	A	G	0.473264	-0.0138548	0.00180588	1.70E-14	0.0247	0.0147	0.0920301	29.35
rs1609010	G	A	0.565725	0.0149337	0.00180594	1.30E-16	0.0102	0.0147	0.4872	33.60
rs1609303	A	T	0.631343	0.0152662	0.00185779	2.10E-16	0.0067	0.0153	0.6598	31.44
rs1625623	T	C	0.371988	0.0107407	0.00187307	9.80E-09	0.0015	0.0153	0.9229	15.36
rs165656	C	G	0.517356	0.0103041	0.00180539	1.10E-08	-0.0105	0.0147	0.4771	16.27
rs1657930	A	G	0.802922	-0.0142481	0.00224816	2.30E-10	0.0121	0.0203	0.5507	12.71
rs1711171	C	T	0.749579	0.0175635	0.00206542	1.80E-17	0.0407	0.0204	0.0460098	27.15
rs17296856	C	A	0.280671	-0.0156228	0.00199629	5.00E-15	0.0014	0.0157	0.9301	24.73
rs1731246	T	G	0.757325	-0.0118366	0.0020818	1.30E-08	0.0348	0.0173	0.04348	11.88
rs17446091	C	T	0.20186	0.0149141	0.00222979	2.30E-11	0.0057	0.0209	0.783401	14.42
rs1752169	A	C	0.250696	0.0143082	0.00206705	4.50E-12	0.015	0.0151	0.3219	18.00
rs17681738	T	C	0.328589	0.0105421	0.00190864	3.30E-08	0.0051	0.0157	0.7461	13.46
rs1834144	A	C	0.373194	-0.0146152	0.00185609	3.40E-15	0.0267	0.0147	0.0695793	29.01
rs1861410	T	C	0.555467	-0.016009	0.00180199	6.40E-19	-0.0125	0.0149	0.4036	38.98
rs1902066	C	T	0.562316	0.0109526	0.00181038	1.40E-09	-0.0213	0.0147	0.1471	18.02

SNP	effect_allele WC	other_allele WC	eaf. WC	beta. WC	se. WC	pval. WC	beta.ALRI	se. ALRI	pval.ALRI	F-statistics
rs2020942	T	C	0.394892	0.0108469	0.00183573	3.40E-09	0.0259	0.0148	0.0795994	16.69
rs2074881	T	C	0.168487	-0.0147612	0.00240193	8.00E-10	-0.016	0.0217	0.461	10.58
rs2133561	T	A	0.611063	-0.0122441	0.00185284	3.90E-11	-0.0096	0.0153	0.5309	20.76
rs215669	A	G	0.611654	-0.0125095	0.00184246	1.10E-11	-8.00E-04	0.0163	0.9602	21.90
rs2161097	T	C	0.437804	0.0142773	0.00180004	2.20E-15	0.0059	0.015	0.6962	30.97
rs2172131	C	T	0.578709	-0.0121742	0.00181299	1.90E-11	-0.0094	0.0146	0.5202	21.99
rs2180454	C	T	0.771831	0.0179823	0.00213539	3.70E-17	0.0044	0.0156	0.779399	24.98
rs2183947	A	G	0.225006	-0.0220783	0.00213628	4.90E-25	-0.0285	0.0174	0.102	37.25
rs2225909	C	T	0.774104	0.015797	0.00213642	1.40E-13	-0.0062	0.019	0.743	19.12
rs2253310	G	C	0.626102	0.018205	0.00184715	6.50E-23	-0.0059	0.0148	0.692	45.48
rs2302209	T	C	0.288806	0.0198329	0.00197713	1.10E-23	0.0175	0.0168	0.2955	41.34
rs2306593	T	C	0.488298	-0.0152919	0.00179509	1.60E-17	-0.0227	0.0147	0.1211	36.27
rs2307111	C	T	0.394972	-0.0239722	0.0018299	3.30E-39	-0.043	0.0148	0.003748	82.04
rs2376885	A	G	0.324117	-0.0106223	0.00191252	2.80E-08	-0.0017	0.015	0.9108	13.52
rs2439823	G	A	0.545601	0.0155254	0.00180145	6.80E-18	0.0126	0.0147	0.3925	36.83
rs245767	G	A	0.730365	0.0145976	0.00201635	4.50E-13	0.0462	0.0156	0.00312802	20.64
rs2470549	C	T	0.598143	-0.0119541	0.00182105	5.20E-11	-4.00E-04	0.0151	0.9806	20.72
rs2470946	T	G	0.401445	0.011687	0.00182473	1.50E-10	0.0309	0.0151	0.0402597	19.71
rs2482704	T	G	0.426761	-0.0115642	0.00180618	1.50E-10	0.0202	0.0152	0.1825	20.06
rs2568958	A	G	0.603683	0.0169206	0.00182184	1.60E-20	-0.0053	0.0153	0.7309	41.28
rs2584205	A	G	0.733265	0.0111592	0.00202662	3.70E-08	-0.0151	0.0169	0.3721	11.86
rs2618039	T	A	0.381482	0.0120777	0.00183977	5.20E-11	0.0051	0.0147	0.7296	20.34
rs2678204	G	T	0.34017	0.0157074	0.00188488	7.90E-17	-0.0029	0.0161	0.8567	31.18
rs2696309	C	T	0.720419	0.0112571	0.00199327	1.60E-08	0.0101	0.0156	0.517101	12.85

SNP	effect_allele WC	other_allele WC	eaf. WC	beta. WC	se. WC	pval. WC	beta.ALRI	se. ALRI	pval.ALRI	F-statistics
rs2725371	G	A	0.696098	-0.0154452	0.00195255	2.60E-15	-0.0207	0.0167	0.2156	26.48
rs2744938	G	A	0.147512	0.0319686	0.00251588	5.40E-37	0.0136	0.0183	0.4568	40.61
rs28350	G	A	0.820658	-0.0139376	0.0023374	2.50E-09	-0.0288	0.0188	0.1257	10.47
rs28366156	C	T	0.130582	-0.0187659	0.00265145	1.50E-12	-0.0034	0.0286	0.9061	11.37
rs28375268	T	G	0.645058	-0.0130789	0.00187766	3.30E-12	0.0265	0.0154	0.08501	22.22
rs28489620	A	G	0.29035	-0.0124131	0.00198899	4.40E-10	0.0206	0.016	0.1977	16.05
rs28580375	G	C	0.21874	0.0126069	0.00216777	6.00E-09	0.0031	0.0174	0.8583	11.56
rs2861692	C	T	0.275371	-0.0166517	0.00199772	7.70E-17	0.0018	0.0161	0.9101	27.73
rs2903738	T	A	0.221402	-0.0133061	0.00215663	6.80E-10	6.00E-04	0.0172	0.973	13.12
rs308911	G	A	0.714436	-0.0114749	0.00198055	6.90E-09	0.0117	0.0163	0.4747	13.70
rs3113509	T	C	0.731966	-0.0123292	0.00201975	1.00E-09	-0.0221	0.0163	0.1741	14.62
rs319775	C	T	0.608753	0.0101556	0.0018335	3.00E-08	-0.023	0.015	0.1241	14.61
rs3212038	G	A	0.328517	0.0126546	0.00191072	3.50E-11	0.0326	0.0149	0.0282898	19.35
rs34045288	T	C	0.334413	0.0203746	0.00189485	5.80E-27	0.0041	0.0151	0.7879	51.47
rs34140906	C	T	0.17033	-0.0179391	0.00238486	5.40E-14	0.0037	0.0184	0.8423	15.99
rs34234296	A	G	0.392393	-0.0131214	0.00184833	1.30E-12	0.0018	0.016	0.9103	24.03
rs34483452	A	C	0.136355	0.0270483	0.0026269	7.30E-25	0.0347	0.0225	0.1221	24.97
rs34517439	A	C	0.121789	0.0305491	0.00276165	1.90E-28	0.0069	0.0221	0.754899	26.18
rs347551	G	C	0.472292	0.0126307	0.00181978	3.90E-12	0.0058	0.0148	0.696899	24.01
rs34882821	T	G	0.338577	0.0106237	0.00189644	2.10E-08	0.021	0.0161	0.192	14.06
rs34994596	C	T	0.297289	-0.014559	0.00195866	1.10E-13	-0.0039	0.0166	0.8131	23.09
rs35023999	C	A	0.508297	-0.0113409	0.00178977	2.40E-10	0.0066	0.0147	0.652501	20.07
rs35243581	T	C	0.317318	0.0173643	0.00192251	1.70E-19	-0.0025	0.0158	0.8755	35.35
rs35681682	C	T	0.407729	-0.0101534	0.00177268	1.00E-08	0.0183	0.0147	0.2151	15.85

SNP	effect_allele WC	other_allele WC	eaf. WC	beta. WC	se. WC	pval. WC	beta.ALRI	se. ALRI	pval.ALRI	F-statistics
rs35882248	T	C	0.317213	0.0157088	0.00191956	2.80E-16	8.00E-04	0.0158	0.9591	29.01
rs36061954	T	C	0.39881	0.0114574	0.00182669	3.60E-10	0.0098	0.015	0.5123	18.87
rs36140	C	A	0.635311	0.0111762	0.001867	2.10E-09	0.0123	0.0154	0.4245	16.61
rs36165342	C	T	0.478618	0.0107118	0.00178943	2.10E-09	-0.0094	0.0149	0.5271	17.88
rs3764002	T	C	0.261552	-0.0160772	0.00203505	2.80E-15	-0.0068	0.0155	0.661301	24.11
rs3768321	T	G	0.196525	0.0176726	0.00224849	3.80E-15	0.0218	0.0198	0.2723	19.51
rs3784692	T	C	0.601752	0.0185824	0.00182662	2.60E-24	-0.0181	0.0153	0.236	49.61
rs3806114	A	G	0.668366	-0.0108144	0.00192005	1.80E-08	-0.0084	0.0171	0.6242	14.06
rs3807566	T	G	0.438304	-0.012149	0.00180583	1.70E-11	-0.0024	0.0151	0.873	22.29
rs3814883	T	C	0.482382	0.0239816	0.00179535	1.10E-40	0.0146	0.0148	0.3258	89.12
rs3816760	A	G	0.307437	0.0133538	0.00193927	5.70E-12	-0.0145	0.0167	0.3859	20.19
rs3826408	T	C	0.456788	0.0112249	0.00179579	4.10E-10	-0.0114	0.0147	0.4391	19.39
rs3845344	T	C	0.391147	0.0107371	0.00182845	4.30E-09	0.0152	0.0148	0.3052	16.42
rs3866805	A	C	0.355681	0.010421	0.00187021	2.50E-08	-0.0038	0.0155	0.8053	14.23
rs3935190	A	G	0.536793	-0.0126195	0.00180625	2.80E-12	0.0337	0.0147	0.0218801	24.28
rs3936510	T	G	0.201255	0.0137147	0.0022268	7.30E-10	-0.0095	0.0212	0.6541	12.20
rs3949781	A	T	0.538137	0.0115117	0.00180564	1.80E-10	0.0128	0.0147	0.3825	20.21
rs40067	A	G	0.170153	-0.0156125	0.00238663	6.10E-11	-0.0188	0.018	0.2956	12.09
rs4017425	T	C	0.47017	-0.0101269	0.00179274	1.60E-08	-0.0056	0.0151	0.7126	15.90
rs4072917	A	G	0.474253	0.0117189	0.0017994	7.40E-11	-0.0048	0.0148	0.7473	21.15
rs4075353	A	G	0.344101	-0.0105885	0.00189213	2.20E-08	0	0.0153	0.9978	14.14
rs4290163	T	G	0.392697	0.0113777	0.00183305	5.40E-10	-0.0129	0.015	0.3919	18.38
rs429343	G	A	0.576576	-0.0124509	0.00180931	5.90E-12	-0.0085	0.0147	0.5623	23.12
rs429358	C	T	0.154146	-0.0271422	0.00248126	7.50E-28	-0.0395	0.0191	0.0384698	31.21

SNP	effect_allele WC	other_allele WC	eaf. WC	beta. WC	se. WC	pval. WC	beta.ALRI	se. ALRI	pval.ALRI	F-statistics
rs4419475	T	A	0.407376	0.0112635	0.00181962	6.00E-10	0.0196	0.0147	0.1805	18.50
rs4456769	T	C	0.333423	0.0134001	0.0019003	1.80E-12	0.023	0.0154	0.1351	22.10
rs4469245	T	A	0.662728	-0.0115211	0.00189126	1.10E-09	-0.0026	0.016	0.8715	16.59
rs4525978	T	C	0.734555	-0.0113759	0.00203018	2.10E-08	0.0211	0.0175	0.2277	12.24
rs4527444	G	A	0.541317	0.0105243	0.00179433	4.50E-09	-0.0175	0.0146	0.2324	17.08
rs4552632	A	G	0.616568	-0.0101695	0.00184128	3.30E-08	0.0032	0.0155	0.8381	14.42
rs4689465	C	T	0.525445	-0.0108689	0.00178976	1.30E-09	0.0166	0.0147	0.2572	18.39
rs4706004	G	A	0.217047	-0.0134593	0.00216831	5.40E-10	-0.0062	0.0166	0.710901	13.10
rs4718964	T	G	0.413175	0.0122483	0.00182202	1.80E-11	-0.01	0.015	0.5021	21.91
rs4742782	G	C	0.315942	0.0124912	0.00192172	8.00E-11	0.0252	0.015	0.0943105	18.26
rs484455	A	G	0.481363	-0.0115336	0.00179638	1.40E-10	0.0138	0.0151	0.3609	20.58
rs4851283	G	C	0.684805	-0.0173961	0.00193525	2.50E-19	0.0073	0.0178	0.681201	34.88
rs4856720	C	G	0.53938	0.0114342	0.0017918	1.80E-10	-0.0112	0.0147	0.4472	20.24
rs4876611	G	A	0.720239	0.0150085	0.0019947	5.30E-14	-0.0052	0.0158	0.7424	22.82
rs4900715	A	G	0.507101	-0.0114279	0.00179331	1.90E-10	-0.0164	0.0149	0.272	20.30
rs4908672	T	C	0.393063	0.0113966	0.00183009	4.70E-10	-0.0148	0.0148	0.3155	18.50
rs520478	T	G	0.701359	-0.0125051	0.00197262	2.30E-10	0.0034	0.0174	0.8465	16.84
rs539515	C	A	0.204929	0.0378223	0.00221181	1.50E-65	-0.0092	0.0191	0.632	95.31
rs55726687	A	G	0.209735	0.0199369	0.00219529	1.10E-19	0.0072	0.0179	0.6863	27.34
rs557951	G	T	0.312953	0.0120789	0.00193163	4.00E-10	-0.013	0.0166	0.435	16.82
rs559231	T	G	0.393054	0.0107518	0.00184107	5.20E-09	0.0042	0.0148	0.7758	16.27
rs56094641	G	A	0.404591	0.057552	0.00182234	1.00E-200	0.0211	0.0148	0.1549	481.03
rs56803094	G	A	0.226708	-0.0127745	0.00214599	2.60E-09	1.00E-04	0.0164	0.9934	12.42

SNP	effect_allele WC	other_allele WC	eaf. WC	beta. WC	se. WC	pval. WC	beta.ALRI	se. ALRI	pval.ALRI	F-statistics
rs57636386	C	T	0.083822	-0.0309067	0.00324088	1.50E-21	-0.0277	0.0336	0.4106	13.97
rs587271	T	C	0.686869	0.0118054	0.0020079	4.10E-09	0.0018	0.0159	0.9102	14.87
rs58862095	T	C	0.419271	-0.0165812	0.00181678	7.10E-20	-0.0013	0.0147	0.9319	40.57
rs588660	A	G	0.584119	0.0155276	0.00181174	1.00E-17	0.0046	0.0149	0.756401	35.69
rs59068084	T	G	0.410212	0.0101403	0.00181938	2.50E-08	0.0372	0.015	0.01339	15.03
rs59104534	T	C	0.298501	0.0107189	0.00196197	4.70E-08	-0.0074	0.0156	0.6368	12.50
rs6001877	A	G	0.339906	-0.010579	0.00189657	2.40E-08	-0.0068	0.0151	0.6519	13.96
rs6069037	A	C	0.731389	-0.011125	0.00202001	3.60E-08	9.00E-04	0.0171	0.9594	11.92
rs61223906	A	G	0.339313	-0.0108726	0.00188655	8.30E-09	0.0196	0.0152	0.1972	14.89
rs61813324	T	C	0.135713	0.0219762	0.00264443	9.50E-17	-0.0052	0.0203	0.7981	16.20
rs61903695	G	A	0.254942	0.0134514	0.00205497	5.90E-11	0.0104	0.0171	0.5408	16.28
rs61969511	A	G	0.278938	0.011857	0.0020127	3.80E-09	0.012	0.0152	0.4309	13.96
rs61992671	G	A	0.491969	-0.0130005	0.00187164	3.80E-12	0.0017	0.0149	0.91	24.12
rs62243489	G	T	0.259239	-0.0155648	0.00204876	3.00E-14	0.0015	0.0161	0.9281	22.17
rs62261725	G	A	0.326101	-0.0148698	0.00190872	6.70E-15	-0.0575	0.0152	0.000163599	26.68
rs6493498	C	T	0.54539	-0.013082	0.001805	4.20E-13	-0.026	0.0149	0.0816601	26.05
rs649458	A	T	0.860102	-0.018179	0.00256951	1.50E-12	0.0193	0.023	0.4023	12.05
rs6536575	C	T	0.51907	0.0108432	0.00178995	1.40E-09	-0.0087	0.0148	0.5569	18.32
rs6551304	G	A	0.831916	0.016827	0.00239416	2.10E-12	-0.0096	0.0212	0.6487	13.82
rs6567160	C	T	0.232725	0.0451644	0.00211824	7.19E-101	0.0065	0.0189	0.728501	162.41
rs6575340	A	G	0.636022	0.0161584	0.00186502	4.60E-18	0.0408	0.0152	0.00704206	34.76
rs6669341	G	A	0.58271	-0.0125214	0.0018097	4.50E-12	-0.0092	0.0151	0.5424	23.28
rs6682438	C	T	0.673097	0.0124771	0.00190203	5.40E-11	-0.0011	0.0155	0.9449	18.94

SNP	effect_allele WC	other_allele WC	eaf. WC	beta. WC	se. WC	pval. WC	beta.ALRI	se. ALRI	pval.ALRI	F-statistics
rs6693294	G	A	0.688535	-0.0170126	0.00192843	1.10E-18	0.0259	0.0151	0.0870001	33.38
rs6739755	G	A	0.603358	-0.0159241	0.00182885	3.10E-18	0.0087	0.0149	0.5596	36.29
rs67609008	C	T	0.283612	0.011102	0.00199233	2.50E-08	-0.005	0.0191	0.793999	12.62
rs6791983	A	C	0.750006	0.0123148	0.00205988	2.30E-09	-0.0134	0.0174	0.441	13.40
rs6799080	A	G	0.353391	0.0102307	0.00186721	4.30E-08	-0.0103	0.0148	0.4845	13.72
rs6846041	G	C	0.320508	0.0123073	0.00191467	1.30E-10	-0.0173	0.0168	0.3022	18.00
rs6849518	T	C	0.12428	0.0218636	0.00270973	7.10E-16	-0.0152	0.0196	0.4388	14.17
rs6938973	C	T	0.601488	0.0120106	0.001827	4.90E-11	0.0147	0.0157	0.3482	20.72
rs7034554	G	A	0.373813	-0.0112644	0.00184813	1.10E-09	-0.0174	0.0161	0.2806	17.39
rs703984	C	G	0.414809	-0.011472	0.00181967	2.90E-10	0.0097	0.0151	0.5196	19.30
rs704061	C	T	0.455057	0.0146223	0.00179659	4.00E-16	0.0242	0.0151	0.1082	32.86
rs7070670	T	C	0.327941	-0.0120318	0.00191737	3.50E-10	-0.0032	0.0163	0.8448	17.36
rs7115013	T	C	0.442738	-0.0106394	0.00180461	3.70E-09	0.009	0.0149	0.5455	17.15
rs7132908	A	G	0.384454	0.0215129	0.00183998	1.40E-31	-0.0243	0.0151	0.1073	64.71
rs7169847	T	G	0.635678	-0.0102703	0.00186771	3.80E-08	0.0203	0.0147	0.1671	14.01
rs7171864	A	G	0.660202	0.0128894	0.00189898	1.10E-11	0.0069	0.0161	0.6665	20.67
rs7206608	G	C	0.321623	0.0120004	0.00191641	3.80E-10	0.0133	0.0153	0.3852	17.11
rs7218014	C	T	0.197315	0.0224089	0.00225393	2.70E-23	0.0341	0.0172	0.0469797	31.31
rs7259070	C	T	0.596078	0.0153811	0.00184133	6.60E-17	-0.0015	0.0148	0.9191	33.60
rs72634826	A	G	0.259865	-0.0148957	0.00206456	5.40E-13	-0.0022	0.0181	0.9037	20.02
rs72892910	T	G	0.172259	0.0301404	0.00237148	5.20E-37	0.0035	0.0182	0.8492	46.07
rs72976986	A	G	0.190131	-0.0157587	0.00230334	7.80E-12	0.0149	0.0202	0.4605	14.42
rs73052033	C	T	0.184916	-0.0210096	0.00230513	7.90E-20	0.0157	0.0199	0.4303	25.04
rs73068448	T	C	0.170691	-0.0153436	0.00241309	2.00E-10	0.0097	0.022	0.6586	11.45

SNP	effect_allele WC	other_allele WC	eaf. WC	beta. WC	se. WC	pval. WC	beta.ALRI	se. ALRI	pval.ALRI	F-statistics
rs73142879	T	C	0.192309	-0.0241942	0.00228107	2.80E-26	-0.0125	0.0201	0.5328	34.95
rs7324067	C	T	0.761254	0.0121297	0.00210064	7.70E-09	-0.0062	0.0165	0.7085	12.12
rs735033	G	A	0.605139	-0.0102658	0.00184312	2.50E-08	-0.0318	0.0147	0.0311	14.83
rs7372674	A	C	0.357239	0.0118947	0.00186339	1.70E-10	-0.0017	0.016	0.9167	18.71
rs7377083	A	C	0.431061	0.0143396	0.00181913	3.20E-15	0.0037	0.0148	0.8023	30.48
rs73985439	C	A	0.307294	0.0124046	0.00193905	1.60E-10	0.018	0.0159	0.2571	17.42
rs7442885	G	C	0.214033	-0.0204724	0.00218339	6.80E-21	4.00E-04	0.0188	0.9815	29.58
rs7498044	A	G	0.217312	-0.0151498	0.00219298	4.90E-12	0.0127	0.0169	0.4536	16.24
rs7498665	G	A	0.399688	0.026616	0.00182769	4.90E-48	0.0102	0.0149	0.494399	101.79
rs7519259	A	G	0.528392	0.0126703	0.00179634	1.70E-12	0.019	0.0148	0.1993	24.80
rs7537581	A	C	0.531758	0.0107362	0.00180248	2.60E-09	-0.0125	0.0148	0.3971	17.67
rs7539903	A	T	0.615583	-0.0107184	0.00183572	5.30E-09	0.0199	0.0149	0.183	16.14
rs756717	A	G	0.399071	-0.0107152	0.00184896	6.80E-09	0.0161	0.015	0.2824	16.11
rs76286777	C	T	0.217783	0.0234759	0.0021623	1.80E-27	0.0319	0.0214	0.136	40.16
rs7630382	T	C	0.531552	0.013362	0.00179336	9.30E-14	4.00E-04	0.0147	0.98	27.65
rs765876	G	A	0.489495	-9.81E-03	0.00178785	4.10E-08	-0.0172	0.0149	0.2488	15.04
rs7707394	A	G	0.357264	-0.0167827	0.00186285	2.10E-19	-0.0314	0.0151	0.0382596	37.28
rs7708584	G	A	0.572375	-0.0121976	0.00180534	1.40E-11	0.0102	0.0149	0.4965	22.35
rs77165542	T	C	0.035495	-0.0700663	0.00488088	9.90E-47	0.1045	0.0564	0.0642007	14.11
rs7752202	T	C	0.145139	0.0177008	0.00253183	2.70E-12	0.0074	0.0239	0.755699	12.13
rs784257	C	T	0.812569	0.0161785	0.00230615	2.30E-12	-7.00E-04	0.0196	0.9713	14.99
rs7845090	A	G	0.709022	-0.0195133	0.00197942	6.30E-23	0.0128	0.0166	0.4413	40.10
rs7925100	A	G	0.396115	0.0140407	0.00182953	1.70E-14	-0.0251	0.0161	0.12	28.18
rs7952436	T	C	0.081988	-0.0289945	0.00326001	5.90E-19	-0.0092	0.0332	0.7807	11.91

SNP	effect_allele WC	other_allele WC	eaf. WC	beta. WC	se. WC	pval. WC	beta.ALRI	se. ALRI	pval.ALRI	F-statistics
rs7966251	A	G	0.255065	-0.0115986	0.00205522	1.70E-08	0.0145	0.0163	0.3748	12.10
rs8013377	C	A	0.269606	-0.0166104	0.002018	1.90E-16	-0.0244	0.0159	0.1254	26.68
rs80243702	A	G	0.160621	0.0151663	0.00245648	6.70E-10	-8.00E-04	0.0249	0.9731	10.28
rs8078135	T	C	0.489933	-0.0101458	0.00179655	1.60E-08	0.0059	0.0146	0.686901	15.94
rs8097672	T	A	0.145231	0.0167802	0.00255407	5.00E-11	0.032	0.0193	0.0980393	10.72
rs815163	C	T	0.563195	-0.0131383	0.00179812	2.70E-13	-0.0125	0.0147	0.3929	26.27
rs8192675	C	T	0.288645	0.0159923	0.00196858	4.50E-16	-0.0055	0.0166	0.7408	27.10
rs852042	G	A	0.758579	-0.0115951	0.00209164	3.00E-08	0.0211	0.0173	0.2214	11.26
rs852983	A	G	0.459562	-9.83E-03	0.00179325	4.20E-08	-0.0167	0.0146	0.2553	14.93
rs862227	G	A	0.457951	-0.0109798	0.00179141	8.80E-10	-0.0068	0.0148	0.646501	18.65
rs862320	T	C	0.409625	-0.0180264	0.0018218	4.40E-23	-0.0195	0.0148	0.1868	47.36
rs876605	G	A	0.739809	-0.0111634	0.00203598	4.20E-08	0.0116	0.0168	0.4883	11.57
rs879620	T	C	0.613194	0.0194431	0.00184223	4.90E-26	-0.0237	0.0149	0.1117	52.85
rs883403	C	T	0.154427	-0.0176676	0.00247511	9.50E-13	8.00E-04	0.0176	0.9632	13.31
rs894736	G	A	0.362765	0.0157371	0.00186806	3.60E-17	0.0065	0.0147	0.6578	32.81
rs923994	G	A	0.783206	-0.0130094	0.0021745	2.20E-09	-0.0051	0.0176	0.7724	12.16
rs9289630	C	G	0.389039	0.0144758	0.00183898	3.50E-15	-0.0021	0.0158	0.8918	29.46
rs9294260	A	G	0.476584	0.0131781	0.00179925	2.40E-13	0.0175	0.0147	0.234	26.76
rs9316661	C	T	0.801292	-0.0156101	0.00224739	3.80E-12	-0.0125	0.0185	0.4982	15.36
rs9378676	C	A	0.234005	0.0130316	0.00211353	7.00E-10	0.0048	0.016	0.7615	13.63
rs945211	C	G	0.615598	0.0100787	0.0018363	4.10E-08	-0.01	0.0153	0.5117	14.26
rs9568867	A	G	0.129253	0.022614	0.00268971	4.20E-17	0.0208	0.0212	0.3252	15.91
rs9584870	C	T	0.366117	-0.0108139	0.00189234	1.10E-08	8.00E-04	0.0156	0.9613	15.16
rs9673839	G	A	0.490971	0.0109063	0.00179863	1.30E-09	-0.0217	0.0147	0.1403	18.38

SNP	effect_allele WC	other_allele WC	eaf. WC	beta. WC	se. WC	pval. WC	beta.ALRI	se. ALRI	pval.ALRI	F-statistics
rs9814758	G	T	0.355886	-0.0111552	0.00187521	2.70E-09	5.00E-04	0.0157	0.9726	16.22
rs9835772	T	A	0.243635	0.0121296	0.00208107	5.60E-09	0.0347	0.0174	0.04596	12.52
rs9843653	C	T	0.511657	0.0195793	0.00178819	6.70E-28	0.0221	0.015	0.1396	59.92
rs9888533	T	C	0.538081	0.0108325	0.00182556	3.00E-09	-0.0268	0.0149	0.0728199	17.50
rs9902846	T	C	0.316036	0.0133756	0.00192994	4.20E-12	-0.0045	0.0159	0.7784	20.77
rs9916444	G	C	0.34174	0.0114738	0.0018902	1.30E-09	0.0039	0.0161	0.8097	16.58

Abbreviations: SNP, single nucleotide polymorphism; se, standard error; pval, p-value; WC, waist circumference; ALRI, acute lower respiratory infections.

Table S21. Detailed information about single-nucleotide polymorphisms of cholecystitis on waist circumference.

SNP	effect_allele cholecystitis	other_allele cholecystitis	eaf. cholecystitis	beta. cholecystitis	se. cholecystitis	pval. cholecystitis	beta.WC	se. WC	pval.WC	F-statistics
rs11239536	A	T	0.2814	0.0907	0.013	3.63E-12	-0.0011518	0.00209343	0.58	19.68814933
rs11887534	C	G	0.08435	0.8069	0.0222	1.00E-200	-0.00747256	0.00359654	0.0379997	204.2614375
rs12672720	G	A	0.1351	-0.2014	0.0175	1.08E-30	-0.00101795	0.00257479	0.69	30.95655021
rs16961277	G	A	0.1383	-0.1175	0.0171	5.53E-12	-0.003813	0.00265959	0.15	11.25409227
rs174565	G	C	0.232	0.0849	0.0139	1.15E-09	0.00376739	0.00267414	0.16	13.29498319
rs1800961	T	C	0.04539	0.3663	0.0289	8.46E-37	0.00918135	0.00516955	0.0759994	13.92253727
rs2290846	A	G	0.2213	0.1205	0.0141	1.49E-17	0.000714575	0.00197645	0.719999	25.17469726
rs2618566	T	G	0.6772	0.0747	0.0127	4.08E-09	-8.96E-05	0.0018897	0.96	15.12657984
rs28473566	A	G	0.1731	-0.2365	0.0159	4.46E-50	0.00429294	0.00241517	0.0749998	63.35370188
rs296391	T	C	0.8484	0.1479	0.0165	3.87E-19	0.00559838	0.00242327	0.021	20.66978801
rs4681502	C	T	0.4576	0.0701	0.0118	3.00E-09	0.000333618	0.0018215	0.85	17.52019998
rs4805129	C	T	0.6779	-0.0689	0.0126	4.65E-08	0.00362851	0.00185147	0.05	13.05888337
rs4814175	T	A	0.6579	-0.0675	0.0124	4.78E-08	-0.00311528	0.00183641	0.0899995	13.33920764
rs635634	T	C	0.2001	0.0876	0.0147	2.50E-09	0.00209482	0.00231125	0.36	11.36855443
rs6712163	T	C	0.5453	0.08	0.0119	1.50E-11	0.00107642	0.0018077	0.55	22.41391534
rs686030	A	C	0.8814	0.1498	0.0183	2.45E-16	-0.00428313	0.00257097	0.0959997	14.00984834
rs708686	T	C	0.334	0.1004	0.0125	8.45E-16	-0.00250742	0.00202395	0.22	28.70463466
rs714583	A	T	0.296	-0.1067	0.0129	1.72E-16	-8.60E-06	0.00216541	1.00	28.51657328
rs75399305	C	T	0.09643	0.1585	0.02	2.49E-15	0.00035366	0.00360467	0.92	10.94512117
rs77070100	C	T	0.4514	0.085	0.0118	6.58E-13	0.00420561	0.00190205	0.0269998	25.70212666
rs7979473	G	A	0.5811	0.0778	0.012	7.99E-11	-0.000559793	0.00185123	0.760001	20.46563513
rs8109951	T	C	0.299	0.0802	0.0128	4.24E-10	0.00295756	0.00186111	0.11	16.45800536

SNP	effect_allele cholecystitis	other_allele cholecystitis	eaf. cholecystitis	beta. cholecystitis	se. cholecystitis	pval. cholecystitis	beta.WC	se. WC	pval.WC	F-statistics
rs9297994	A	G	0.6226	-0.1277	0.0121	6.44E-26	-0.00250067	0.00189587	0.19	52.35446811
rs9843304	T	C	0.4925	-0.1228	0.0118	1.66E-25	0.00022389	0.00179835	0.9	54.15147801

Abbreviations: SNP, single nucleotide polymorphism; se, standard error; pval, p-value; WC, waist circumference.

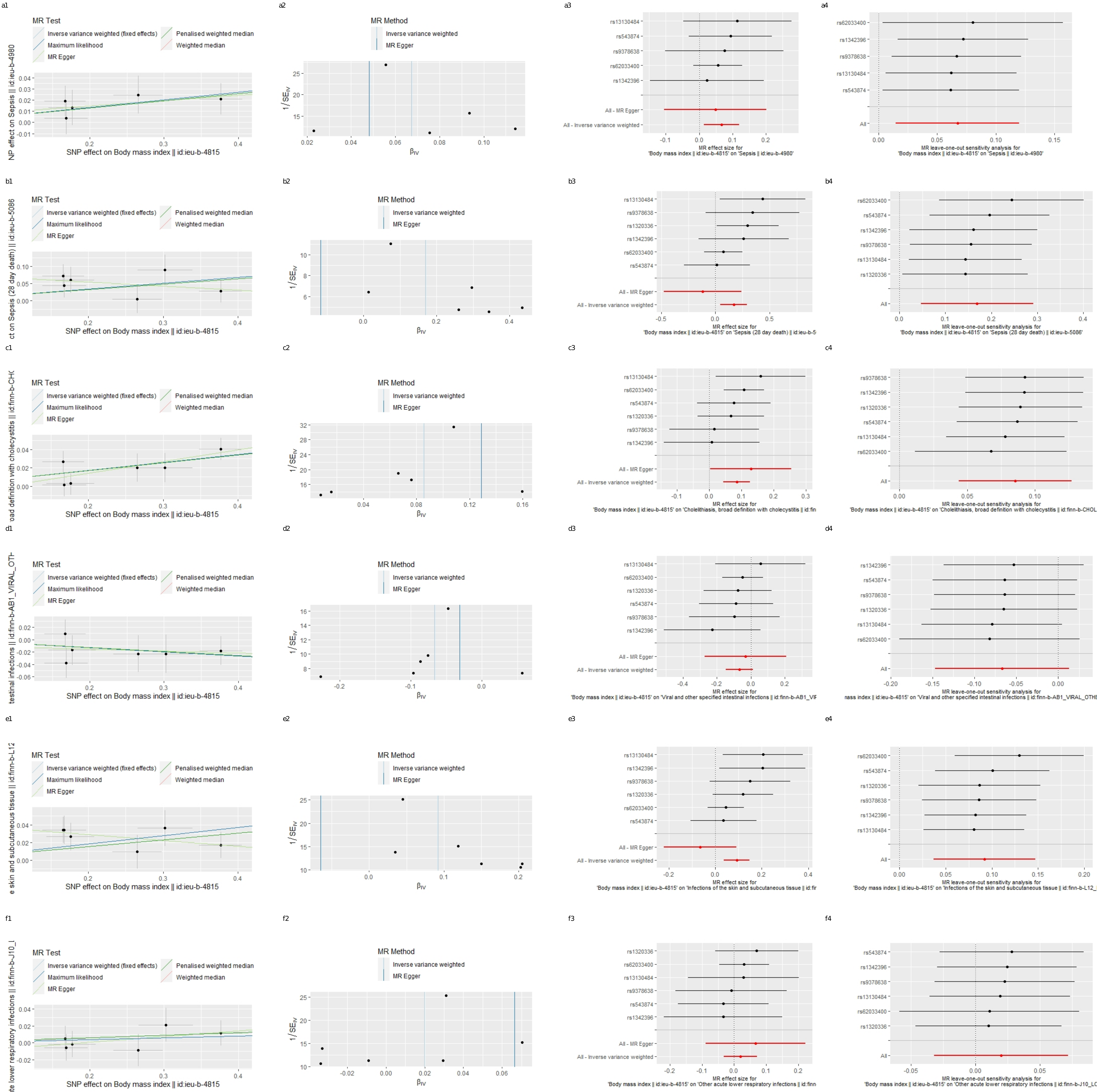

a1
MR Test
Inverse variance weighted (fixed effects)
Maximum likelihood
MR Egger
Penalised weighted median
Weighted median
SNP effect on Body mass index || id:ieu-b-4815
a2
MR Method
Inverse variance weighted
MR Egger
$1/SE_{IV}$
$β_{IV}$
a3
rs13130484
rs543874
rs9378638
rs62033400
rs1342396
All - MR Egger
All - Inverse variance weighted
MR effect size for
'Body mass index || id:ieu-b-4815' on 'Sepsis || id:ieu-b-4980'
a4
rs62033400
rs1342396
rs9378638
rs13130484
rs543874
All
MR leave-one-out sensitivity analysis for
'Body mass index || id:ieu-b-4815' on 'Sepsis || id:ieu-b-4980'
b1
MR Test
SNP effect on Body mass index || id:ieu-b-4815
b2
MR Method
b3
rs13130484
rs9378638
rs1320336
rs1342396
rs62033400
rs543874
All - MR Egger
All - Inverse variance weighted
MR effect size for
b4
MR leave-one-out sensitivity analysis for
'Body mass index || id:ieu-b-4815' on 'Sepsis (28 day death) || id:ieu-b-5086'
c1
MR Test
SNP effect on Body mass index || id:ieu-b-4815
c2
MR Method
c3
MR effect size for
c4
MR leave-one-out sensitivity analysis for
d1
MR Test
SNP effect on Body mass index || id:ieu-b-4815
d2
MR Method
d3
MR effect size for
d4
MR leave-one-out sensitivity analysis for
e1
MR Test
SNP effect on Body mass index || id:ieu-b-4815
e2
MR Method
e3
MR effect size for
e4
MR leave-one-out sensitivity analysis for
'Body mass index || id:ieu-b-4815' on 'Infections of the skin and subcutaneous tissue || id:finn-b-L12_
f1
MR Test
SNP effect on Body mass index || id:ieu-b-4815
f2
MR Method
f3
MR effect size for
f4
MR leave-one-out sensitivity analysis for

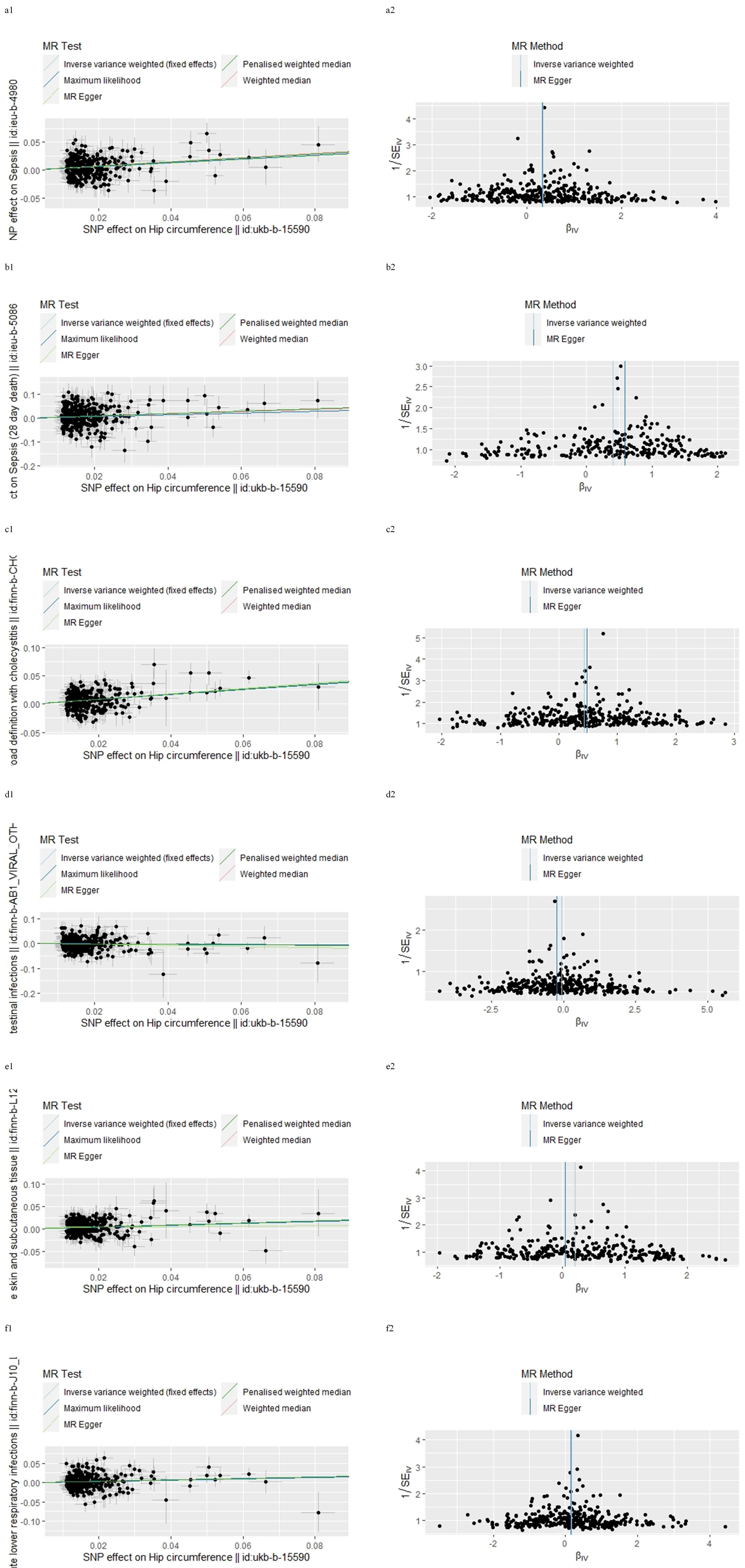

a1
MR Test
Inverse variance weighted (fixed effects)
Maximum likelihood
MR Egger
Penalised weighted median
Weighted median
NP effect on Sepsis || id:ieu-b-4980
SNP effect on Hip circumference || id:ukb-b-15590
a2
MR Method
Inverse variance weighted
MR Egger
1/SE_IV
β_IV
b1
MR Test
Inverse variance weighted (fixed effects)
Maximum likelihood
MR Egger
Penalised weighted median
Weighted median
ct on Sepsis (28 day death) || id:ieu-b-5086
SNP effect on Hip circumference || id:ukb-b-15590
b2
MR Method
Inverse variance weighted
MR Egger
1/SE_IV
β_IV
c1
MR Test
Inverse variance weighted (fixed effects)
Maximum likelihood
MR Egger
Penalised weighted median
Weighted median
oad definition with cholecystitis || id:finn-b-CH
SNP effect on Hip circumference || id:ukb-b-15590
c2
MR Method
Inverse variance weighted
MR Egger
1/SE_IV
β_IV
d1
MR Test
Inverse variance weighted (fixed effects)
Maximum likelihood
MR Egger
Penalised weighted median
Weighted median
testinal infections || id:finn-b-AB1_VIRAL_OTH
SNP effect on Hip circumference || id:ukb-b-15590
d2
MR Method
Inverse variance weighted
MR Egger
1/SE_IV
β_IV
e1
MR Test
Inverse variance weighted (fixed effects)
Maximum likelihood
MR Egger
Penalised weighted median
Weighted median
e skin and subcutaneous tissue || id:finn-b-L12
SNP effect on Hip circumference || id:ukb-b-15590
e2
MR Method
Inverse variance weighted
MR Egger
1/SE_IV
β_IV
f1
MR Test
Inverse variance weighted (fixed effects)
Maximum likelihood
MR Egger
Penalised weighted median
Weighted median
te lower respiratory infections || id:finn-b-J10_
SNP effect on Hip circumference || id:ukb-b-15590
f2
MR Method
Inverse variance weighted
MR Egger
1/SE_IV
β_IV

a1

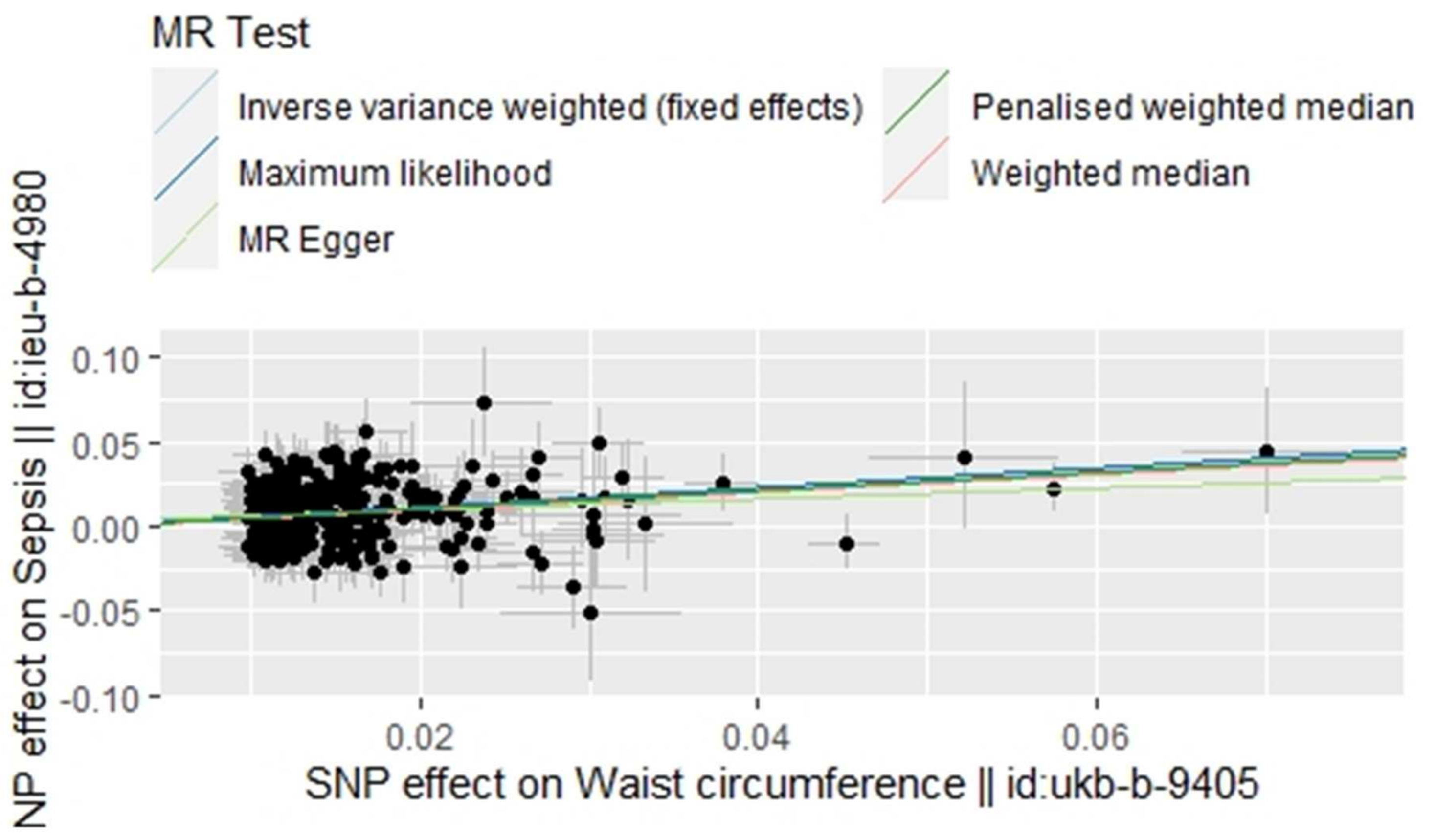

a2

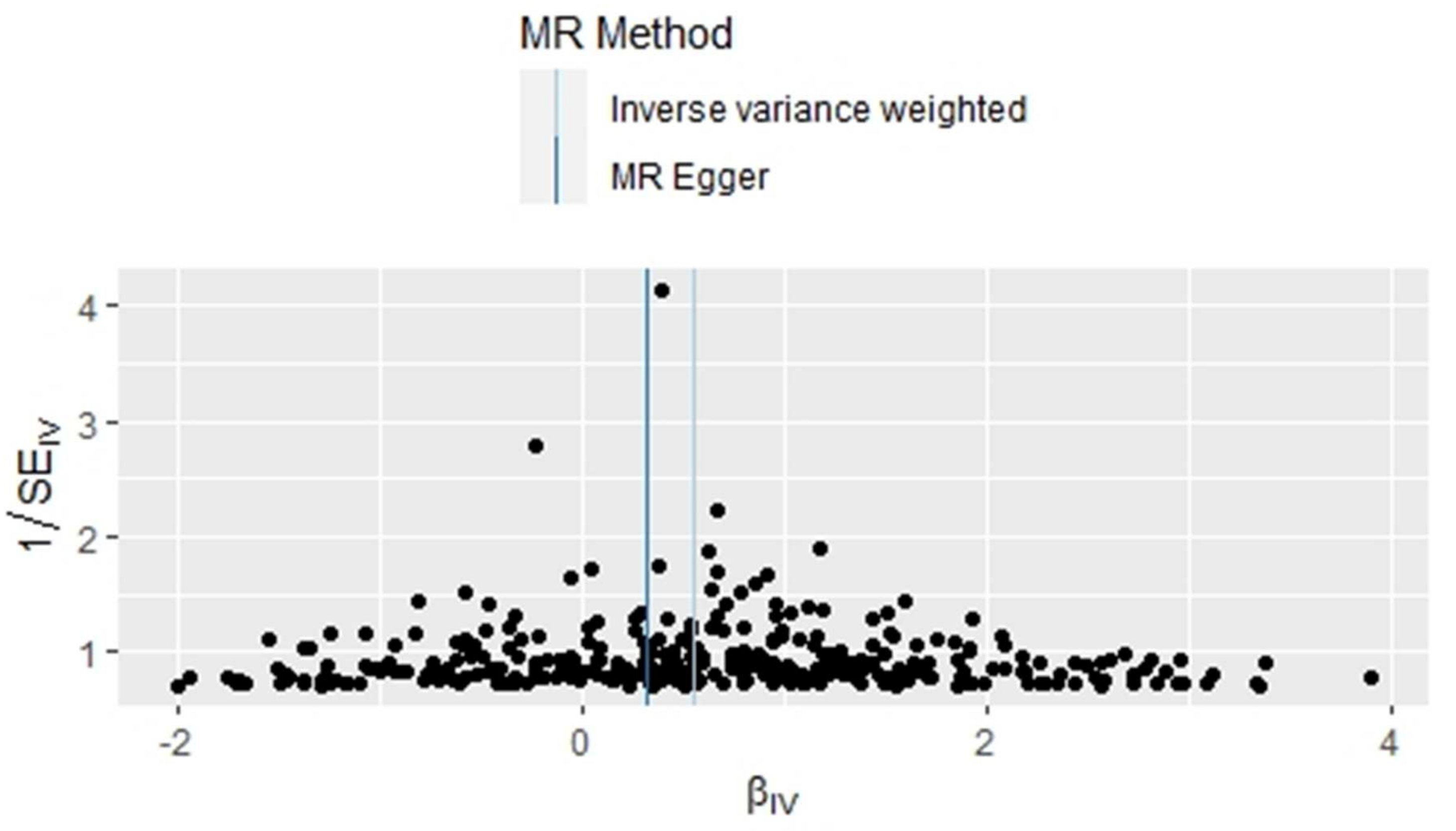

b1

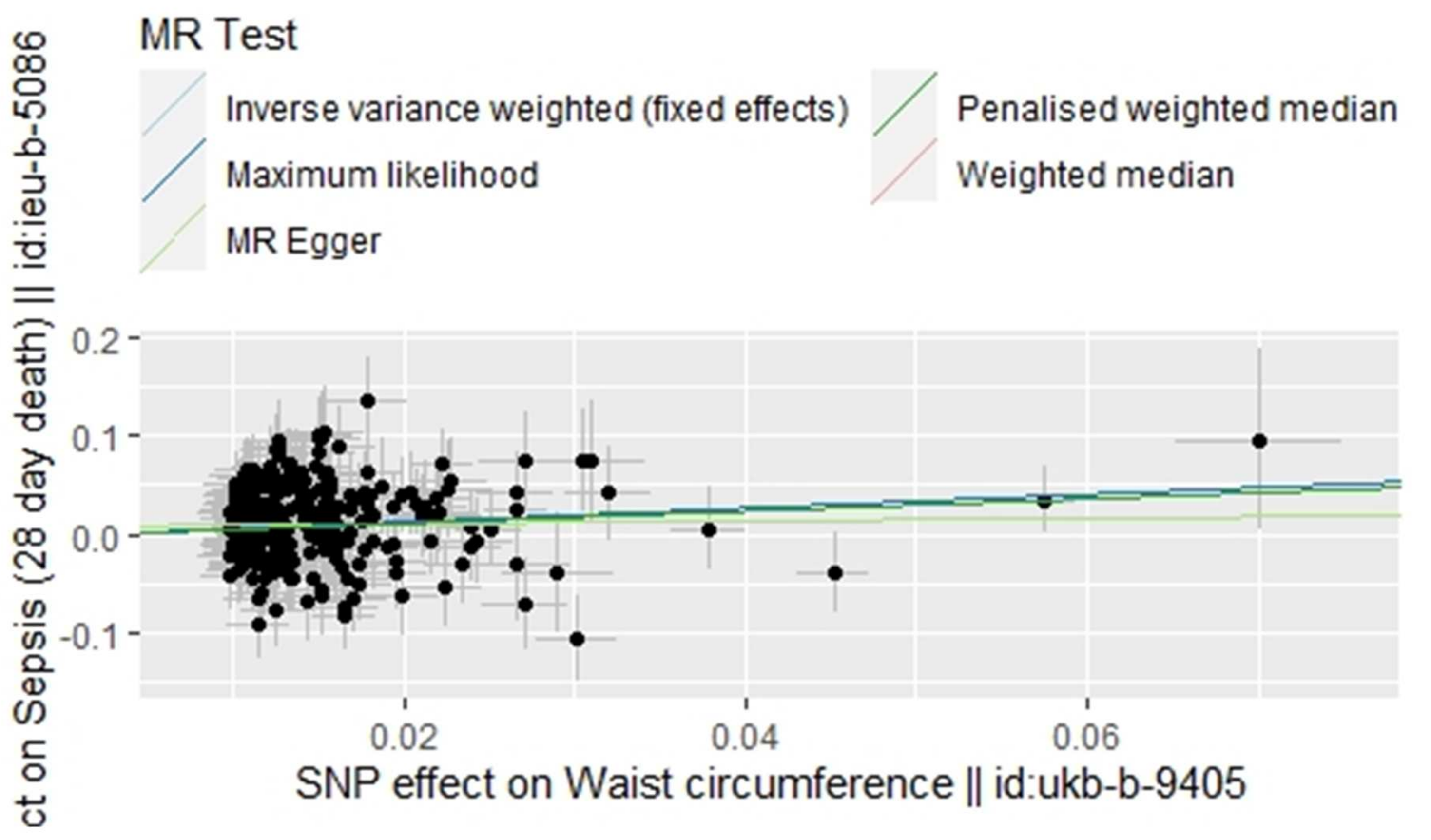

b2

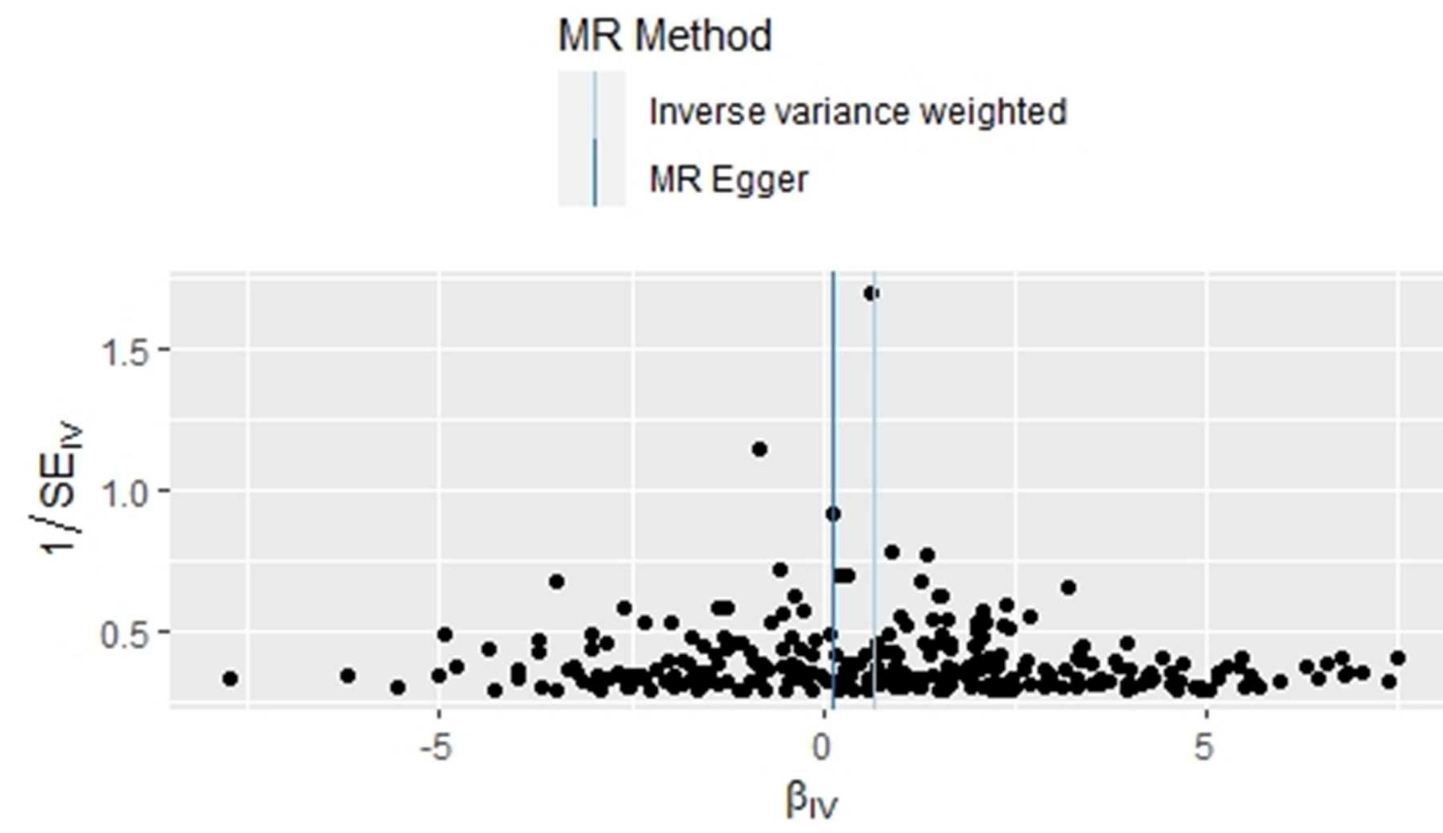

c1

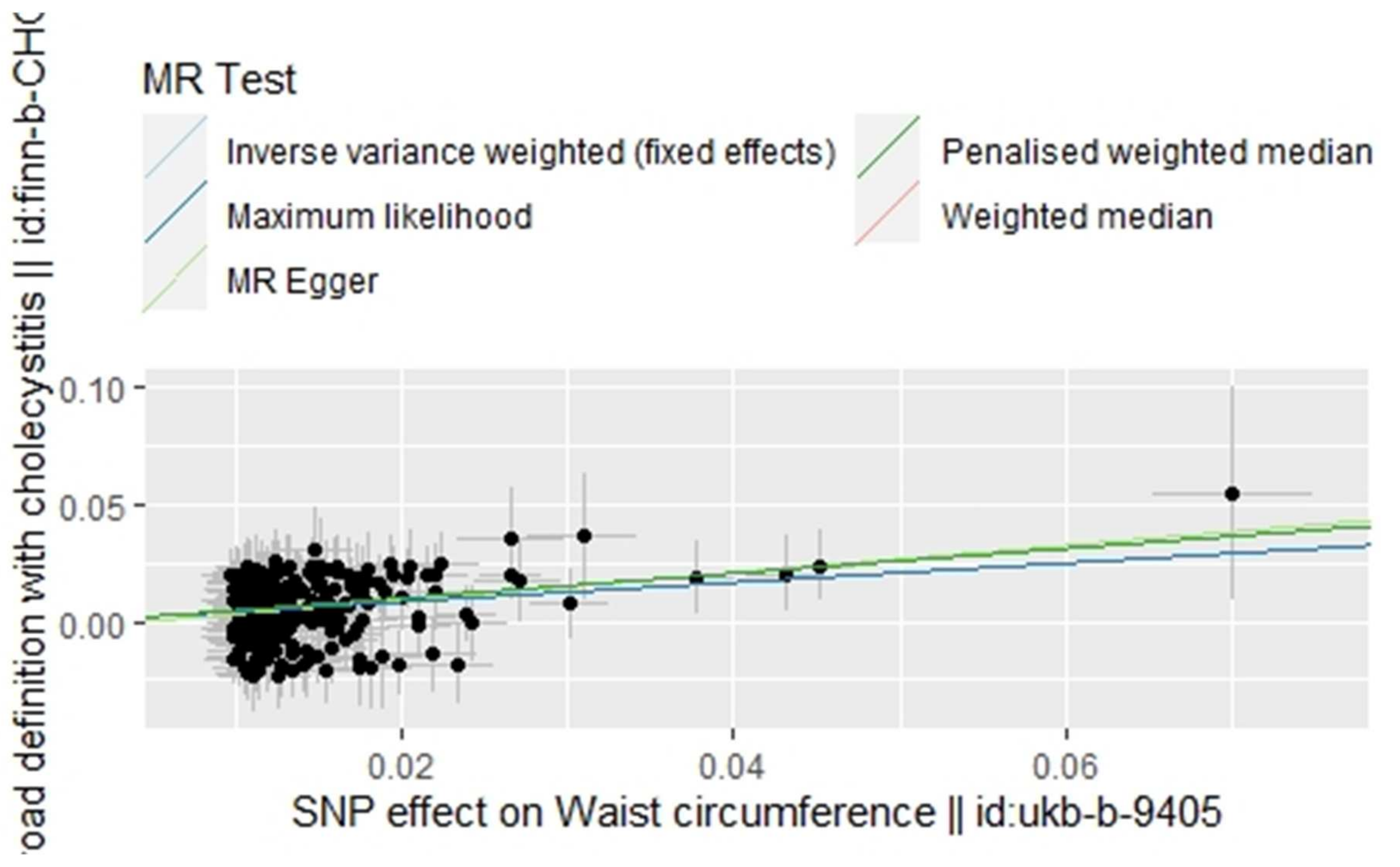

c2

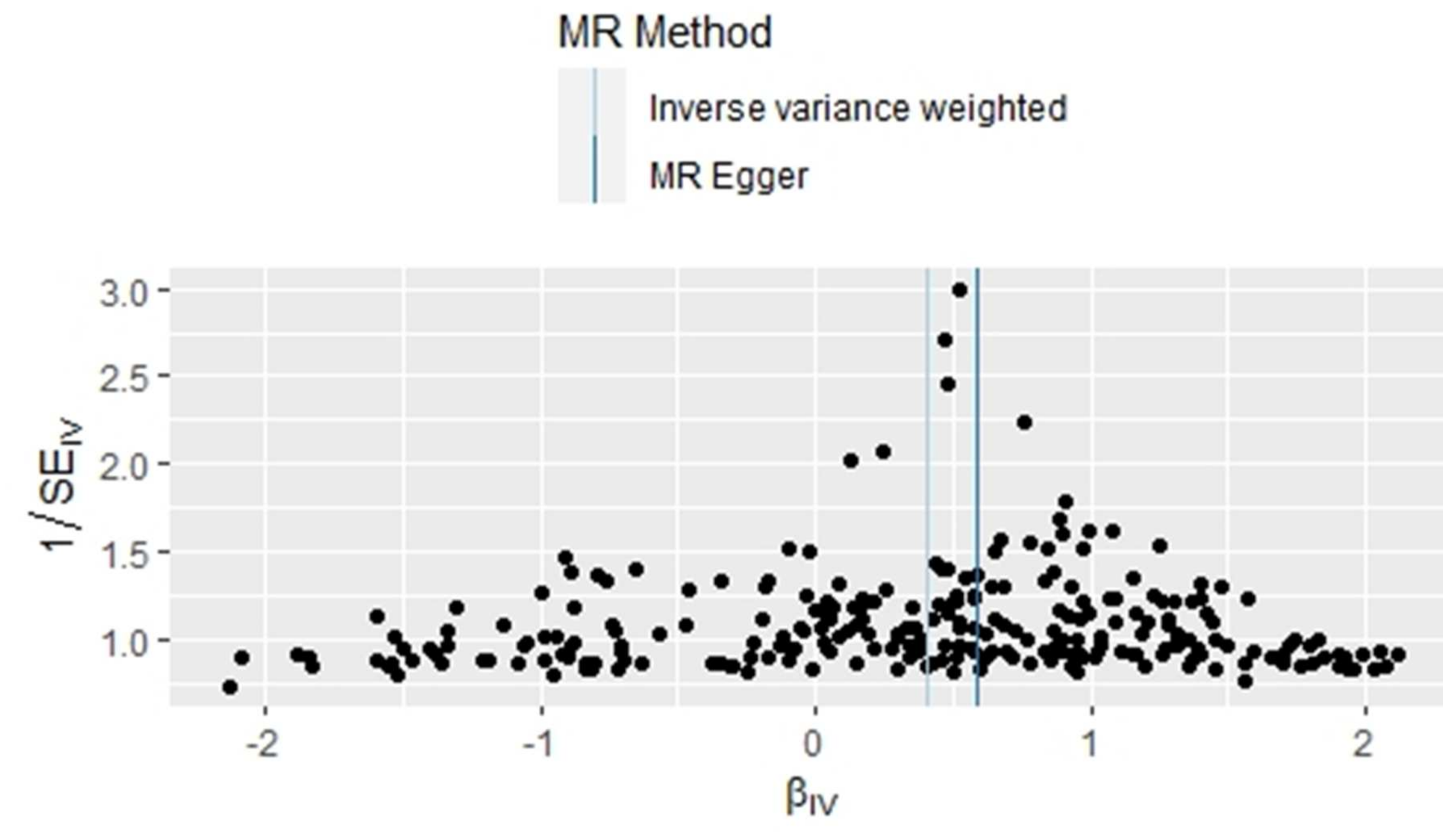

d1

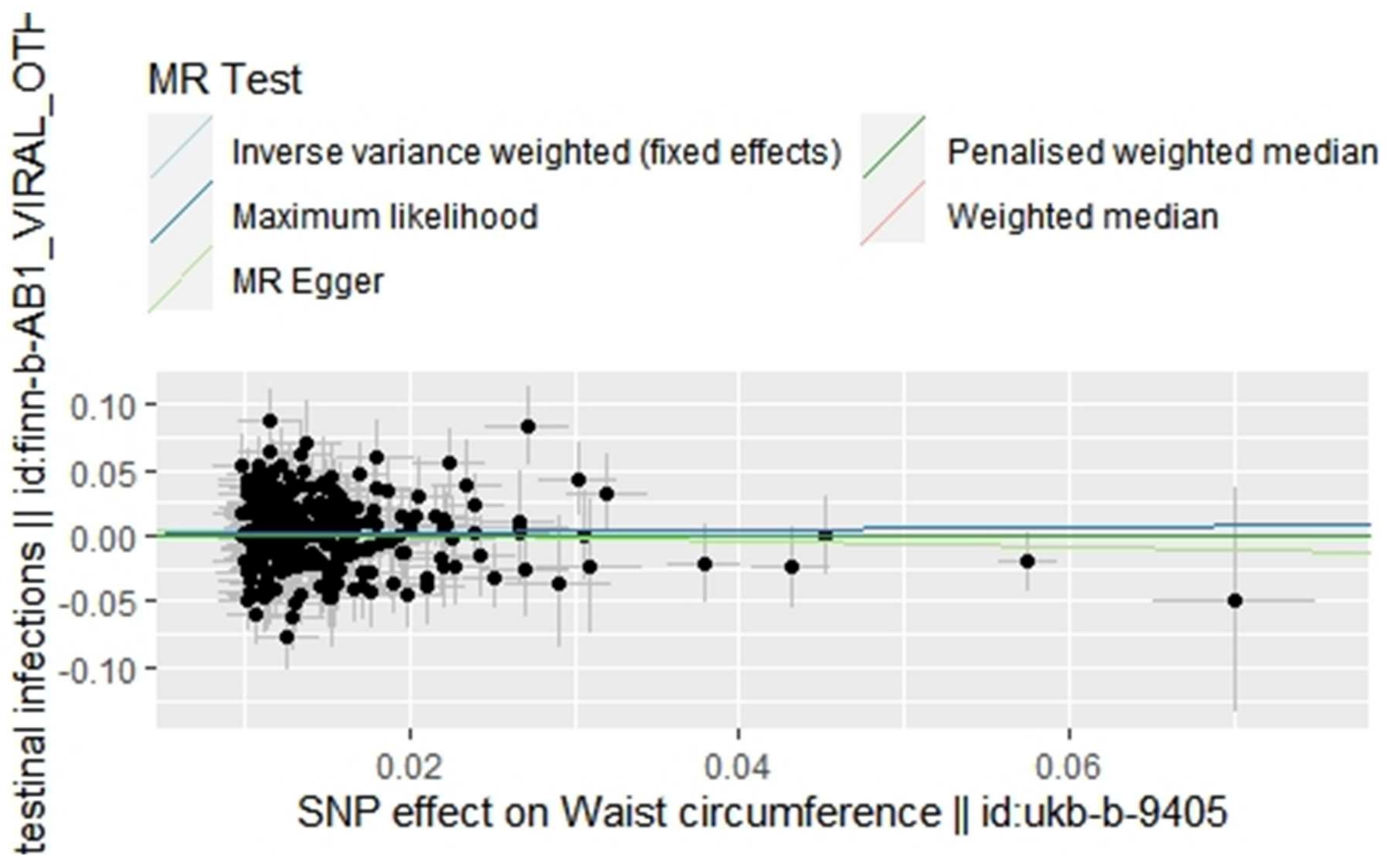

d2

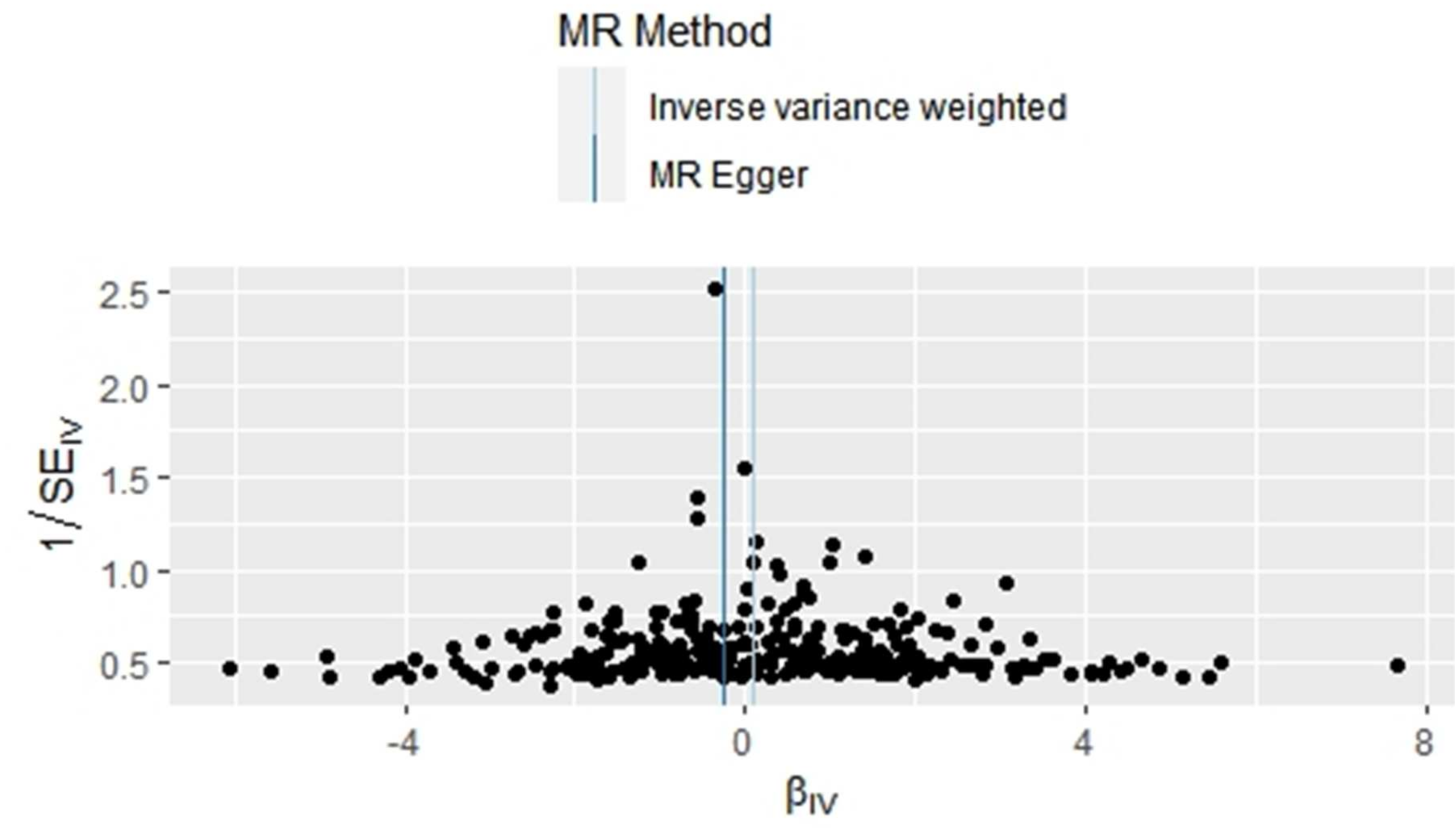

e1

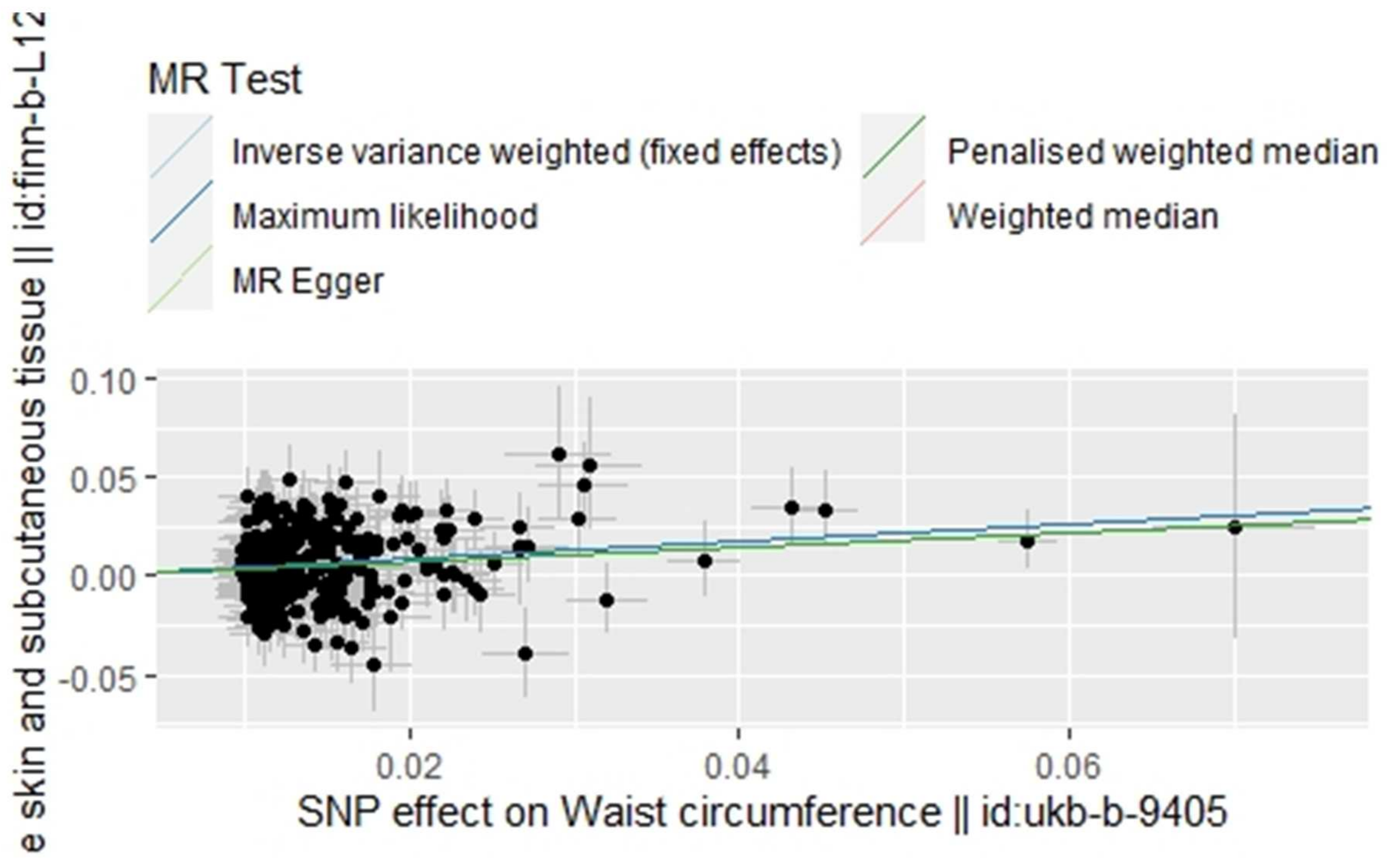

e2

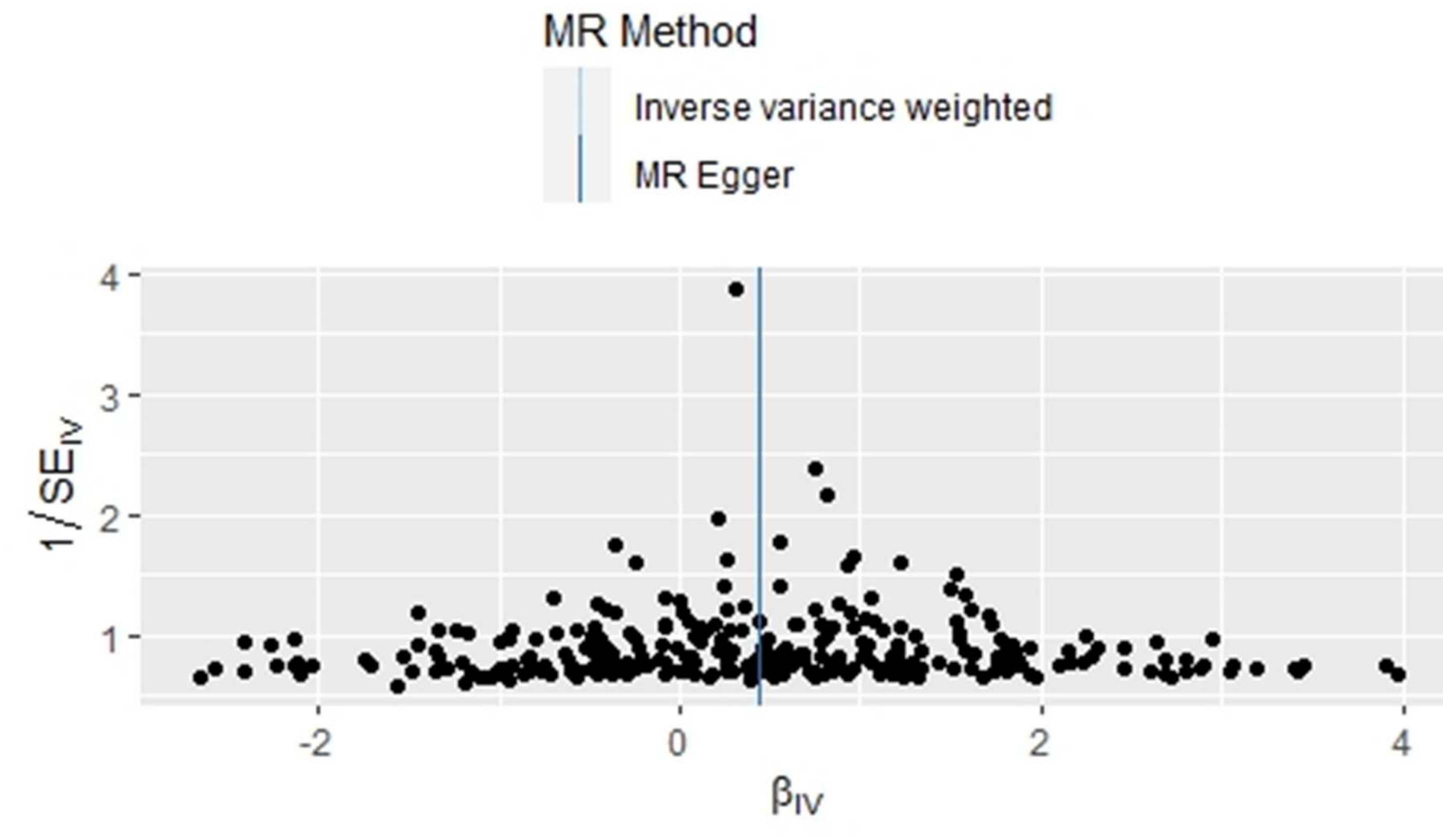

f1

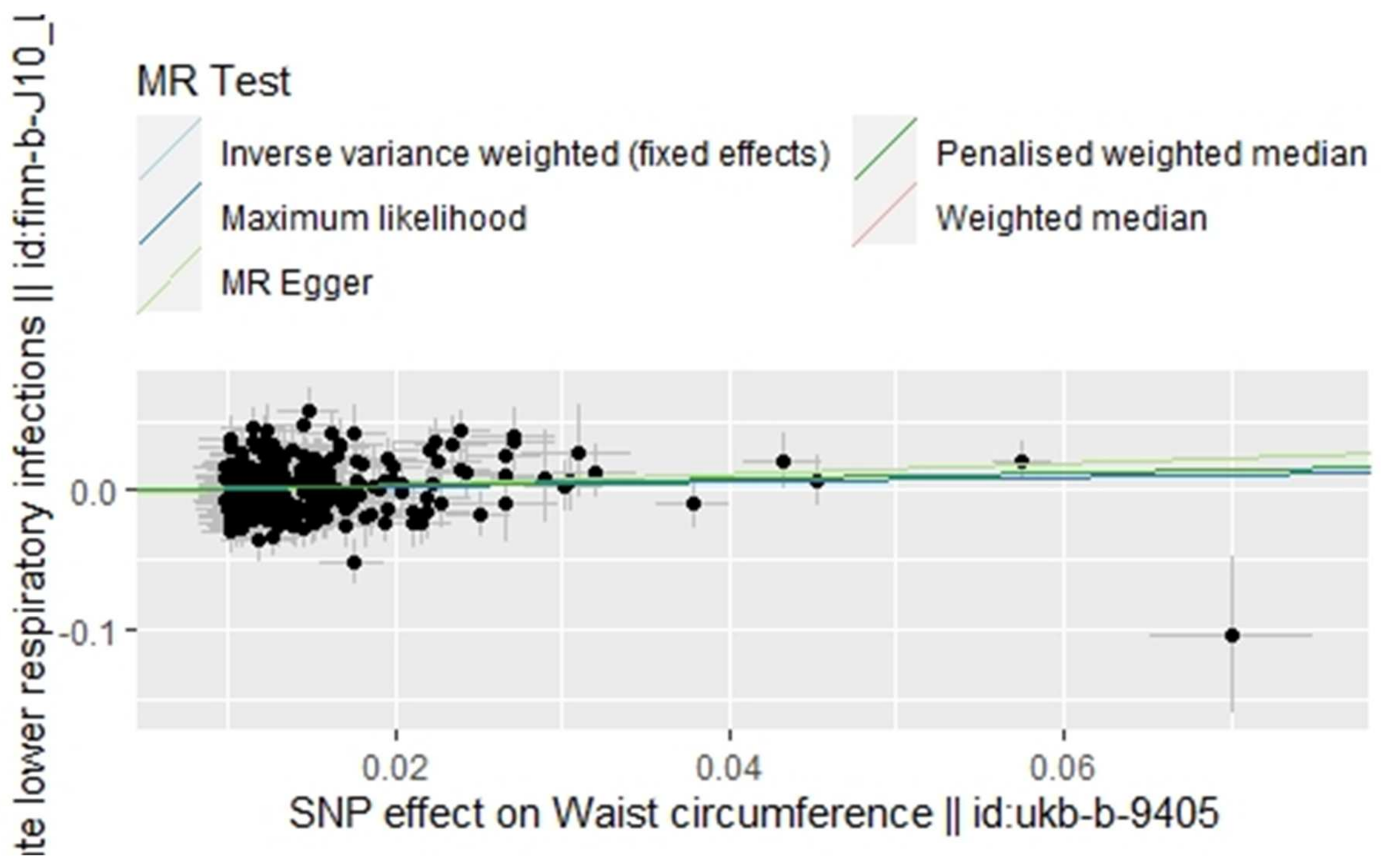

f2

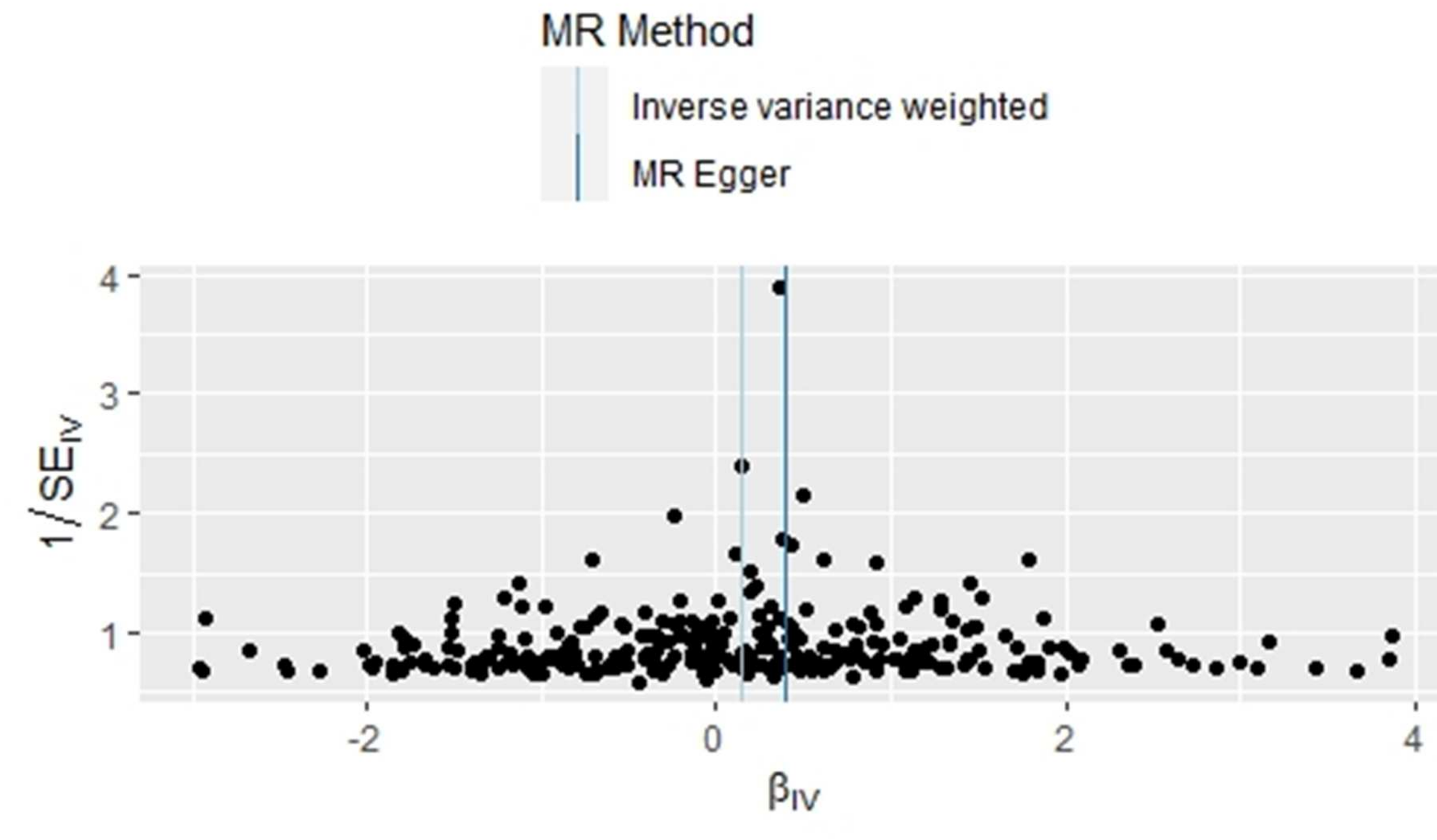

Supplement: Supplementary file 1 [file medi-104-e45775-s001.pdf]
